# Supplementary material for: Electroreductive hydroxy fluorosulfonylation of alkenes
Source: Nat Commun. 2023 Dec 13;14:8278. doi: 10.1038/s41467-023-44029-w (PMC10719349; doi:10.1038/s41467-023-44029-w)
Supplement: Supplementary file 1 — Supplementary Information [file 41467_2023_44029_MOESM1_ESM.pdf]

Supplementary Information for

Electroreductive hydroxy fluorosulfonylation of alkenes

Qingyuan Feng,[1] Tianyu He,[1] Shencheng Qian,[1] Peng Xu,[1] Saihu Liao,[2] and Shenlin Huang[1,*]
[1]Jiangsu Co-Innovation Center of Efficient Processing and Utilization of Forest Resources, International Innovation Center for Forest Chemicals and Materials, Nanjing Forestry University, Nanjing 210037, China
[2] State Key Laboratory of Physical Chemistry of Solid Surfaces, Xiamen University, Xiamen 361005, China
[*]email: shuang@njfu.edu.cn

Table of contents

1. Supplementary methods S1
1.1 General information S1
1.2 The synthesis of substrates S2
1.3 Initial studies for hydroxy fluorosulfonylation S3
1.3.1 Initial trials for the synthesis of B-hydroxy sulfonyl fluoride **3** S3
1.3.2 Reduction of β-keto sulfonyl fluorides(unsuccessful) S3
1.4 Tables of optimization S5
1.5 Electrochemical synthesis of 3, 5-53 .... S9
1.5.1 Experimental procedure A and reaction setup S9
1.5.2 Experimental procedure B S10
1.6 Gram-scale synthesis S11
1.7 Characterization data S12
1.8 Other substrate attempts S29
1.9 Representative derivatizations S30
1.10 Mechanism studies S33
1.11 X-ray crystal data S44
1.12 Preliminary studies for biological activities S46
1.12.1 In vitro antifungal activities S46
1.12.2 In vitro anti-*Bursaphelenchus xylophilus* S50
2. Supplementary NMR spectra S52
3. Supplementary references S137

1. Supplementary methods

1.1 General information

Unless otherwise stated, all glassware was oven dried. All solvents were distilled from appropriate drying agents prior to use. All reagents were used as received from commercial suppliers unless otherwise indicated. All electrolysis reactions were performed in EletraSyn 2.0 setup or domestic dual display DC stabilized power supply (UTP1303 / UTP3305). Reactions were monitored using Thin Layer Chromatography (TLC) carried out on Merck silica gel plates (60F-254), using short-wave UV light and $KMnO_4$ stain as the visualizing agent. Flash column chromatography was performed using silica gel 60 (200-300 mesh). High-resolution mass spectra (HRMS) were recorded on a Bruker MTQ III q-TOF instrument. Gas chromatography (GC) were recorded on an PANNA A91PLUS. All ^{1}H NMR, ^{13}C NMR, ^{19}F NMR spectra were recorded on Bruker DRX-600 and AMX-400 instruments. Chemical shifts were given in parts per million (ppm, δ), referenced to the solvent peak of $CDCl_3$, defined at δ = 7.26 (^{1}H NMR), defined at δ = 77.16 (^{13}C NMR). or ^{d}Acetone, defined at δ = 2.05 (^{1}H NMR), defined at δ = 206.26 (^{13}C NMR). Coupling constants were quoted in Hz (J). ^{1}H NMR Spectroscopy splitting patterns were designated as singlet (s), doublet (d), triplet (t), quartet (q). Splitting patterns that could not be interpreted or easily visualized were designated as multiplet (m) or broad singlet (bs).

$ClSO_2F$ was prepared according to the literature procedure[1].

1.2 The synthesis of substrates

Most of alkenes were purchased from commercial sources. The alkenes for the product **11**, **13-14**, **25**[2], **21**[3], **28**[4], **29**[5], **50**[6] were synthesized according to the reported literature. All the characterization data are consistent with those in the reported literature.

1.3 Initial studies for hydroxy fluorosulfonylation

1.3.1 Initial trials for the synthesis of Β-hydroxy sulfonyl fluoride **3**

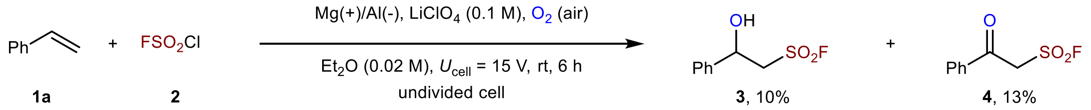

A three necked round bottom flask (25 mL) equipped with one magnesium (anode) plate electrode (10 mm x 10 mm x 1 mm), one aluminum (cathode) wire electrode (10 mm x 1 mm x 1 mm) and a stir bar was charged with $LiClO_4$ (106 mg, 0.1 M), Et_2O (10 mL, 0.02 M) and **1a** (0.2 mmol). After pre-stirring for 1 min, $ClSO_2F$ was added (0.3 mmol, 1.5 eq, 1 M in anhydrous $PhCF_3$). The mixture was electrolyzed at a constant voltage of 15 V for 6 h. Subsequently, the reaction was quenched with water and electrodes were rinsed with EtOAc. The resulting mixture was extracted with EtOAc and the combined organic layers were dried over Na_2SO_4 and concentrated in vacuo. The residue was purified by column chromatography to afford the desired product **3** (4.2 mg, 10%) and **4** (5.4 mg, 13%).

1.3.2 Reduction of β-keto sulfonyl fluorides(unsuccessful)

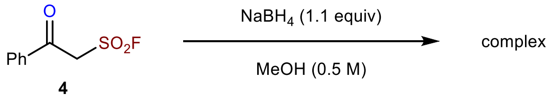

A 4 mL vial with a stir bar was charged compound **4** (40.4 mg, 0.2 mmol), MeOH (0.4 mL, 0.5 M). Then, $NaBH_4$ (8.3 mg, 1.1 equiv) was added, and the reaction mixture was stirred at room temperature for 2 h. The reaction detected by TLC.

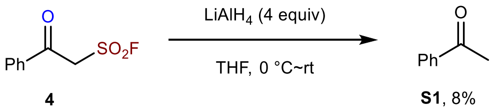

A flask (10 mL) with a stir bar and compound **4** (40.4 mg, 0.2 mmol) was dissolved in anhydrous THF (2 mL, 0.1 M) under nitrogen and cooled to 0 °C. Then, $LiAlH_4$ (32 mg, 4 equiv) was added slowly to the solution. The reaction was allowed to warm to room temperature and stirred overnight. After the reaction was complete, it was placed in the ice bath and quenched with H_2O slowly. Then the mixture was added 1 M HCl, extracted with DCM (3 x 2 mL) The residue was purified by column chromatography to afford acetophenone **S1** (2 mg, 8%).

Acetophenone(**S1**)

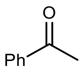

^{1}H NMR (400 MHz, $CDCl_3$) δ 7.96 (d, J = 7.7 Hz, 2H), 7.57 (t, J = 7.2 Hz, 1H), 7.46 (t, J = 7.4 Hz, 2H), 2.61 (s, 3H).

All analytic data match to the reported data[7].

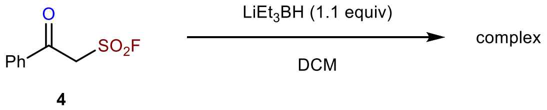

A flask (25 mL) with a stir bar and compound **4** (40.4 mg, 0.2 mmol) was dissolved in anhydrous DCM (6 mL). Then, $LiEt_3BH$ (0.22 mL, 1.0 M in THF, 1.1 equiv) was added slowly to the solution. After the reaction was complete, it was quenched with H_2O slowly. The reaction detected by TLC.

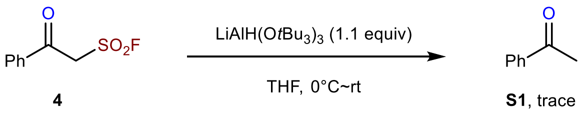

A flask (10 mL) with a stir bar and compound **4** (40.4 mg, 0.2 mmol) was dissolved in anhydrous THF (2 mL, 0.1 M) under nitrogen and cooled to 0 °C. Then, LiAlH(O*t*Bu$_3$)$_3$ (109 mg, 4 equiv) was added slowly to the solution. The reaction was allowed to warm to room temperature and stirred overnight. After the reaction was complete, it was placed in the ice bath and quenched with H_2O slowly. Then the mixture was added 1 M HCl, extracted with DCM (3 x 2 mL) The reaction detected by TLC.

1.4 Tables of optimization

Supplementary Table 1. Electrodes

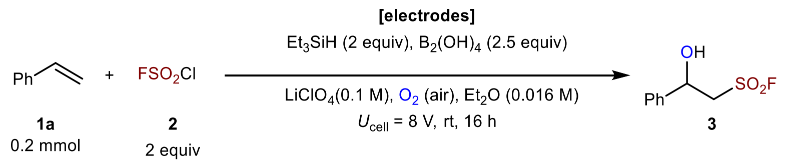

Entry	Anode(+)	Cathode(-)	**3**(%)[a]
1	Mg	Al	10
2	Zn	Al	32
3	Zn	Zn	0
4	Al	Al	38
5	Al	Pb	0
6	C	C	0
7	Al	Zn	**96**
8	GF	Zn	75
9	C	Zn	73
10	Pt	Zn	65

a. isolated yields.

GF = graphite felt.

Supplementary Table 2. Electrolytes

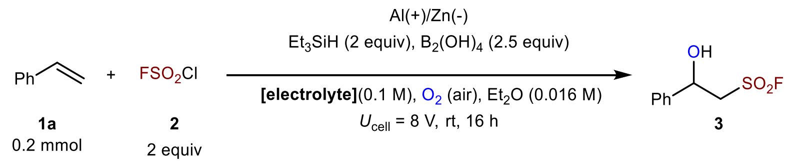

Entry	Electrolyte	**3**(%)[a]
1	$LiClO_4$	**96**
2	$NaClO_4$	74
3	$LiBF_4$	42
4	LiBr	0
5	Et_4NPF_6	<10

a. isolated yields.

Supplementary Table 3. Solvents

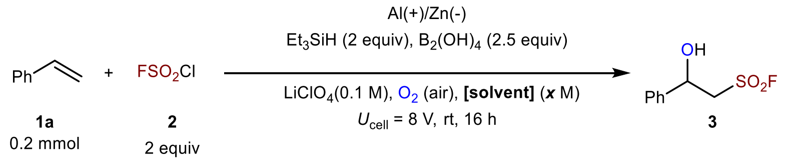

Entry	Solvent	(*x* M)	**3**(%)[a]
1	Et_2O	0.04	28
2	Et_2O	0.02	77
3	Et_2O	0.016	**96**
4	Et_2O	0.013	73
5	Et_2O	0.01	35
6	THF	0.016	27
7	1,4-dioxane	0.016	49
8	DCM	0.016	0
9	MeCN	0.016	0
10	HFIP	0.016	0
11	TFE	0.016	0

a. isolated yields.

Supplementary Table 4. “H-donor” sources

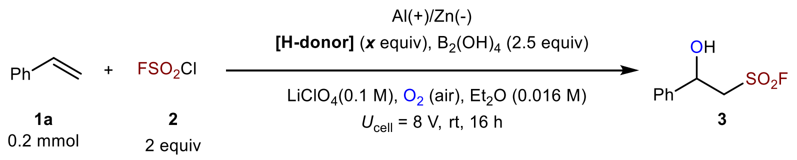

Entry	H-donor	(*x* equiv)	**3**(%)[a]
1	---	0	74
2	Et_3SiH	0.5	79
3	Et_3SiH	1.0	88
4	Et_3SiH	2.0	**96**
5	Et_3SiH	3.0	95
6	$PhSiH_3$	2.0	40
7	$(EtO)_3SiH$	2.0	51
8	$^tBuMe_2SiH$	2.0	70

a. isolated yields.

Supplementary Table 5. B sources

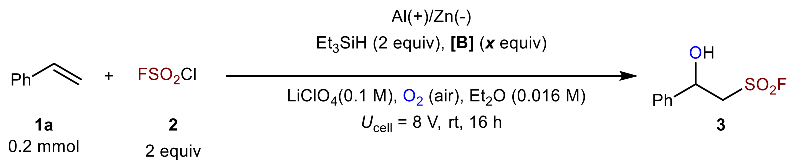

Entry	[B]	(*x* equiv)	**3**(%)[a]
1	---	0	69
1	$B_2(OH)_4$	1.0	78
2	$B_2(OH)_4$	1.5	85
3	$B_2(OH)_4$	2.5	**96**
4	$B_2(OH)_4$	3.0	84
5	Et_3B	2.5	0
6	$BF_3{\cdot}Et_2O$	2.5	60
7	$BH_3{\cdot}THF$	2.5	0
8	$B(C_6F_5)_3$	20%	76

a. isolated yields.

Supplementary Table 6. Cell voltages

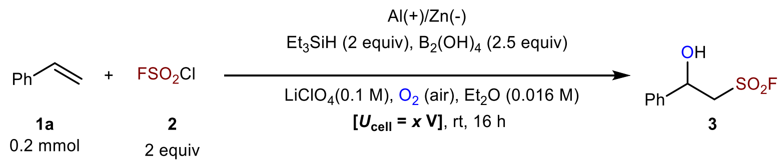

Entry	U_{cell} (*x* V)	**3**(%)[a]
1	4	85
2	8	**96**
3	12	93
4	16	87

a. isolated yields.

Supplementary Table 7. Time

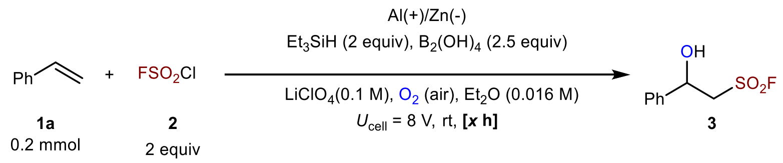

Entry	Time (*x* h)	**3**(%)[a]
1	4	51
2	8	66
3	12	86
4	16	**96**

a. isolated yields.

Supplementary Table 8. Control experiments

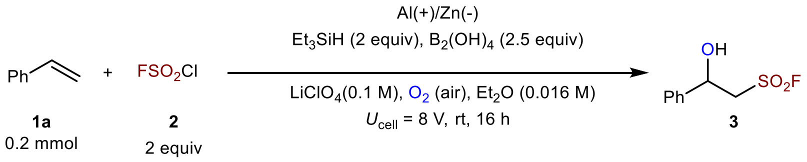

Entry	Conditions	**3**(%)[a]
1	under nitrogen	0
2	no electrolysis	78
3	no Et_3SiH	74
4	no $B_2(OH)_4$	69

a. isolated yields.

1.5 Electrochemical synthesis of 3, 5-53

1.5.1 Experimental procedure A and reaction setup

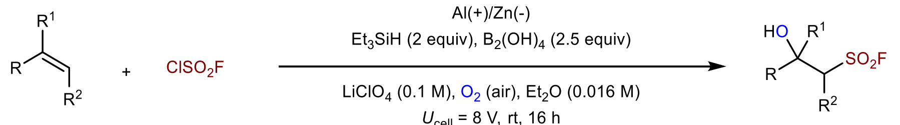

1 (0.2 mmol) **2** (2 equiv) **3, 5-53**

A 20-mL vial with one aluminum (anode) plate electrode (10 mm x 15 mm x 1 mm), one zinc (cathode) plate electrode (10 mm x 15 mm x 1 mm) and a stir bar was charged with $LiClO_4$ (130 mg, 0.1 M), Et_2O (12 mL, 0.016M), $B_2(OH)_4$ (0.5 mmol, 2.5 equiv), Et_3SiH (0.4 mmol, 2 equiv) and **1** alkenes (0.2 mmol). Then, $ClSO_2F$ was added (0.4 mmol, 2 eq, 1 M in anhydrous $PhCF_3$). The mixture was electrolyzed at a constant cell voltage of 8 V for 16 h under an atmosphere of air (1 atm, balloon). Subsequently, the reaction (Figure S1) was quenched with water and electrodes were rinsed with EtOAc. The resulting mixture was extracted with EtOAc and the combined organic layers were dried over Na_2SO_4 and concentrated in vacuo. The residue was purified by column chromatography to afford the desired product (PE/DCM/EA = 20/20/1 ~ 2/2/1).

Graphical guide for the 0.2 mmol reaction setup

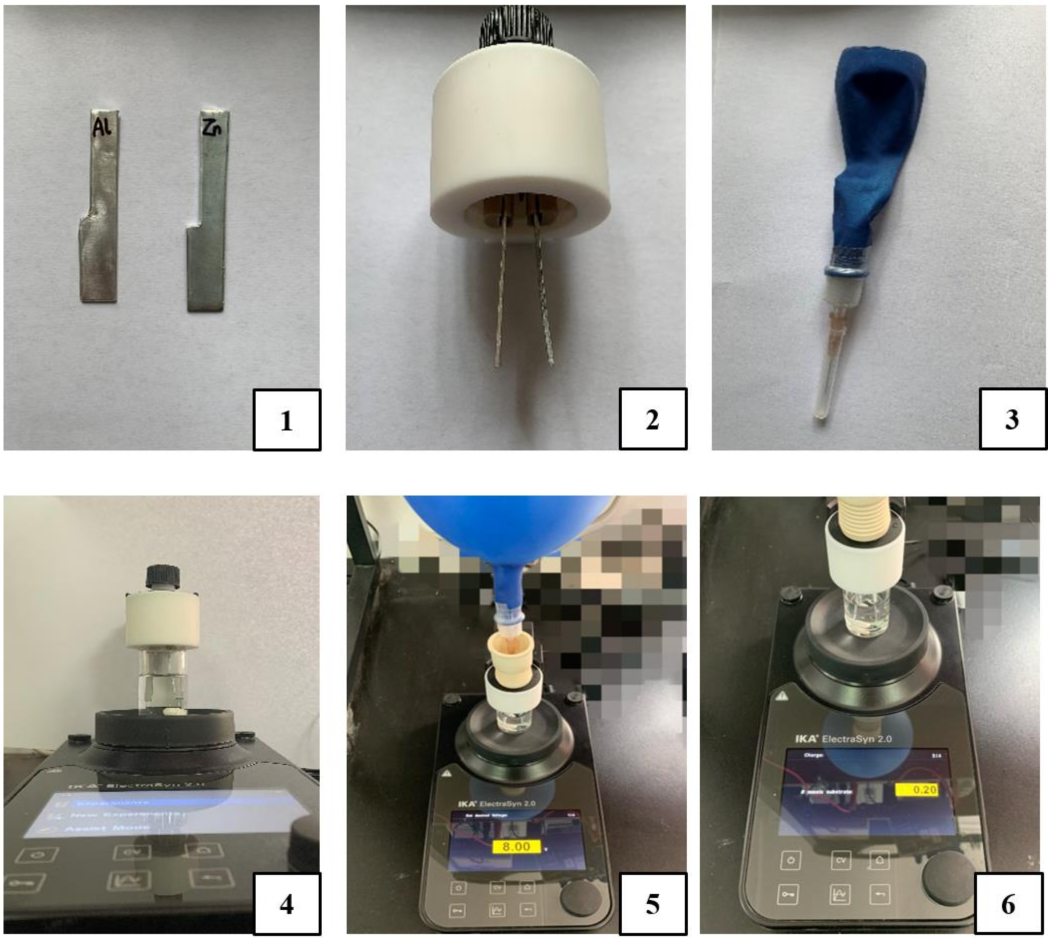

Supplementary Figure 1. (1 and 2) aluminum (anode) plate electrode (10 mm x 15 mm x 1 mm), one zinc (cathode) plate electrode (10 mm x 15 mm x 1 mm)); (3) air ball; (4-6) standard reaction unit.

1.5.2 Experimental procedure B

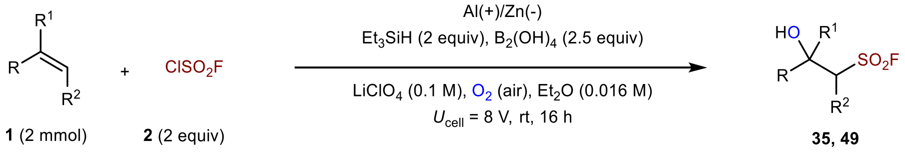

A beaker (250 mL) with one aluminum (anode) plate electrode (40 mm x 50 mm x 1 mm), one zinc (cathode) plate electrode (40 mm x 50 mm x 1 mm) and a stir bar was charged with $LiClO_4$ (1.3 g, 0.1 M), Et_2O (120 mL, 0.016M), $B_2(OH)_4$ (5 mmol, 2.5 equiv), Et_3SiH (4 mmol, 2 equiv) and **1** alkenes (2.0 mmol). Then, $ClSO_2F$ was added (4.0 mmol, 2 eq, 1 M in anhydrous $PhCF_3$). The mixture was electrolyzed at a constant cell voltage of 8 V for 16 h under an atmosphere of air (1 atm, balloon). Subsequently, the reaction was quenched with water and electrodes were rinsed with EtOAc. The resulting mixture was extracted with EtOAc and the combined organic layers were dried over Na_2SO_4 and concentrated in vacuo. The residue was purified by column chromatography to afford the desired product (PE/DCM/EA = 20/20/1 ~ 2/2/1).

1.6 Gram-scale synthesis

Typical procedure for gram-scale synthesis for **3**:

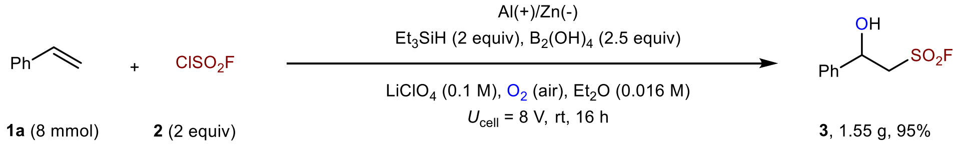

A beaker (500 mL) (as shown below) with one aluminum (anode) plate electrode (50 mm x 100 mm x 1 mm), one zinc (cathode) plate electrode (50 mm x 100 mm x 1 mm), and a stir bar was charged with $LiClO_4$ (5.2 g, 0.1 M), anhydrous Et_2O (480 mL, 0.016 M), $B_2(OH)_4$ (20 mmol, 2.5 equiv), Et_3SiH (16 mmol, 2 equiv) and **1a** (8 mmol). Then, $ClSO_2F$ was added (16 mmol, 2 eq, 1 M in anhydrous $PhCF_3$). The mixture was electrolyzed at a constant cell voltage of 8 V for 16 h. Subsequently, the reaction was quenched with water and electrodes were rinsed with EtOAc. The resulting mixture was extracted with EtOAc and the combined organic layers were dried over Na_2SO_4 and concentrated in vacuo. The residue was purified by column chromatography to afford the desired product. (PE/DCM/EA = 20/20/1).

Graphical guide for the 8 mmol scale reaction setup

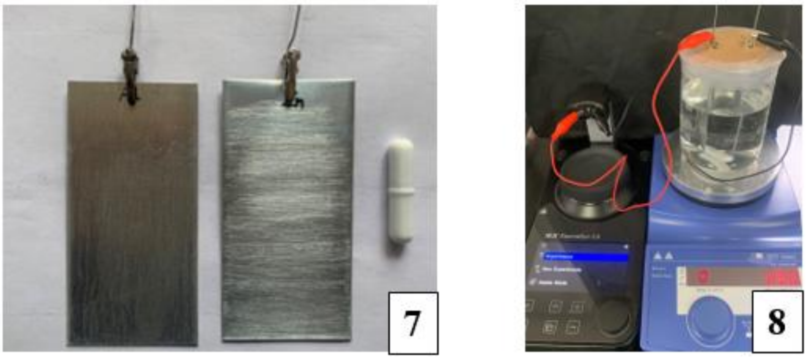

Supplementary Figure 2. (7) aluminum (anode) plate electrode (50 mm x 100 mm x 1 mm), one zinc (cathode) plate electrode (50 mm x 100 mm x 1 mm)); (8) standard reaction unit.

1.7 Characterization data

2-Hydroxy-2-phenylethane-1-sulfonyl fluoride (3)

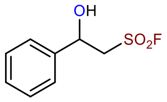

Prepared according to procedure A using styrene (23.2 μL, 0.20 mmol).
Flash chromatography: PE/DCM/EA = 20/20/1
39.2 mg, 96% yield, colorless oil.
^{1}H NMR (600 MHz, $CDCl_3$) δ 7.43 – 7.36 (m, 5H), 5.37 (dd, *J* = 9.8, 2.4 Hz, 1H), 3.83 – 3.77 (m, 1H), 3.64 (ddd, *J* = 14.9, 4.0, 2.5 Hz, 1H), 2.86 (bs, 1H).
^{13}C NMR (150 MHz, $CDCl_3$) δ 139.7 (d, *J* = 1.5 Hz), 129.28, 129.26, 125.8, 69.1, 58.9 (d, *J* = 12.9 Hz).
^{19}F NMR (565 MHz, $CDCl_3$) δ 59.41 (t, *J* = 4.0 Hz).
HRMS-ESI (m/z) $[M+Na]^+$ calculated for $C_8H_9FNaO_3S$ 227.0149, found 227.0156.

2-(4-Bromophenyl)-2-hydroxyethane-1-sulfonyl fluoride (5)

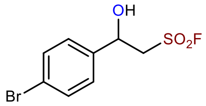

Prepared according to procedure A using 1-bromo-4-vinylbenzene (27.0 μL, 0.20 mmol).
Flash chromatography: PE/DCM/EA = 20/20/1
37.8 mg, 67% yield, colorless oil.
^{1}H NMR (400 MHz, $CDCl_3$) δ 7.55 (d, *J* = 8.4 Hz, 2H), 7.29 (d, *J* = 8.4 Hz, 2H), 5.34 (dd, *J* = 9.6, 2.4 Hz, 1H), 3.76 (ddd, *J* = 14.1, 9.6, 4.4 Hz, 1H), 3.61 (ddd, *J* = 14.9, 3.6, 2.7 Hz, 1H), 2.84 (bs, 1H).
^{13}C NMR (100 MHz, $CDCl_3$) δ 138.6 (d, *J* = 1.8 Hz), 132.5, 127.5, 123.3, 68.6, 58.8 (d, *J* = 13.3 Hz).
^{19}F NMR (377 MHz, $CDCl_3$) δ 59.46 (t, *J* = 4.1 Hz).
HRMS-ESI (m/z) $[2M+Na]^+$ calculated for $C_{16}H_{16}Br_2F_2NaO_6S_2$ 586.8615, found 586.8641.

2-(4-Chlorophenyl)-2-hydroxyethane-1-sulfonyl fluoride (6)

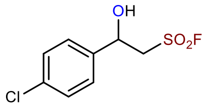

Prepared according to procedure A using 1-chloro-4-vinylbenzene (24.7 μL, 0.20 mmol).
Flash chromatography: PE/DCM/EA = 20/20/1
21.9 mg, 46% yield, colorless oil.
^{1}H NMR (600 MHz, $CDCl_3$) δ 7.36 (d, *J* = 20.9 Hz, 4H), 5.35 (d, *J* = 5.0 Hz, 1H), 3.89 – 3.45 (m, 2H), 2.93 (bs, 1H).
^{13}C NMR (150 MHz, $CDCl_3$) δ 138.1 (d, *J* = 1.6 Hz), 135.1, 129.5, 127.2 68.5, 58.8 (d, *J* = 13.5 Hz).
^{19}F NMR (565 MHz, $CDCl_3$) δ 59.48 (t, *J* = 4.0 Hz).
HRMS-ESI (m/z) $[M-H]^-$ calculated for $C_8H_7ClFO_3S$ 236.9794, found 236.9796.

2-(4-Fluorophenyl)-2-hydroxyethane-1-sulfonyl fluoride (7)

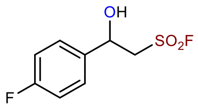

Prepared according to procedure A using 1-fluoro-4-vinylbenzene (24.1 μL, 0.20 mmol).

Flash chromatography: PE/DCM/EA = 20/20/1

19.1 mg, 43% yield, colorless oil.

^{1}H NMR (600 MHz, $CDCl_3$) δ 7.39 (dd, J = 8.6, 5.2 Hz, 2H), 7.10 (t, J = 8.6 Hz, 2H), 5.35 (dd, J = 9.7, 2.5 Hz, 1H), 3.81 – 3.73 (m, 1H), 3.64 – 3.58 (m, 1H), 2.70 (bs, 1H).

^{13}C NMR (150 MHz, $CDCl_3$) δ 163.1 (d, J = 248.4 Hz), 135.6 (dd, J = 3.1, 1.7 Hz), 127.7 (d, J = 8.4 Hz), 116.3 (d, J = 21.8 Hz), 68.5, 59.0 (d, J = 13.2 Hz).

^{19}F NMR (565 MHz, $CDCl_3$) δ 59.45 (t, J = 4.5 Hz), -112.17 (ddd, J = 14.3, 9.0, 5.4 Hz).

HRMS-ESI (m/z) $[M+H]^+$ calculated for $C_8H_9F_2O_3S$ 223.0235, found 223.0235.

2-(3-Bromophenyl)-2-hydroxyethane-1-sulfonyl fluoride (8)

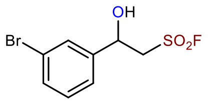

Prepared according to procedure A using 1-bromo-3-vinylbenzene (26.8 μL, 0.20 mmol).

Flash chromatography: PE/DCM/EA = 20/20/1

36.1 mg, 64% yield, colorless oil.

^{1}H NMR (600 MHz, $CDCl_3$) δ 7.58 (s, 1H), 7.50 (d, J = 7.8 Hz, 1H), 7.33 (d, J = 7.7 Hz, 1H), 7.29 (t, J = 7.8 Hz, 1H), 5.34 (dd, J = 9.8, 2.0 Hz, 1H), 3.76 (ddd, J = 14.1, 9.8, 4.1 Hz, 1H), 3.65 – 3.60 (m, 1H), 2.68 (bs, 1H).

^{13}C NMR (150 MHz, $CDCl_3$) δ 141.8 (d, J = 1.7 Hz), 132.4, 130.8, 129.0, 124.4, 123.3, 68.4, 58.9 (d, J = 13.4 Hz).

^{19}F NMR (565 MHz, $CDCl_3$) δ 59.53 (t, J = 4.0 Hz).

HRMS-ESI (m/z) $[M-H]^-$ calculated for $C_8H_7BrFO_3S$ 280.9289, found 280.9290.

2-(2-Bromophenyl)-2-hydroxyethane-1-sulfonyl fluoride (9)

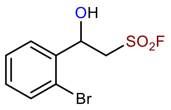

Prepared according to procedure A using 1-bromo-2-vinylbenzene (25.9 μL, 0.20 mmol).

Flash chromatography: PE/DCM/EA = 20/20/1

44.0 mg, 78% yield, colorless oil.

^{1}H NMR (600 MHz, $CDCl_3$) δ 7.68 (dd, J = 7.8, 1.5 Hz, 1H), 7.57 (dd, J = 8.0, 1.1 Hz, 1H), 7.41 (td, J = 7.6, 1.0 Hz, 1H), 7.24 (td, J = 7.7, 1.7 Hz, 1H), 5.68 (dd, J = 9.9, 1.6 Hz, 1H), 3.83 (ddd, J = 14.9, 3.6, 1.8 Hz, 1H), 3.59 (ddd, J = 15.0, 9.9, 5.2 Hz, 1H), 3.17 (bs, 1H).

^{13}C NMR (100 MHz, $CDCl_3$) δ 138.5, 133.2, 130.5, 128.4, 127.5, 121.2, 68.1, 57.3 (d, J = 13.4 Hz).

^{19}F NMR (377 MHz, $CDCl_3$) δ 59.22.

HRMS-ESI (m/z) $[M-H]^-$ calculated for $C_8H_7BrFO_3S$ 280.9289, found 280.9288.

2-Hydroxy-2-(4-(trifluoromethyl)phenyl)ethane-1-sulfonyl fluoride (10)

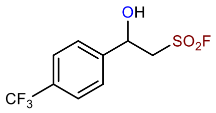

Prepared according to procedure A using 1-(trifluoromethyl)-4-vinylbenzene (30.2 μL, 0.20 mmol).

Flash chromatography: PE/DCM/EA = 20/20/1

49.0 mg, 90% yield, yellow solid.

^{1}H NMR (600 MHz, $CDCl_3$) δ 7.68 (d, J = 8.2 Hz, 1H), 7.55 (d, J = 8.1 Hz, 1H), 5.45 (dd, J = 9.7, 2.1 Hz, 1H), 3.82

– 3.74 (m, 1H), 3.65 (ddd, *J* = 14.9, 3.6, 2.7 Hz, 1H), 3.18 (bs, 1H).
^{13}C NMR (150 MHz, $CDCl_3$) δ 143.5, 131.5 (q, *J* = 32.7 Hz), 126.3 (q, *J* = 7.5, 3.8 Hz), 126.2, 123.9 (q, *J* = 272.1 Hz), 68.5, 58.8 (d, *J* = 13.5 Hz).
^{19}F NMR (565 MHz, $CDCl_3$) δ 59.62 (t, *J* = 3.9 Hz), -62.77.
HRMS-ESI (m/z) $[M+NH_4]^+$ calculated for $C_9H_{12}F_4NO_3S$ 290.0469, found 290.0475.

2-(4-Formylphenyl)-2-hydroxyethane-1-sulfonyl fluoride (11)

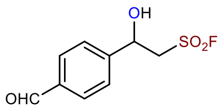

Prepared according to procedure A using 4-vinylbenzaldehyde (27 mg, 0.20 mmol).
Flash chromatography: PE/DCM/EA = 5/5/1
26.1 mg, 56% yield, white solid.
^{1}H NMR (600 MHz, $CDCl_3$) δ 10.03 (s, 1H), 7.94 (d, *J* = 8.2 Hz, 2H), 7.61 (d, *J* = 8.1 Hz, 2H), 5.47 (d, *J* = 9.2 Hz, 1H), 3.79 (ddd, *J* = 14.3, 9.7, 4.4 Hz, 1H), 3.71 – 3.62 (m, 1H), 3.13 (bs, 1H).
^{13}C NMR (150 MHz, $CDCl_3$) δ 191.7, 145.9 (d, *J* = 1.4 Hz), 136.9, 130.6, 126.5, 68.7, 58.8 (d, *J* = 13.6 Hz).
^{19}F NMR (565 MHz, $CDCl_3$) δ 59.63 (t, *J* = 4.1 Hz).
HRMS-ESI (m/z) $[M-H]^-$ calculated for $C_9H_8FO_4S$ 231.0133, found 231.0122.

Methyl 4-(2-(fluorosulfonyl)-1-hydroxyethyl)benzoate (12)

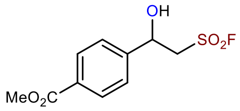

Prepared according to procedure A using methyl 4-vinylbenzoate (33.4 mg, 0.20 mmol).
Flash chromatography: PE/DCM/EA = 10/10/1
21.0 mg, 40% yield, white solid.
^{1}H NMR (600 MHz, $CDCl_3$) δ 8.05 (d, *J* = 8.3 Hz, 2H), 7.48 (d, *J* = 8.3 Hz, 2H), 5.43 (dd, *J* = 9.7, 2.3 Hz, 1H), 3.92 (s, 3H), 3.82 – 3.75 (m, 1H), 3.65 (m, 1H), 3.18 (bs, 1H).
^{13}C NMR (150 MHz, $CDCl_3$) δ 166.6, 144.5, 131.0, 130.5, 125.8, 68.7, 58.8 (d, *J* = 13.4 Hz), 52.5.
^{19}F NMR (565 MHz, $CDCl_3$) δ 59.62 (t, *J* = 3.9 Hz).
HRMS-ESI (m/z) $[M-H]^-$ calculated for $C_{10}H_{10}FO_5S$ 261.0238, found 261.0231.

2-Hydroxy-2-(4-methyl-3-nitrophenyl)ethane-1-sulfonyl fluoride (13)

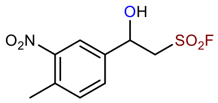

Prepared according to procedure A using 1-methyl-2-nitro-4-vinylbenzene (33.3 mg, 0.20 mmol).
Flash chromatography: PE/DCM/EA = 20/20/1
30.0 mg, 57% yield, yellow oil.
^{1}H NMR (600 MHz, $CDCl_3$) δ 8.04 (s, 1H), 7.56 (d, *J* = 7.8 Hz, 1H), 7.41 (d, *J* = 7.9 Hz, 1H), 5.44 (d, *J* = 8.0 Hz, 1H), 3.85 – 3.76 (m, 1H), 3.68 (d, *J* = 14.9 Hz, 1H), 2.60 (s, 3H).
^{13}C NMR (150 MHz, $CDCl_3$) δ 149.5, 139.1 (d, *J* = 1.4 Hz), 134.5, 133.8, 130.2, 122.1, 67.9, 58.6 (d, *J* = 13.6 Hz), 20.3.
^{19}F NMR (565 MHz, $CDCl_3$) δ 59.88 (t, *J* = 3.4 Hz).

HRMS-ESI (m/z) $[M+NH_4]^+$ calculated for $C_9H_{14}FN_2O_5S$ 281.0602, found 281.0604.

2-(3-Cyano-4-methylphenyl)-2-hydroxyethane-1-sulfonyl fluoride (14)

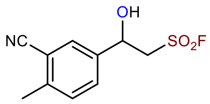

Prepared according to procedure A using 2-methyl-5-vinylbenzonitrile (29.2 mg, 0.20 mmol).
Flash chromatography: PE/DCM/EA = 20/20/1
30.1 mg, 62% yield, colorless oil.
^{1}H NMR (600 MHz, $CDCl_3$) δ 7.68 (d, J = 1.5 Hz, 1H), 7.52 (dd, J = 8.0, 1.7 Hz, 1H), 7.38 (d, J = 8.0 Hz, 1H), 5.38 (dd, J = 9.5, 2.5 Hz, 1H), 3.81 – 3.73 (m, 1H), 3.66 – 3.60 (m, 1H), 3.28 (bs, 1H), 2.55 (s, 3H).
^{13}C NMR (150 MHz, $CDCl_3$) δ 143.0, 138.3 (d, J = 1.6 Hz), 131.2, 130.1, 129.9, 117.6, 113.6, 68.0, 58.7 (d, J = 13.5 Hz), 20.4.
^{19}F NMR (565 MHz, $CDCl_3$) δ 59.80 (t, J = 3.8 Hz).
HRMS-ESI (m/z) $[M+K]^+$ calculated for $C_{10}H_{10}FKNO_3S$ 281.9997, found 281.9996.

2-Hydroxy-2-(p-tolyl)ethane-1-sulfonyl fluoride (15)

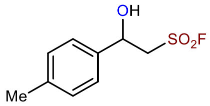

Prepared according to procedure A using 1-methyl-4-vinylbenzene (27.5 μL, 0.20 mmol).
Flash chromatography: PE/DCM/EA = 20/20/1
31.7 mg, 73% yield, colorless oil.
^{1}H NMR (600 MHz, $CDCl_3$) δ 7.27 (d, J = 7.5 Hz, 2H), 7.21 (d, J = 7.2 Hz, 2H), 5.30 (d, J = 9.2 Hz, 1H), 3.85 – 3.71 (m, 1H), 3.59 (d, J = 14.6 Hz, 1H), 2.82 (bs, 1H), 2.36 (s, 3H).
^{13}C NMR (150 MHz, $CDCl_3$) δ 139.3, 136.8 (d, J = 1.5 Hz), 129.9, 125.7, 69.0, 58.9 (d, J = 12.7 Hz), 21.3.
^{19}F NMR (565 MHz, $CDCl_3$) δ 59.32 (t, J = 3.9 Hz).
HRMS-ESI (m/z) $[M+Na]^+$ calculated for $C_9H_{11}FNaO_3S$ 241.0305, found 241.0304.

2-(4-(*tert*-Butyl)phenyl)-2-hydroxyethane-1-sulfonyl fluoride (16)

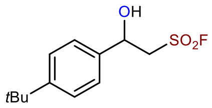

Prepared according to procedure A using 1-(*tert*-butyl)-4-vinylbenzene (39.4 μL, 0.20 mmol).
Flash chromatography: PE/DCM/EA = 20/20/1
33.0 mg, 63% yield, colorless oil.
^{1}H NMR (600 MHz, $CDCl_3$) δ 7.46 – 7.41 (m, 2H), 7.33 (d, J = 8.3 Hz, 2H), 5.34 (dd, J = 9.9, 2.3 Hz, 1H), 3.80 (ddd, J = 14.7, 9.9, 3.6 Hz, 1H), 3.62 (ddd, J = 14.9, 4.2, 2.5 Hz, 1H), 1.33 (s, 9H).
^{13}C NMR (150 MHz, $CDCl_3$) δ 152.5, 136.8 (d, J = 1.5 Hz), 126.2, 125.5, 69.0, 58.9 (d, J = 12.7 Hz), 34.8, 31.4.
^{19}F NMR (565 MHz, $CDCl_3$) δ 59.35 (t, J = 4.0 Hz).
HRMS-ESI (m/z) $[M+Na]^+$ calculated for $C_{12}H_{17}FNaO_3S$ 283.0775, found 283.0782.

2-Hydroxy-2-(*m*-tolyl)ethane-1-sulfonyl fluoride (17)

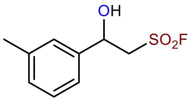

Prepared according to procedure A using 1-methyl-3-vinylbenzene (26.8 µL, 0.20 mmol).
Flash chromatography: PE/DCM/EA = 20/20/1
21.8 mg, 50% yield, colorless oil.
^{1}H NMR (600 MHz, $CDCl_3$) δ 7.30 (t, *J* = 7.6 Hz, 1H), 7.22 (s, 1H), 7.19 (dd, *J* = 7.4, 2.0 Hz, 2H), 5.33 (dd, *J* = 9.8, 2.1 Hz, 1H), 3.79 (ddd, *J* = 14.1, 9.9, 4.0 Hz, 1H), 3.63 (ddd, *J* = 14.9, 3.9, 2.5 Hz, 1H), 2.38 (s, 3H).
^{13}C NMR (100 MHz, $CDCl_3$) δ 139.7 (d, *J* = 1.3 Hz), 139.2, 130.0, 129.2, 126.4, 122.8, 69.2, 59.0 (d, *J* = 12.8 Hz), 21.5.
^{19}F NMR (565 MHz, $CDCl_3$) δ 59.27 (t, *J* = 4.0 Hz).
HRMS-ESI (m/z) $[M+Na]^+$ calculated for $C_9H_{11}FNaO_3S$ 241.0305, found 241.0307.

2-Hydroxy-2-mesitylethane-1-sulfonyl fluoride (18)

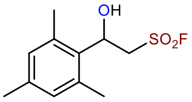

Prepared according to procedure A using 1,3,5-trimethyl-2-vinylbenzene (34.0 µL, 0.20 mmol).
Flash chromatography: PE/DCM/EA = 20/20/1
24.0 mg, 49% yield, colorless oil.
^{1}H NMR (600 MHz, $CDCl_3$) δ 6.86 (s, 2H), 5.80 (dd, *J* = 9.8, 2.5 Hz, 1H), 4.11 (ddd, *J* = 14.9, 9.8, 4.0 Hz, 1H), 3.54 (ddd, *J* = 15.0, 4.6, 2.5 Hz, 1H), 2.43 (s, 6H), 2.26 (s, 3H).
^{13}C NMR (150 MHz, $CDCl_3$) δ 138.6, 136.1 (d, *J* = 0.8 Hz), 132.2, 130.7, 66.0, 56.3 (d, *J* = 12.5 Hz), 20.9, 20.6.
^{19}F NMR (565 MHz, $CDCl_3$) δ 58.15 (t, *J* = 4.3 Hz).
HRMS-ESI (m/z) $[M-H]^-$ calculated for $C_{11}H_{14}FO_3S$ 245.0653, found 245.0649.

2-Hydroxy-2-(3-methoxyphenyl)ethane-1-sulfonyl fluoride (19)

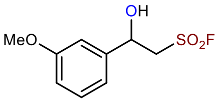

Prepared according to procedure A using 1-methoxy-3-vinylbenzene (28.6 µL, 0.20 mmol).
Flash chromatography: PE/DCM/EA = 20/20/1
21.1 mg, 45% yield, colorless oil.
^{1}H NMR (600 MHz, $CDCl_3$) δ 7.32 (t, *J* = 8.2 Hz, 1H), 6.97 – 6.94 (m, 2H), 6.91 – 6.88 (m, 1H), 5.34 (dd, *J* = 9.8, 2.3 Hz, 1H), 3.82 (s, 3H), 3.78 (ddd, *J* = 14.8, 9.9, 3.7 Hz, 1H), 3.63 (ddd, *J* = 14.9, 4.2, 2.4 Hz, 1H), 2.88 (bs, 1H).
^{13}C NMR (150 MHz, $CDCl_3$) δ 160.3, 141.3 (d, J = 1.4 Hz), 130.4, 117.8, 114.7, 111.3, 69.1, 59.0 (d, *J* = 12.9 Hz), 55.5.
^{19}F NMR (565 MHz, $CDCl_3$) δ 59.39 (t, *J* = 3.9 Hz).
HRMS-ESI (m/z) $[M+K]^+$ calculated for $C_9H_{11}FKO_4S$ 272.9994, found 273.0004.

4-(2-(Fluorosulfonyl)-1-hydroxyethyl)phenyl acetate (20)

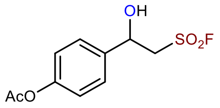

Prepared according to procedure A using 4-vinylphenyl acetate (33.1 mg, 0.20 mmol).
Flash chromatography: PE/DCM/EA = 20/20/1
32.0 mg, 61% yield, colorless oil.
^{1}H NMR (600 MHz, $CDCl_3$) δ 7.40 (d, J = 8.5 Hz, 2H), 7.13 – 7.09 (m, 2H), 5.33 (dd, J = 9.8, 2.4 Hz, 1H), 3.75 (ddd, J = 14.7, 9.8, 3.4 Hz, 1H), 3.60 (ddd, J = 14.9, 4.1, 2.6 Hz, 1H), 3.13 (bs, 1H), 2.30 (s, 3H).
^{13}C NMR (150 MHz, $CDCl_3$) δ 169.7, 151.1, 137.4 (d, J = 1.5 Hz), 127.0, 122.5, 68.6, 58.8 (d, J = 13.0 Hz), 21.2.
^{19}F NMR (565 MHz, $CDCl_3$) δ 59.58 (t, J = 3.7 Hz).
HRMS-ESI (m/z) $[M+Na]^+$ calculated for $C_{10}H_{11}FNaO_5S$ 285.0203, found 285.0209.

4-(2-(Fluorosulfonyl)-1-hydroxyethyl)phenyl 4-methylbenzenesulfonate (21)

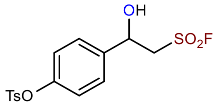

Prepared according to procedure A using 4-vinylphenyl 4-methylbenzenesulfonate (56.0 mg, 0.20 mmol).
Flash chromatography: PE/DCM/EA = 20/20/1
29.0 mg, 39% yield, white solid.
^{1}H NMR (600 MHz, $CDCl_3$) δ 7.70 (d, J = 8.2 Hz, 2H), 7.33 (dd, J = 12.8, 8.4 Hz, 4H), 7.03 (d, J = 8.5 Hz, 2H), 5.34 (dd, J = 9.6, 2.1 Hz, 1H), 3.74 (m, 1H), 3.63 – 3.55 (m, 1H), 2.45 (s, 3H).
^{13}C NMR (150 MHz, $CDCl_3$) δ 149.9, 145.8, 138.6 (d, J = 1.3 Hz), 132.3, 130.0, 128.6, 127.2, 123.3, 68.4, 58.9 (d, J = 13.1 Hz), 21.9.
^{19}F NMR (565 MHz, $CDCl_3$) δ 59.58 (t, J = 3.6 Hz).
HRMS-ESI (m/z) $[M+Na]^+$ calculated for $C_{15}H_{15}FNaO_6S_2$ 397.0186, found 397.0195.

2-([1,1'-Biphenyl]-4-yl)-2-hydroxyethane-1-sulfonyl fluoride (22)

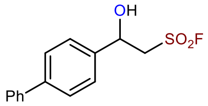

Prepared according to procedure A using 4-vinyl-1,1'-biphenyl (36.8 mg, 0.20 mmol).
Flash chromatography: PE/DCM/EA = 20/20/1
33.0 mg, 59% yield, white solid.
^{1}H NMR (600 MHz, $CDCl_3$) δ 7.64 (d, J = 7.9 Hz, 2H), 7.59 (d, J = 7.5 Hz, 2H), 7.52 – 7.44 (m, 4H), 7.39 (t, J = 7.2 Hz, 1H), 5.42 (d, J = 9.4 Hz, 1H), 3.88 – 3.80 (m, 1H), 3.67 (d, J = 14.8 Hz, 1H), 2.89 (bs, 1H).
^{13}C NMR (150 MHz, $CDCl_3$) δ 142.4 (d, J = 3.5 Hz), 140.3, 129.1, 128.0, 127.9, 127.3, 126.3, 126.2, 69.0, 59.0 (d, J = 13.5 Hz).
^{19}F NMR (565 MHz, $CDCl_3$) δ 59.34 (t, J = 3.8 Hz).
HRMS-ESI (m/z) $[2M+H]^+$ calculated for $C_{29}H_{26}F_2O_6S_2$ 561.1212, found 561.1238.

2-Hydroxy-2-(naphthalen-2-yl)ethane-1-sulfonyl fluoride (23)

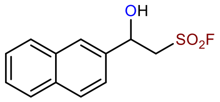

Prepared according to procedure A using 2-vinylnaphthalene (31.8 mg, 0.20 mmol).

Flash chromatography: PE/DCM/EA = 20/20/1

20.3 mg, 40% yield, white solid.

^{1}H NMR (600 MHz, $CDCl_3$) δ 7.92 – 7.83 (m, 4H), 7.56 – 7.51 (m, 2H), 7.46 (dd, *J* = 8.5, 1.4 Hz, 1H), 5.53 (dd, *J* = 9.8, 2.1 Hz, 1H), 3.87 (ddd, *J* = 14.0, 9.8, 3.9 Hz, 1H), 3.74 – 3.68 (m, 1H), 2.69 (bs, 1H).

^{13}C NMR (150 MHz, $CDCl_3$) δ 136.9 (d, *J* = 1.5 Hz), 133.6, 133.3, 129.4, 128.2, 127.0, 126.98, 126.95, 125.2, 122.9, 69.3, 58.9 (d, *J* = 13.0 Hz).

^{19}F NMR (565 MHz, $CDCl_3$) δ 59.43 (t, *J* = 4.0 Hz).

HRMS-ESI (m/z) $[M+Na]^+$ calculated for $C_{12}H_{11}FNaO_3S$ 277.0305, found 277.0309.

2-Hydroxy-2-(naphthalen-1-yl)ethane-1-sulfonyl fluoride (24)

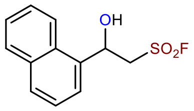

Prepared according to procedure A using 1-vinylnaphthalene (31.2 μL, 0.20 mmol).

Flash chromatography: PE/DCM/EA = 20/20/1

30.0 mg, 59% yield, yellow solid.

^{1}H NMR (600 MHz, $CDCl_3$) δ 7.95 (d, *J* = 8.5 Hz, 1H), 7.92 (d, *J* = 8.1 Hz, 1H), 7.86 (d, *J* = 8.2 Hz, 1H), 7.76 (d, *J* = 7.1 Hz, 1H), 7.60 (t, *J* = 7.5 Hz, 1H), 7.55 (t, *J* = 7.4 Hz, 1H), 7.52 (t, *J* = 7.7 Hz, 1H), 6.12 (dd, *J* = 8.9, 2.2 Hz, 1H), 3.86 – 3.77 (m, 2H), 3.08 (bs, 1H).

^{13}C NMR (150 MHz, $CDCl_3$) δ 135.0 (d, *J* = 1.1 Hz), 134.0, 129.7, 129.6, 129.3, 127.4, 126.3, 125.6, 123.6, 121.8, 66.2, 58.4 (d, *J* = 12.7 Hz).

^{19}F NMR (565 MHz, $CDCl_3$) δ 58.92 (t, *J* = 3.8 Hz).

HRMS-ESI (m/z) $[M-H]^-$ calculated for $C_{12}H_{10}FO_3S$ 253.0340, found 253.0342.

2-(Benzo[*b*]thiophen-5-yl)-2-hydroxyethane-1-sulfonyl fluoride (25)

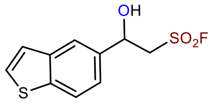

Prepared according to procedure A using 5-vinylbenzo[*b*]thiophene (32.7 mg, 0.20 mmol).

Flash chromatography: PE/DCM/EA = 10/10/1

29.0 mg, 56% yield, colorless oil.

^{1}H NMR (600 MHz, $CDCl_3$) δ 7.91 (d, *J* = 8.3 Hz, 1H), 7.87 (s, 1H), 7.52 (d, *J* = 5.4 Hz, 1H), 7.37 – 7.33 (m, 2H), 5.49 (dd, *J* = 9.8, 2.3 Hz, 1H), 3.89 – 3.82 (m, 1H), 3.68 (ddd, *J* = 14.9, 3.9, 2.6 Hz, 1H), 2.41 (bs, 1H).

^{13}C NMR (150 MHz, $CDCl_3$) δ 140.5, 140.06, 136.0 (d, *J* = 1.5 Hz), 128.1, 123.9, 123.5, 121.6, 120.9, 69.3, 59.2 (d, *J* = 12.8 Hz).

^{19}F NMR (565 MHz, $CDCl_3$) δ 59.38 (t, *J* = 3.9 Hz).

HRMS-ESI (m/z) $[M+K]^+$ calculated for $C_{10}H_9FKO_3S_2$ 298.9609, found 298.9616.

2-Hydroxy-2-phenylpropane-1-sulfonyl fluoride (26)

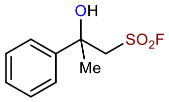

Prepared according to procedure A using prop-1-en-2-ylbenzene (26.3µL, 0.20 mmol).
Flash chromatography: PE/DCM/EA = 10/10/1
27.0 mg, 62% yield, colorless oil.
^{1}H NMR (600 MHz, $CDCl_3$) δ 7.49 – 7.46 (m, 2H), 7.41 (dd, *J* = 10.5, 5.0 Hz, 2H), 7.34 (t, *J* = 7.3 Hz, 1H), 3.90 (dd, *J* = 14.9, 3.8 Hz, 1H), 3.83 (dd, *J* = 14.9, 2.7 Hz, 1H), 3.24 (bs, 1H), 1.84 (s, 3H).
^{13}C NMR (150 MHz, $CDCl_3$) δ 143.7 (d, *J* = 2.1 Hz), 128.9, 128.3, 124.5, 72.3, 62.9 (d, *J* = 10.1 Hz), 29.3 (d, *J* = 2.1 Hz).
^{19}F NMR (565 MHz, $CDCl_3$) δ 65.64.
HRMS-ESI (m/z) $[M+Na]^+$ calculated for $C_9H_{11}FNaO_3S$ 241.0305, found 241.0305.

2-Bromo-2-hydroxy-2-phenylethane-1-sulfonyl fluoride (27)

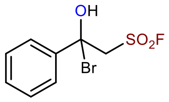

Prepared according to procedure A using (1-bromovinyl)benzene (27.3µL, 0.20 mmol).
Flash chromatography: PE/DCM/EA = 10/10/1
11.3 mg, 20% yield, colorless oil.
^{1}H NMR (400 MHz, $CDCl_3$) δ 7.96 (d, *J* = 7.7 Hz, 2H), 7.70 (t, *J* = 7.3 Hz, 1H), 7.56 (t, *J* = 7.4 Hz, 2H), 4.99 (s, 2H), 1.70 (bs, 1H).
^{13}C NMR (100 MHz, $CDCl_3$) δ 135.4, 134.6 (d, *J* = 2.7 Hz), 129.4, 129.0, 57.6 (d, *J* = 15.8 Hz), 29.8.
^{19}F NMR (377 MHz, $CDCl_3$) δ 62.94.
HRMS-ESI (m/z) $[2M+Na]^+$ calculated for $C_{16}H_{16}Br_2F_2NaO_6S_2$ 586.8615, found 586.8585.

2-Hydroxy-2-((8*R*,9*S*,13*S*,14*S*)-13-methyl-17-oxo-7,8,9,11,12,13,14,15,16,17-decahydro-6*H*-cyclopenta[a]phenanthren-3-yl)ethane-1-sulfonyl fluoride (28)

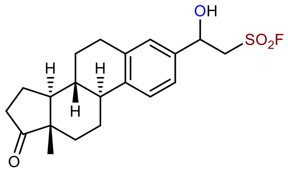

Prepared according to procedure A using (8*R*,9*S*,13*S*,14*S*)-13-methyl-3-vinyl-6,7,8,9,11,12,13,14,15,16-decahydro-17*H*-cyclopenta[*a*]phenanthren-17-one (57.2 mg, 0.20 mmol).
Flash chromatography: PE/DCM/EA = 10/10/1
27.4 mg, 36% yield, colorless oil.
^{1}H NMR (600 MHz, $CDCl_3$) δ 7.32 (d, *J* = 8.1 Hz, 1H), 7.16 (d, *J* = 8.0 Hz, 1H), 7.13 (s, 1H), 5.30 (dd, *J* = 9.8, 2.2 Hz, 1H), 3.82 – 3.76 (m, 1H), 3.61 (ddd, *J* = 14.9, 4.1, 2.6 Hz, 1H), 2.95 – 2.90 (m, 2H), 2.50 (dd, *J* = 19.0, 8.8 Hz, 1H), 2.42 (dd, *J* = 8.8, 6.6 Hz, 1H), 2.32 – 2.27 (m, 1H), 2.18 – 2.11 (m, 1H), 2.09 – 2.02 (m, 2H), 1.98 – 1.94 (m, 1H), 1.67 – 1.58 (m, 2H), 1.54 – 1.43 (m, 4H), 0.90 (s, 3H).
^{13}C NMR (150 MHz, $CDCl_3$) δ 140.9, 137.6, 137.3, 126.4, 126.3, 126.2, 123.1, 123.1, 68.9, 68.8, 58.9 (dd, *J* = 12.6, 1.9 Hz), 50.5, 48.0, 44.4, 38.1, 35.9, 31.6, 29.5, 29.5, 26.4, 25.8, 21.7, 13.9.

^{19}F NMR (565 MHz, $CDCl_3$) δ 59.47 (dd, *J* = 8.4, 4.3 Hz).
HRMS-ESI (m/z) $[M+Na]^+$ calculated for $C_{20}H_{25}FNaO_4S$ 403.1350, found 403.1357.

(3*S*,5*S*,8*R*,9*S*,10*S*,13*R*,14*S*,17*R*)-10,13-Dimethyl-17-((*R*)-6-methylheptan-2-yl)hexadecahydro-1*H*-cyclopenta[*a*]phenanthren-3-yl 4-(2-(fluorosulfonyl)-1-hydroxyethyl)benzoate (29)

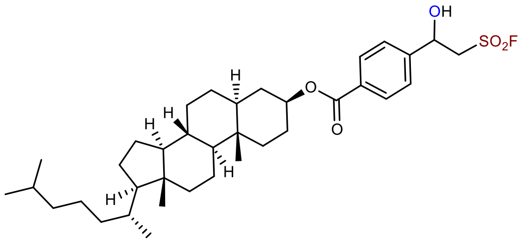

Prepared according to procedure A using (3*S*,5*S*,8*R*,9*S*,10*S*,13*R*,14*S*,17*R*)-10,13-dimethyl-17-((*R*)-6-methylheptan-2-yl)hexadecahydro-1*H*-cyclopenta[*a*]phenanthren-3-yl 4-vinylbenzoate (105.8 mg, 0.20 mmol).
Flash chromatography: PE/DCM/EA = 10/10/1
68.0 mg, 55% yield, white solid.
^{1}H NMR (600 MHz, $CDCl_3$) δ 8.05 (d, *J* = 8.0 Hz, 2H), 7.47 (d, *J* = 8.3 Hz, 2H), 5.43 (dd, *J* = 9.7, 2.2 Hz, 1H), 4.96 – 4.89 (m, 1H), 3.81 – 3.75 (m, 1H), 3.66 – 3.61 (m, 1H), 3.15 (bs, 1H), 1.98 (dt, *J* = 12.4, 3.1 Hz, 1H), 1.92 (dd, *J* = 9.0, 3.1 Hz, 1H), 1.85 – 1.76 (m, 2H), 1.73 – 1.62 (m, 3H), 1.58 – 1.48 (m, 4H), 1.38 – 1.21 (m, 11H), 1.16 – 0.97 (m, 10H), 0.91 (d, *J* = 6.5 Hz, 3H), 0.88 – 0.85 (m, 9H), 0.66 (s, 3H).
^{13}C NMR (150 MHz, $CDCl_3$) δ 165.6, 144.2, 131.8, 130.5, 125.7, 75.0, 68.8, 58.8 (d, *J* = 13.5 Hz), 56.6, 56.4, 54.4, 44.8, 42.7, 40.1, 39.7, 36.9, 36.3, 36.0, 35.65, 35.63, 34.2, 32.1, 28.8, 28.4, 28.2, 27.7, 24.4, 24.0, 23.0, 22.7, 21.37, 18.8, 12.4, 12.2.
^{19}F NMR (565 MHz, $CDCl_3$) δ 59.63 (d, *J* = 3.9 Hz).
HRMS-ESI (m/z) $[M+H]^+$ calculated for $C_{36}H_{56}FO_5S$ 619.3827, found 619.3832.

2-Hydroxy-3-phenylpropane-1-sulfonyl fluoride (30)

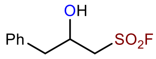

Prepared according to procedure A using allylbenzene (27.0 μL, 0.20 mmol).
Flash chromatography: PE/DCM/EA = 20/20/1
28.2 mg, 65% yield, colorless oil.
^{1}H NMR (600 MHz, $CDCl_3$) δ 7.36 (dd, *J* = 10.0, 4.7 Hz, 2H), 7.30 (t, *J* = 7.3 Hz, 1H), 7.22 (d, *J* = 7.1 Hz, 2H), 5.30 (bs, 1H), 4.55 – 4.47 (m, 1H), 3.54 – 3.46 (m, 2H), 2.96 (dd, *J* = 13.8, 7.3 Hz, 1H), 2.90 (dd, *J* = 13.8, 5.9 Hz, 1H).
^{13}C NMR (150 MHz, $CDCl_3$) δ 135.6, 129.5, 129.2, 127.6, 67.6, 56.6 (d, *J* = 13.8 Hz), 42.7 (d, *J* = 1.7 Hz).
^{19}F NMR (565 MHz, $CDCl_3$) δ 59.75 (t, *J* = 4.0 Hz).
HRMS-ESI (m/z) $[M+Na]^+$ calculated for $C_9H_{11}FNaO_3S$ 241.0305, found 241.0307.

2-Hydroxy-4-phenylbutane-1-sulfonyl fluoride (31)

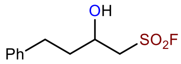

Prepared according to procedure A using but-3-en-1-ylbenzene (30.7 μL, 0.20 mmol).
Flash chromatography: PE/DCM/EA = 20/20/1
22.7 mg, 49% yield, colorless oil.

^{1}**H NMR** (600 MHz, $CDCl_3$) δ 7.32 (t, J = 7.5 Hz, 2H), 7.25 – 7.17 (m, 3H), 4.28 (ddd, J = 11.9, 8.4, 3.5 Hz, 1H), 4.15 (bs, 1H), 3.56 – 3.45 (m, 2H), 2.86 (ddd, J = 14.4, 9.2, 5.6 Hz, 1H), 2.79 – 2.72 (m, 1H), 2.00 – 1.93 (m, 1H), 1.92 – 1.84 (m, 1H).
^{13}**C NMR** (150 MHz, $CDCl_3$) δ 140.5, 128.8, 128.5, 126.6, 65.9, 57.7 (d, J = 12.9 Hz), 37.8 (d, J = 1.1 Hz), 31.4.
^{19}**F NMR** (565 MHz, $CDCl_3$) δ 59.27 (dd, J = 5.1, 3.3 Hz).
HRMS-ESI (m/z) $[M+Na]^+$ calculated for $C_{10}H_{13}FNaO_3S$ 255.0462, found 255.0462.

2-Hydroxyhexane-1-sulfonyl fluoride (32)

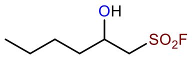

Prepared according to procedure A using hex-1-ene (25.9 μL, 0.20 mmol).
Flash chromatography: PE/DCM/EA = 20/20/1
20.0 mg, 54% yield, colorless oil.
^{1}**H NMR** (600 MHz, $CDCl_3$) δ 4.27 (dq, J = 7.9, 5.5 Hz, 1H), 3.51 (dd, J = 5.3, 4.6 Hz, 2H), 3.34 (bs, 1H), 1.68 – 1.62 (m, 1H), 1.61 – 1.54 (m, 1H), 1.50 – 1.43 (m, 1H), 1.40 – 1.32 (m, 3H), 0.92 (t, J = 7.1 Hz, 3H).
^{13}**C NMR** (150 MHz, $CDCl_3$) δ 66.7, 57.7 (d, J = 12.8 Hz), 36.1 (d, J = 1.4 Hz), 27.3, 22.4, 14.0.
^{19}**F NMR** (565 MHz, $CDCl_3$) δ 59.10 (t, J = 4.3 Hz).
HRMS-ESI (m/z) $[M+Na]^+$ calculated for $C_6H_{13}FNaO_3S$ 207.0462, found 207.0465.

2-Hydroxydodecane-1-sulfonyl fluoride (33)

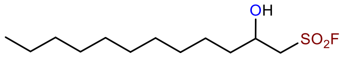

Prepared according to procedure A using dodec-1-ene (46.7 μL, 0.20 mmol).
Flash chromatography: PE/DCM/EA = 10/10/1
22.0 mg, 41% yield, colorless oil.
^{1}**H NMR** (600 MHz, $CDCl_3$) δ 4.64 (bs, 1H), 4.27 (dq, J = 7.9, 5.4 Hz, 1H), 3.50 (dd, J = 5.3, 4.4 Hz, 2H), 1.68 – 1.61 (m, 1H), 1.60 – 1.54 (m, 1H), 1.31 – 1.24 (m, 16H), 0.88 (t, J = 7.0 Hz, 3H).
^{13}**C NMR** (150 MHz, $CDCl_3$) δ 66.7, 57.7 (d, J = 12.8 Hz), 36.4 (d, J = 1.4 Hz), 32.0, 29.7, 29.6, 29.6, 29.4, 29.3, 25.2, 22.8, 14.2.
^{19}**F NMR** (565 MHz, $CDCl_3$) δ 59.10 (t, J = 4.2 Hz).
HRMS-ESI (m/z) $[M+Na]^+$ calculated for $C_{12}H_{25}FNaO_3S$ 291.1401, found 291.1409.

2-Cyclohexyl-2-hydroxyethane-1-sulfonyl fluoride (34)

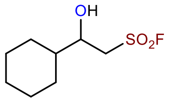

Prepared according to procedure A using vinylcyclohexane (27.9 μL, 0.20 mmol).
Flash chromatography: PE/DCM/EA = 10/10/1
17.0 mg, 40% yield, colorless oil.
^{1}**H NMR** (600 MHz, $CDCl_3$) δ 4.07 (ddd, J = 8.4, 5.5, 2.8 Hz, 1H), 3.56 – 3.48 (m, 2H), 2.49 – 2.35 (m, 2H), 1.83 – 1.77 (m, 3H), 1.71 – 1.67 (m, 2H), 1.50 (dddd, J = 11.8, 8.7, 5.8, 3.1 Hz, 1H), 1.30 – 1.21 (m, 3H), 1.19 – 1.05 (m, 3H).
^{13}**C NMR** (150 MHz, $CDCl_3$) δ 70.6, 55.9 (d, J = 12.8 Hz), 43.2 (d, J = 1.2 Hz), 28.8, 27.6, 26.2, 26.0, 25.8.
^{19}**F NMR** (565 MHz, $CDCl_3$) δ 59.06 (t, J = 4.1 Hz).

HRMS-ESI (m/z) $[M+Na]^+$ calculated for $C_8H_{15}FNaO_3S$ 233.0618, found 233.0619.

2-Hydroxy-3-oxopentane-1-sulfonyl fluoride (35)

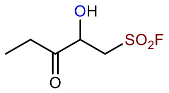

Prepared according to procedure B using pent-1-en-3-one (220 µL, 2.0 mmol).
Flash chromatography: PE/DCM/EA = 10/10/1
162.0 mg, 44% yield, colorless oil.
^{1}H NMR (600 MHz, $CDCl_3$) δ 4.66 (dd, J = 8.0, 2.4 Hz, 1H), 3.98 (ddd, J = 15.1, 3.6, 3.1 Hz, 1H), 3.63 (ddd, J = 15.1, 8.1, 4.3 Hz, 1H), 3.26 (bs, 1H), 2.73 – 2.62 (m, 2H), 1.15 (t, J = 7.2 Hz, 3H).
^{13}C NMR (150 MHz, $CDCl_3$) δ 207.6, 71.6, 54.4 (d, J = 15.6 Hz), 31.7, 7.4.
^{19}F NMR (565 MHz, $CDCl_3$) δ 61.09 (t, J = 3.9 Hz).
HRMS-ESI (m/z) $[M+Na]^+$ calculated for $C_5H_9FNaO_4S$ 207.0098, found 207.0103.

3-(Fluorosulfonyl)-2-hydroxypropyl acetate (36)

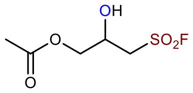

Prepared according to procedure A using allyl acetate (22.0 µL, 0.20 mmol).
Flash chromatography: PE/DCM/EA = 10/10/1
17.6 mg, 44% yield, yellow oil.
^{1}H NMR (600 MHz, $CDCl_3$) δ 4.51 (dq, J = 10.1, 5.0 Hz, 1H), 4.20 (d, J = 5.0 Hz, 2H), 3.64 – 3.59 (m, 2H), 2.86 (bs, 1H), 2.12 (s, 3H).
^{13}C NMR (150 MHz, $CDCl_3$) δ 171.0, 66.0 (d, J = 1.9 Hz), 65.1, 54.6 (d, J = 15.1 Hz), 20.8.
^{19}F NMR (565 MHz, $CDCl_3$) δ 60.04 (t, J = 4.0 Hz).
HRMS-ESI (m/z) $[M+Na]^+$ calculated for $C_5H_9FNaO_5S$ 223.0047, found 223.0048.

11-(Fluorosulfonyl)-10-hydroxyundecanoic acid (37)

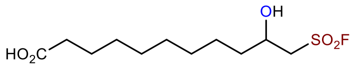

Prepared according to procedure A using undec-10-enoic acid (41.2 µL, 0.20 mmol).
Flash chromatography: PE/DCM/EA = 2/2/1
42.0 mg, 74% yield, colorless oil.
^{1}H NMR (600 MHz, $CDCl_3$) δ 4.30 – 4.23 (m, 1H), 3.52 – 3.47 (m, 2H), 2.35 (t, J = 7.5 Hz, 2H), 1.93 – 1.70 (m, 1H), 1.67 – 1.53 (m, 4H), 1.51 – 1.43 (m, 1H), 1.39 – 1.26 (m, 8H).
^{13}C NMR (150 MHz, $CDCl_3$) δ 179.8, 66.6, 57.8 (d, J = 12.7 Hz), 36.3 (d, J = 1.2 Hz), 34.1, 29.2, 29.2, 29.1, 29.0, 25.1, 24.7.
^{19}F NMR (565 MHz, $CDCl_3$) δ 59.16.
HRMS-ESI (m/z) $[M+Na]^+$ calculated for $C_{11}H_{21}FNaO_5S$ 307.0986, found 307.0985.

Diethyl (4-(fluorosulfonyl)-3-hydroxybutyl)phosphonate (38)

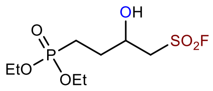

Prepared according to procedure A using diethyl but-3-en-1-ylphosphonate (40.3 μL, 0.20 mmol).
Flash chromatography: PE/DCM/EA = 10/10/1
19.8 mg, 34% yield, yellow oil.
^{1}H NMR (600 MHz, $CDCl_3$) δ 4.99 (bs, 1H), 4.32 (s, 1H), 4.14 – 4.05 (m, 2H), 3.65 – 3.59 (m, 1H), 3.51 (dt, *J* = 14.6, 3.7 Hz, 1H), 2.03 – 1.81 (m, 2H), 1.32 (t, *J* = 7.0 Hz, 3H).
^{13}C NMR (150 MHz, $CDCl_3$) δ 66.1 (d, *J* = 13.9 Hz), 62.4 (d, *J* = 6.5 Hz), 57.3 (d, *J* = 13.1 Hz), 29.4 (dd, *J* = 3.9, 2.0 Hz), 21.5 (d, *J* = 142.3 Hz), 16.5 (d, *J* = 5.6 Hz).
^{19}F NMR (565 MHz, $CDCl_3$) δ 59.94.
HRMS-ESI (m/z) $[M+Na]^+$ calculated for $C_8H_{18}FNaO_6PS$ 315.0438, found 315. 0440.

Diethyl (6-(fluorosulfonyl)-5-hydroxyhexyl)phosphonate (39)

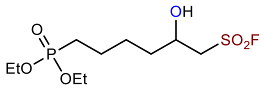

Prepared according to procedure A using diethyl hex-5-en-1-ylphosphonate (44.9 mg, 0.20 mmol).
Flash chromatography: PE/DCM/EA = 10/10/1
21.1 mg, 33% yield, yellow oil.
^{1}H NMR (600 MHz, $CDCl_3$) δ 4.27 (dd, *J* = 9.5, 6.6 Hz, 1H), 4.13 – 4.04 (m, 4H), 3.58 – 3.53 (m, 1H), 3.48 (dt, *J* = 14.7, 3.3 Hz, 1H), 3.20 (bs, 1H), 1.75 (dd, *J* = 17.3, 7.3 Hz, 2H), 1.67 – 1.55 (m, 5H), 1.32 (t, *J* = 7.0 Hz, 6H).
^{13}C NMR (150 MHz, $CDCl_3$) δ 66.0, 61.9 (d, *J* = 6.2 Hz), 57.7 (d, *J* = 12.6 Hz), 25.8 (d, *J* = 11.7 Hz), 25.3 (d, *J* = 143.6 Hz), 22.0 (d, *J* = 5.0 Hz), 16.6 (d, *J* = 6.0 Hz)
^{13}C NMR (150 MHz, $CDCl_3$) δ 66.0, 61.9 (d, *J* = 6.2 Hz), 57.7 (d, *J* = 12.6 Hz), 35.7, 25.8 (d, *J* = 3.0 Hz), 25.7, 22.0 (d, *J* = 5.0 Hz), 16.6 (d, *J* = 6.0 Hz).
^{19}F NMR (565 MHz, $CDCl_3$) δ 59.51 (t, *J* = 3.5 Hz).
HRMS-ESI (m/z) $[M+Na]^+$ calculated for $C_{10}H_{22}FNaO_6PS$ 343.0751, found 343.0757.

6-Bromo-2-hydroxyhexane-1-sulfonyl fluoride (40)

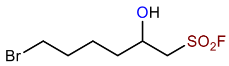

Prepared according to procedure A using 6-bromohex-1-ene (27.6 μL, 0.20 mmol).
Flash chromatography: PE/DCM/EA = 10/10/1
22.0 mg, 42% yield, yellow oil.
^{1}H NMR (600 MHz, $CDCl_3$) δ 4.88 (bs, 1H), 4.33 – 4.26 (m, 1H), 3.56 – 3.48 (m, 2H), 3.42 (t, *J* = 6.6 Hz, 2H), 1.91 (tt, *J* = 14.2, 7.1 Hz, 2H), 1.70 – 1.56 (m, 4H).
^{13}C NMR (150 MHz, $CDCl_3$) δ 66.5, 57.7 (d, *J* = 13.1 Hz), 35.4 (d, *J* = 1.4 Hz), 33.2, 32.3, 23.9.
^{19}F NMR (565 MHz, $CDCl_3$) δ 59.34 (t, *J* = 4.0 Hz).
HRMS-ESI (m/z) $[M+NH_4]^+$ calculated for $C_6H_{16}BrFNO_3S$ 280.0013, found 279.9999.

(±)-2-Hydroxycyclopentane-1-sulfonyl fluoride (41)

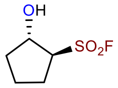

Prepared according to procedure A using cyclopentene (18.0 μL, 0.20 mmol).
Flash chromatography: PE/DCM/EA = 10/10/1
20.8 mg, 62% yield, colorless oil.
The diastereomeric ratio > 20:1 (Isomer A : Isomer B) as determined by **^{1}H NMR** analysis.
^{1}H NMR (600 MHz, $CDCl_3$) δ 4.71 (dd, *J* = 12.3, 6.2 Hz, 1H), 3.71 – 3.62 (m, 1H), 2.35 (dt, *J* = 14.6, 8.0 Hz, 1H), 2.22 (bs, 1H), 2.18 – 2.09 (m, 2H), 1.94 – 1.74 (m, 3H).
^{13}C NMR (150 MHz, $CDCl_3$) δ 74.5, 68.5 (d, *J* = 11.5 Hz), 34.7 (d, *J* = 1.1 Hz), 26.8, 22.4.
^{19}F NMR (565 MHz, $CDCl_3$) δ 47.78.
HRMS-ESI (m/z) $[M+Na]^+$ calculated for $C_5H_9FNaO_3S$ 191.0149, found 191.0153.

(±)-2-Hydroxycyclohexane-1-sulfonyl fluoride (42)

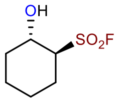

Prepared according to procedure A using cyclohexene (20.5 μL, 0.20 mmol).
Flash chromatography: PE/DCM/EA = 10/10/1
33.8 mg, 93% yield, colorless oil.
The diastereomeric ratio was 4/1 (Isomer A : Isomer B) as determined by **^{1}H NMR** analysis.
Isomer A
^{1}H NMR (600 MHz, $CDCl_3$) δ 3.99 (td, *J* = 10.1, 4.7 Hz, 1H), 3.33 (ddt, *J* = 12.8, 9.6, 3.4 Hz, 1H), 2.36 (dq, *J* = 12.7, 3.4 Hz, 1H), 2.21 – 2.16 (m, 1H), 1.91 – 1.81 (m, 2H), 1.68 (qd, *J* = 12.9, 3.9 Hz, 2H), 1.43 – 1.29 (m, 4H).
^{13}C NMR (150 MHz, $CDCl_3$) δ 68.9, 68.0 (d, *J* = 8.7 Hz), 34.2 (d, *J* = 1.4 Hz), 26.2, 24.3, 23.6.
^{19}F NMR (565 MHz, $CDCl_3$) δ 49.43.
Isomer B
^{1}H NMR (400 MHz, $CDCl_3$) δ 4.63 – 4.49 (m, 1H), 3.45 – 3.40 (m, 1H), 2.59 (s,1H), 2.13 – 2.07 (m, 1H), 2.05 – 1.99 (m, 1H), 1.75 – 1.63 (m, 4H), 1.56 – 1.49 (m, 2H).
^{13}C NMR (150 MHz, $CDCl_3$) δ65.46 (d, *J* = 9.3 Hz), 64.34, 32.53 (d, *J* = 1.3 Hz), 24.70, 21.18, 18.40.
^{19}F NMR (565 MHz, $CDCl_3$) δ 49.99.
HRMS-ESI (m/z) $[M+Na]^+$ calculated for $C_6H_{11}FNaO_3S$ 205.0305, found 205.0305.

(±)-1-Hydroxy-2,3-dihydro-1*H*-indene-2-sulfonyl fluoride (43)

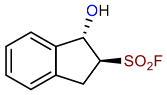

Prepared according to procedure A using 1*H*-indene (24.6 μL, 0.20 mmol).
Flash chromatography: PE/DCM/EA = 20/20/1
22.7 mg, 53% yield, colorless oil.
The diastereomeric ratio > 20:1 (Isomer A : Isomer B) as determined by **^{1}H NMR** analysis.
^{1}H NMR (600 MHz, $CDCl_3$) δ 7.45 – 7.41 (m, 1H), 7.38 – 7.34 (m, 2H), 7.29 – 7.26 (m, 1H), 5.77 (d, *J* = 6.5 Hz, 1H), 4.09 – 4.03 (m, 1H), 3.53 (dd, *J* = 16.3, 9.0 Hz, 1H), 3.43 (dd, *J* = 16.2, 8.7 Hz, 1H), 2.87 (bs, 1H).

^{13}C NMR (150 MHz, $CDCl_3$) δ 140.3 (d, J = 1.4 Hz), 137.0, 129.8, 128.4, 124.9, 124.7, 76.8, 68.9 (d, J = 12.4 Hz), 32.1.
^{19}F NMR (565 MHz, $CDCl_3$) δ 48.97 (d, J = 1.6 Hz).
HRMS-ESI (m/z) $[M+Na]^+$ calculated for $C_9H_9FNaO_3S$ 239.0149, found 239.0148.

(±)-1-Hydroxy-1,2,3,4-tetrahydronaphthalene-2-sulfonyl fluoride (44)

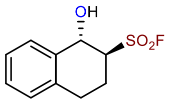

Prepared according to procedure A using 1,2-dihydronaphthalene (26.6 μL, 0.20 mmol).
Flash chromatography: PE/DCM/EA = 20/20/1
39.4 mg, 86% yield, white solid.
The diastereomeric ratio > 20:1 (Isomer A : Isomer B) as determined by **^{1}H NMR** analysis.
^{1}H NMR (600 MHz, $CDCl_3$) δ 7.60 (d, J = 7.6 Hz, 1H), 7.31 (t, J = 7.3 Hz, 1H), 7.27 (d, J = 8.0 Hz, 1H), 7.13 (d, J = 7.4 Hz, 1H), 5.31 (dd, J = 7.6, 3.2 Hz, 1H), 3.75 (ddd, J = 15.4, 8.2, 3.9 Hz, 1H), 3.16 (d, J = 4.1 Hz, 1H), 3.00 – 2.95 (m, 2H), 2.59 – 2.51 (m, 1H), 2.26 – 2.17 (m, 1H).
^{13}C NMR (150 MHz, $CDCl_3$) δ 135.0 (d, J = 7.2 Hz), 134.7, 128.5, 128.4, 127.8, 127.4, 67.6, 66.2 (d, J = 7.7 Hz), 27.6, 23.0.
^{19}F NMR (565 MHz, $CDCl_3$) δ 47.94 (d, J = 101.0 Hz).
HRMS-ESI (m/z) $[M+Na]^+$ calculated for $C_{10}H_{11}FNaO_3S$ 253.0305, found 253.0304.

(±)-1-Hydroxy-1-methyl-2,3-dihydro-1H-indene-2-sulfonyl fluoride (45)

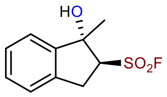

Prepared according to procedure A using 3-methyl-1*H*-indene (26.8 mg, 0.20 mmol).
Flash chromatography: PE/DCM/EA = 20/20/1
23.5 mg, 51% yield, colorless oil.
The diastereomeric ratio > 20:1 (Isomer A : Isomer B) as determined by **^{1}H NMR** analysis.
^{1}H NMR (600 MHz, $CDCl_3$) δ 7.40 – 7.31 (m, 3H), 7.24 (dd, J = 5.3, 2.5 Hz, 1H), 4.19 (dd, J = 9.7, 8.5 Hz, 1H), 3.49 – 3.37 (m, 2H), 2.85 (bs, 1H), 1.73 (d, J = 1.2 Hz, 3H).
^{13}C NMR (150 MHz, $CDCl_3$) δ 145.2 (d, J = 3.7 Hz), 135.2, 129.5, 128.6, 124.9, 122.8, 82.2, 72.3 (d, J = 10.9 Hz), 32.0, 25.8.
^{19}F NMR (565 MHz, $CDCl_3$) δ 58.37.
HRMS-ESI (m/z) $[M+Na]^+$ calculated for $C_{10}H_{11}FNaO_3S$ 253.0305,found 253.0311.

(±)-2-Hydroxy-2-methyl-2,3-dihydro-1H-indene-1-sulfonyl fluoride (46)

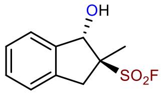

Prepared according to procedure A using 2-methyl-1*H*-indene (26.6 mg ,0.20 mmol).
Flash chromatography: PE/DCM/EA = 20/20/1
30.4 mg, 66% yield, white solid.
The diastereomeric ratio was 11/1 (Isomer A : Isomer B) as determined by **^{1}H NMR** analysis.

Isomer A

^{1}H NMR (600 MHz, $CDCl_3$) δ 7.44 – 7.40 (m, 1H), 7.37 – 7.32 (m, 2H), 7.25 – 7.22 (m, 1H), 5.85 (s, 1H), 3.71 (d, J = 15.9 Hz, 1H), 3.11 (d, J = 15.9 Hz, 1H), 2.46 (bs, 1H), 1.62 (s, 3H).

^{13}C NMR (150 MHz, $CDCl_3$) δ 139.7 (d, J = 1.5 Hz), 135.8, 129.5, 128.3, 125.1, 124.9, 77.6, 73.0 (d, J = 9.1 Hz), 40.0, 16.0.

^{19}F NMR (565 MHz, $CDCl_3$) δ 38.85.

Isomer B

^{1}H NMR (600 MHz, $CDCl_3$) δ 7.44 – 7.40 (m,1H), 7.37 – 7.32 (m, 2H), 7.26 – 7.22 (m, 1H), 5.10 (s, 1H), 3.18 (d, J = 15.8 Hz, 1H), 3.02 (d, J = 15.9 Hz, 1H), 2.46 (bs, 12H), 1.55 (s, 3H).

^{13}C NMR (150 MHz, $CDCl_3$) δ 139.7 (d, J = 1.5 Hz), 135.8, 129.2, 127.6, 125.6, 125.4, 77.6, 71.36 (d, J = 2.3 Hz), 70.4, 45.0, 23.8.

^{19}F NMR (565 MHz, CDCl3) δ38.81.

HRMS-ESI (m/z) $[M+Na]^+$ calculated for $C_{10}H_{11}FNaO_3S$ 253.0305, found 253.0310.

(±)-1-Hydroxy-1-methyl-1,2,3,4-tetrahydronaphthalene-2-sulfonyl fluoride (47)

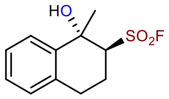

Prepared according to procedure A using 4-methyl-1,2-dihydronaphthalene (29.7 mg,0.20 mmol).

Flash chromatography: PE/DCM/EA = 20/20/1

28.8 mg, 59% yield, colorless oil.

The diastereomeric ratio > 20:1 (Isomer A : Isomer B) as determined by **^{1}H NMR** analysis.

^{1}H NMR (600 MHz, $CDCl_3$) δ 7.66 (d, J = 7.9 Hz, 1H), 7.30 (t, J = 7.6 Hz, 1H), 7.23 (t, J = 7.4 Hz, 1H), 7.08 (d, J = 7.5 Hz, 1H), 3.96 (ddd, J = 12.8, 4.6, 2.8 Hz, 1H), 3.04 (dd, J = 8.9, 3.8 Hz, 2H), 2.59 (ddd, J = 13.5, 7.3, 3.6 Hz, 1H), 2.26 (tt, J = 13.3, 9.5 Hz, 1H), 1.79 (d, J = 0.9 Hz, 3H).

^{13}C NMR (150 MHz, $CDCl_3$) δ 140.8 (d, J = 3.6 Hz), 132.8, 128.4, 128.2, 127.5, 126.5, 72.0, 70.4 (d, J = 9.7 Hz), 28.6, 28.2, 23.2.

^{19}F NMR (565 MHz, $CDCl_3$) δ 54.86 (d, J = 2.7 Hz).

HRMS-ESI (m/z) $[M+Na]^+$ calculated for $C_{11}H_{13}FNaO_3S$ 267.0462, found 267.0467.

1-Hydroxy-1-phenylpropane-2-sulfonyl fluoride (48)

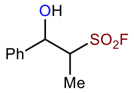

Prepared according to procedure A using (E)-prop-1-en-1-ylbenzene (27.3 μL,0.20 mmol).

Flash chromatography: PE/DCM/EA = 10/10/1

20.0 mg, 46% yield, yellow oil.

The diastereomeric ratio was 19/1 (Isomer A : Isomer B) as determined by **^{1}H NMR** analysis.

Isomer A

^{1}H NMR (600 MHz, $CDCl_3$) δ 7.43 – 7.37 (m, 4H), 7.37 – 7.33 (m, 1H), 5.60 (m, 1H), 3.66 (q, J = 7.1 Hz, 1H), 2.42 (bs, 1H), 1.44 (d, J = 7.1 Hz, 3H).

^{13}C NMR (150 MHz, $CDCl_3$) δ 138.9 (d, J = 1.5 Hz), 129.0, 128.7, 125.8, 70.4, 64.6 (d, J = 9.0 Hz), 7.3.

^{19}F NMR (565 MHz, $CDCl_3$) δ 48.49.

Isomer B

^{1}H NMR (600 MHz, $CDCl_3$) δ 7.43 – 7.37 (m, 4H), 7.37 – 7.33 (m, 1H), 5.01 (d, *J* = 8.8 Hz, 1H), 3.47 (q, *J* = 7.0 Hz, 1H), 2.42 (bs, 1H), 1.44 (d, *J* = 7.1 Hz, 3H).
^{13}C NMR (150 MHz, $CDCl_3$) δ 138.9 (d, *J* = 1.5 Hz), 129.2, 128.7, 127.1, 70.4, 64.6 (d, *J* = 9.0 Hz), 13.0.
^{19}F NMR (565 MHz, $CDCl_3$) δ 50.83.
HRMS-ESI (m/z) $[M+Na]^+$ calculated for $C_9H_{11}FNaO_3S$ 241.0305, found 241.0315.

2-(Fluorosulfonyl)-3-hydroxy-3-phenylpropyl acetate (49)

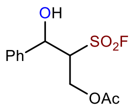

Prepared according to procedure B using 3-phenylallyl acetate (343.7 μL, 2.00 mmol).
Flash chromatography: PE/DCM/EA = 10/10/1
279.9 mg, 51% yield, yellow oil.
^{1}H NMR (600 MHz, $CDCl_3$) δ 7.45 – 7.40 (m, 4H), 7.39 – 7.34 (m, 1H), 5.62 (s, 1H), 4.71 (dd, *J* = 12.6, 7.6 Hz, 1H), 4.49 (ddd, *J* = 12.6, 4.0, 2.7 Hz, 1H), 4.05 – 3.98 (m, 1H), 2.81 (d, *J* = 2.8 Hz, 1H), 2.09 (bs, 1H), 1.95 (s, 3H).
^{13}C NMR (150 MHz, $CDCl_3$) δ 170.5, 138.3 (d, *J* = 0.8 Hz), 129.2, 129.0, 125.7, 70.3, 67.9 (d, *J* = 8.7 Hz), 57.8, 20.6.
^{19}F NMR (565 MHz, $CDCl_3$) δ 58.08 (d, *J* = 1.6 Hz).
HRMS-ESI (m/z) $[M-H]^-$ calculated for $C_{11}H_{12}FO_5S$ 275.0395, found 275.0394.

(*E*)-2-Hydroxy-3-methyl-4-phenylbut-3-ene-1-sulfonyl fluoride (50)

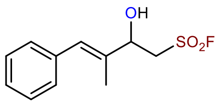

Prepared according to procedure A using (*E*)-(2-methylbuta-1,3-dien-1-yl)benzene (29.4 mg, 0.20 mmol).
Flash chromatography: PE/DCM/EA = 5/5/1
11.2 mg, 23% yield, yellow oil.
^{1}H NMR (600 MHz, $CDCl_3$) δ 7.38 – 7.31 (m, 5H), 5.95 (t, *J* = 7.9 Hz, 1H), 5.17 (s, 1H), 4.22 – 4.12 (m, 2H), 1.95 (bs, 1H), 1.63 (s, 3H).
^{13}C NMR (150 MHz, $CDCl_3$) δ 149.4, 140.9, 128.9, 128.4, 126.8, 108.6, 78.1, 50.2 (d, *J* = 17.7 Hz), 13.7.
^{19}F NMR (565 MHz, $CDCl_3$) δ 52.75 (t, *J* = 3.8 Hz).
HRMS-ESI (m/z) $[M+Na]^+$ calculated for $C_{11}H_{13}FNaO_3S$ 267.0462, found 267.0464.

(±)-6-Hydroxy-3-isopropyl-6-methylcyclohex-3-ene-1-sulfonyl fluoride (51)

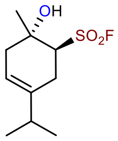

Prepared according to procedure A using γ-Terpinene (33.7 μL, 0.20 mmol).
Flash chromatography: PE/DCM/EA = 20/20/1
35.8 mg, 76% yield, colorless oil.
The diastereomeric ratio > 20:1 (Isomer A : Isomer B) as determined by **^{1}H NMR** analysis.
^{1}H NMR (600 MHz, $CDCl_3$) δ 5.38 (dd, *J* = 2.6, 1.4 Hz, 1H), 3.79 (ddd, *J* = 11.0, 5.7, 1.9 Hz, 1H), 2.74 – 2.65 (m, 2H), 2.60 – 2.52 (m, 1H), 2.37 (d, *J* = 17.6 Hz, 1H), 2.32 – 2.23 (m, 2H), 1.45 (s, 3H), 1.03 (d, *J* = 6.9 Hz, 6H).

^{13}C NMR (150 MHz, $CDCl_3$) δ 139.1, 116.9, 70.0, 68.3 (d, J = 7.6 Hz), 41.2 (d, J = 3.1 Hz), 34.3, 27.6, 22.9, 21.5, 21.1.

^{19}F NMR (565 MHz, $CDCl_3$) δ 57.85.

HRMS-ESI (m/z) $[M+Na]^+$ calculated for $C_{10}H_{17}FNaO_3S$ 259.0775, found 259.0778.

2-Hydroxyoct-7-ene-1-sulfonyl fluoride (52)

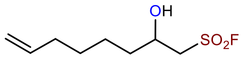

Prepared according to procedure A using octa-1,7-diene (30.5 μL, 0.20 mmol).

Flash chromatography: PE/DCM/EA = 20/20/1

16.0 mg, 38% yield, yellow oil.

^{1}H NMR (600 MHz, $CDCl_3$) δ 5.79 (ddt, J = 16.9, 10.2, 6.7 Hz, 1H), 5.05 – 4.94 (m, 2H), 4.28 (ddd, J = 11.2, 8.0, 5.1 Hz, 1H), 3.95 (bs, 1H), 3.53 – 3.47 (m, 2H), 2.08 (q, J = 6.8 Hz, 2H), 1.69 – 1.62 (m, 1H), 1.61 – 1.55 (m, 1H), 1.53 – 1.47 (m, 1H), 1.47 – 1.38 (m, 3H).

^{13}C NMR (150 MHz, $CDCl_3$) δ 138.5, 115.0, 66.6, 57.7 (d, J = 12.8 Hz), 36.2 (d, J = 1.5 Hz), 33.6, 28.5, 24.6.

^{19}F NMR (565 MHz, $CDCl_3$) δ 59.09 (t, J = 4.2 Hz).

HRMS-ESI (m/z) $[M+Na]^+$ calculated for $C_8H_{15}FNaO_3S$ 233.0618, found 233.0618.

(±)-6-Hydroxycyclohex-3-ene-1-sulfonyl fluoride (53)

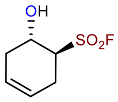

Prepared according to procedure A using cyclohexa-1,4-diene (19.5 μL, 0.20 mmol).

Flash chromatography: PE/DCM/EA = 10/10/1

26.0 mg, 72% yield, yellow oil.

The diastereomeric ratio was 5/1 (Isomer A : Isomer B) as determined by **^{1}H NMR** analysis.

Isomer A

^{1}H NMR (600 MHz, $CDCl_3$) δ 5.69 – 5.62 (m, 2H), 4.30 (td, J = 9.6, 5.9 Hz, 1H), 3.68 – 3.64 (m, 1H), 2.78 – 2.71 (m, 2H), 2.67 – 2.60 (m, 2H), 2.26 – 2.19 (m, 1H).

^{13}C NMR (150 MHz, $CDCl_3$) δ 125.0, 122.4, 66.4, 64.4 (d, J = 9.3 Hz), 33.8 (d, J = 1.3 Hz), 26.5.

^{19}F NMR (565 MHz, $CDCl_3$) δ 52.87.

Isomer B

^{1}H NMR (600 MHz, $CDCl_3$) δ 5.79 – 5.70 (m, 2H), 4.70 – 4.67 (m, 1H), 3.64 – 3.61 (m, 1H), 2.85 – 2.80 (m, 2H), 2.58 (dd, J = 4.1, 2.2 Hz, 2H), 2.27 – 2.19 (m, 1H).

^{13}C NMR (150 MHz, $CDCl_3$) δ 123.5, 122.8, 63.0, 61.9 (d, J = 10.4 Hz), 33.3 (d, J = 1.4 Hz), 21.3.

^{19}F NMR (565 MHz, $CDCl_3$) δ50.20.

HRMS-ESI (m/z) $[M+K]^+$ calculated for $C_6H_9FKO_3S$ 218.9888, found 218.9880.

1.8 Other substrate attempts

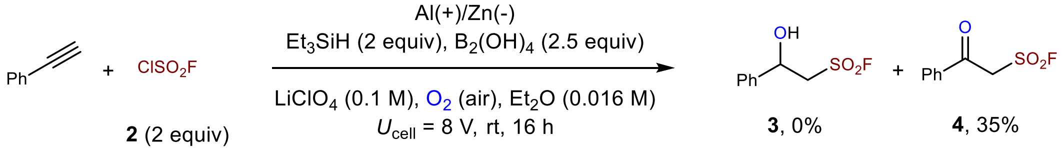

A 20-mL vial with one aluminum (anode) plate electrode (10 mm x 15 mm x 1 mm), one zinc (cathode) plate electrode (10 mm x 15 mm x 1 mm) and a stir bar was charged with $LiClO_4$ (130 mg, 0.1 M), Et_2O (12 mL, 0.016M), $B_2(OH)_4$ (0.5 mmol, 2.5 equiv), Et_3SiH (0.4 mmol, 2 equiv) and **1** alkenes (0.2 mmol). Then, $ClSO_2F$ was added (0.4 mmol, 2 eq, 1 M in anhydrous $PhCF_3$). The mixture was electrolyzed at a constant cell voltage of 8 V for 16 h under an atmosphere of air (1 atm, balloon). Subsequently, the reaction was quenched with water and electrodes were rinsed with EtOAc. The resulting mixture was extracted with EtOAc and the combined organic layers were dried over Na_2SO_4 and concentrated in vacuo. The residue was purified by column chromatography to afford the product **4** in 35% yield.

2-Oxo-2-phenylethane-1-sulfonyl fluoride (4)

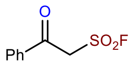

Flash chromatography: PE/DCM/EA = 20/20/1

14.1 mg, 35% yield, white solid.

^{1}H NMR (600 MHz, $CDCl_3$) δ 7.98 – 7.93 (m, 2H), 7.70 (t, *J* = 7.4 Hz, 1H), 7.56 (t, *J* = 7.9 Hz, 2H), 5.00 (d, *J* = 2.2 Hz, 2H).

All analytic data match to the reported data[8].

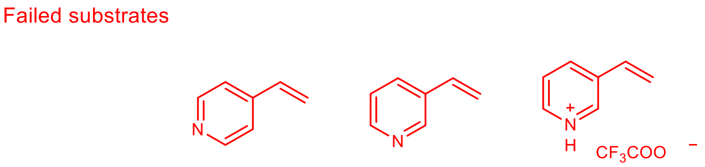

1.9 Representative derivatizations

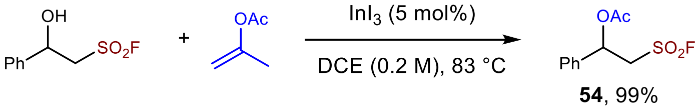

To a solution of compound **3** (41.8 mg, 0.2 mmol), InI_3 (5.0 mg, 0.01 mmol, 5 mol%) in DCE (1 mL, 0.2 M) was added isopropenyl acetatee (44.0 μL, 0.4 mmol, 2 equiv). After heating at 83 °C for 2 hour, the resulting mixture was concentrated in vacuo and the residue was purified by column chromatography to afford 2-(fluorosulfonyl)-1-phenylethyl acetate **54** as a light yellow oil (48.8 mg, 99%).

2-(Fluorosulfonyl)-1-phenylethyl acetate (54)

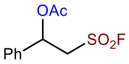

^{1}H NMR (600 MHz, $CDCl_3$) δ 7.43 – 7.36 (m, 5H), 6.33 (dd, *J* = 9.8, 3.2 Hz, 1H), 3.99 (ddd, *J* = 15.1, 9.8, 2.6 Hz, 1H), 3.71 (ddd, *J* = 15.1, 5.0, 3.3 Hz, 1H), 2.13 (s, 3H).
^{13}C NMR (150 MHz, $CDCl_3$) δ 169.2, 136.5 (d, *J* = 1.0 Hz), 129.6, 129.3, 126.4, 69.4, 56.1 (d, *J* = 15.6 Hz), 20.9.
^{19}F NMR (565 MHz, $CDCl_3$) δ 59.73 (dd, *J* = 4.4, 2.4 Hz).
HRMS-ESI (m/z) $[M+Na]^+$ calculated for $C_{10}H_{11}FNaO_4S$ 269.0254, found 269.0259.

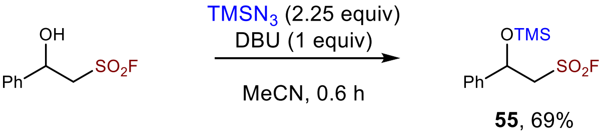

To a solution of compound **3** (41.8 mg, 0.2 mmol), $TMSN_3$ (62.4 μL, 0.45 mmol, 2.25 equiv) in MeCN (1 mL, 0.2 M) was added DBU(30.4 μL, 0.2 mmol, 1 equiv). After stirring for 0.5 hour, the resulting mixture was concentrated in vacuo and the residue was purified by column chromatography to afford 2-Phenyl-2-((trimethylsilyl)oxy)ethane-1-sulfonyl fluoride **55** as a colorless oil (38.1 mg, 69%).

2-Phenyl-2-((trimethylsilyl)oxy)ethane-1-sulfonyl fluoride (55)

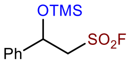

^{1}H NMR (600 MHz, $CDCl_3$) δ 7.40 – 7.32 (m, 5H), 5.30 (dd, *J* = 9.8, 2.2 Hz, 1H), 3.76 (dd, *J* = 14.6, 9.8 Hz, 1H), 3.51 (ddd, *J* = 14.7, 7.0, 2.5 Hz, 1H), 0.07 (s, 9H).
^{13}C NMR (150 MHz, $CDCl_3$) δ 140.8 (d, J = 2.1 Hz), 129.1, 128.9, 125.8, 70.2, 60.3 (d, *J* = 11.3 Hz), -0.1.
^{19}F NMR (565 MHz, $CDCl_3$) δ 60.72 (d, *J* = 7.0 Hz).
HRMS-ESI (m/z) $[M+Na]^+$ calculated for $C_{11}H_{17}FNaO_3SSi$ 299.0544, found 299.0551.

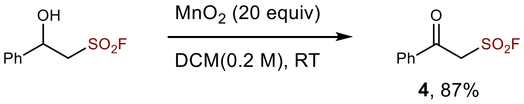

A 10 mL vial with a stir bar was charged compound **3** (41.8 mg, 0.2 mmol), DCM (1 mL, 0.2 M). MnO_2 (378.0 mg, 20 equiv) was added, and the reaction mixture was stirred at room temperature for 8 h. The crude mixture was concentrated in vacuo and the residue was purified by column chromatography to afford 2-oxo-2-phenylethane-1-sulfonyl fluoride **4** as a white solid (35.0 mg, 87%).

2-Oxo-2-phenylethane-1-sulfonyl fluoride (4)

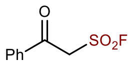

^{1}H NMR (600 MHz, $CDCl_3$) δ 7.98 – 7.93 (m, 2H), 7.70 (t, J = 7.4 Hz, 1H), 7.56 (t, J = 7.9 Hz, 2H), 5.00 (d, J = 2.2 Hz, 2H).

All analytic data match to the reported data[8].

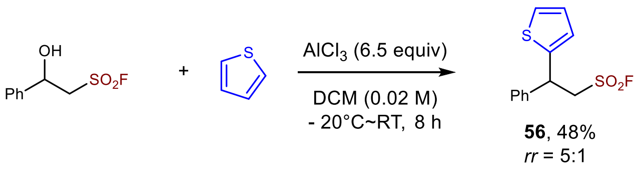

A 50 mL flask with a stir bar was charged compound **3** (41.8 mg, 0.2 mmol), thiophene (388.2 µL, 24 equiv), DCM (10 mL, 0.2 M). The mixture was stirred at – 20 °C, and $AlCl_3$ (175.2 mg, 6.5 equiv) was added, Then, the reaction mixture was warm to room temperature. The crude mixture was concentrated in vacuo and the residue was purified by column chromatography to afford 2-phenyl-2-(thiophen-2-yl)ethane-1-sulfonyl fluoride **56** as a white solid (25.9 mg, 48%).

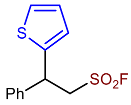

56

Major: 2-phenyl-2-(thiophen-2-yl)ethane-1-sulfonyl fluoride (56)

^{1}H NMR (600 MHz, $CDCl_3$) δ 7.36 (dt, J = 29.8, 7.6 Hz, 5H), 7.25 – 7.22 (m, 1H), 6.96 (d, J = 3.2 Hz, 2H), 4.93 (t, J = 7.3 Hz, 1H), 4.18 – 4.07 (m, 2H).

^{13}C NMR (150 MHz, $CDCl_3$) δ 139.8, 129.3, 129.3, 128.3, 127.5, 127.2, 125.6, 125.4, 57.6 (d, J = 13.4 Hz), 42.3.

^{19}F NMR (565 MHz, $CDCl_3$) δ 59.38 (t, J = 3.2 Hz).

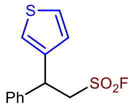

56'

Minor: 2-phenyl-2-(thiophen-3-yl)ethane-1-sulfonyl fluoride (56')

^{1}H NMR (600 MHz, $CDCl_3$) δ 7.29 (dd, J = 15.4, 6.8 Hz, 5H), 7.11 – 7.08 (m, 1H), 6.99 – 6.93 (m, 2H), 4.77 (t, J = 7.3 Hz, 1H), 4.09 – 4.02 (m, 2H).

^{13}C NMR (150 MHz, $CDCl_3$) δ 143.77, 129.26, 128.00, 127.67, 127.12, 126.83, 125.63, 125.46, 57.65 (d, J = 13.7 Hz), 42.27.

^{19}F NMR (565 MHz, $CDCl_3$) δ 59.22 (t, J = 3.2 Hz).

HRMS-ESI (m/z) $[M+K]^+$ calculated for $C_{12}H_{11}FKO_2S_2$ 308.9816, found 308.9829.

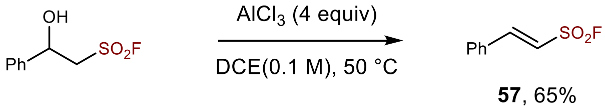

A 10 mL vial with a stir bar was charged compound **3** (41.8 mg, 0.2 mmol), DCE (2 mL, 0.2 M). $AlCl_3$ (107.8 mg, 4 equiv) was added, and the reaction mixture was stirred at 50 °C for 4 h. The crude mixture was concentrated in vacuo and the residue was purified by column chromatography to afford (E)-2-phenylethene-1-sulfonyl fluoride **57** as a

white solid (24.2 mg, 65%).

(*E*)-2-Phenylethene-1-sulfonyl fluoride (57)

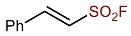

^{1}H NMR (600 MHz, $CDCl_3$) δ 7.82 (d, J = 15.5 Hz, 1H), 7.58 – 7.53 (m, 3H), 7.48 (t, J = 7.5 Hz, 2H), 6.87 (dd, J = 15.5, 2.5 Hz, 1H).
^{13}C NMR (150 MHz, $CDCl_3$) δ 149.0 (d, J = 2.5 Hz), 132.8, 129.6, 129.2, 118.1 (d, J = 28.2 Hz).
^{19}F NMR (565 MHz, $CDCl_3$) δ 62.30.
All analytic data match to the reported data[9].

1.10 Mechanism studies

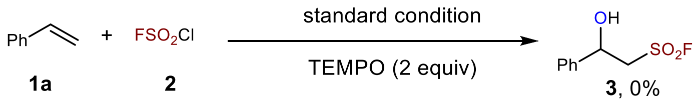

A flask (20 mL) equipped with one aluminum (anode) plate electrode (10 mm x 15 mm x 1 mm), one zinc (cathode) plate electrode (10 mm x 15 mm x 1 mm) and a stir bar was charged with $LiClO_4$ (130 mg, 0.1 M), Et_2O (12 mL, 0.016M), $B_2(OH)_4$ (0.5 mmol, 2.5 equiv), Et_3SiH (0.4 mmol, 2 equiv), **TEMPO** (0.4 mmol, 2 equiv) and **1a** (0.2 mmol). Then, $ClSO_2F$ was added (0.4 mmol, 2 eq, 1 M in anhydrous $PhCF_3$). The mixture was electrolyzed at a constant cell voltage of 8 V for 16 h under an atmosphere of air (1 atm, balloon). The reaction detected by TLC.

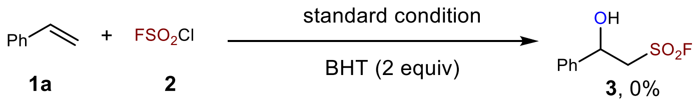

A flask (20 mL) equipped with one aluminum (anode) plate electrode (10 mm x 15 mm x 1 mm), one zinc (cathode) plate electrode (10 mm x 15 mm x 1 mm) and a stir bar was charged with $LiClO_4$ (130 mg, 0.1 M), Et_2O (12 mL, 0.016M), $B_2(OH)_4$ (0.5 mmol, 2.5 equiv), Et_3SiH (0.4 mmol, 2 equiv), **BHT** (0.4 mmol, 2 equiv) and **1a** (0.2 mmol). Then, $ClSO_2F$ was added (0.4 mmol, 2 eq, 1 M in anhydrous $PhCF_3$). The mixture was electrolyzed at a constant cell voltage of 8 V for 16 h under an atmosphere of air (1 atm, balloon). The reaction detected by TLC.

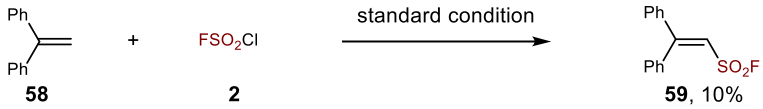

A flask (20 mL) equipped with one aluminum (anode) plate electrode (10 mm x 15 mm x 1 mm), one zinc (cathode) plate electrode (10 mm x 15 mm x 1 mm) and a stir bar was charged with $LiClO_4$ (130 mg, 0.1 M), Et_2O (12 mL, 0.016M) and **58** (0.2 mmol). Then, $ClSO_2F$ was added (0.4 mmol, 2 eq, 1 M in anhydrous $PhCF_3$). The mixture was electrolyzed at a constant cell voltage of 8 V for 16 h under an atmosphere of air (1 atm, balloon). Subsequently, the reaction was quenched with water and electrodes were rinsed with EtOAc. The resulting mixture was extracted with EtOAc and the combined organic layers were dried over Na_2SO_4 and concentrated in vacuo. The residue was purified by column chromatography to afford the product **59** (5.2 mg, 10%) (PE/DCM/EA = 200/120/1).

2,2-Diphenylethene-1-sulfonyl fluoride (59)

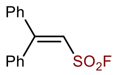

^{1}H NMR (600 MHz, $CDCl_3$) δ 7.51 – 7.45 (m, 4H), 7.40 (t, J = 7.8 Hz, 2H), 7.34 – 7.30 (m, 4H), 6.84 (s, 1H).
All analytic data match to the reported data[9].

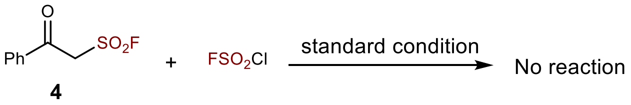

A flask (20 mL) equipped with one aluminum (anode) plate electrode (10 mm x 15 mm x 1 mm), one zinc (cathode) plate electrode (10 mm x 15 mm x 1 mm) and a stir bar was charged with $LiClO_4$ (130 mg, 0.1 M), Et_2O (12 mL, 0.016M) , $B_2(OH)_4$ (0.5 mmol, 2.5 equiv), Et_3SiH (0.4 mmol, 2 equiv) and **4** (0.2 mmol). Then, $ClSO_2F$ was added (0.4 mmol, 2 eq, 1 M in anhydrous $PhCF_3$). The mixture was electrolyzed at a constant cell voltage of 8 V for 16 h under an atmosphere of air (1 atm, balloon). The reaction detected by TLC.

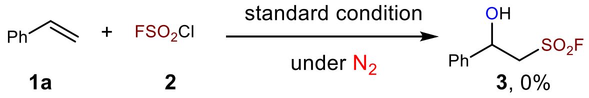

A flask (20 mL) equipped with one aluminum (anode) plate electrode (10 mm x 15 mm x 1 mm), one zinc (cathode) plate electrode (10 mm x 15 mm x 1 mm) and a stir bar was charged with $LiClO_4$ (130 mg, 0.1 M), $B_2(OH)_4$ (0.5 mmol, 2.5 equiv), Et_2O (12 mL, 0.016M), Et_3SiH (0.4 mmol, 2 equiv), and **1a** (0.2 mmol), Then, $ClSO_2F$ was added (0.4 mmol, 2 eq, 1 M in anhydrous $PhCF_3$) under nitrogen. The mixture was electrolyzed at a constant cell voltage of 8 V for 16 h. The reaction detected by TLC.

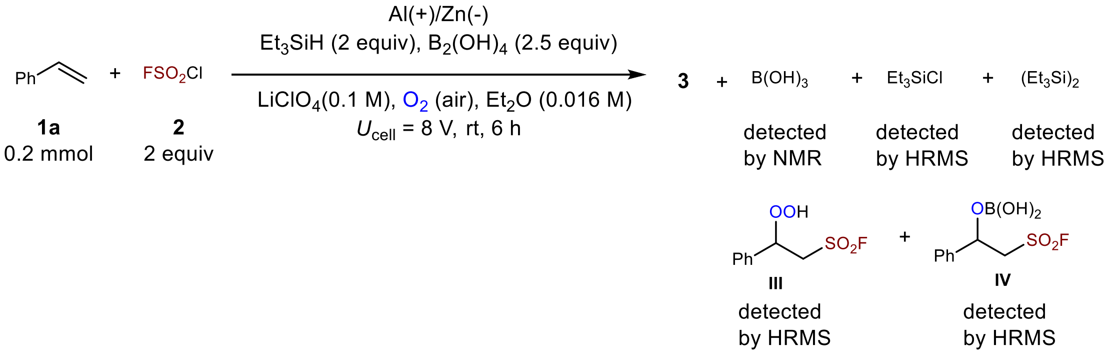

A flask (20 mL) equipped with one aluminum (anode) plate electrode (10 mm x 15 mm x 1 mm), one zinc (cathode) plate electrode (10 mm x 15 mm x 1 mm) and a stir bar was charged with $LiClO_4$ (130 mg, 0.1 M), Et_2O (12 mL, 0.016M), $B_2(OH)_4$ (0.5 mmol, 2.5 equiv), Et_3SiH (0.4 mmol, 2 equiv) and **1a** (0.2 mmol). Then, $ClSO_2F$ was added (0.4 mmol, 2 eq, 1 M in anhydrous $PhCF_3$). The mixture was electrolyzed at a constant cell voltage of 8 V for **6 h** under an atmosphere of air (1 atm, balloon).

$B(OH)_3$ in reaction:

^{11}B NMR (193 MHz, DMSO) δ19.6.

All analytic data match to the reported data[10].

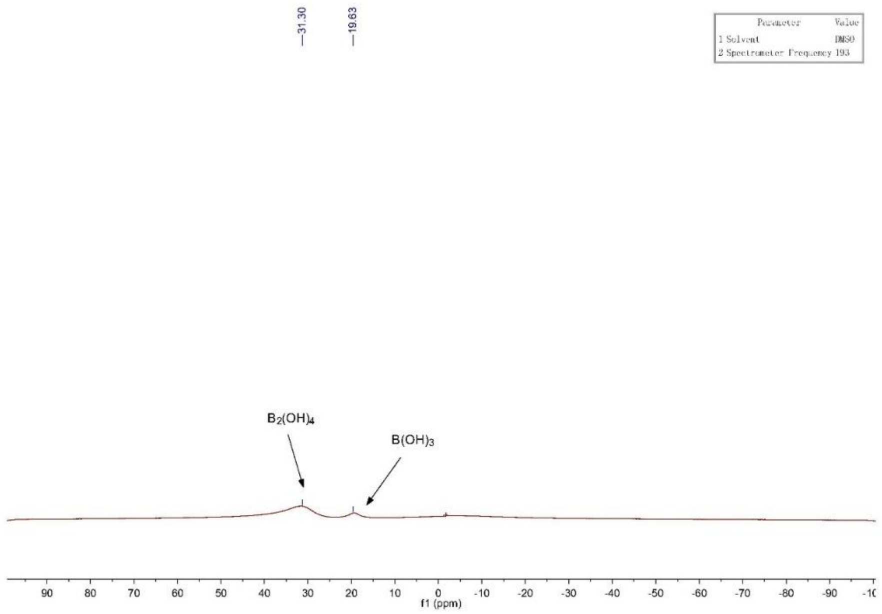

Supplementary Figure 3. ^{11}B NMR for reaction

Et_3SiCl in reaction:

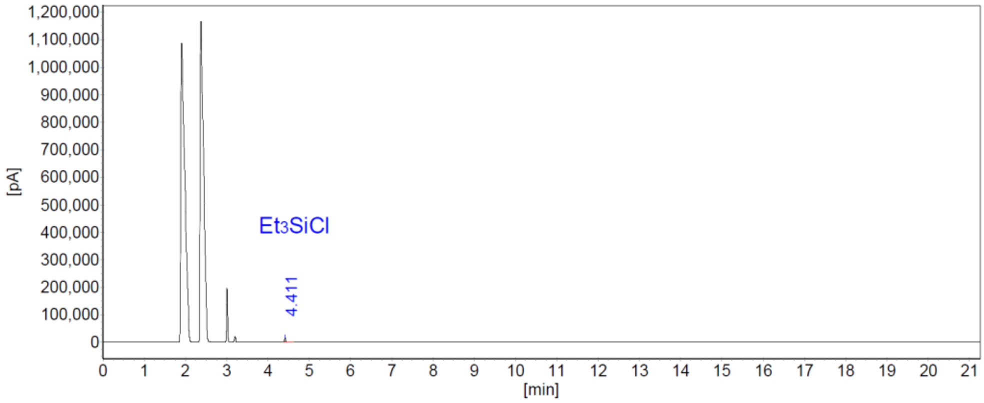

Supplementary Figure 4. GC for reaction

HRMS-ESI (m/z) $[M+Na]^+$ calculated for $C_6H_{15}ClNaSi$ 173.0524, found 173.0522.

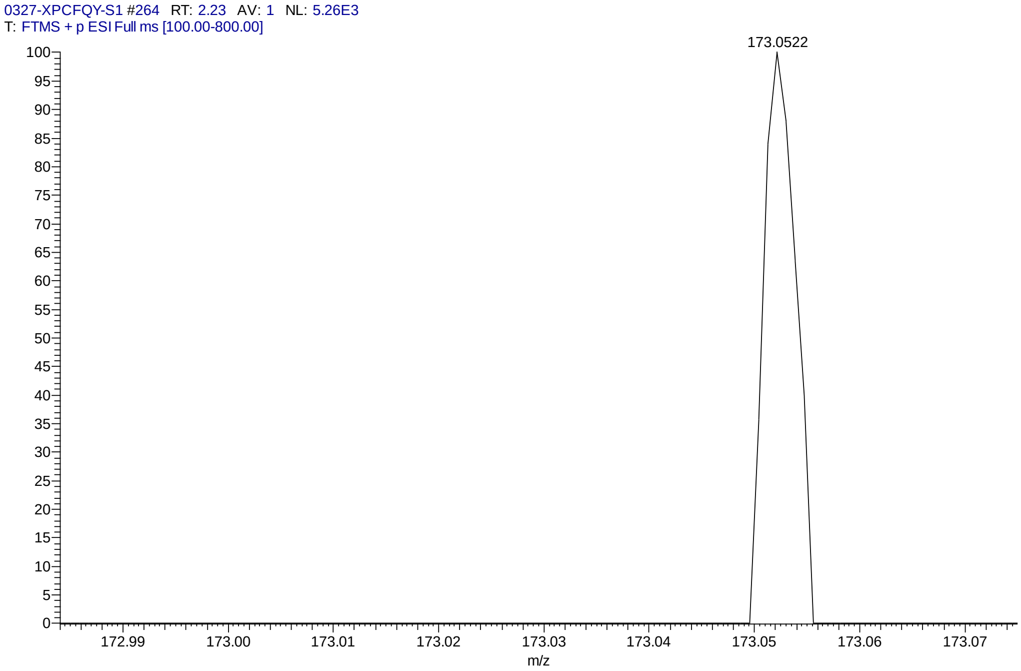

Supplementary Figure 5. HRMS-ESI for reaction

$(Et_3Si)_2$ in reaction:

HRMS-ESI (m/z) $[M+Na]^+$ calculated for $C_{12}H_{30}NaSi_2$ 253.1778, found 253.1774.

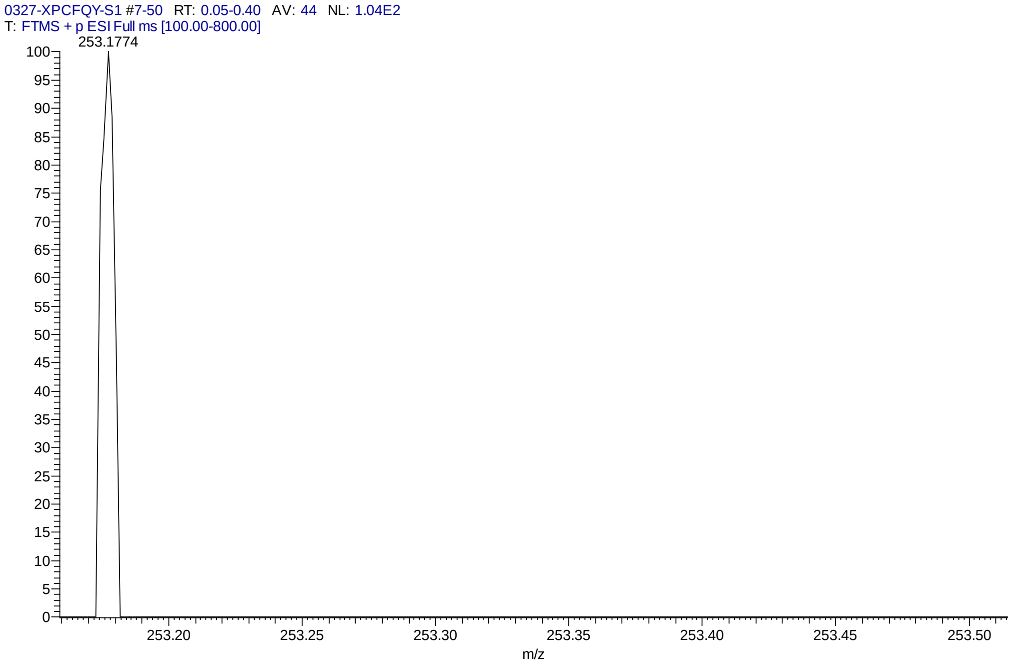

Supplementary Figure 6. HRMS-ESI for reaction

III in reaction:

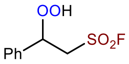

III

HRMS-ESI (m/z) $[M+Na]^+$ calculated for $C_8H_9FNaO_4S$ 243.0098, found 243.0087.

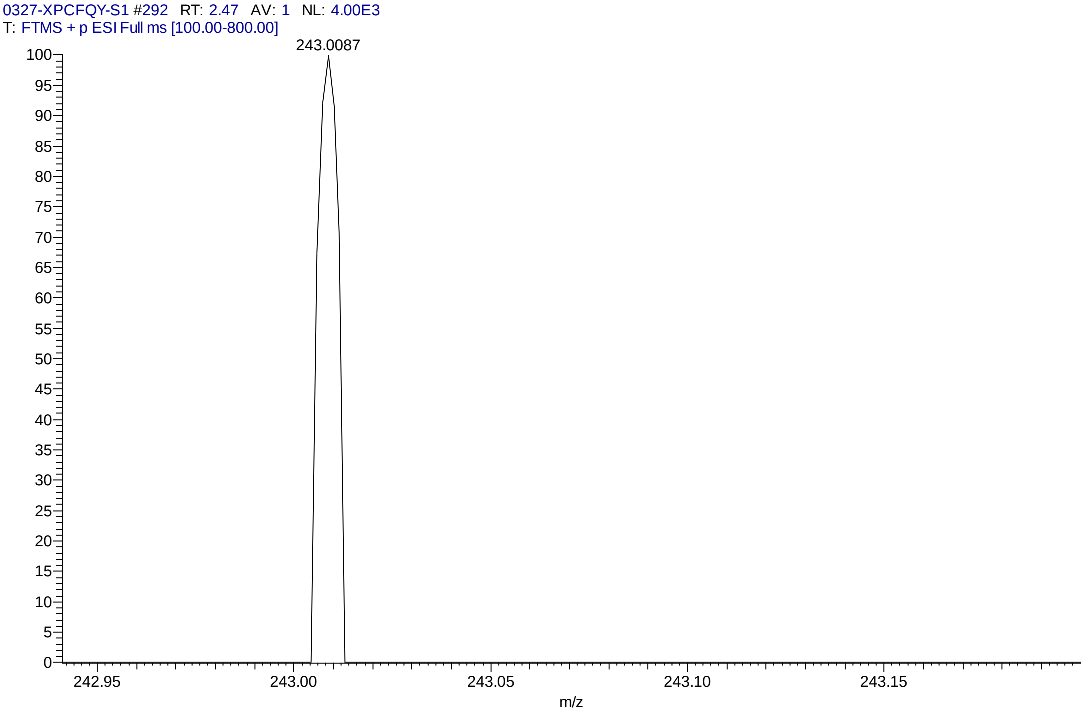

Supplementary Figure 7. HRMS-ESI for reaction

VI in reaction:

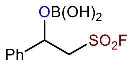

VI

HRMS-ESI (m/z) $[M+Na]^+$ calculated for $C_8H_{10}BFNaO_5S$ 271.0218, found 271.0225.

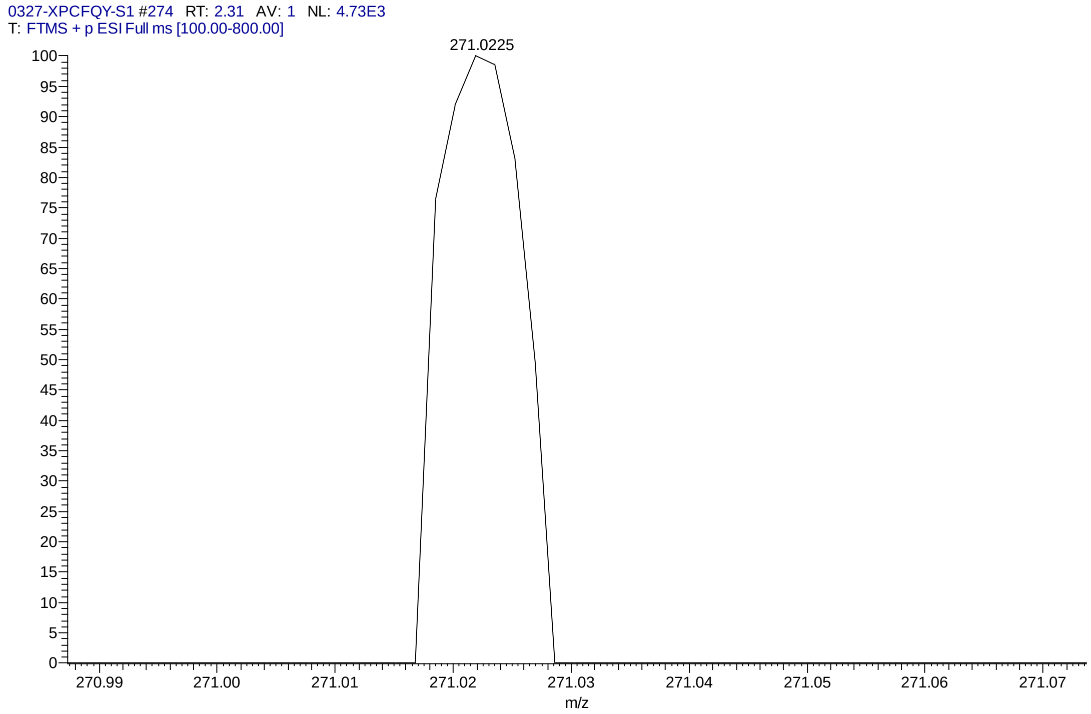

Supplementary Figure 8. HRMS-ESI for reaction

Cyclic voltammentry studies:

Cyclic voltammetry was obtained using a glassy carbon working electrode, a platinum wire counter electrode, Ag/AgCl reference electrode or aqueous saturated calomel electrode (SCE) on CHI660E station. The scan rate was 0.10 V/s.

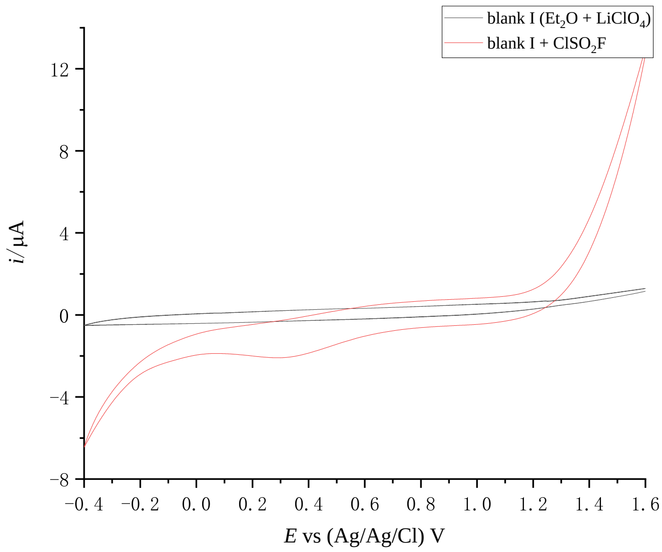

Supplementary Figure 9. Cyclic voltammetry of $ClSO_2F$. (a): (black line) blank I Et_2O (12 mL) + $LiClO_4$ (6 mmol). (b): (red line) blank I + $ClSO_2F$ (0.4 mmol).

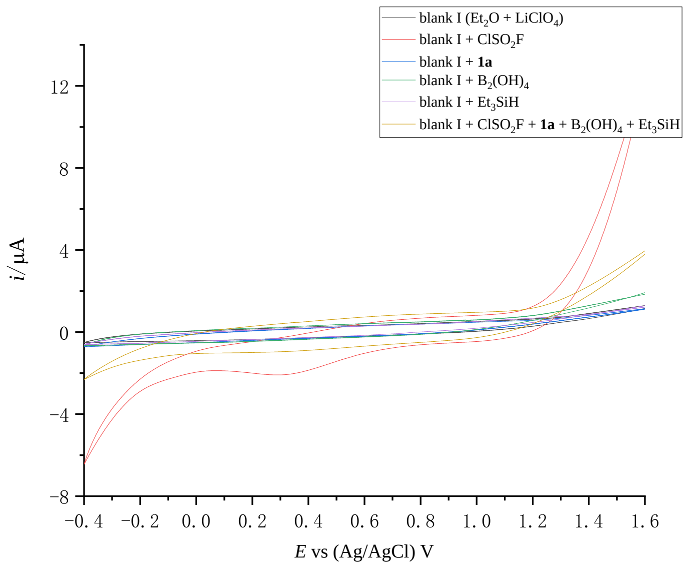

Supplementary Figure 10. Cyclic voltammetry studies. (a): (black line) blank I Et_2O (12 mL) + $LiClO_4$ (6 mmol). (b): (red line) blank I + $ClSO_2F$ (0.4 mmol). (c): (blue line) blank I +Et_3SiH (0.4 mmol). (d): (green line) blank I + $B_2(OH)_4$ (0.5 mmol). (e): (purple line) blank I + **1a** (0.2 mmol). **f.**(yellow line) blank I + **1a** (0.2 mmol) + $ClSO_2F$ (0.4 mmol) + Et_3SiH (0.4 mmol) + $B_2(OH)_4$ (0.5 mmol).

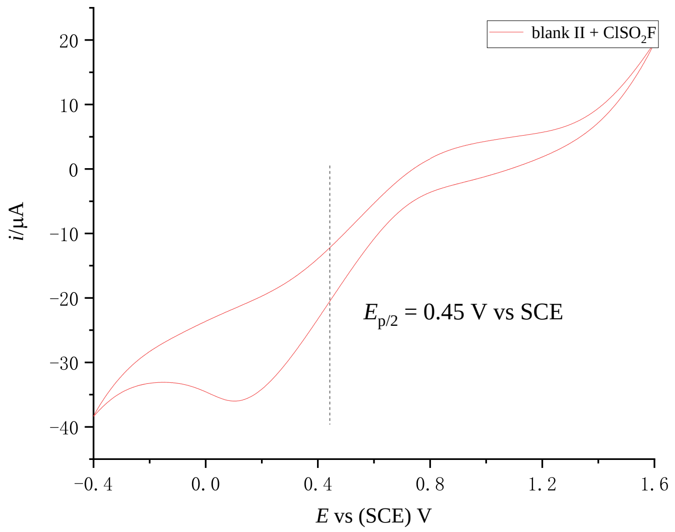

Supplementary Figure 11. Cyclic voltammetry of $ClSO_2F$. blank II: THF (12 mL) + $LiClO_4$ (3 mmol); (red line) blank II + $ClSO_2F$ (0.4 mmol).

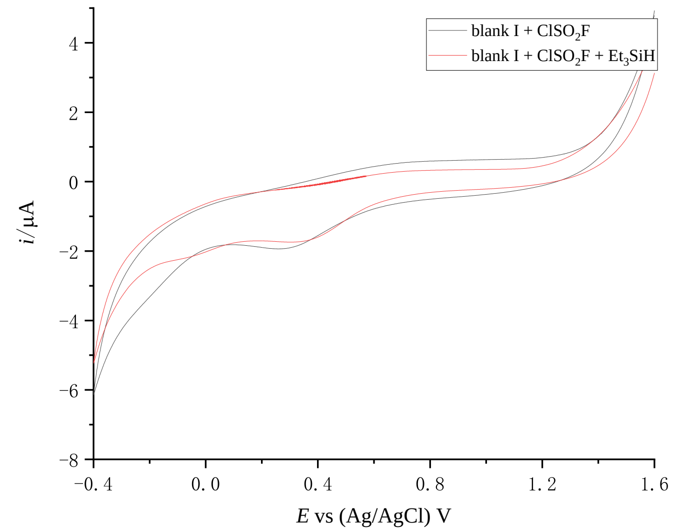

Supplementary Figure 12. Cyclic voltammetry of Et_3SiH. (a): (blank line) blank I + $ClSO_2F$ (0.4 mmol); (b): (red line) blank I + $ClSO_2F$ (0.4 mmol) + Et_3SiH (0.4 mmol).

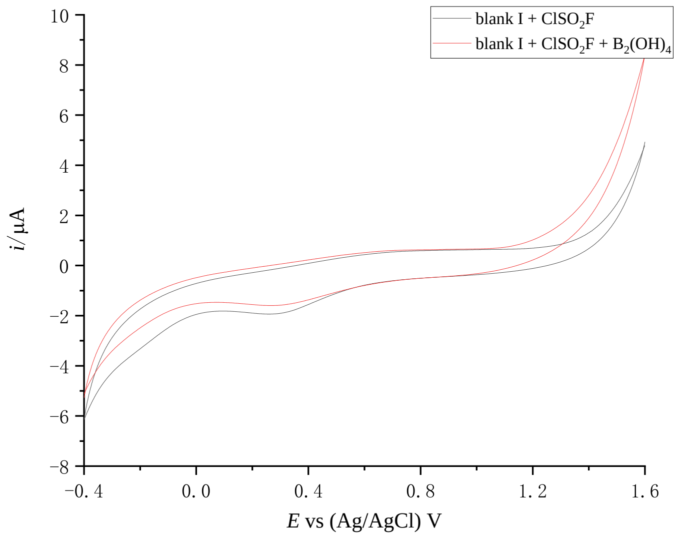

Supplementary Figure 13. Cyclic voltammetry of $B_2(OH)_4$. (a): (blank line) blank I + $ClSO_2F$ (0.4 mmol); (b): (red line) blank I + $ClSO_2F$ (0.4 mmol) + $B_2(OH)_4$ (0.5 mmol).

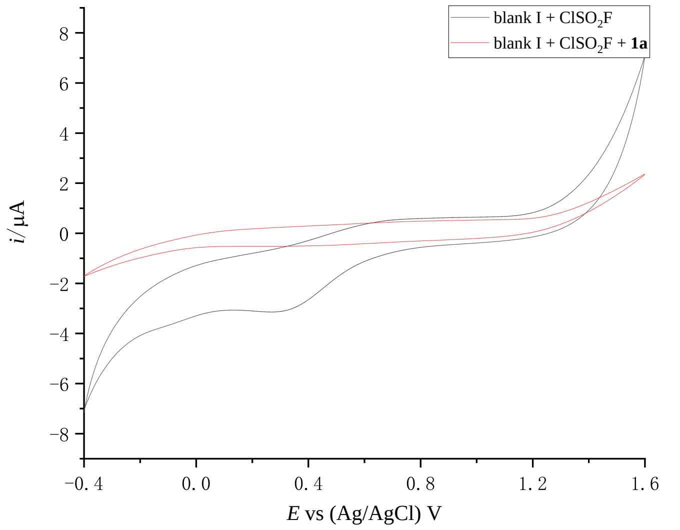

Supplementary Figure 14. Cyclic voltammetry of **1a**. (a): (blank line) blank I + $ClSO_2F$ (0.4 mmol); (b): (red line) blank I + $ClSO_2F$ (0.4 mmol) + **1a** (0.2 mmol).

Constant potential experiments:

Experimental Procedure C: In an oven-dried undivided three-necked flask (25 ml) equipped with a stir bar, $LiClO_4$ (130 mg, 0.1 M), Et_2O (12 mL, 0.016M), $B_2(OH)_4$ (0.5 mmol, 2.5 equiv), Et_3SiH (0.4 mmol, 2 equiv) and **1a** alkenes (0.2 mmol) were combined and added. Then, $ClSO_2F$ was added (0.4 mmol, 2 eq, 1 M in anhydrous $PhCF_3$). The bottle was equipped with one aluminum (anode) plate electrode (10 mm x 15 mm x 1 mm), one zinc (cathode) plate electrode (10 mm x 15 mm x 1 mm) and SCE as reference electrode. The reaction mixture was stirred and electrolyzed

at a constant potential on CHI660E station under room temperature for 16 h. when the reaction was finished, the solution was quenched with water and electrodes were rinsed with EtOAc. The resulting mixture was extracted with EtOAc and the combined organic layers were dried over Na_2SO_4 and concentrated in vacuo. The residue was purified by column chromatography to afford the desired product (PE/DCM/EA = 20/20/1)

Supplementary Table 9. Constant potential electrolysis (vs SCE)

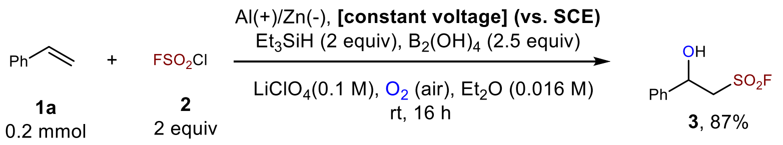

Entry	Voltages (V vs. SCE)	**3**(%)^a
1	-0.1	87
2	-0.5	84
3	-1.0	49

a. isolated yields.

Experimental procedure D (EDA conditions)

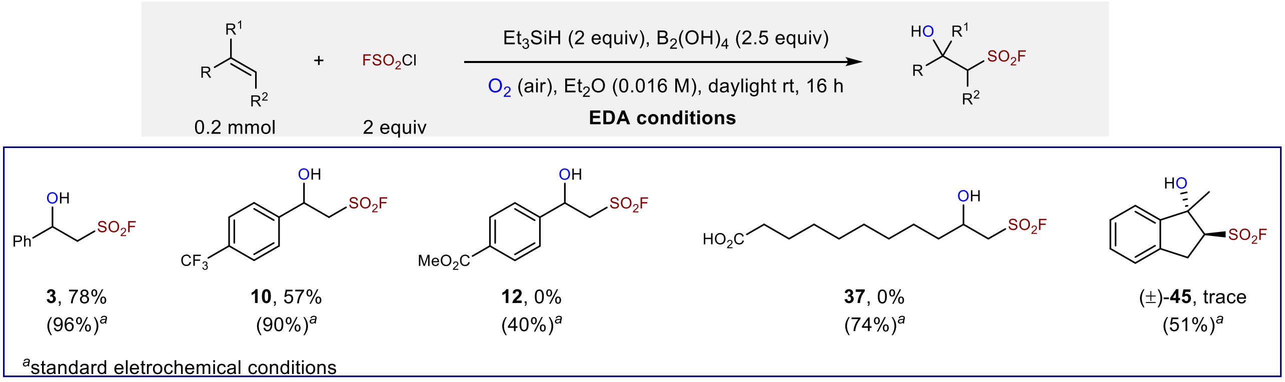

Supplementary Figure 15. Scope of EDA conditons

A 20-mL vial with a stir bar was charged with Et_2O (12 mL, 0.016M), $B_2(OH)_4$ (0.5 mmol, 2.5 equiv), Et_3SiH (0.4 mmol, 2 equiv) and alkenes (0.2 mmol). Then, $ClSO_2F$ was added (0.4 mmol, 2 eq, 1 M in anhydrous $PhCF_3$). The mixture was stirred for 16 h under an atmosphere of air (1 atm, balloon). Subsequently, the reaction was quenched with water and electrodes were rinsed with EtOAc. The resulting mixture was extracted with EtOAc and the combined organic layers were dried over Na_2SO_4 and concentrated in vacuo. The residue was purified by column chromatography to afford the desired product (PE/DCM/EA = 20/20/1 ~ 2/2/1).

Mechanism via EDA photoactivation

a. UV-vis Absorption Spectra between 1a and ClSO$_2$F

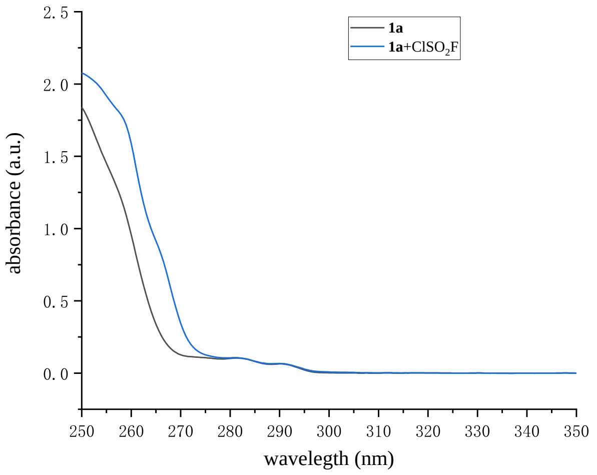

b. Proposed mechanism via EDA photoactivation

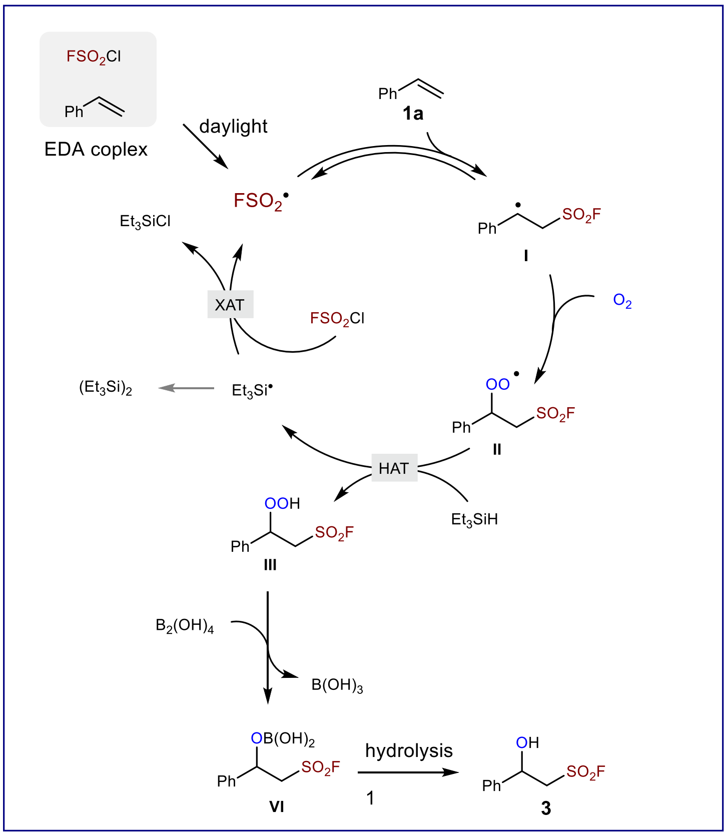

Supplementary Figure 16. Proposed mechanism via EDA photoactivation. **a** UV-vis Absorption Spectra of **1a** (0.016 M in Et_2O) and mixture of **1a** and $ClSO_2F$. **b** Proposed mechanism via EDA photoactivation.

1.11 X-ray crystal data

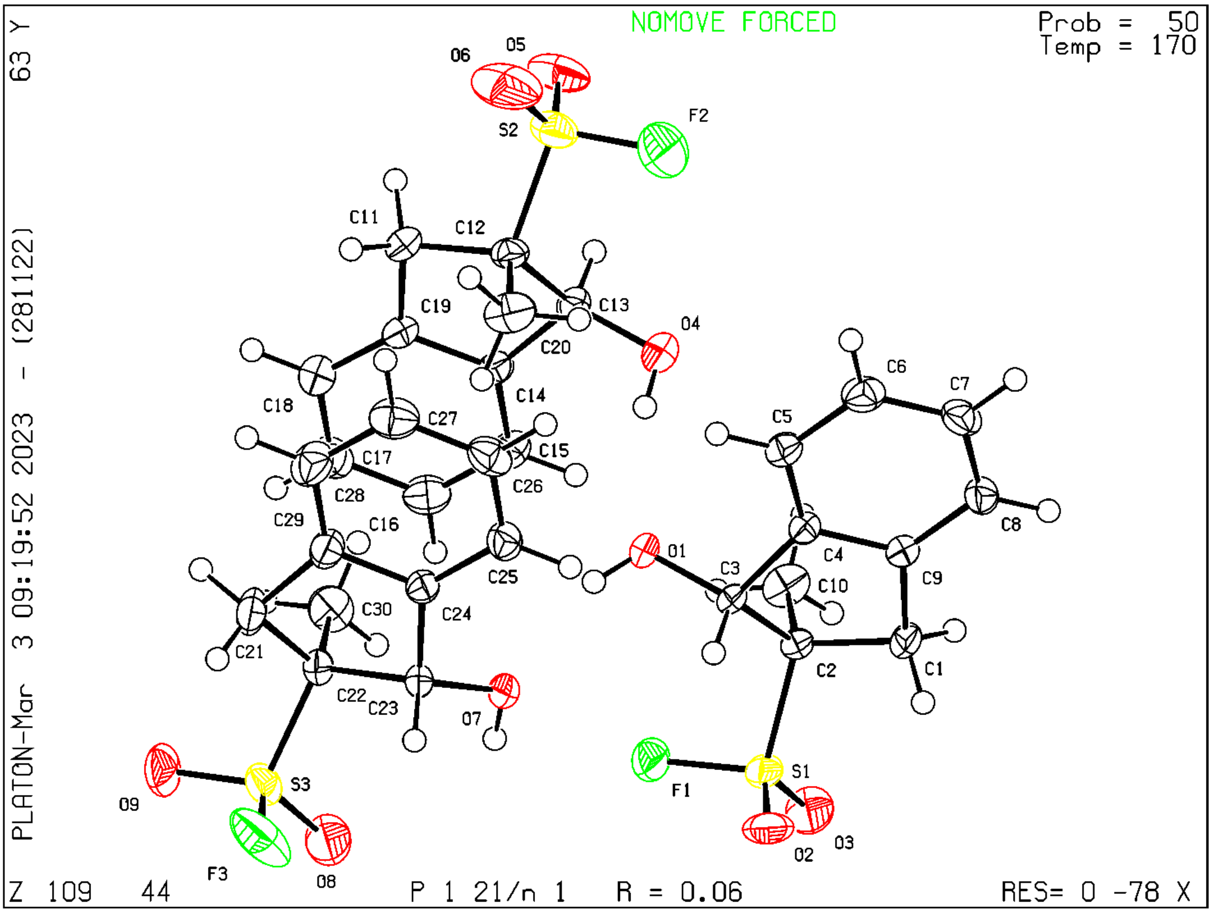

Supplementary Figure 17. X-ray structure of (±)-**46** (2248411)

Supplementary Table 10. Crystal data and structure refinement for (±)-**46.**

Identification code	(±)-**46**
Empirical formula	$C_{10}H_{11}FO_3S$
Formula weight	230.25
Temperature/K	169.99(10)
Crystal system	monoclinic
Space group	$P2_1/n$
a/Å	9.4264(2)
b/Å	10.2280(2)
c/Å	32.5215(6)
α/°	90
β/°	95.956(2)
γ/°	90
Volume/Å^3	3118.58(11)
Z	12

ρ_{calc}g/cm^3	1.471
μ/mm^{-1}	2.804
F(000)	1440.0
Crystal size/mm^3	0.15 × 0.13 × 0.08
Radiation	Cu Kα (λ = 1.54184)
2Θ range for data collection/°	5.464 to 147.766
Index ranges	$-7 \le h \le 11$, $-12 \le k \le 10$, $-37 \le l \le 40$
Reflections collected	12498
Independent reflections	6136 [R_{int} = 0.0451, R_{sigma} = 0.0525]
Data/restraints/parameters	6136/0/410
Goodness-of-fit on F^2	1.031
Final R indexes [I>=2σ (I)]	R_1 = 0.0604, wR_2 = 0.1548
Final R indexes [all data]	R_1 = 0.0660, wR_2 = 0.1610
Largest diff. peak/hole / e Å^{-3}	0.53/-0.97

1.12 Preliminary studies for biological activities

1.12.1 In vitro antifungal activities

Materials and methods

1. Each target compound was dissolved in MeOH to prepare the stock solution (2 mg/mL).
2. The stock solution was added to the PDA medium, and the concentration of target compounds in the medium was 20 μg/mL and 10 μg/mL.
3. Pure MeOH without the target compounds was utilized as the blank control, and Chlorothalonil was coassayed as the reference compound.
4. Fresh dishes with a diameter of 5 mm were taken from the edge of the PDA-cultured fungi colonies and inoculated on the above three PDA media. Each treatment was tested for three replicates, and the antifungal effect was averaged. The relative inhibitory rate I (%) of all the tested compounds was calculated through the equation: I (%) = [(C−T)/(C−5)] × 100. In this equation, I is the inhibitory rate and C and T are the colony diameter of the blank control (mm) and treatment (mm), respectively.

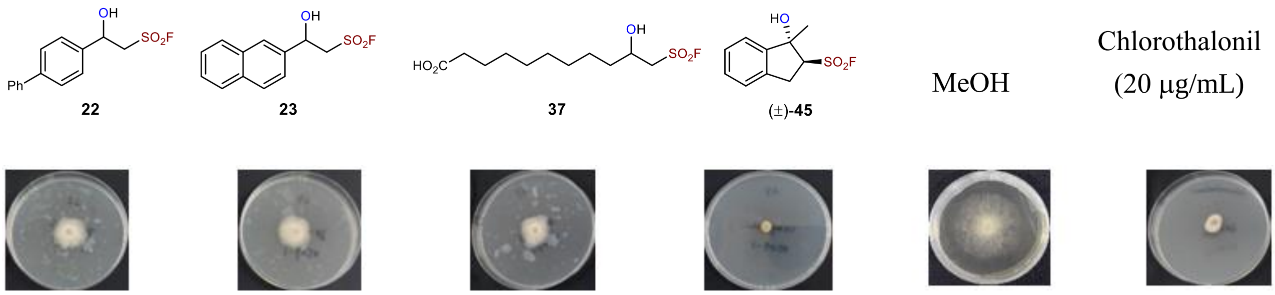

Supplementary Figure 18. In vitro antifungal activities of the target coumpounds against *B. cinerea* in 20 μg/mL

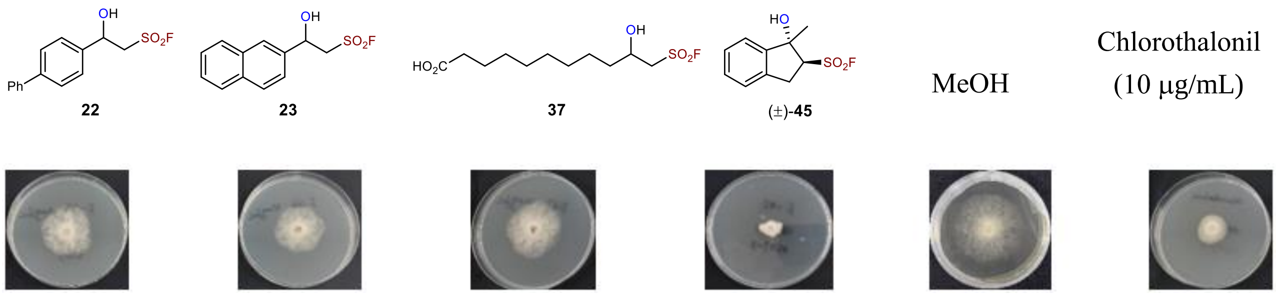

Supplementary Figure 19. In vitro antifungal activities of the target coumpounds against *B. cinerea* in 10 μg/mL

Supplementary Table 11. EC_{50} values of compound **(±)-45**

Conc.	Colony diameter (cm)		
(μg/mL)	1st	2nd	3rd
5.0	17.0	15.0	14.0
1.0	36.5	33.0	36.0
0.5	38.5	43.0	45.5
0.3	42.0	49.0	42.0
0.1	42.5	50.0	46.0
blank control	52.0	55.0	51.0

a

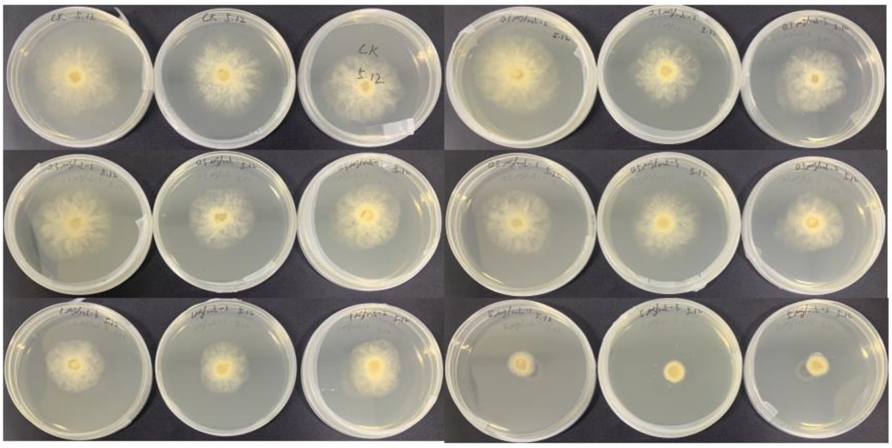

b

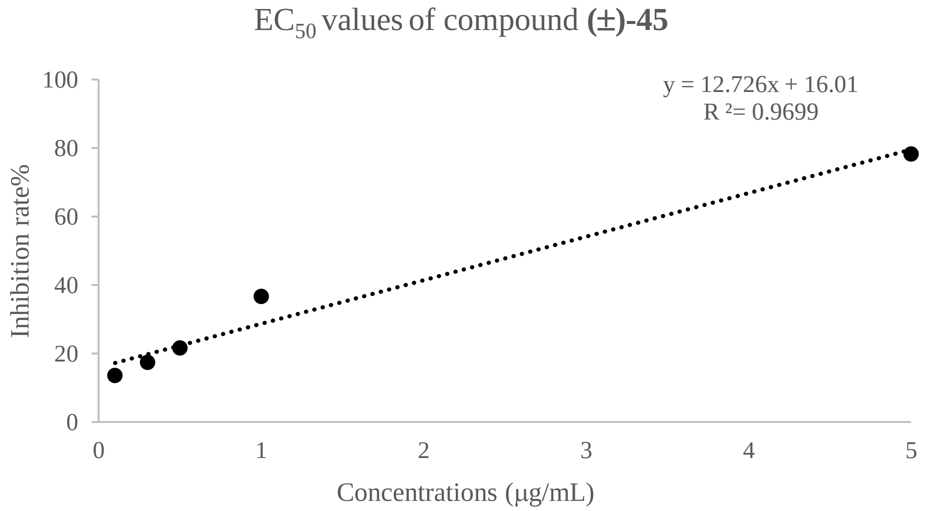

Supplementary Figure 20. In vitro antifungal activities of compound **(±)-45** against *B. cinerea* at various concentrations. **a** Colonoies of *B. cinerea* on PDA plates supplenmented with different concentrations (0.1, 0.3, 0.5, 1.0 and 5.0 μg/mL). **b** The regression equation for the standard curve.

Supplementary Table 12. Structure-activity relationship of compound (±)-**45**

Compound	*B. cinerea* (inhibition rate/%)
	20 μg/mL
**45**	100.0 ± 0.0
**S2**[11]	0.2 ± 1.1
**S3**	92.4 ± 0.9
**S4**[9]	9.7 ± 3.0
**S5**[12]	-0.1 ± 1.4

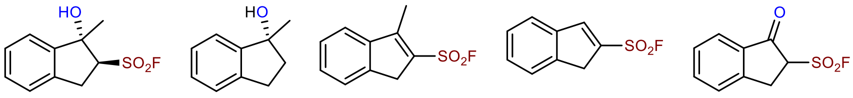

(±)-**45** **S2** **S3** **S4** **S5**

Syntheise of **S3**:

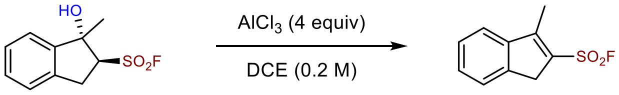

(±)-**45** S3

A 10 mL vial with a stir bar was charged compound **(±)-45** (47 mg, 0.2 mmol), DCE (2 mL, 0.2 M). $AlCl_3$ (107.8 mg, 4 equiv) was added, and the reaction mixture was stirred at 50 °C for 4 h. The crude mixture was concentrated in vacuo and the residue was purified by column chromatography to afford 3-Methyl-1*H*-indene-2-sulfonyl fluoride **S3** as a yellow solid (20 mg, 47%).

3-Methyl-1*H*-indene-2-sulfonyl fluoride (S3)

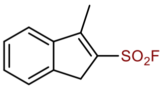

^{1}H NMR (600 MHz, $CDCl_3$) δ 7.61 (d, *J* = 7.0 Hz, 1H), 7.56 (d, *J* = 7.3 Hz, 1H), 7.52 – 7.45 (m, 2H), 3.89 (s, 2H), 2.61 (dd, *J* = 4.4, 2.3 Hz, 3H).

^{3}C NMR (150 MHz, $CDCl_3$) δ 156.2 (d, *J* = 3.2 Hz), 142.7 (d, *J* = 0.7 Hz), 142.6 (d, *J* = 1.8 Hz), 130.0 128.1 (d, *J* = 26.0 Hz), 127.7, 124.5, 122.5, 39.3, 12.2.

^{19}F NMR (565 MHz, $CDCl_3$) δ 67.43.

HRMS-ESI (m/z) $[M-H]^-$ calculated for $C_{10}H_8FO_2S$ 211.0235, found 211.0236.

Supplementary Table 13. In vitro antifungal activities of β-hydroxy sulfonyl fluorides and β-keto sulfonyl fluorides

Compound	*B. cinerea* (inhibition rate/%)
	20 μg/mL
**22**	56.0 ± 4.4
**S6**[13]	1.3 ± 1.9
**23**	50.4 ± 8.8
**S7**[8]	9.1 ± 1.9
**37**	60.3 ± 4.4
**S8**	2.0 ± 1.1
**45**	100.0 ± 0.0
**S5**[12]	-0.1 ± 1.4

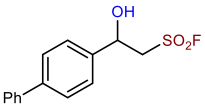

22

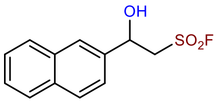

23

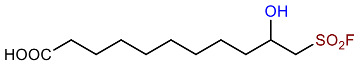

37

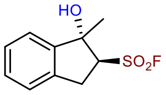

(±)-**45**

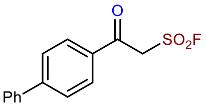

S6

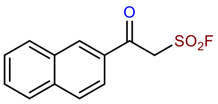

S7

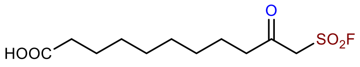

S8

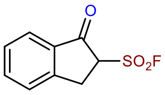

S5

Syntheise of **S8**:

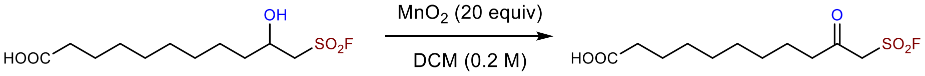

A 10 mL vial with a stir bar was charged compound **37** (29.0 mg, 0.2 mmol), DCM (0.5 mL, 0.2 M). MnO_2 (189.0 mg, 20 equiv) was added, and the reaction mixture was stirred at room temperature for 8 h. The crude mixture was concentrated in vacuo and the residue was purified by column chromatography to afford **S8** as a white solid (24.0 mg, 85%).

11-(Fluorosulfonyl)-10-oxoundecanoic acid (S8)

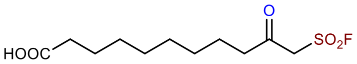

^{1}H NMR (600 MHz, ^{d}Acetone) δ 10.47 (bs, 1H), 5.11 (d, J = 2.9 Hz, 2H), 2.75 (t, J = 7.3 Hz, 2H), 2.27 (t, J = 7.4 Hz, 2H), 1.63 – 1.57 (m, 4H), 1.34 – 1.31 (m, 8H).

^{13}C NMR (150 MHz, ^{d}Acetone) δ197.0, 174.7, 60.8 (d, J = 13.6 Hz), 43.5 (d, J = 3.2 Hz), 34.2, 30.4, 29.9, 29.8, 29.5, 25.6, 23.6.

^{19}F NMR (565 MHz, ^{d}Acetone) δ 60.50.

HRMS-ESI (m/z) $[M+Na]^+$ calculated for $C_{11}H_{19}FNaO_5S$ 305.0829, found 305.0835.

1.12.2 In vitro anti-*Bursaphelenchus xylophilus*

Materials and methods

B. xylophilus is provided by Co-Innovation Center for Sustainable Forestry in Southerm China, Nanjing Forestry University.

1. Nematodes were considered dead when they were stiff and does not respond to acupuncture. In this experiment, those who kept swinging or showed "S" shape, curl shape, wave shape or spiral shape were judged to be living insects, and those who did not move and showed "J" or "C" shapes, or those with stiff bodies were judged to be dead insects.
2. Each target compound was dissolved in MeOH to prepare the stock solution (2 mg/mL).
3. Prepare the test solution (200 μg/mL): 100 μL stock solution + 900 μL water.
4. The nematodes were collected and diluted to approximately 2000 nematodes mL^{-1}.
5. Mix the test solution with nematodes (100 μL + 100 μL) and left for 24 hours at 25°C protected against exposure to light. The kill rates were checked with an optical microscope.

(Each compound was tested for 3 times)

Supplementary Table 14. The insecticidal activity of target compounds against Pinewood nematode, *B. xylophilus* (24 h)

Compd.	*B. xylophilus* (mortality/%) (100 μg/mL)			
	1st	2nd	3rd	Mortality (%) ± SD
**3**	51.6	50	45.8	49.1 ± 3.0
**5**	96.2	92.3	90.5	93.0 ± 2.9
**6**	42.8	50	51.6	48.1 ± 4.7
**7**	70	60	50	60.0 ± 10.0
**9**	78.8	81.6	89.2	83.2 ± 5.4
**10**	68.6	75	68	70.5 ± 3.9
**13**	60	61.4	73.7	65.0 ± 7.5
**15**	75	86.1	69.8	77.0 ± 8.3
**16**	69	67.4	67.8	68.1 ± 0.8
**19**	71.4	74	63.5	69.6 ± 5.5
**22**	0	0	0	0
**23**	19.2	25.9	19.2	21.4 ± 3.9
**35**	100	100	100	100
**45**	0	0	0	0

Supplementary Table 15. LC_{50} values of compound **35**

Conc.	*B. xylophilus* (mortality/%)			
(μg/mL)	1st	2nd	3rd	Mortality (%) ± SD
80	83.5	75.4	84.3	81.1 ± 4.9
60	82.3	69.4	72.9	74.9 ± 6.7
50	71.6	77.8	59.9	69.8 ± 9.1
40	57.3	57.2	64.0	59.5 ± 3.9
30	52.4	58.6	54.3	55.1 ± 3.2
blank control	0	0	0	0

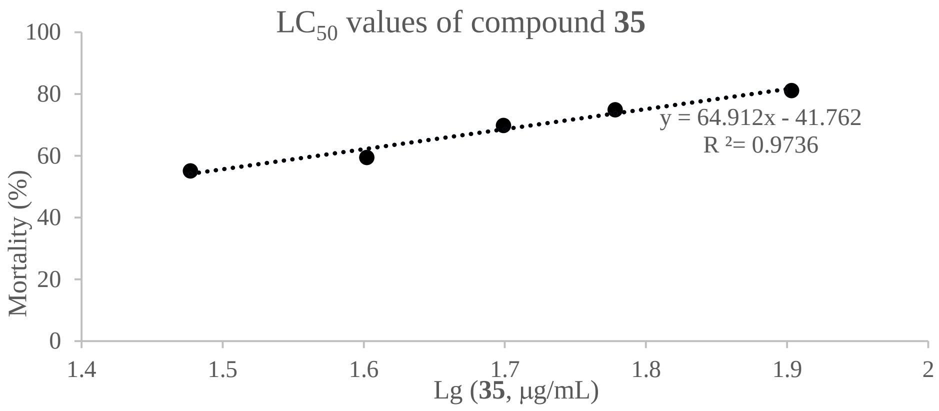

Supplementary Figure 21. The regression equation for the standard curve.

Supplementary Table 16. In vitro anti-*Bursaphelenchus xylophilus* of β-hydroxy sulfonyl fluorides and β-keto sulfonyl fluorides

Compound	*B. xylophilus* (mortality/%) (100 μg/mL)
**10**	70.5 ± 3.9
**S9**[12]	29.5 ± 15.6
**23**	21.4 ± 3.9
**S7**[8]	0.15 ± 0.7

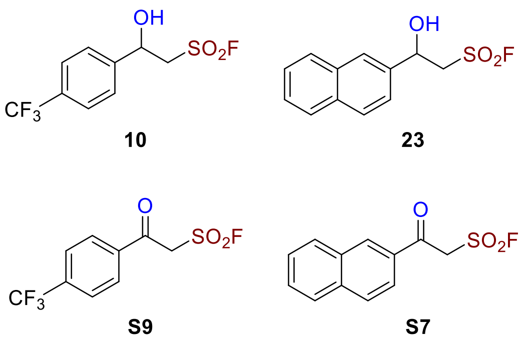

2. Supplementary NMR spectra

Supplementary Figure 22. ^{1}H, ^{13}C and ^{19}F NMR spectra of **3**.

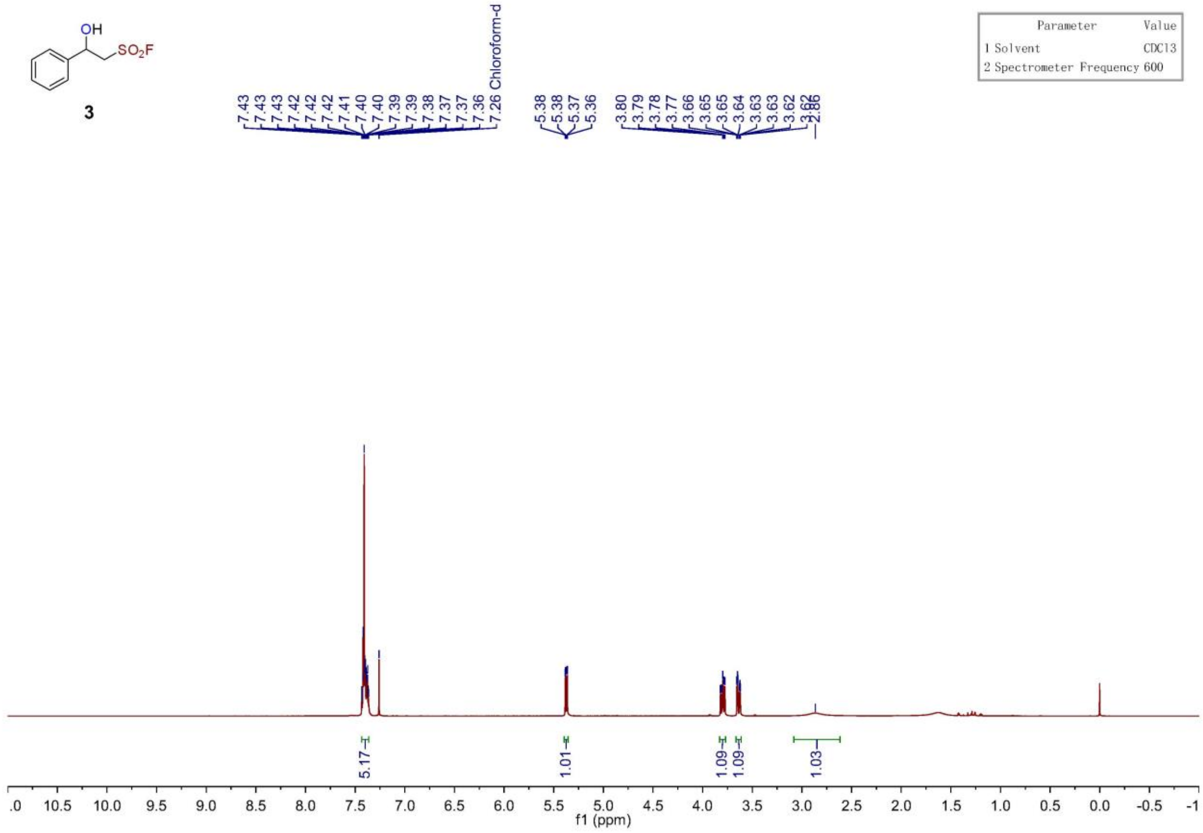

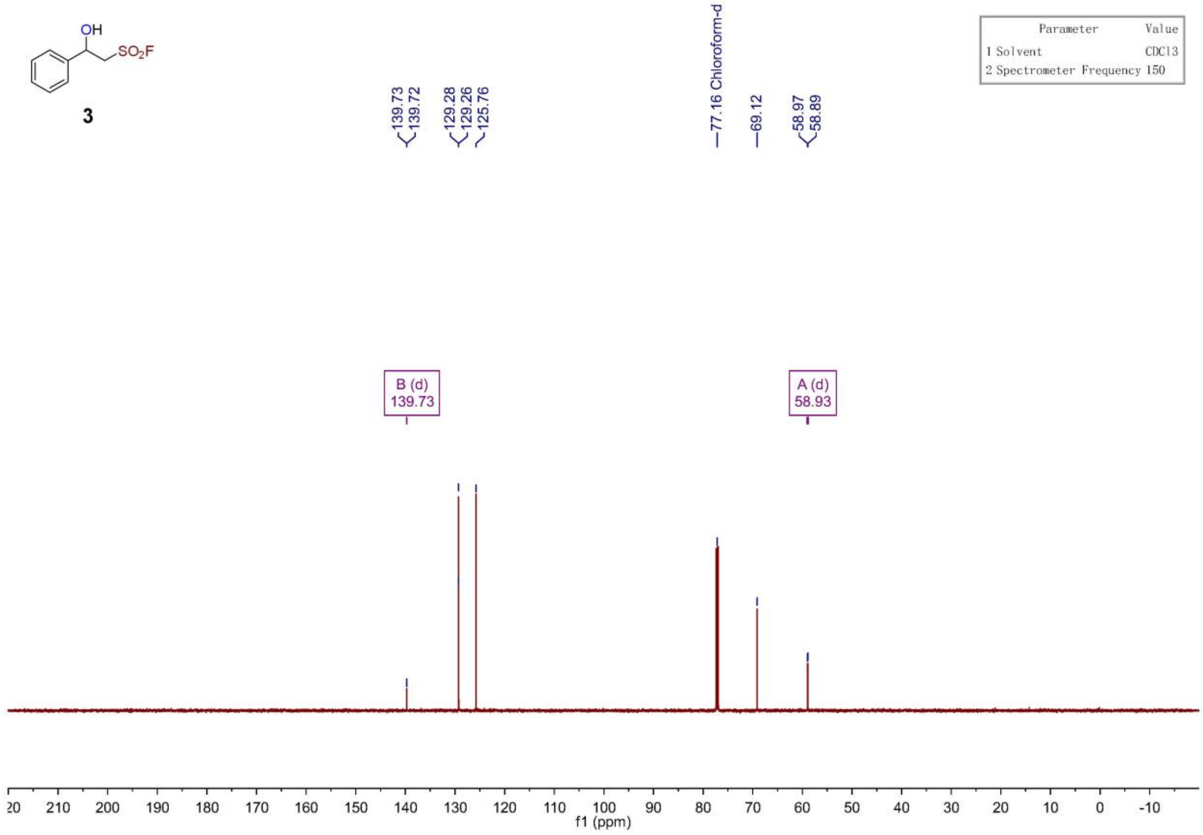

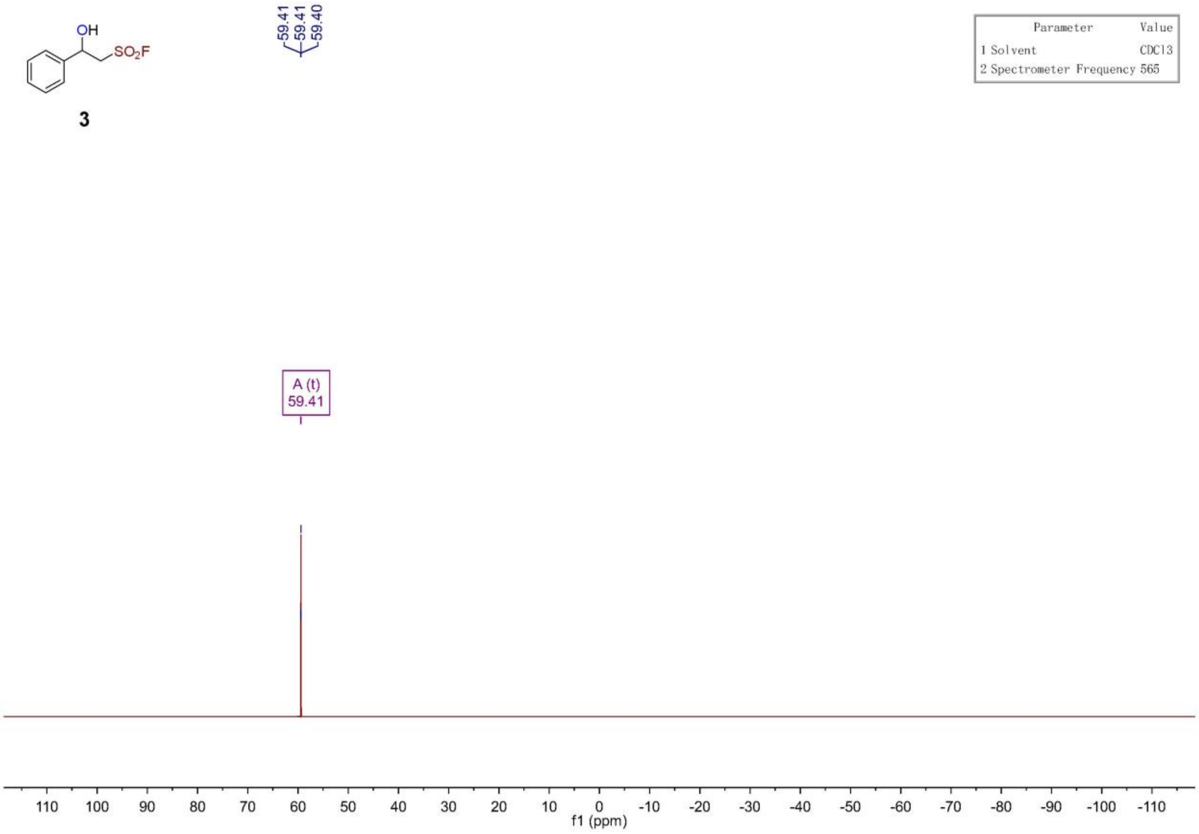

Supplementary Figure 23. ^{1}H, ^{13}C and ^{19}F NMR spectra of **5**.

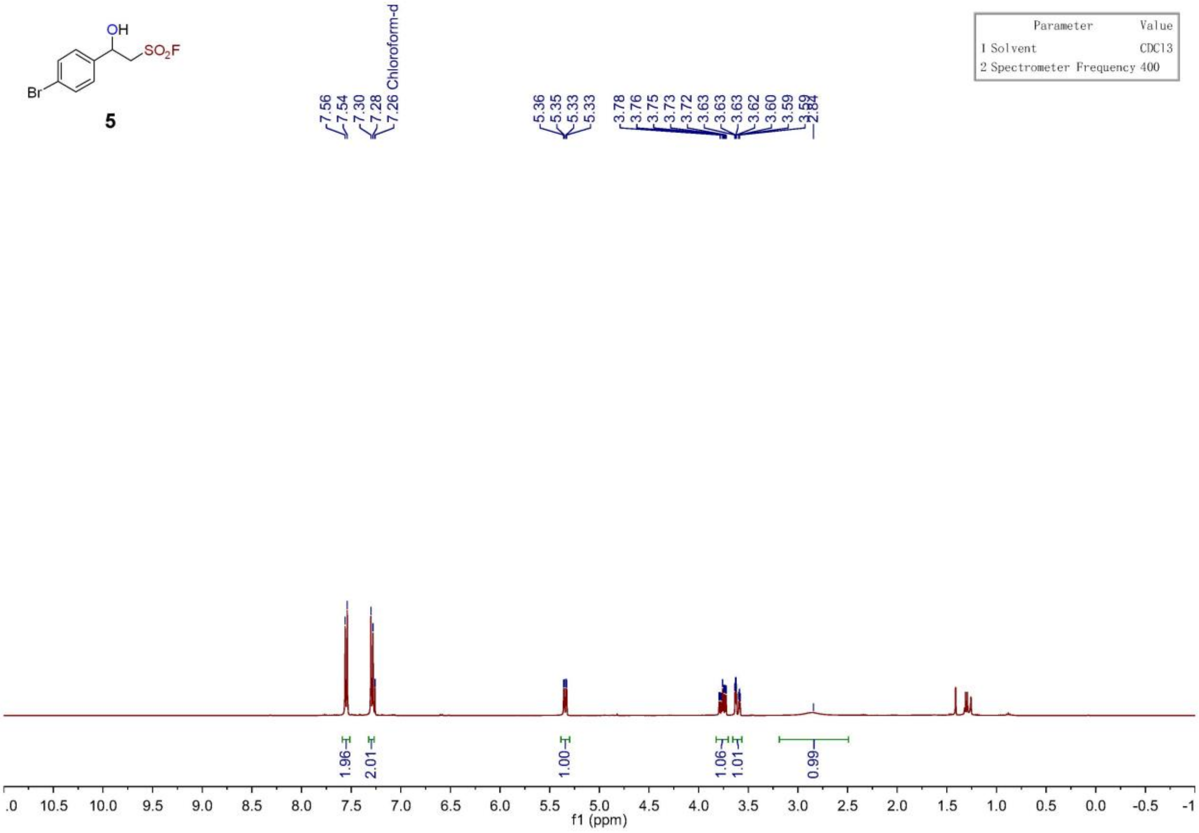

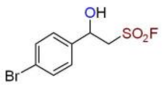

5

Parameter		Value
1	Solvent	CDCl3
2	Spectrometer Frequency	100

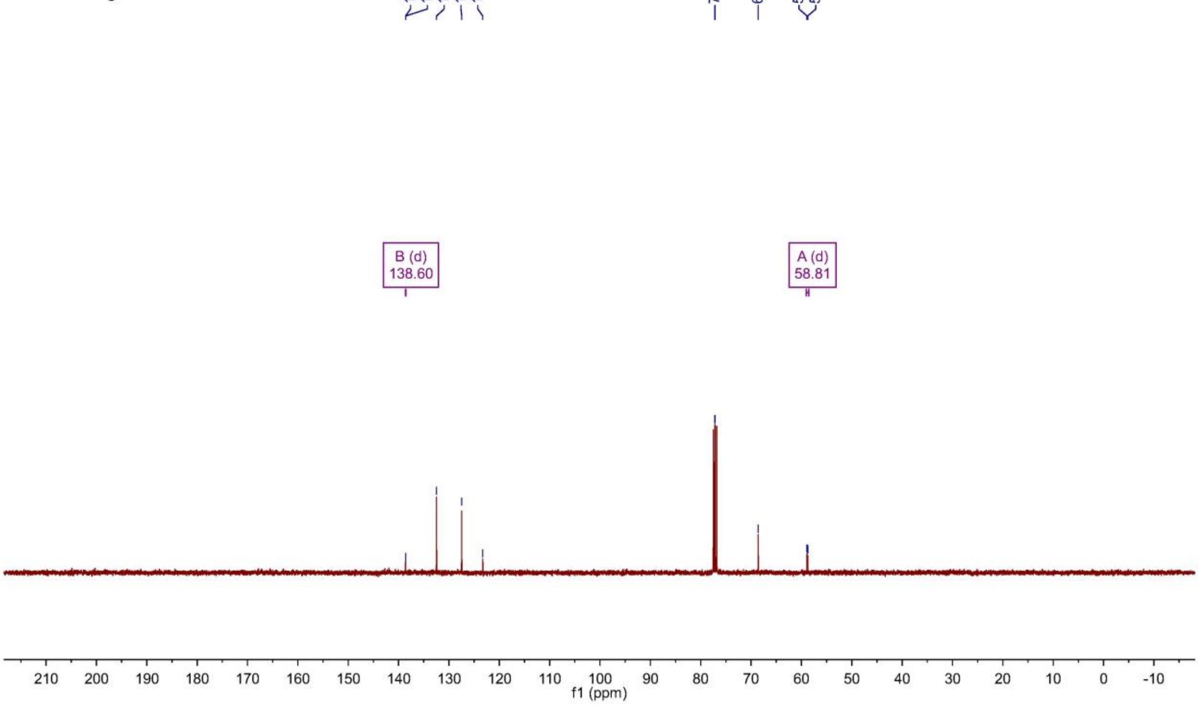

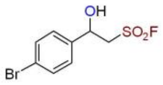

5

Parameter		Value
1	Solvent	CDCl3
2	Spectrometer Frequency	377

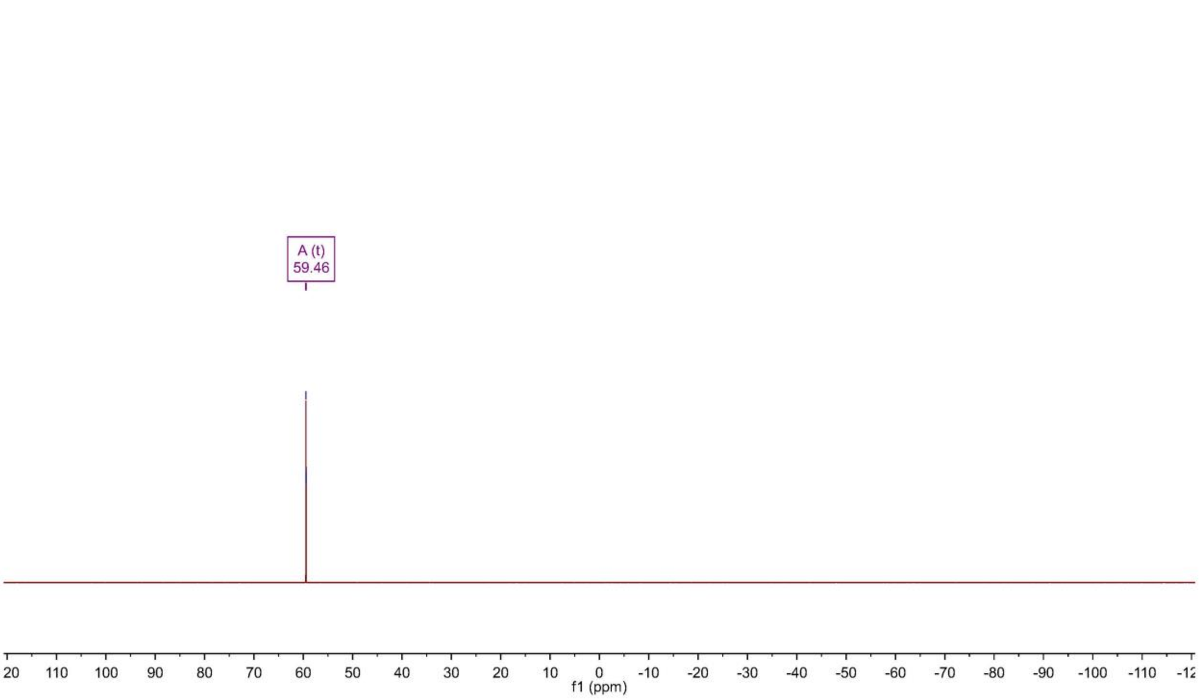

Supplementary Figure 24. ^{1}H, ^{13}C and ^{19}F NMR spectra of **6**.

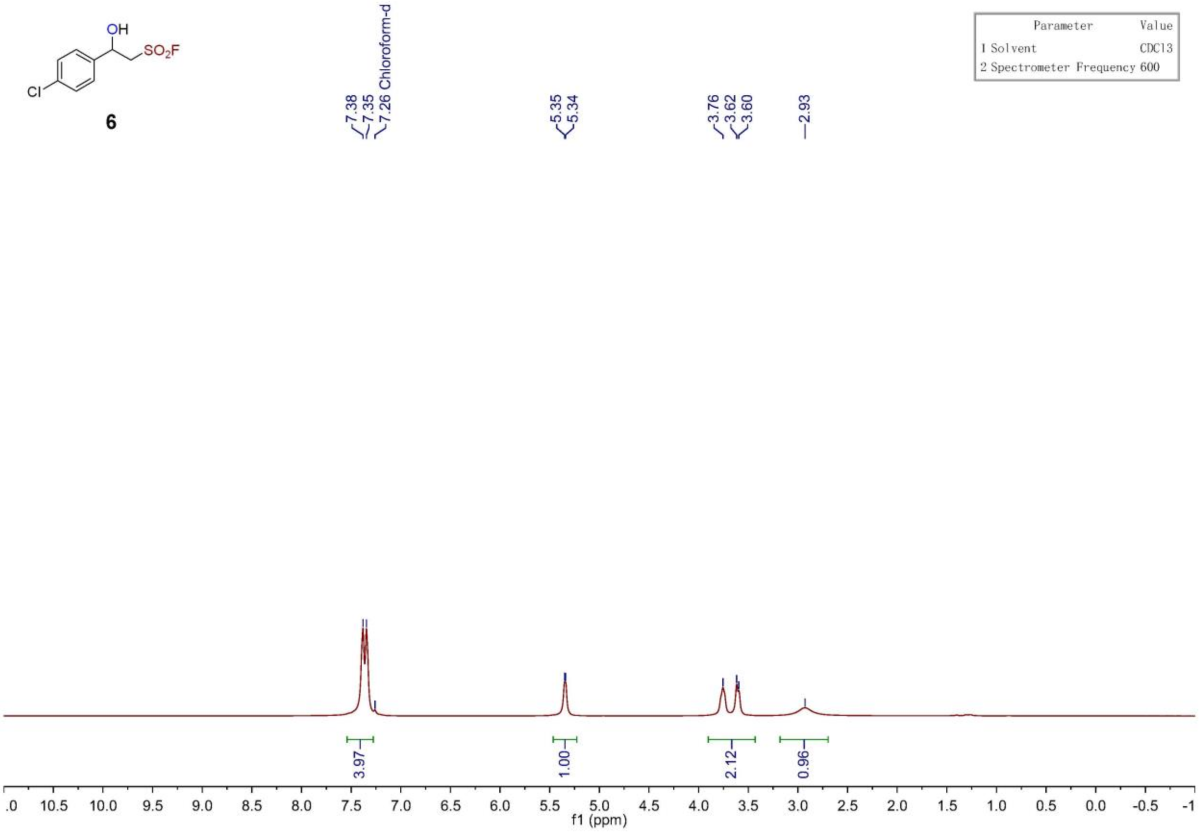

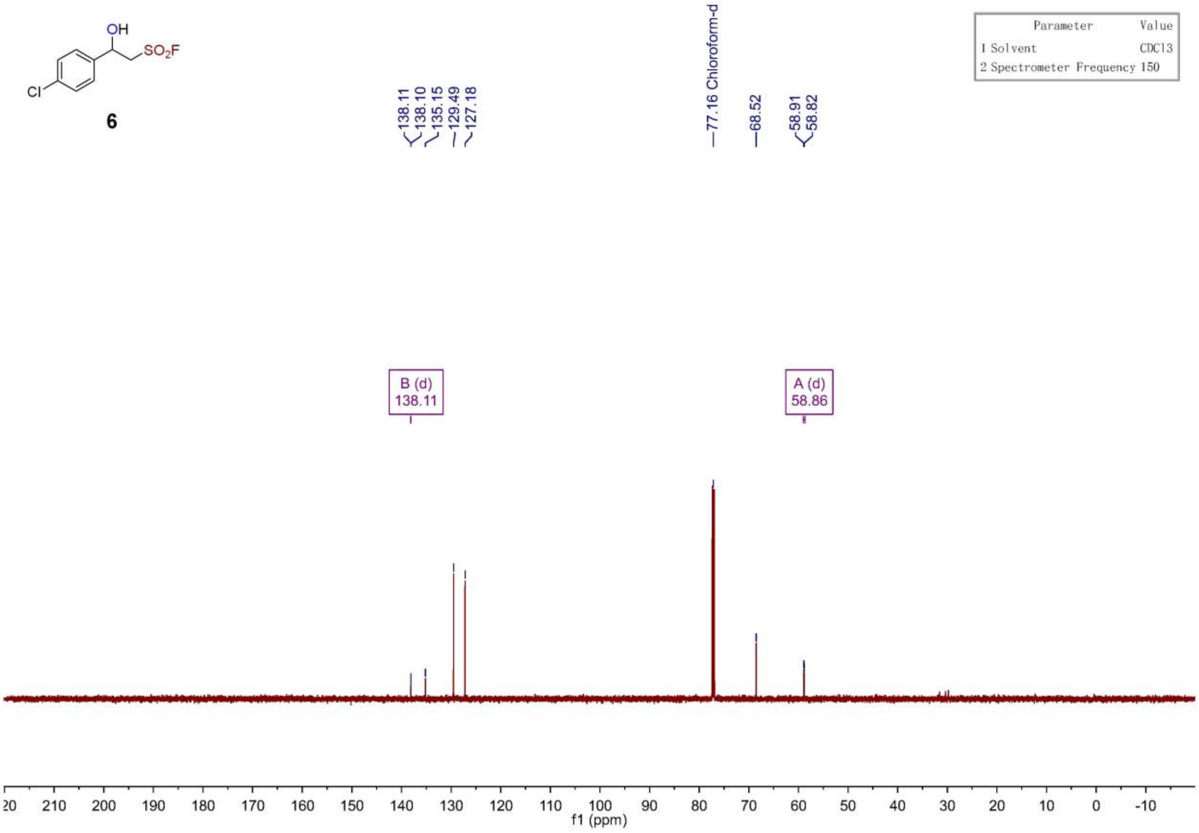

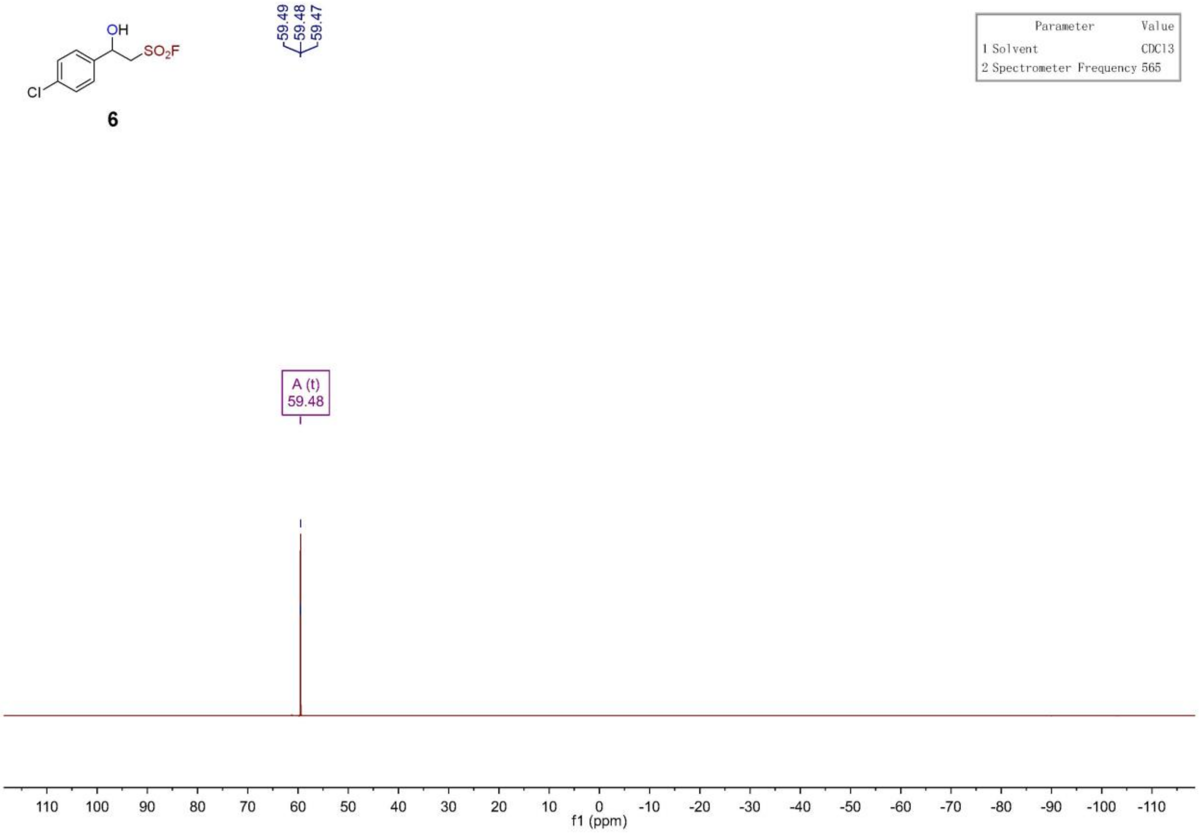

Supplementary Figure 25. ^{1}H, ^{13}C and ^{19}F NMR spectra of **7**.

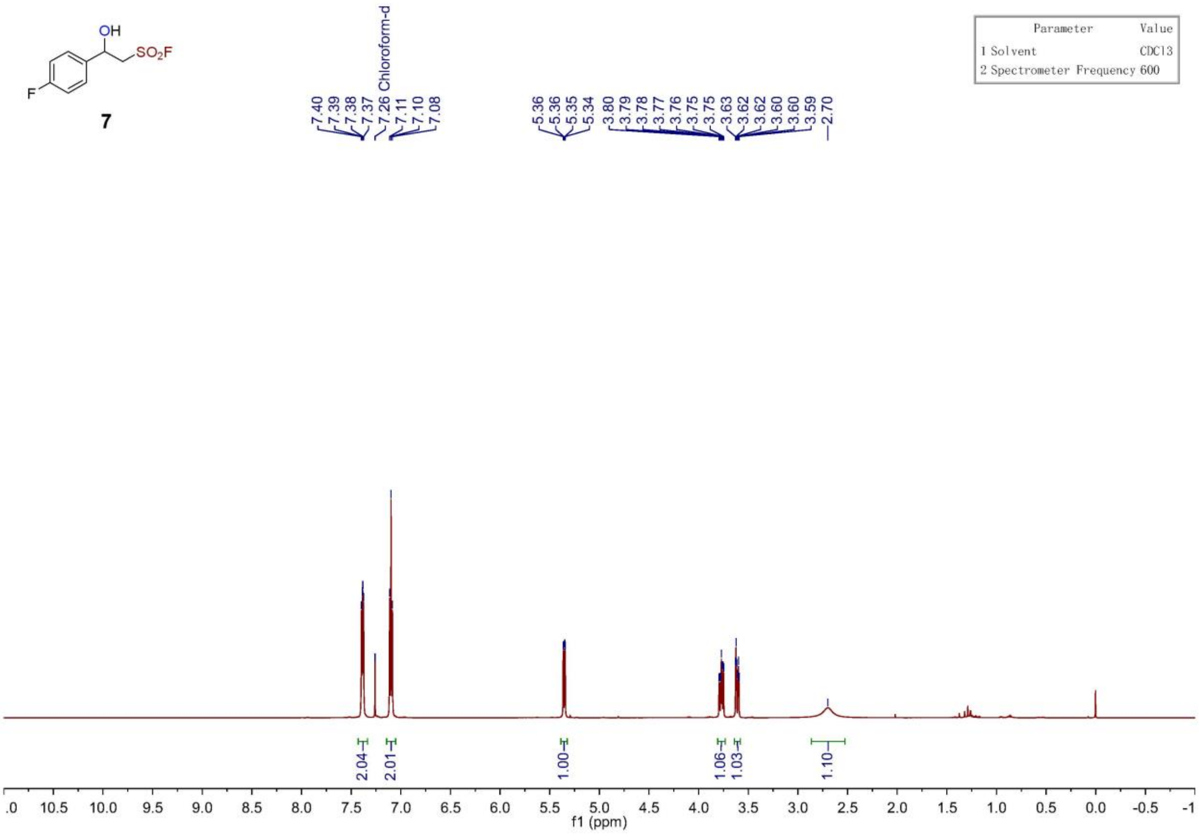

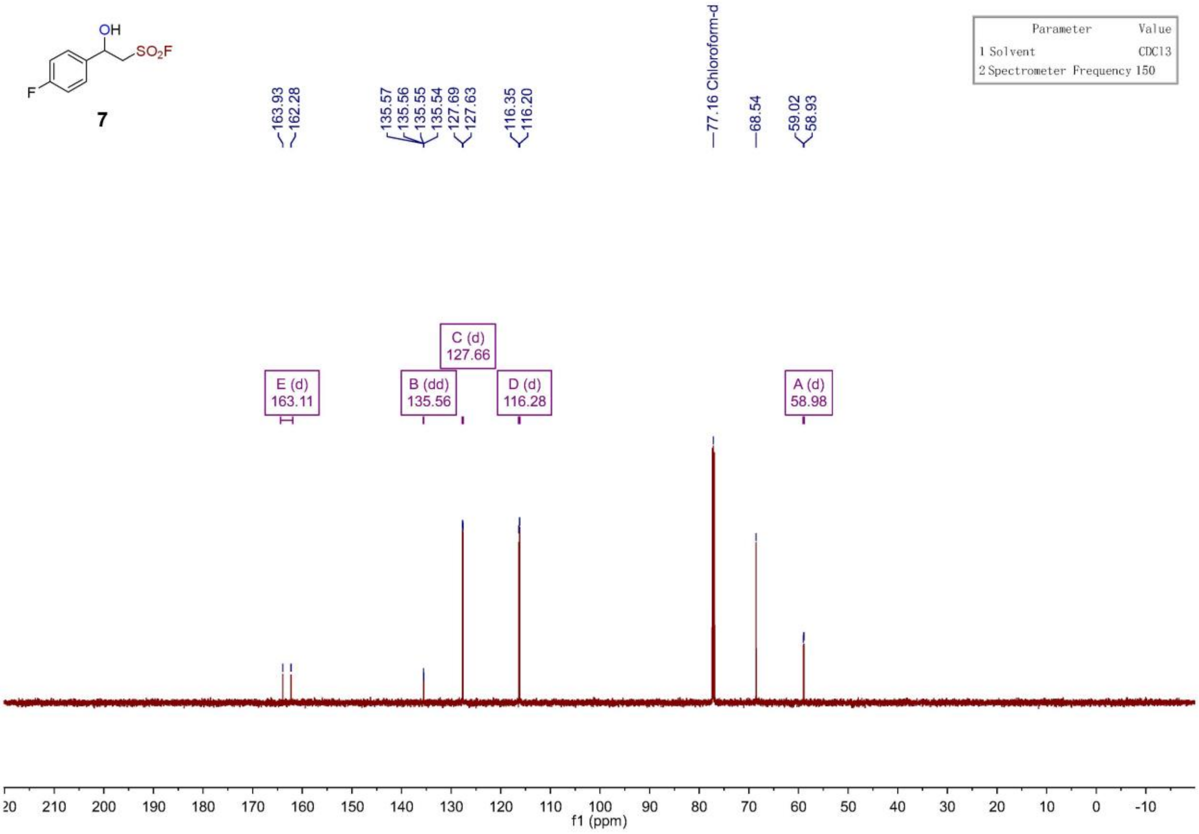

Parameter	Value
1 Solvent	CDCl3
2 Spectrometer Frequency	150

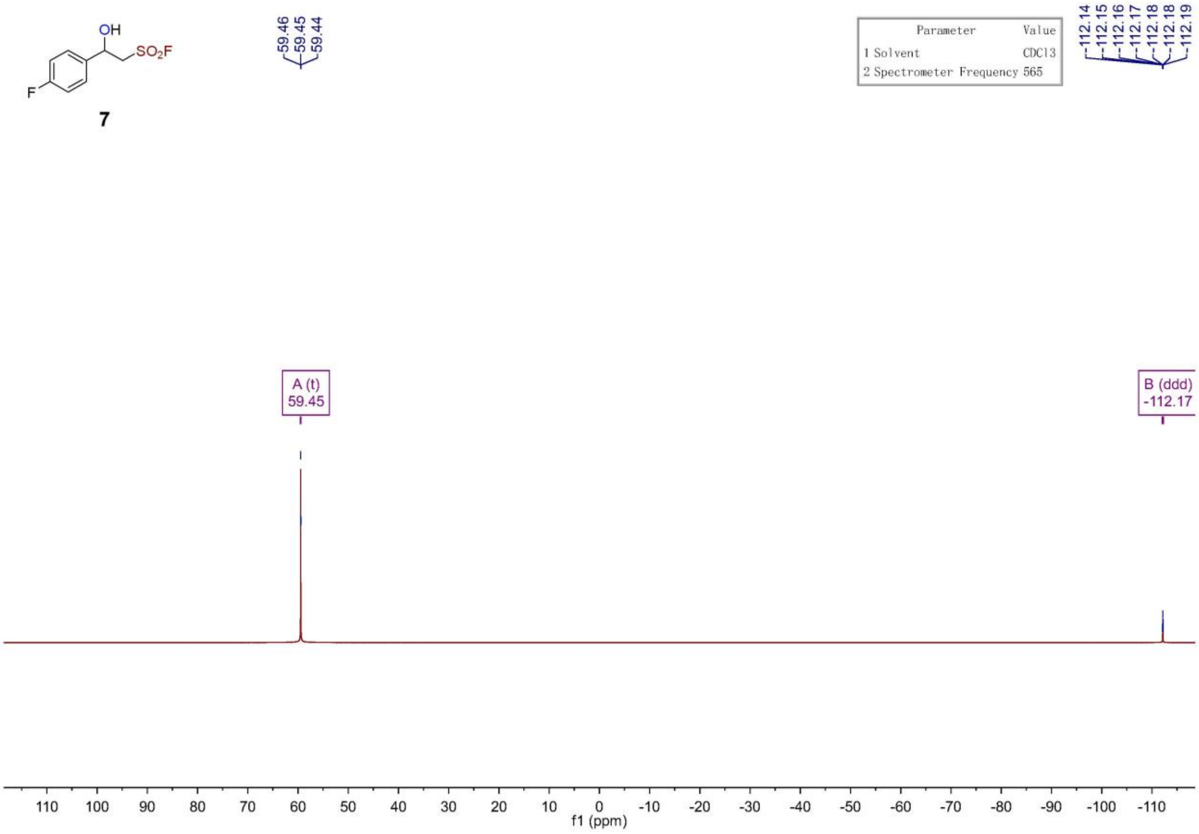

Parameter	Value
1 Solvent	CDCl3
2 Spectrometer Frequency	565

Supplementary Figure 26. ^{1}H, ^{13}C and ^{19}F NMR spectra of **8**.

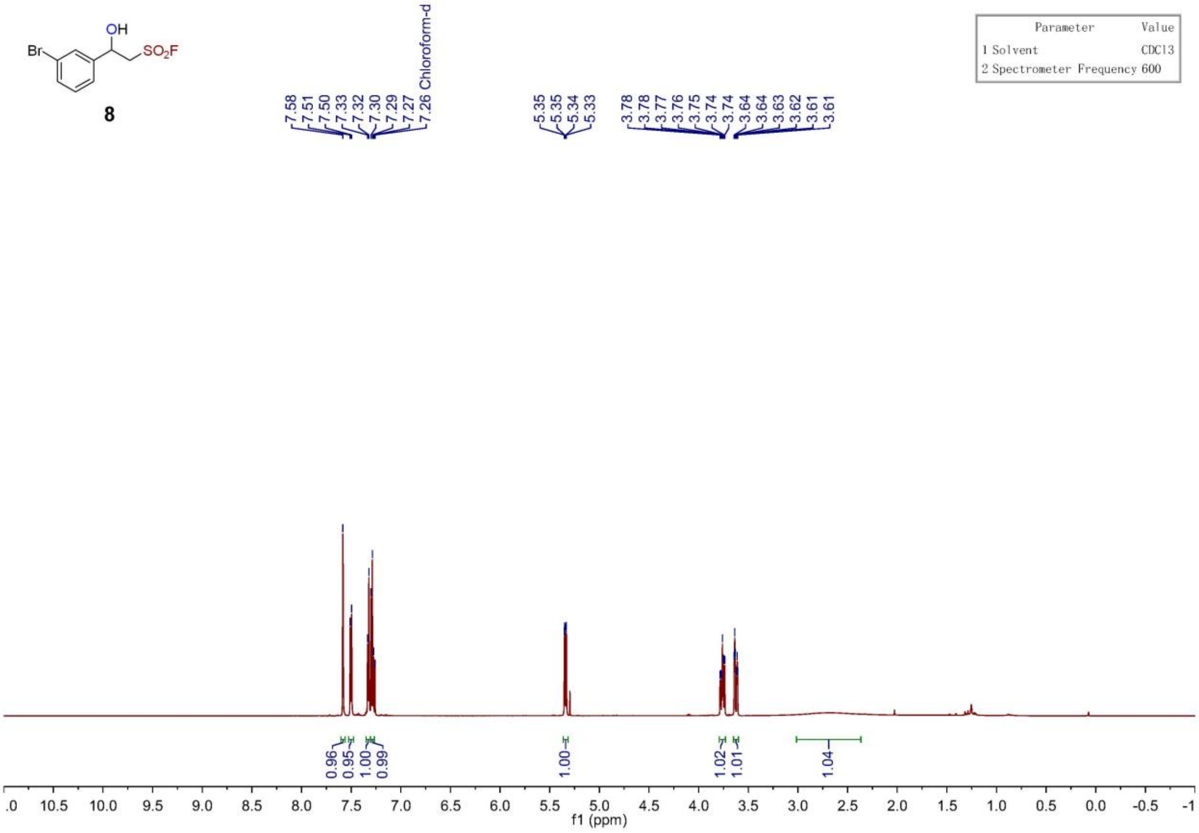

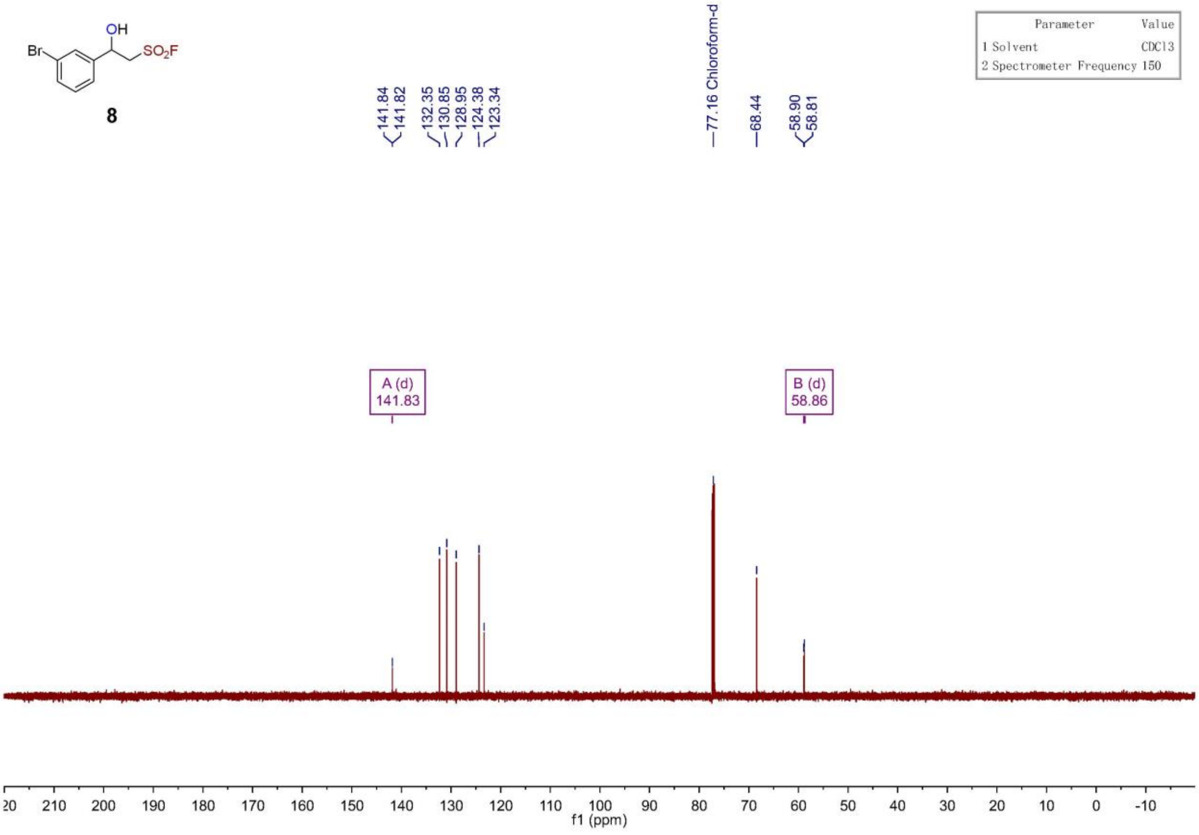

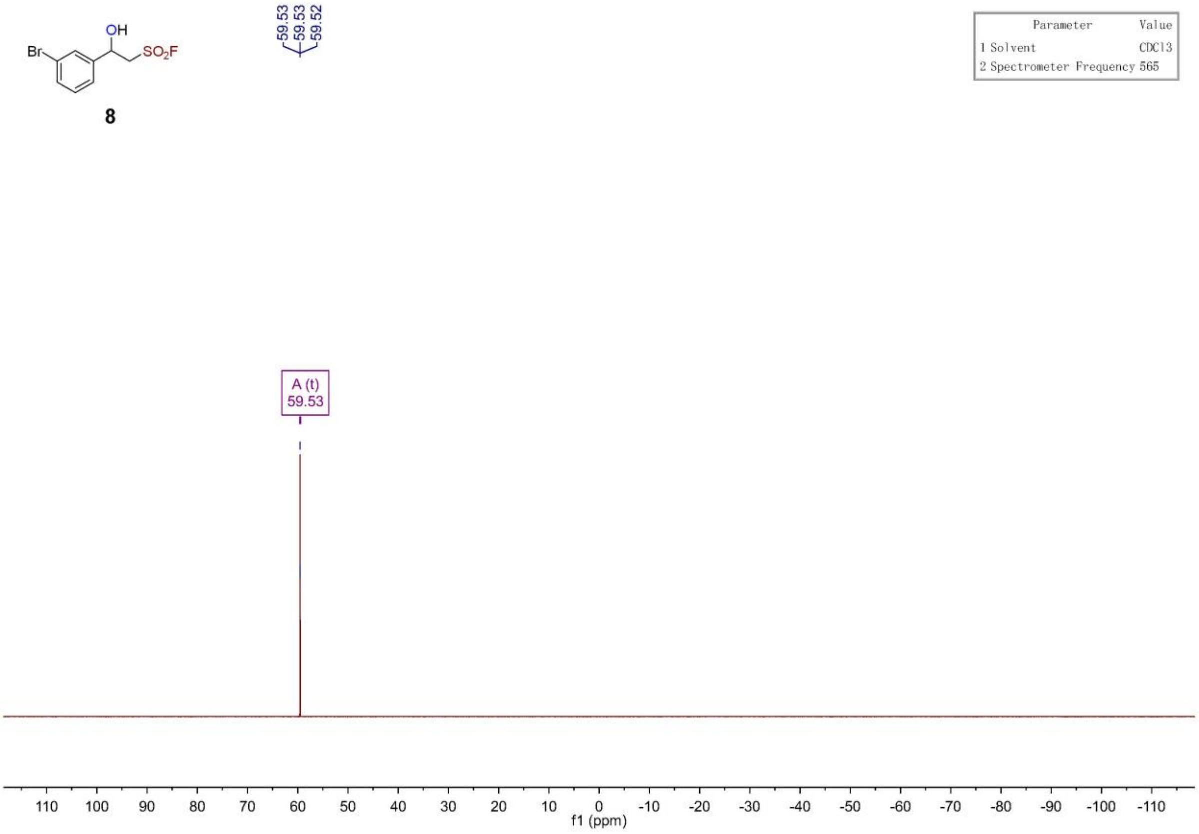

Supplementary Figure 27. ^{1}H, ^{13}C and ^{19}F NMR spectra of **9**.

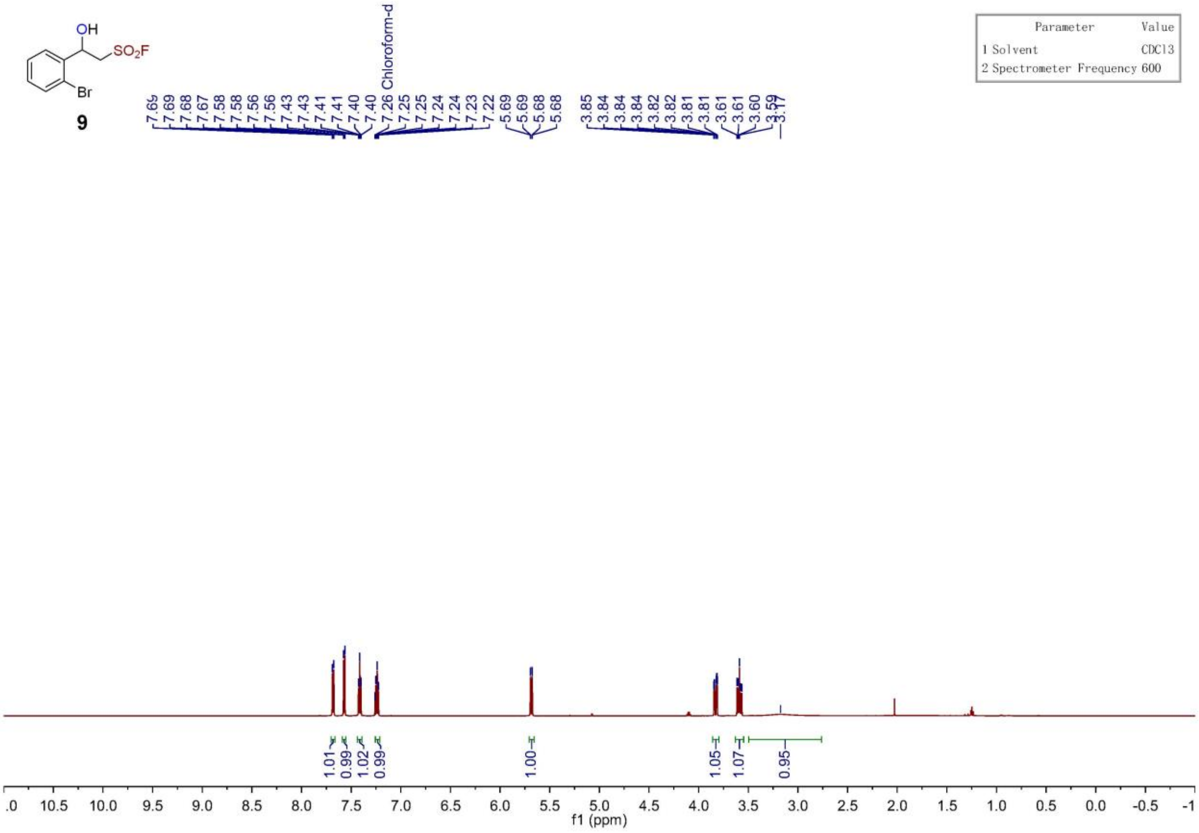

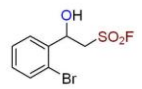

9

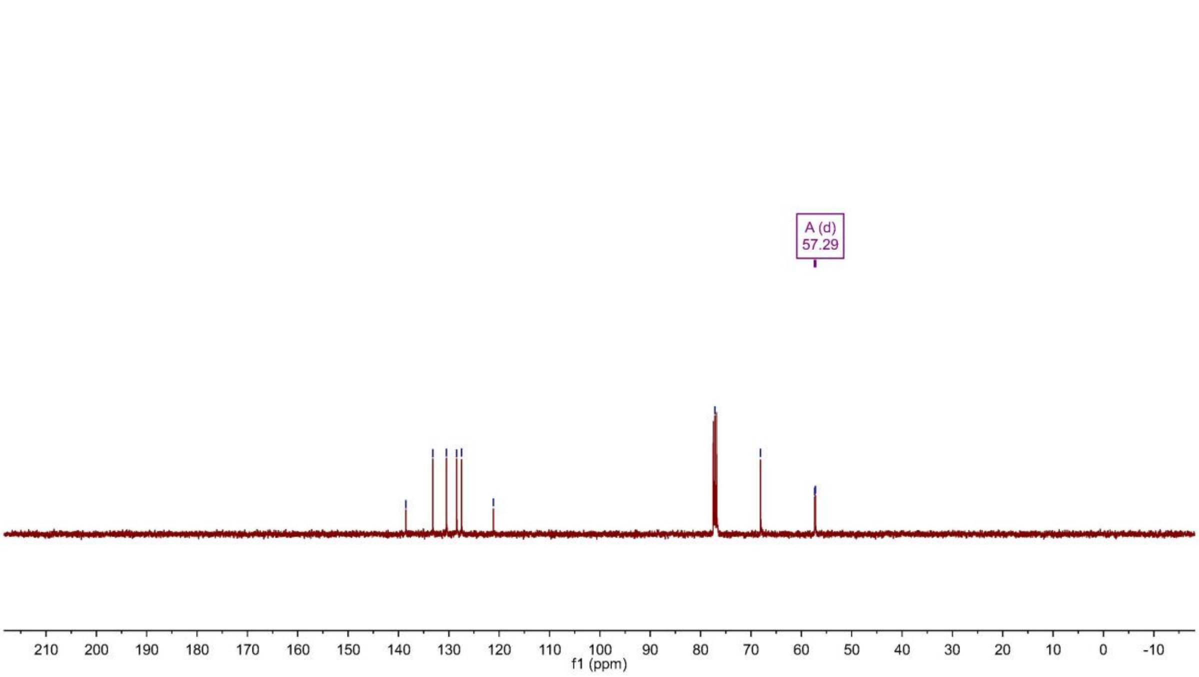

Parameter	Value
1 Solvent	CDCl3
2 Spectrometer Frequency	100

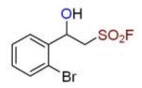

9

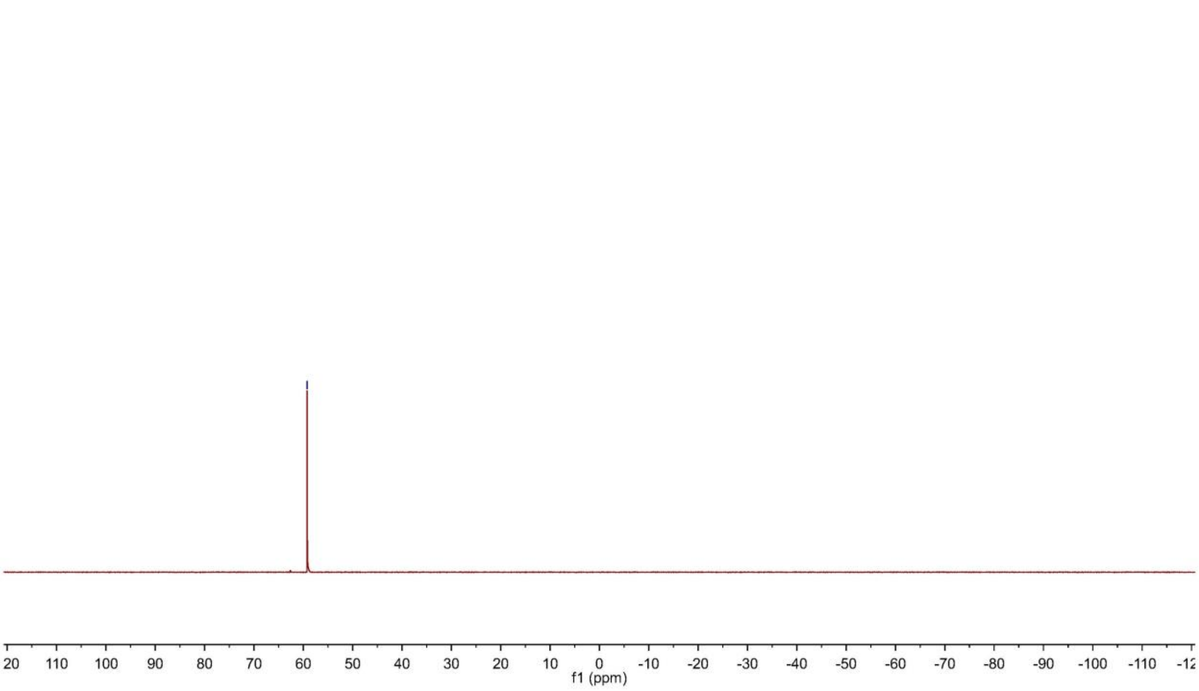

Parameter	Value
1 Solvent	CDCl3
2 Spectrometer Frequency	377

Supplementary Figure 28. ^{1}H, ^{13}C and ^{19}F NMR spectra of **10**.

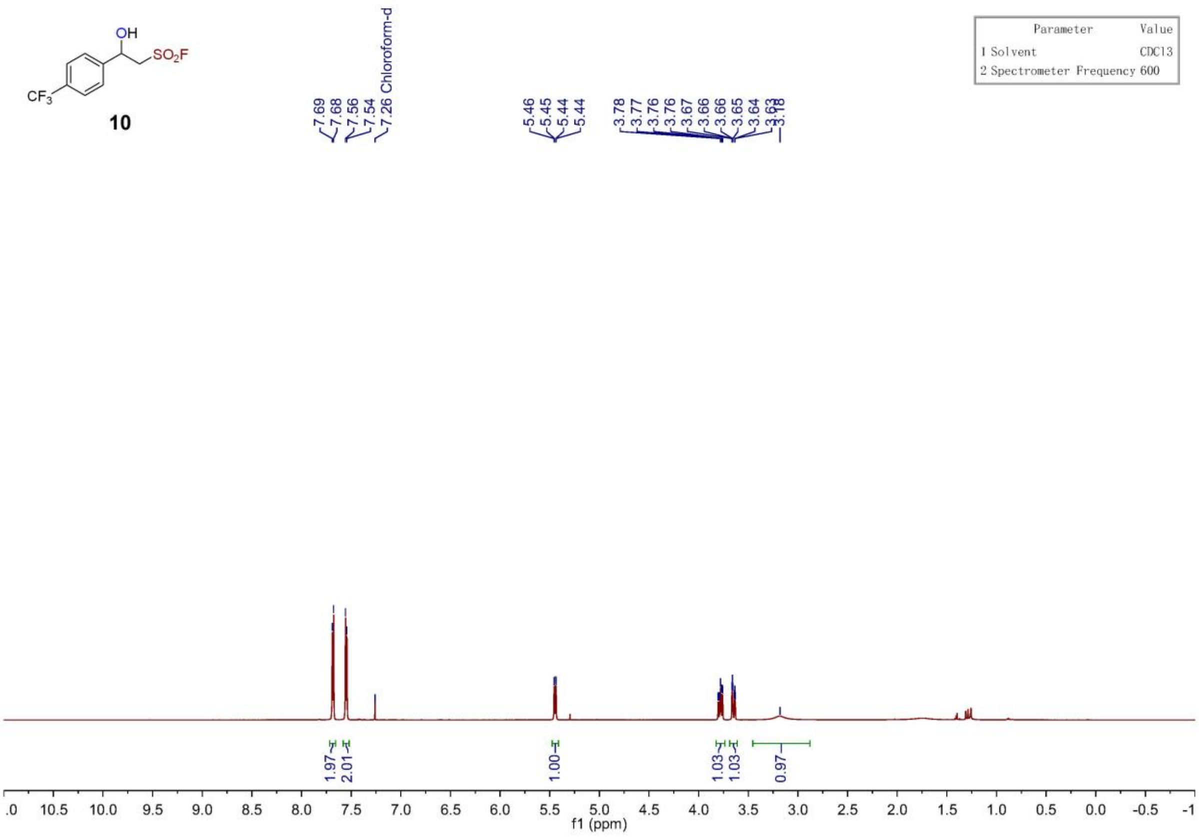

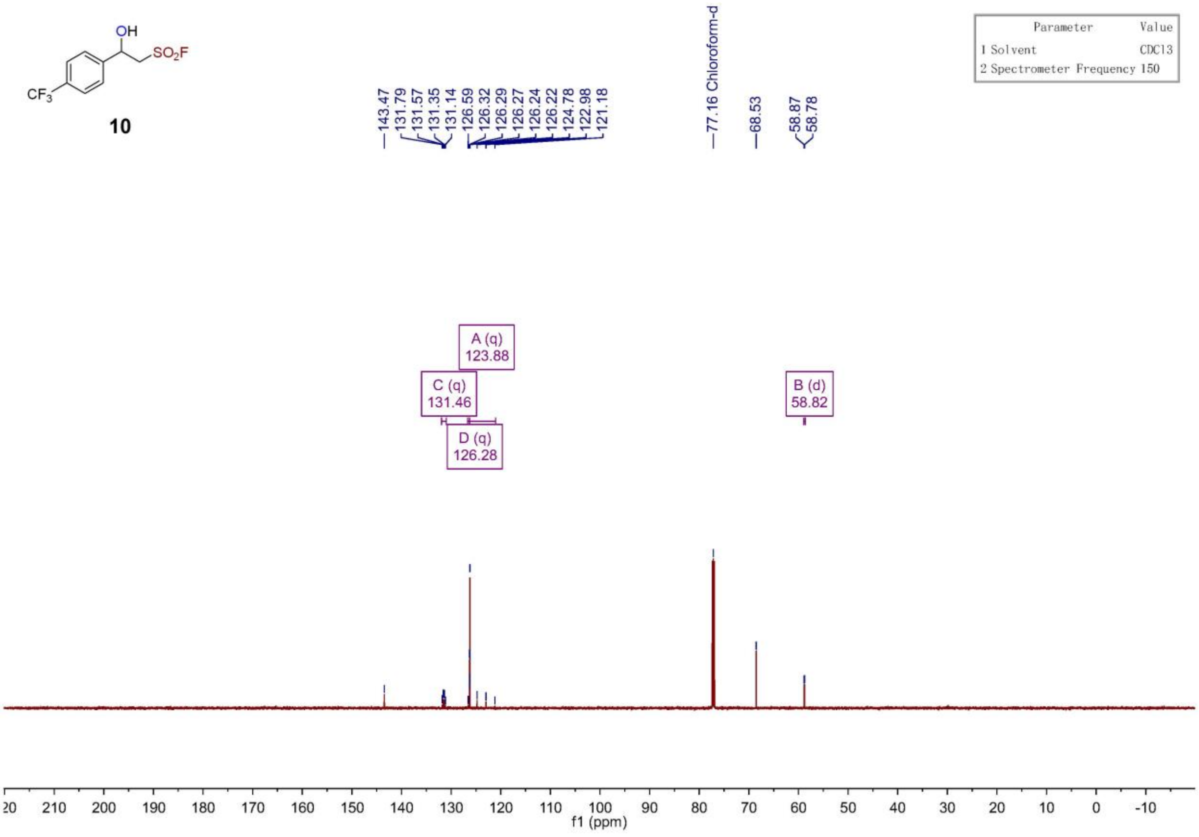

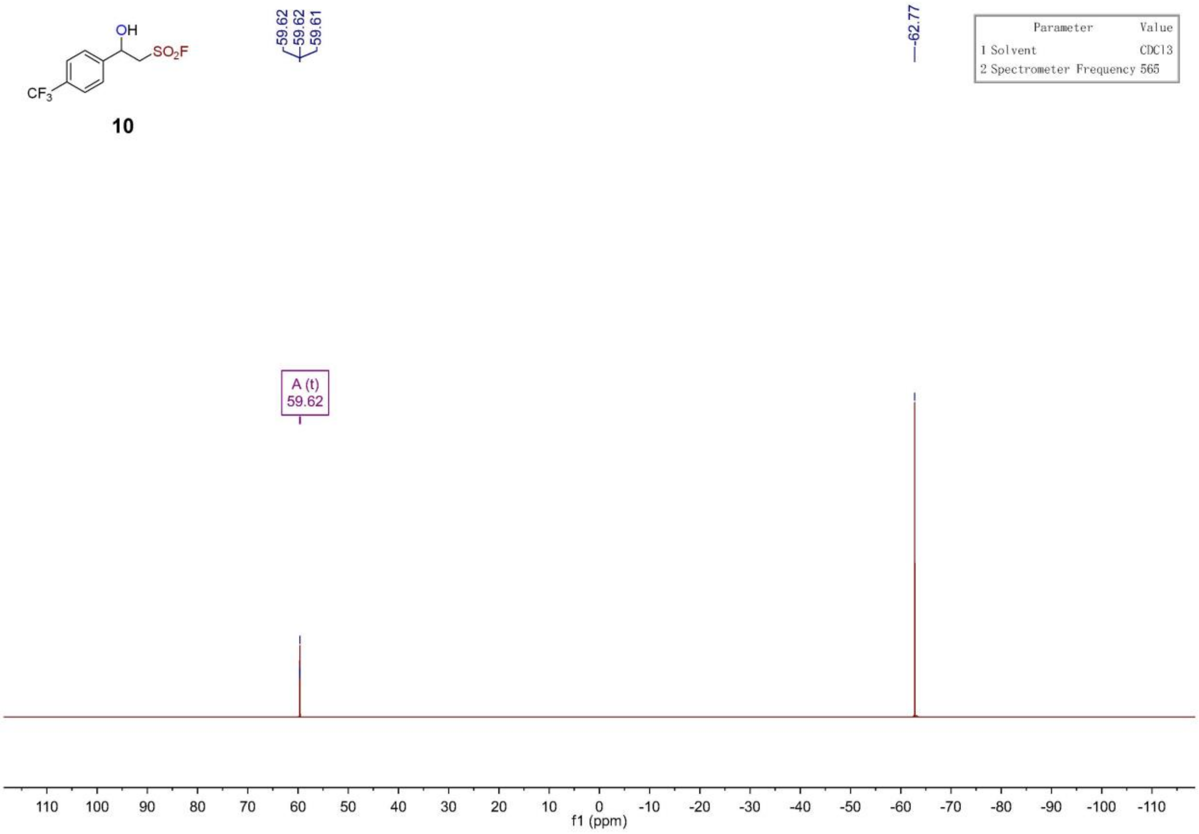

Supplementary Figure 29. ^{1}H, ^{13}C and ^{19}F NMR spectra of **11**.

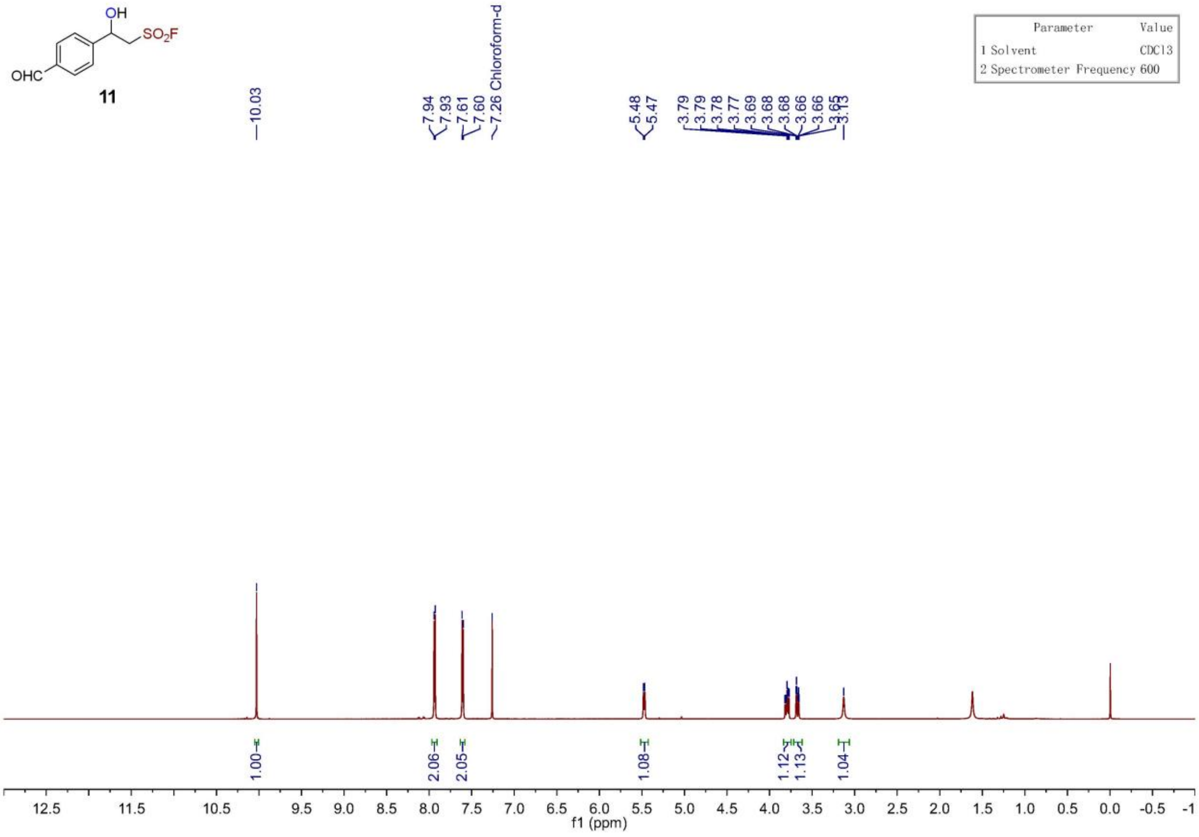

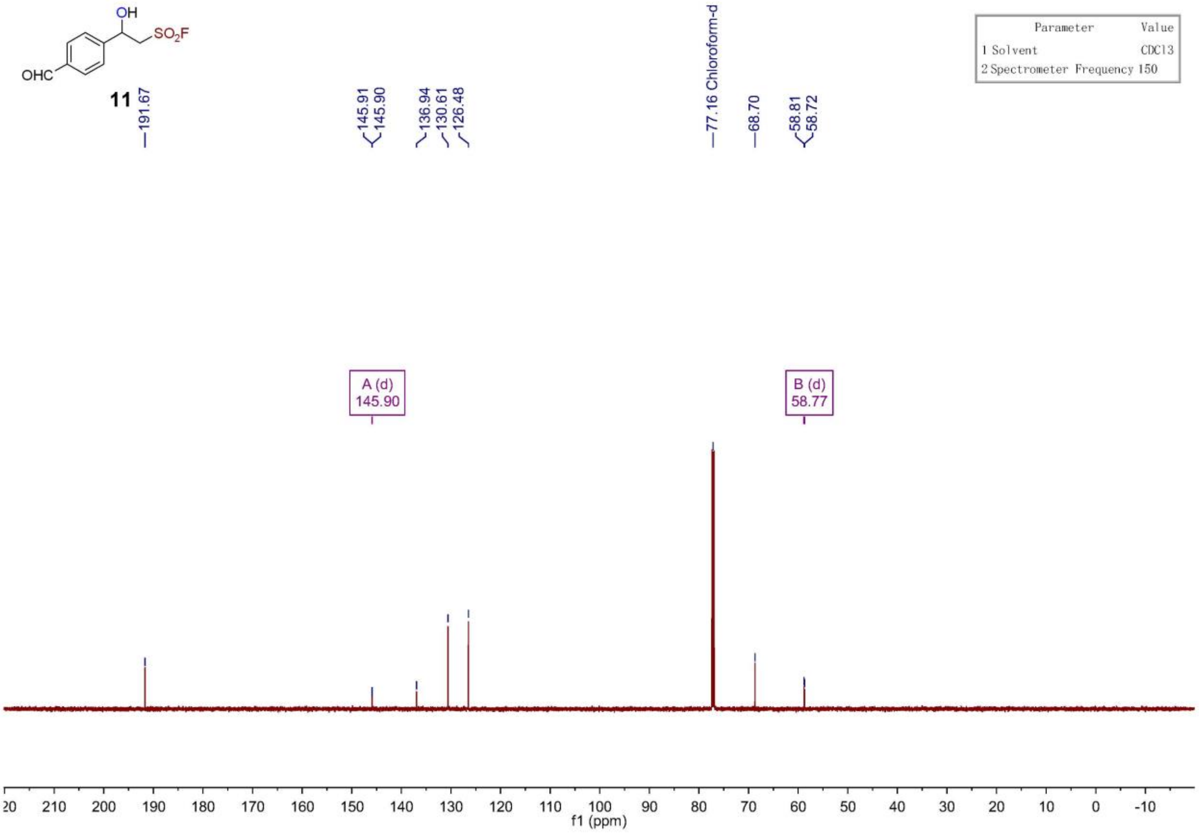

OH
SO2F
OHC
11
191.67
145.91
145.90
136.94
130.61
126.48
77.16 Chloroform-d
68.70
58.81
58.72
Parameter Value
1 Solvent CDCl3
2 Spectrometer Frequency 150
A (d) 145.90
B (d) 58.77
f1 (ppm)

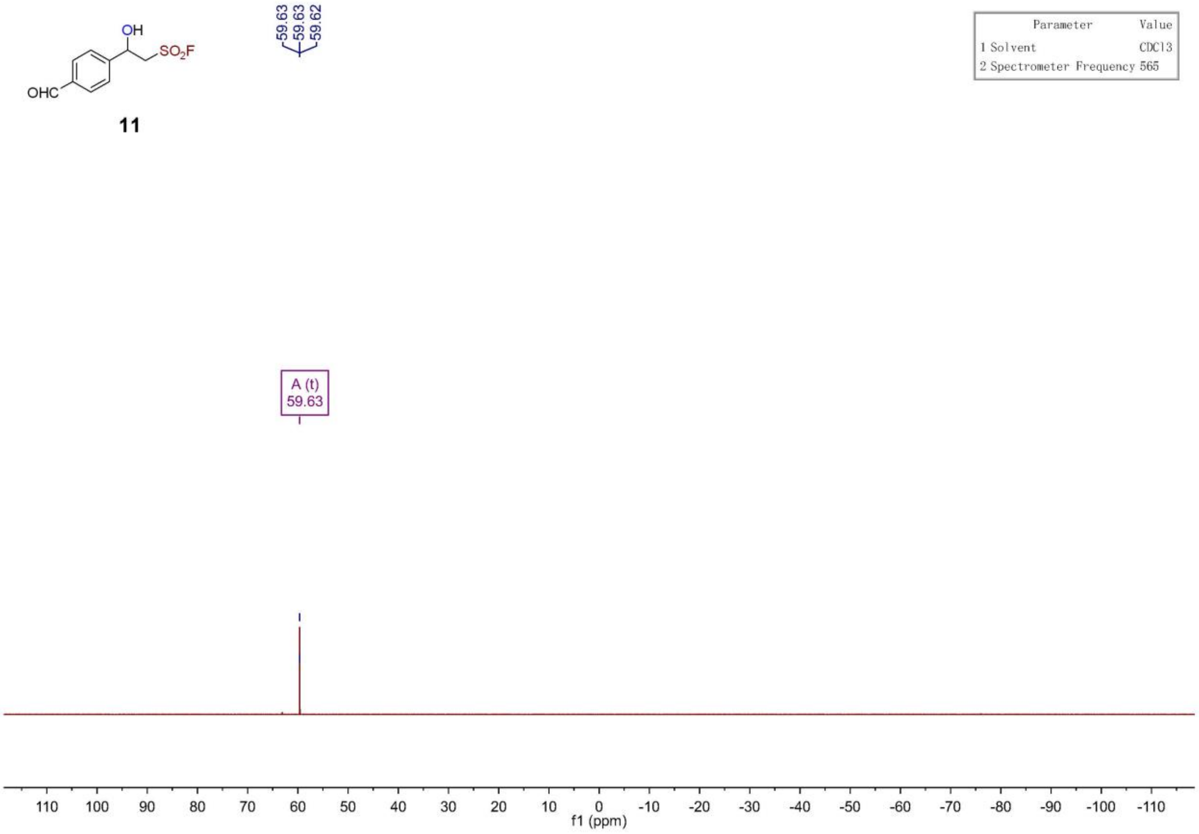

OH
SO2F
OHC
11
59.63
59.63
59.62
Parameter Value
1 Solvent CDCl3
2 Spectrometer Frequency 565
A (t) 59.63
f1 (ppm)

Supplementary Figure 30. ^{1}H, ^{13}C and ^{19}F NMR spectra of **12**.

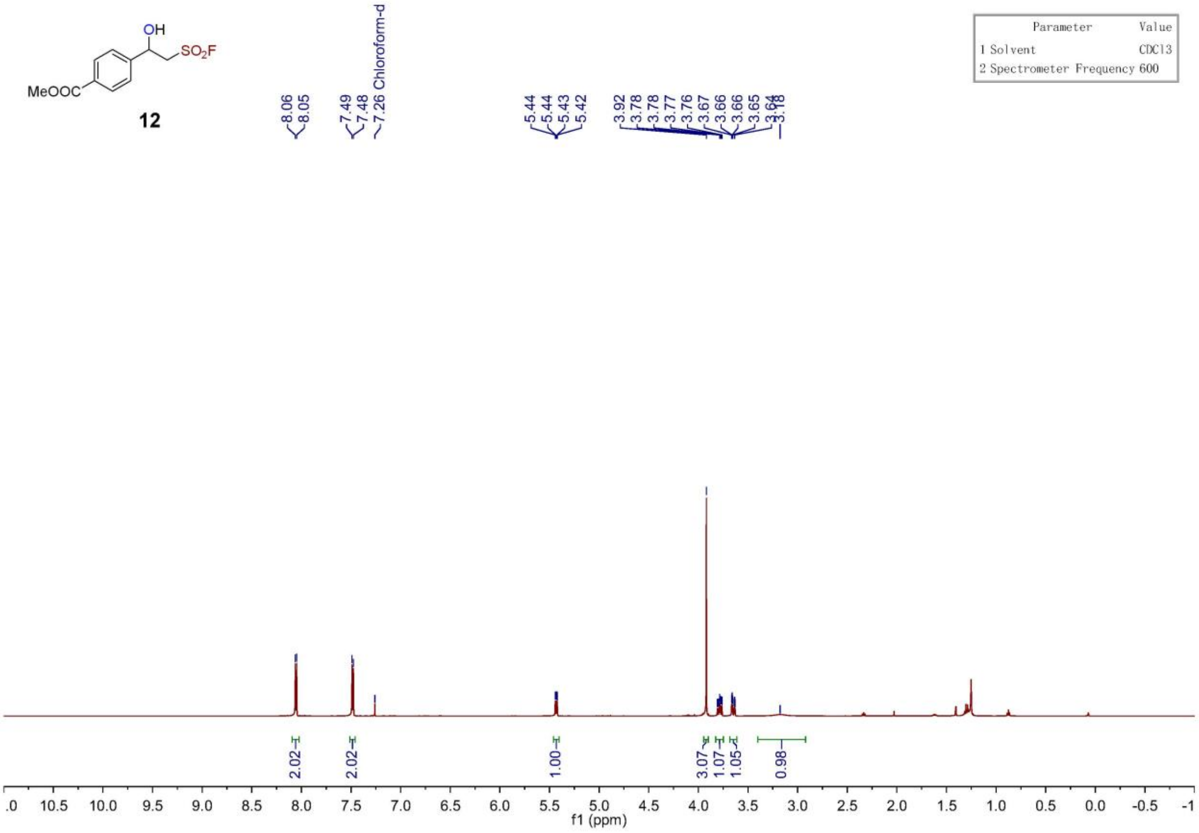

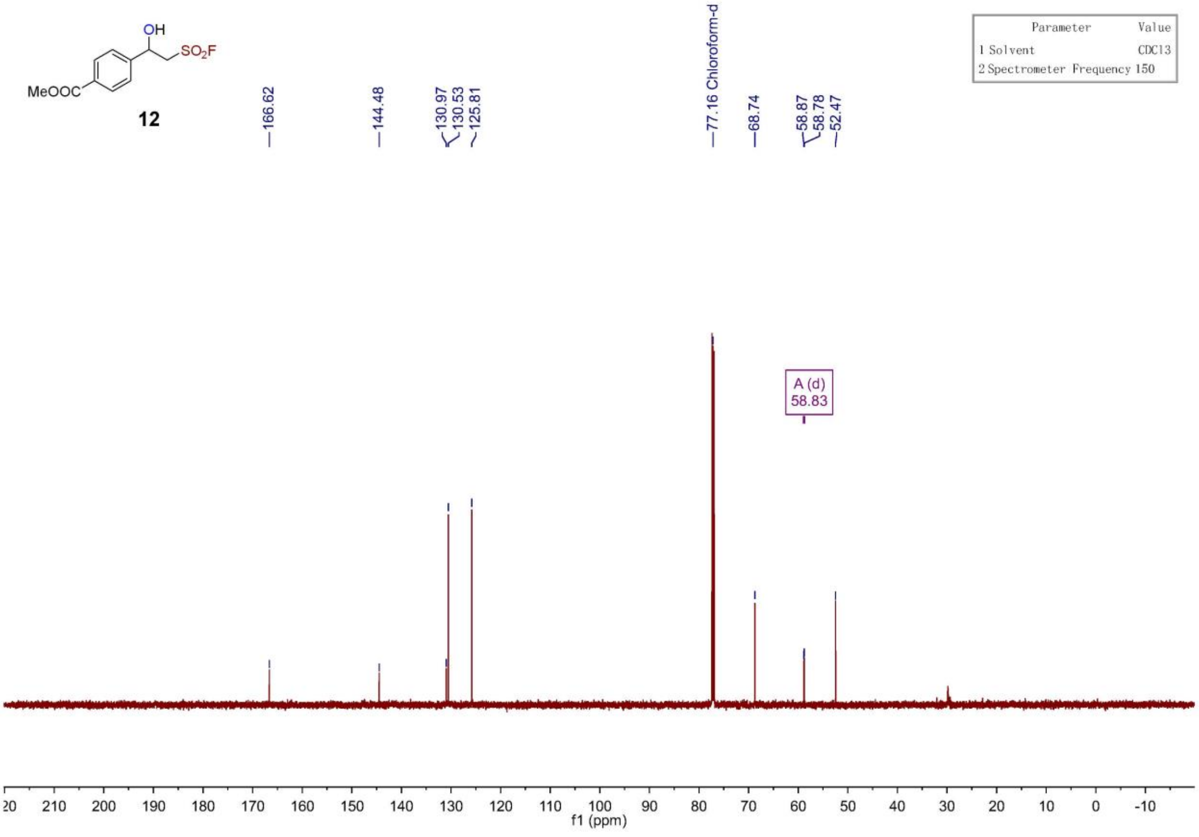

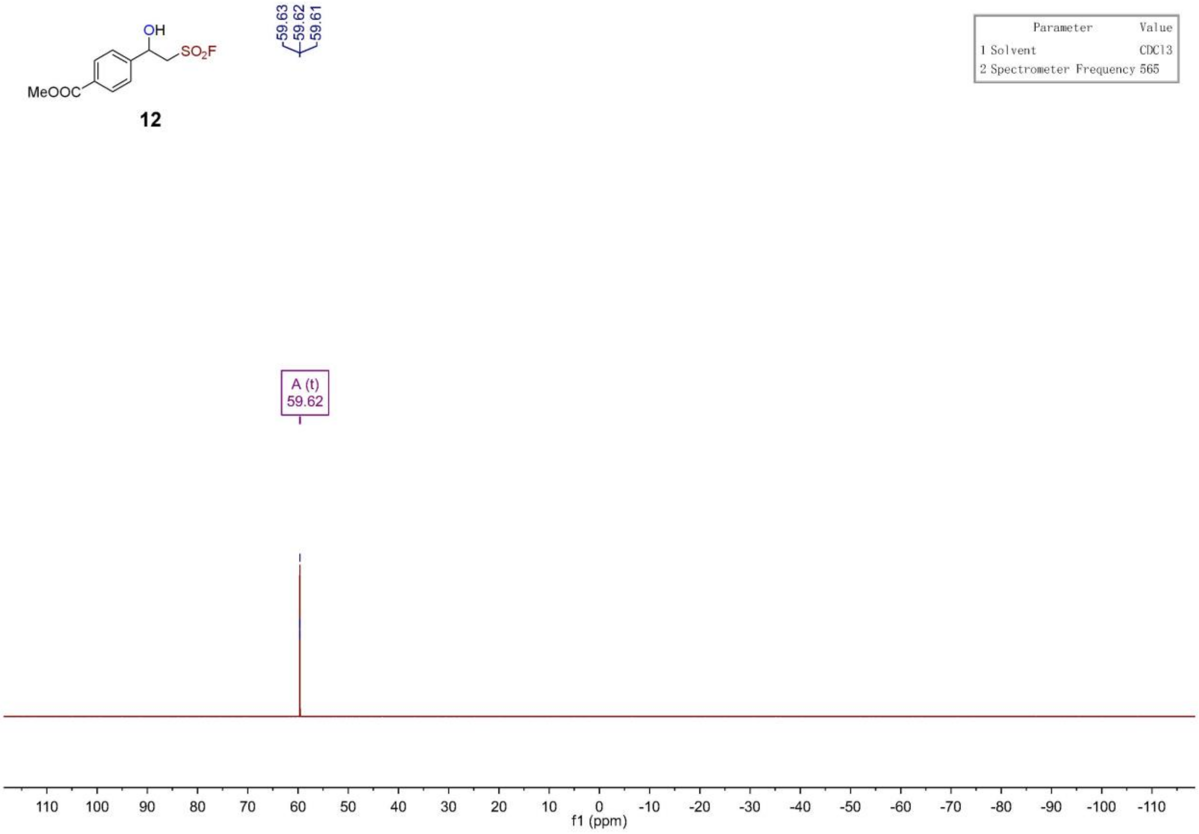

Supplementary Figure 31. ^{1}H, ^{13}C and ^{19}F NMR spectra of **13**.

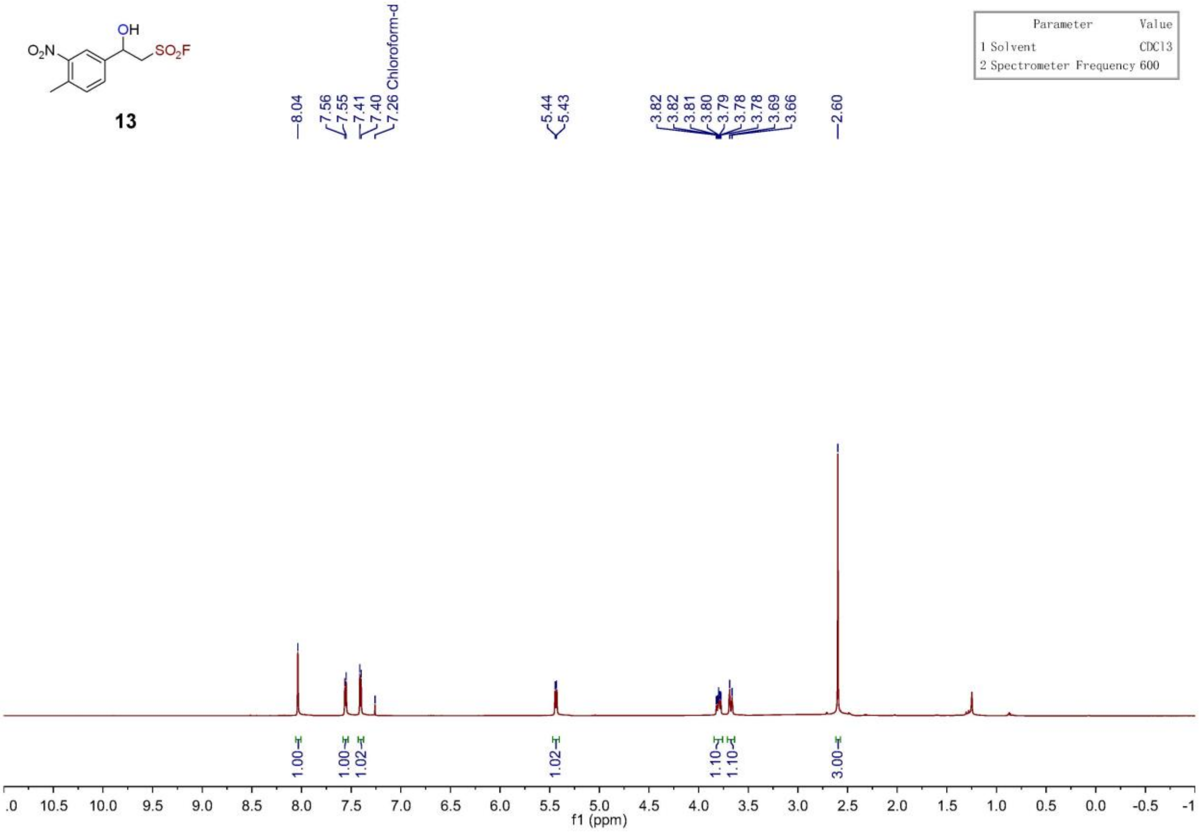

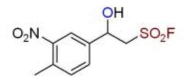
OH
O_2N
SO_2F

13

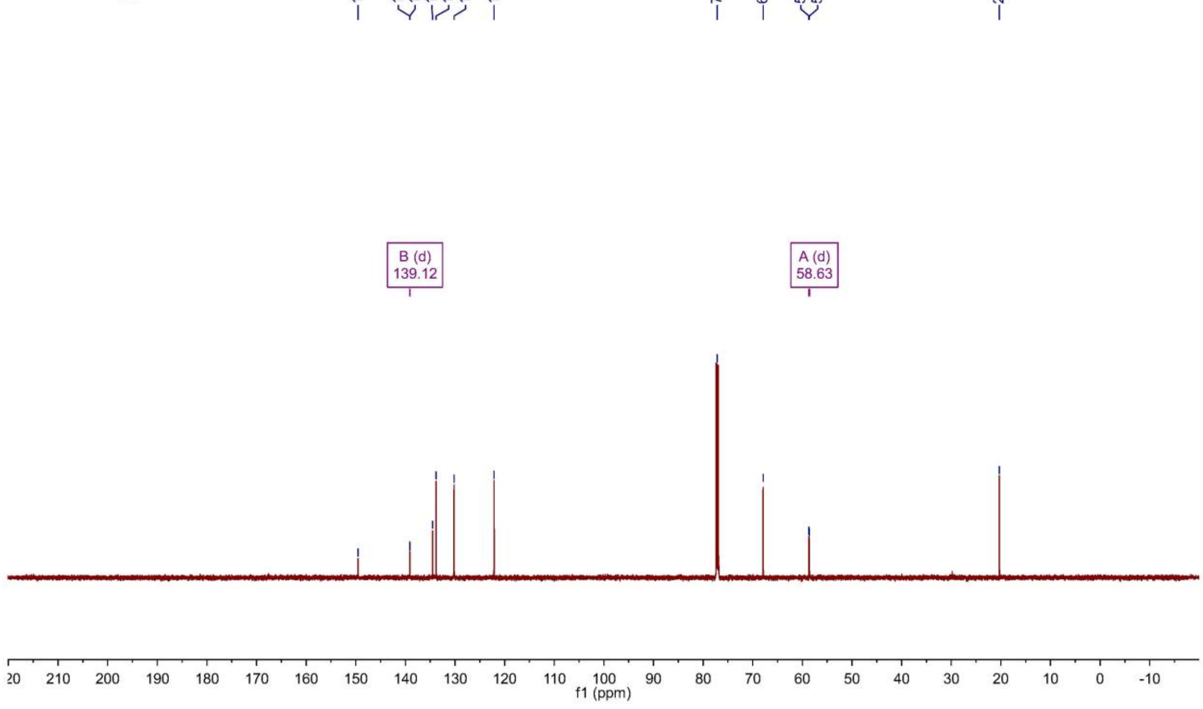
149.54
139.13
139.12
134.52
133.80
130.20
122.13
77.16 Chloroform-d
67.91
58.67
58.59
20.31
Parameter Value
1 Solvent CDCl3
2 Spectrometer Frequency 150
B (d)
139.12
A (d)
58.63
f1 (ppm)

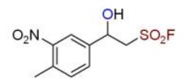
OH
O_2N
SO_2F

13

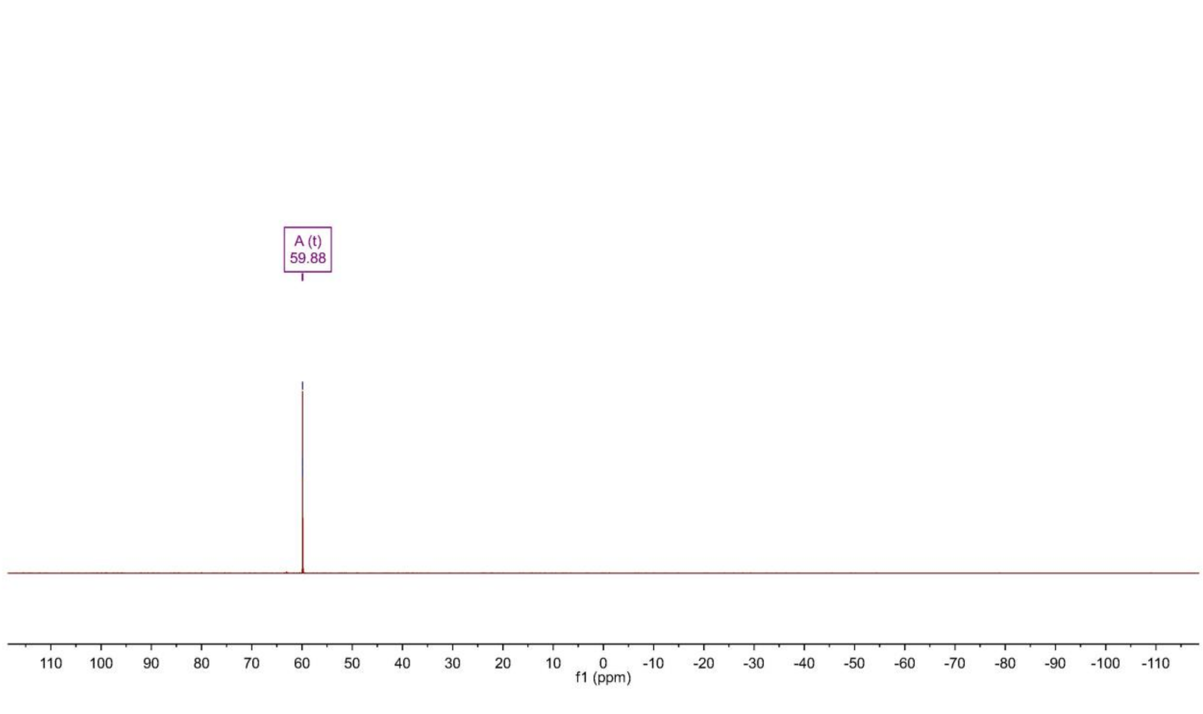
59.88
59.88
59.87
Parameter Value
1 Solvent CDCl3
2 Spectrometer Frequency 565
A (t)
59.88
f1 (ppm)

Supplementary Figure 32. ^{1}H, ^{13}C and ^{19}F NMR spectra of **14**.

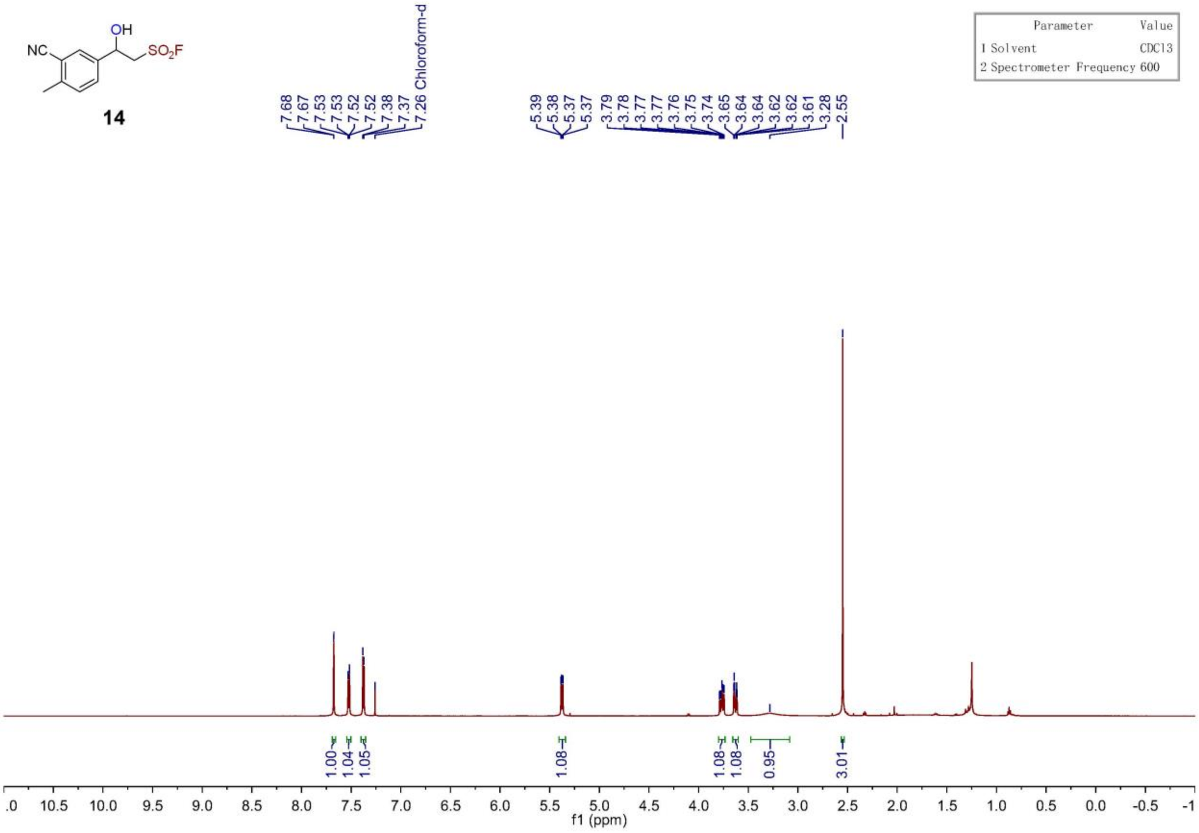

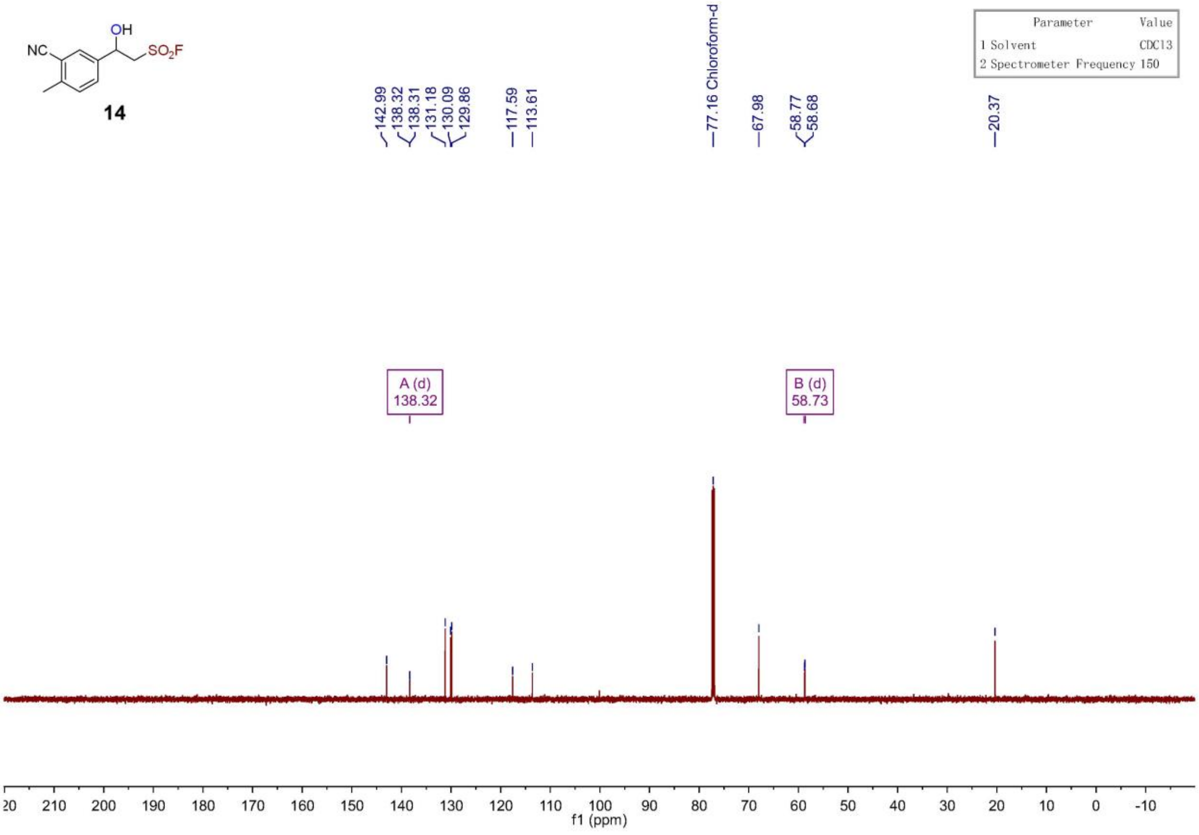

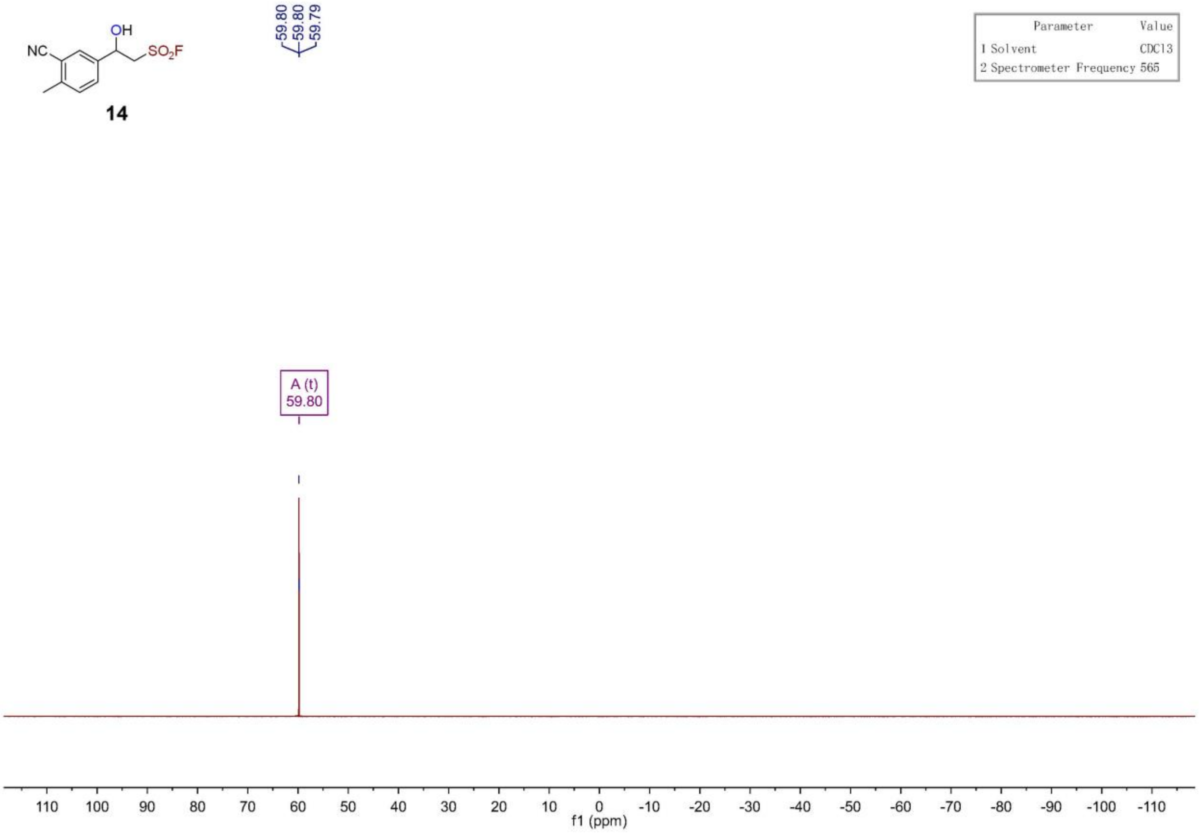

Supplementary Figure 33. ^{1}H, ^{13}C and ^{19}F NMR spectra of **15**.

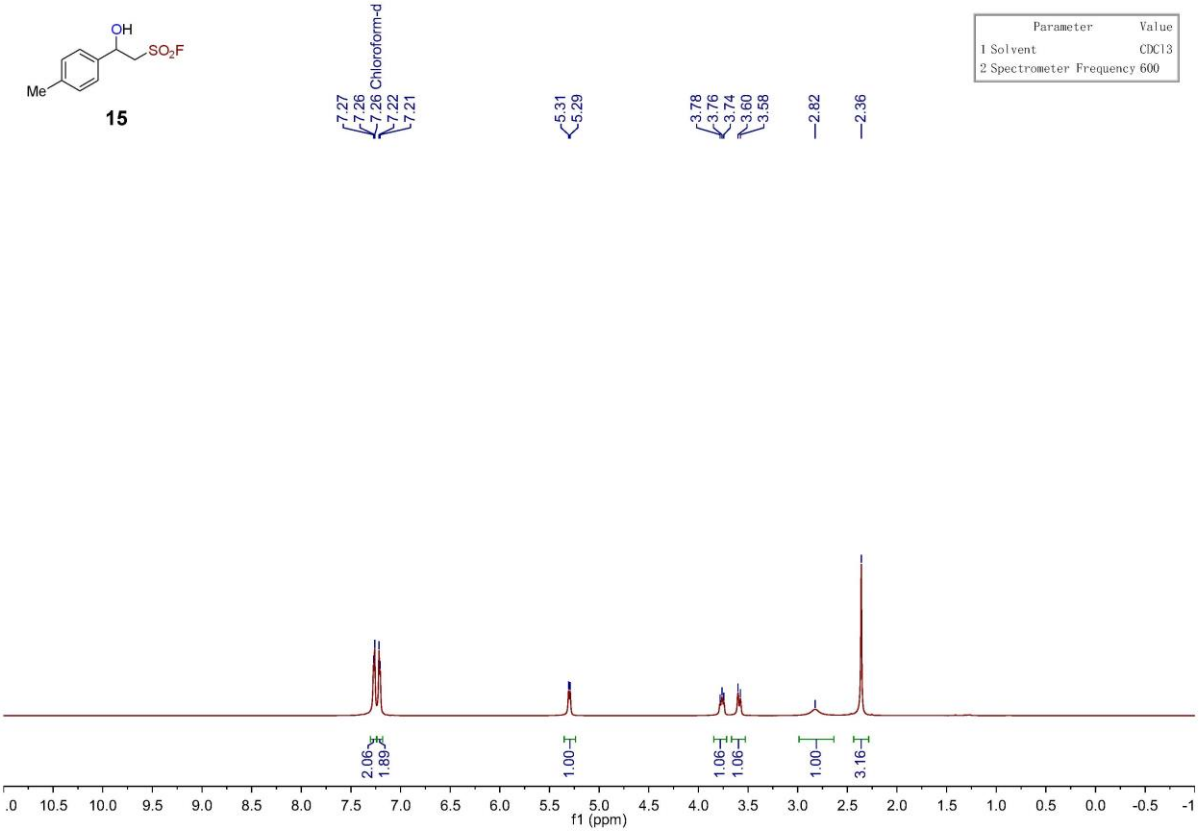

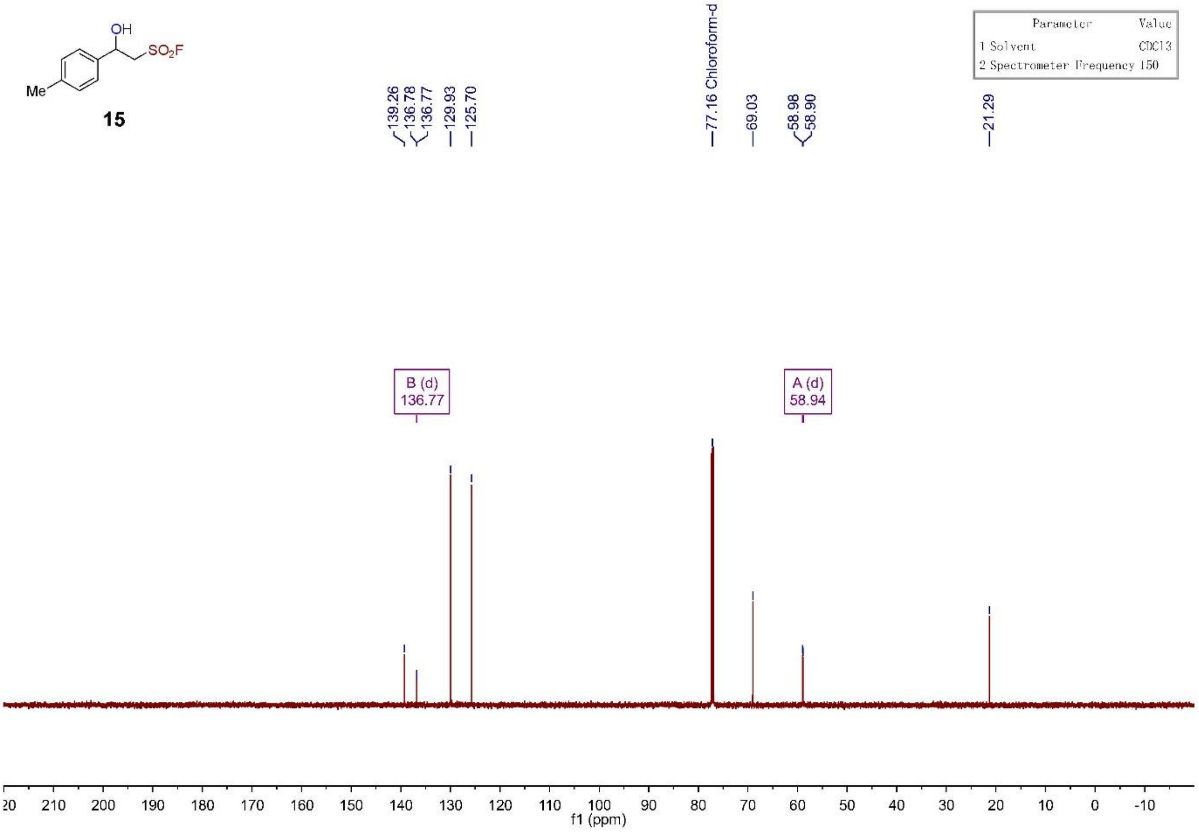
OH
SO2F
Me
15
139.26
136.78
136.77
129.93
125.70
77.16 Chloroform-d
69.03
58.98
58.90
21.29
Parameter Value
1 Solvent CDCl3
2 Spectrometer Frequency 150
B (d)
136.77
A (d)
58.94
f1 (ppm)

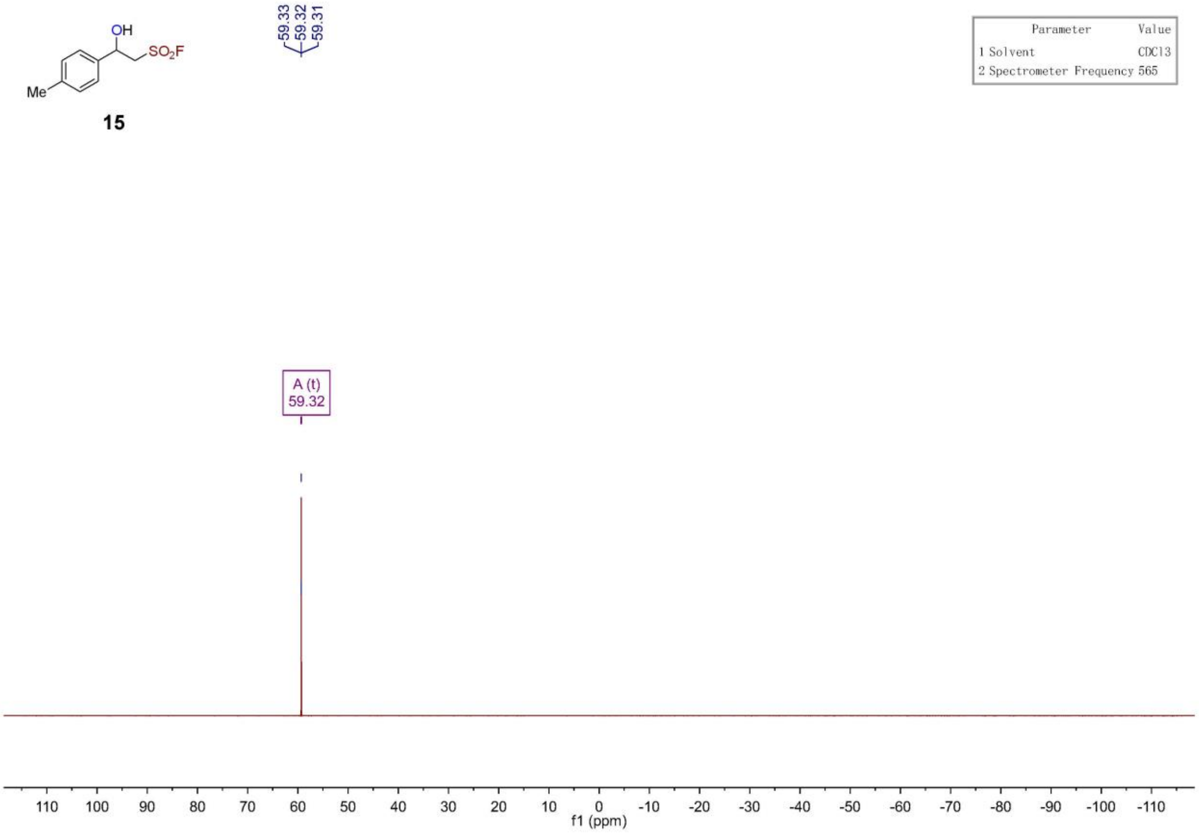
OH
SO2F
Me
15
59.33
59.32
59.31
Parameter Value
1 Solvent CDCl3
2 Spectrometer Frequency 565
A (t)
59.32
f1 (ppm)

Supplementary Figure 34. ^{1}H, ^{13}C and ^{19}F NMR spectra of **16**.

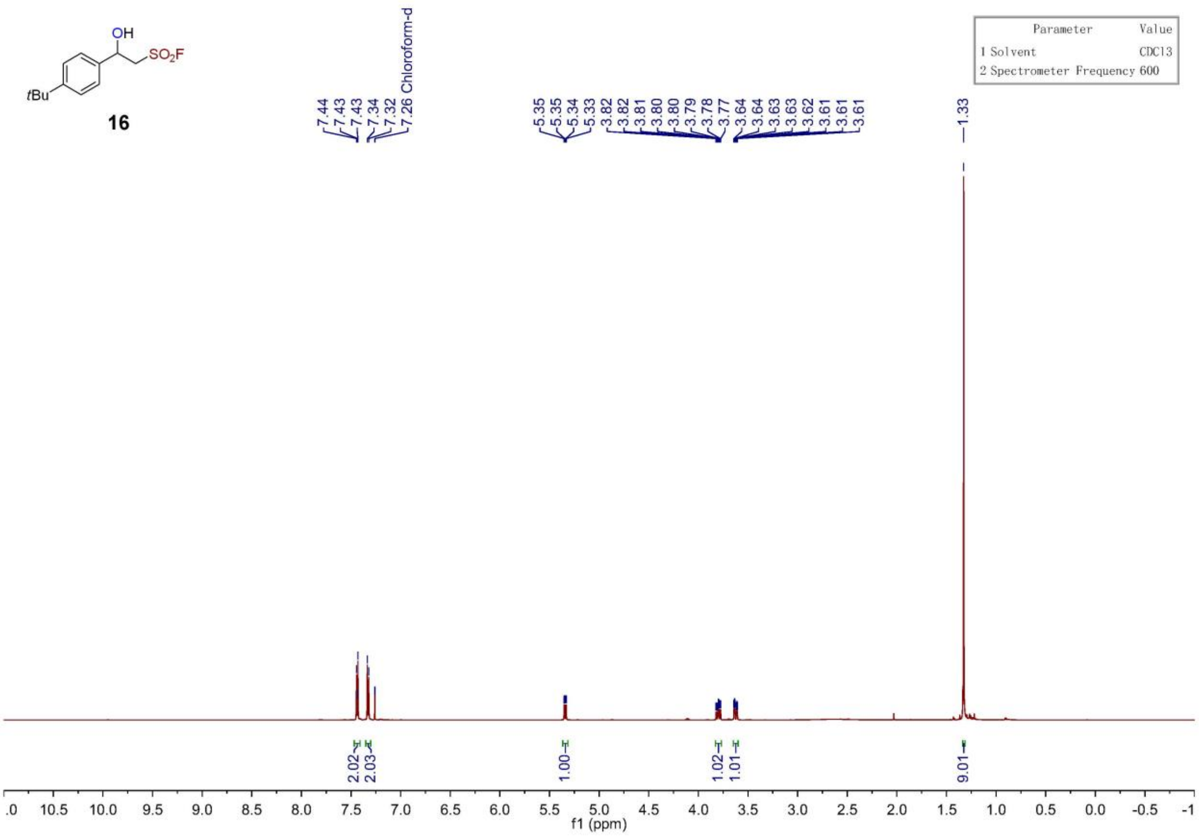

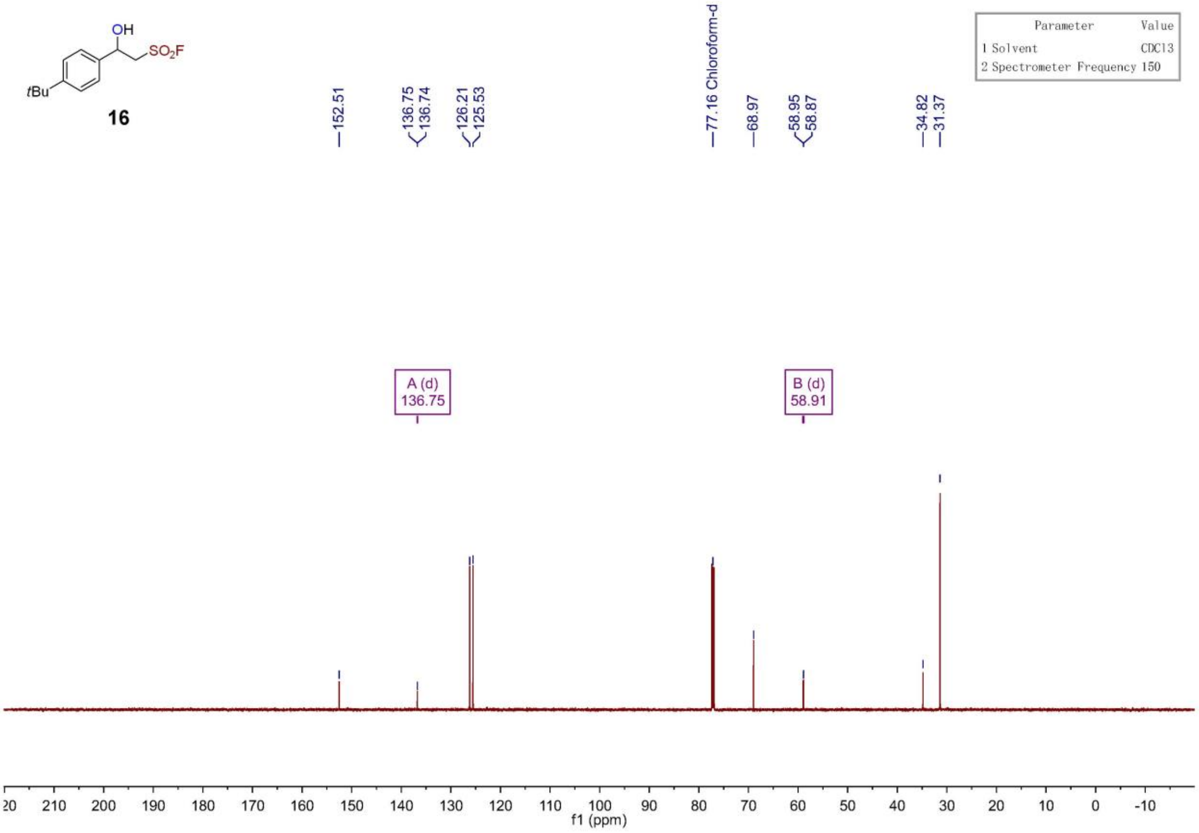

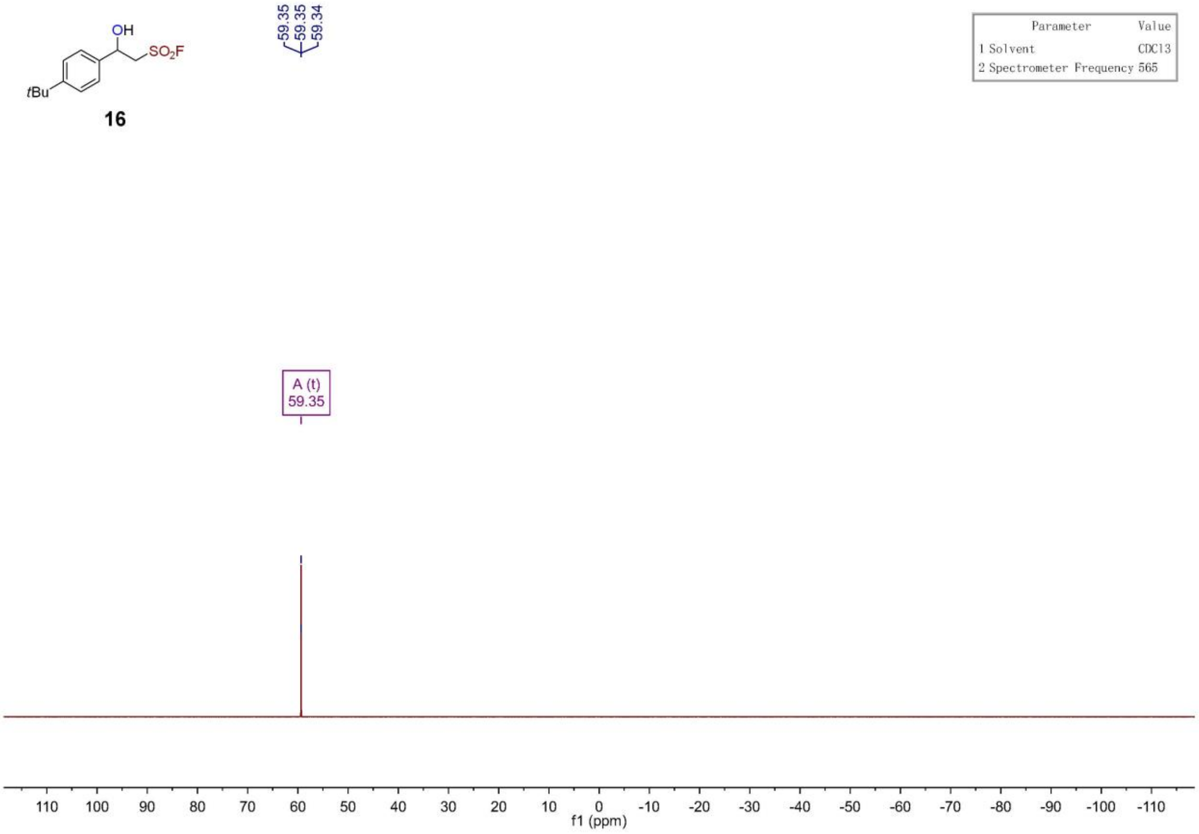

Supplementary Figure 35. ^{1}H, ^{13}C and ^{19}F NMR spectra of **17**.

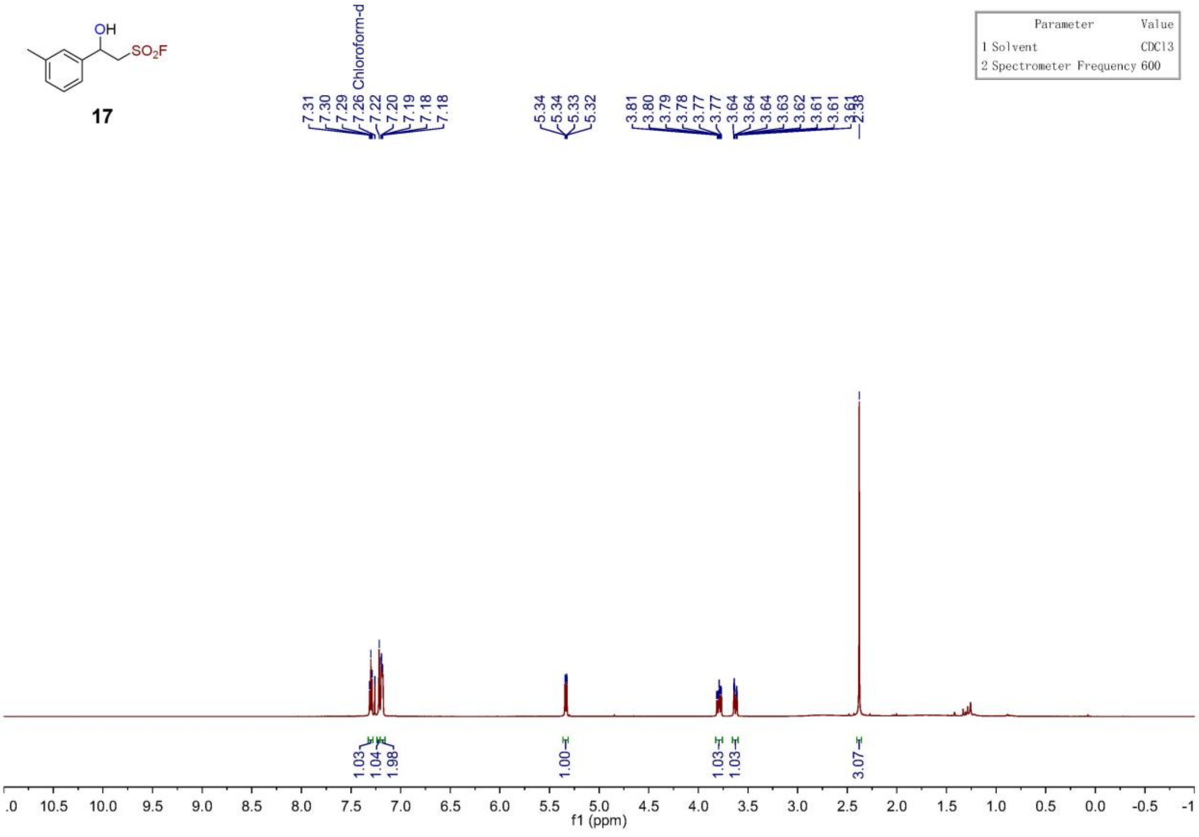

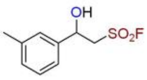

17

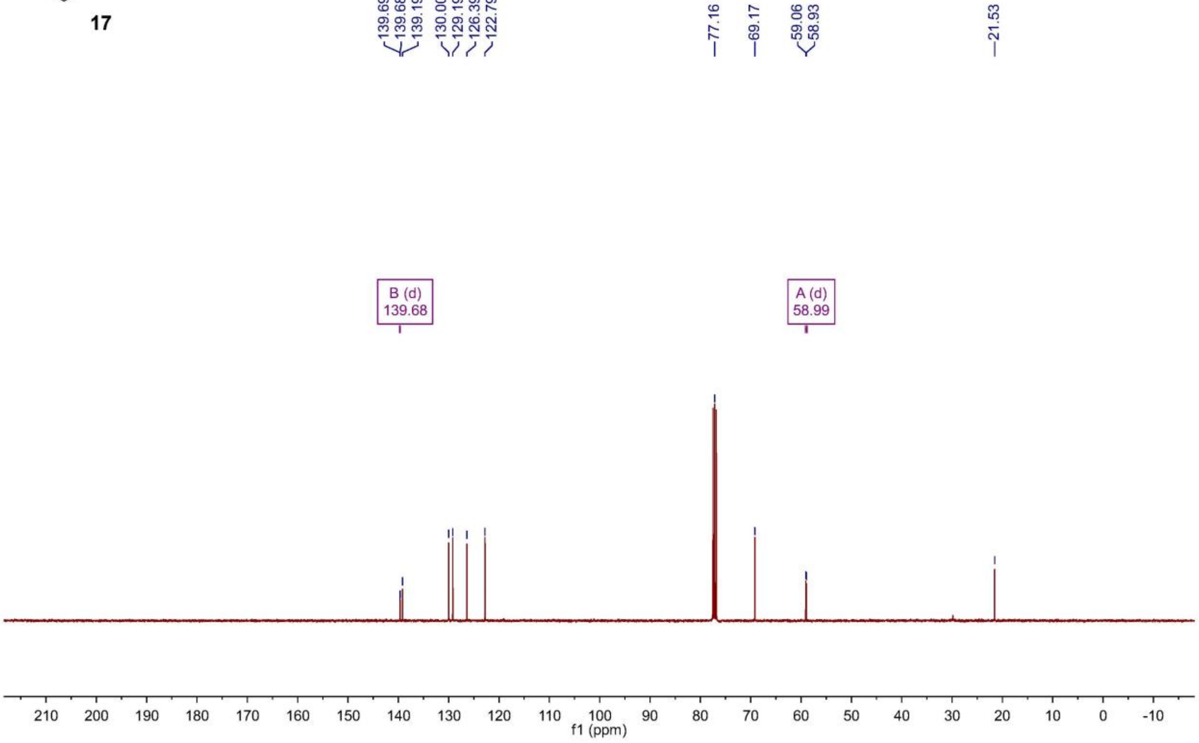

Parameter	Value
1 Solvent	CDCl3
2 Spectrometer Frequency	100

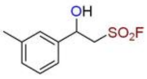

17

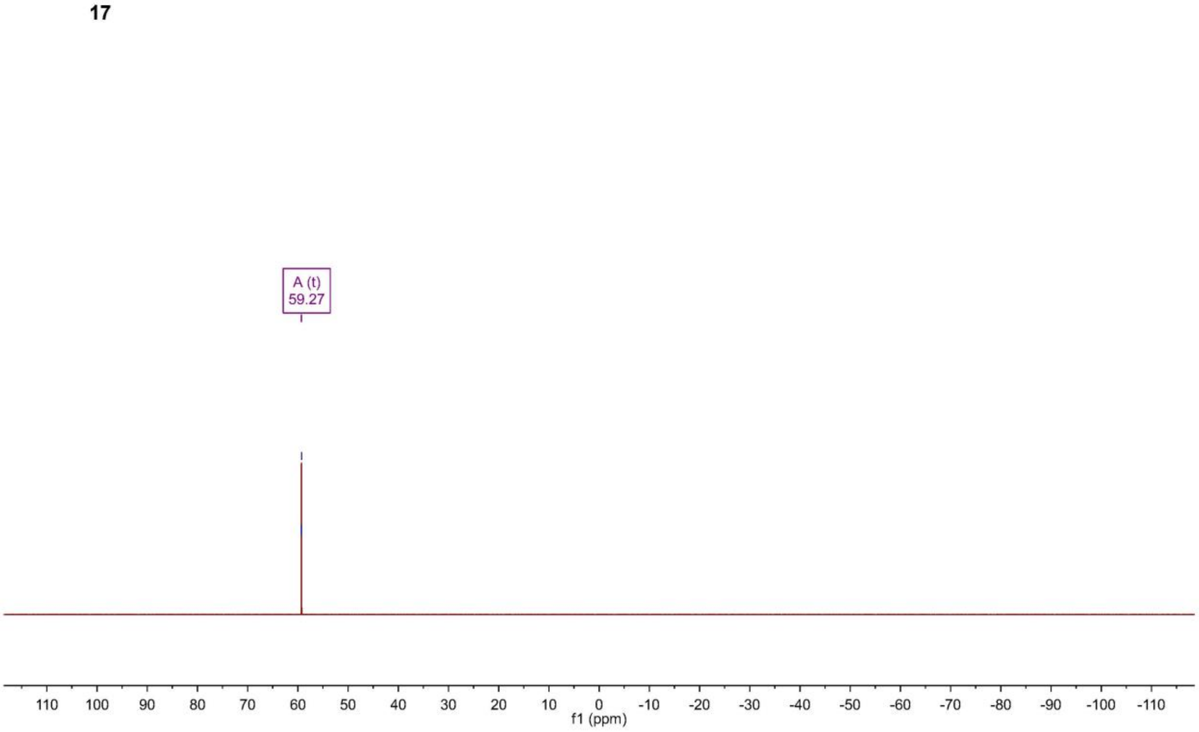

Parameter	Value
1 Solvent	CDCl3
2 Spectrometer Frequency	565

Supplementary Figure 36. ^{1}H, ^{13}C and ^{19}F NMR spectra of **18**.

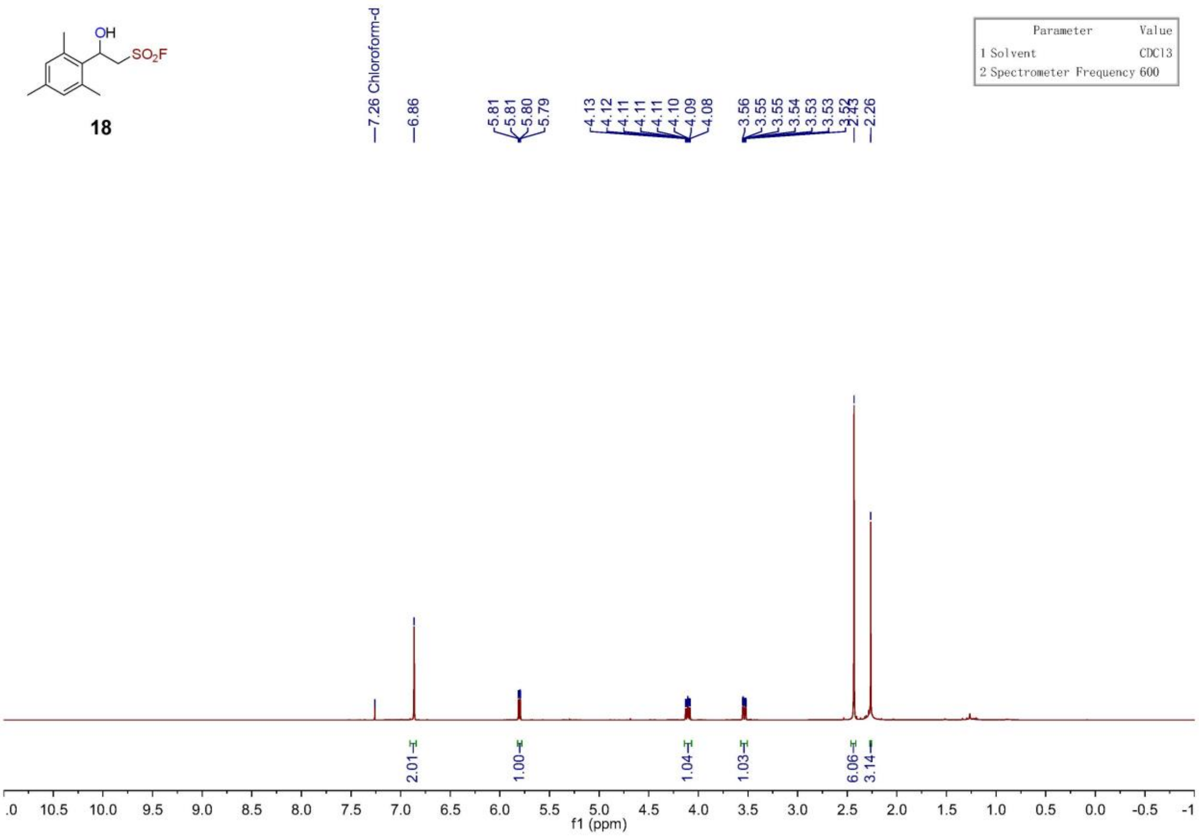

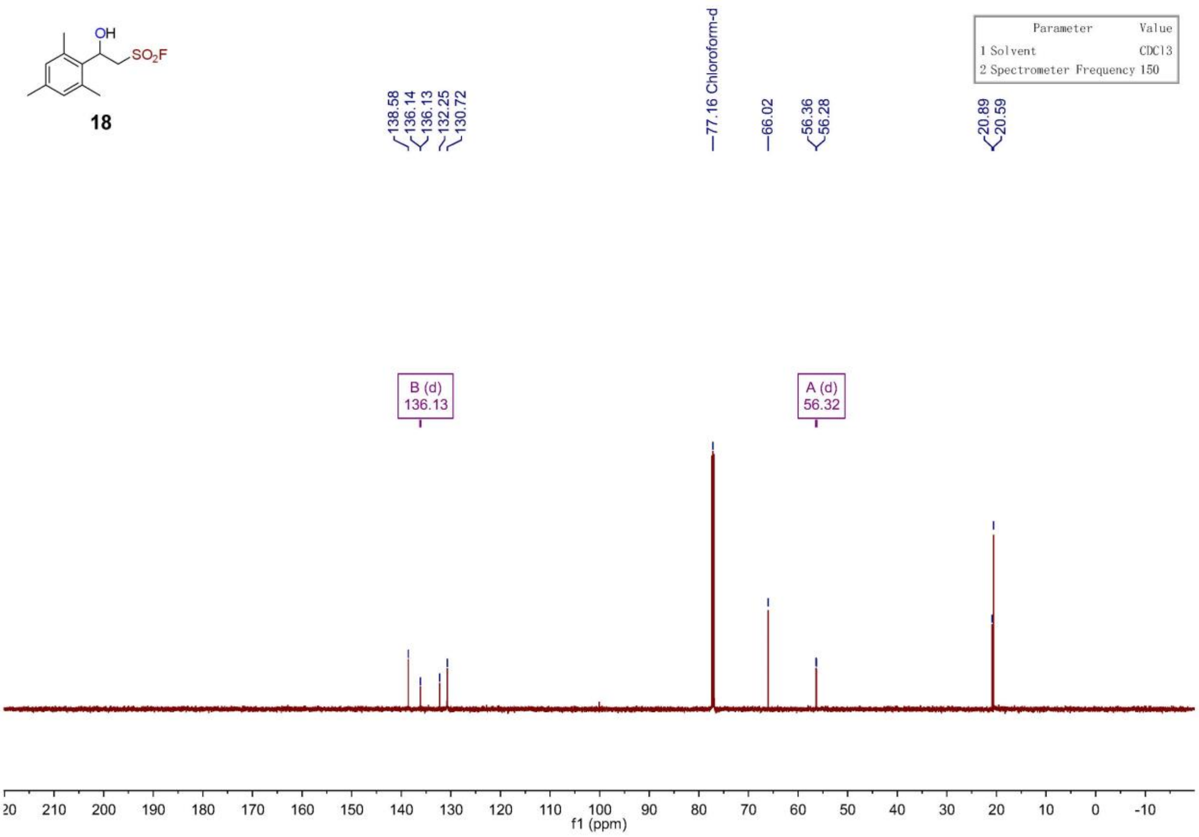

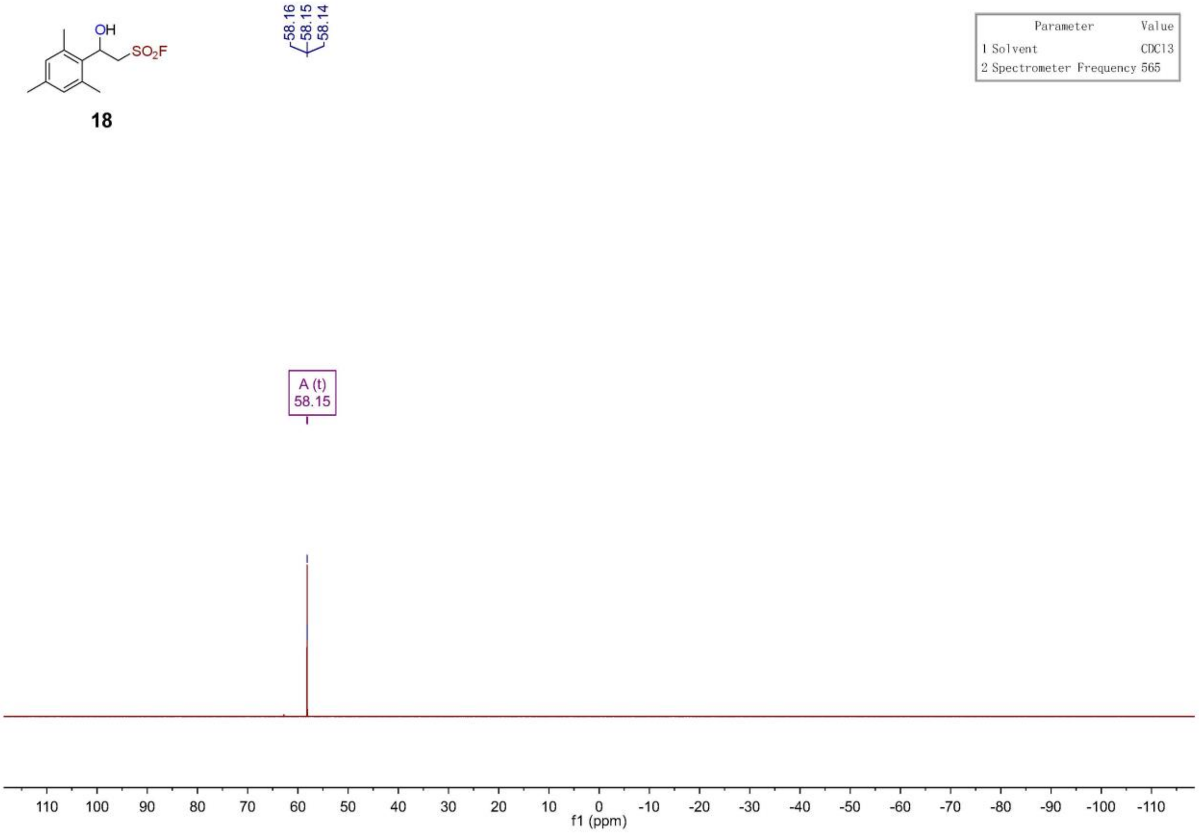

Supplementary Figure 37. ^{1}H, ^{13}C and ^{19}F NMR spectra of **19**.

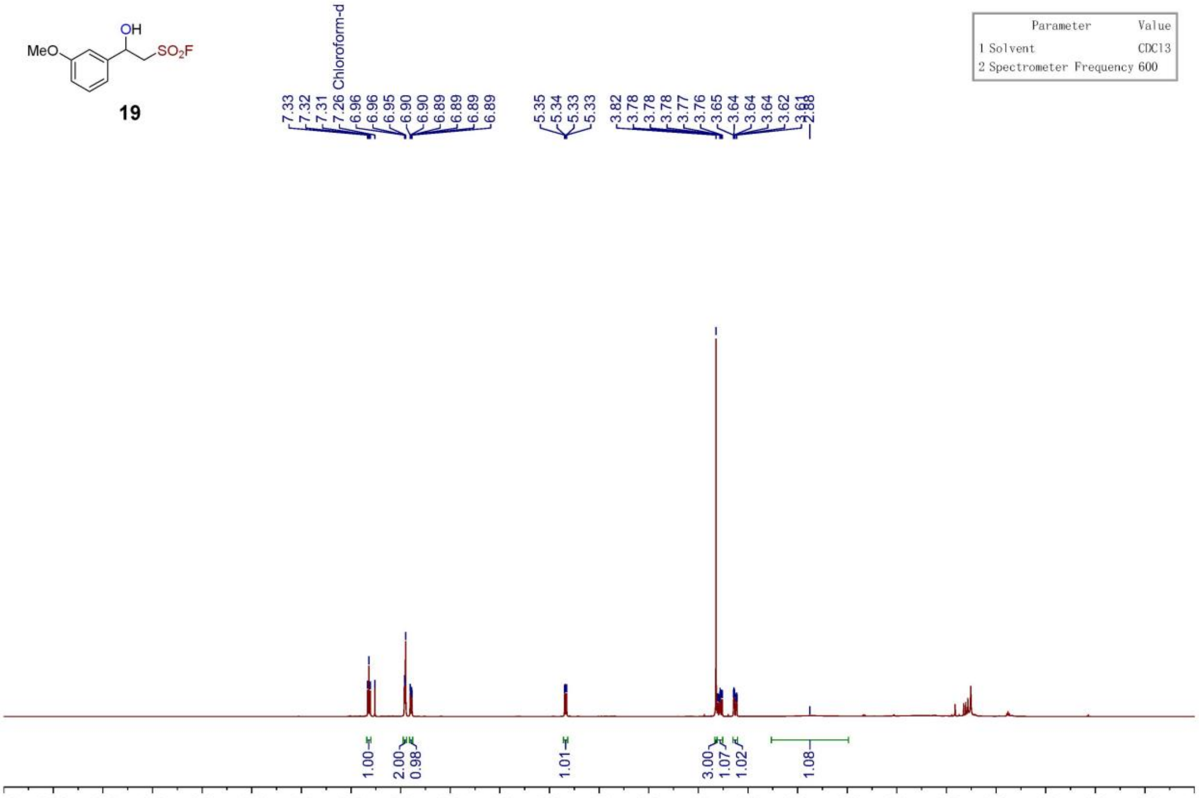

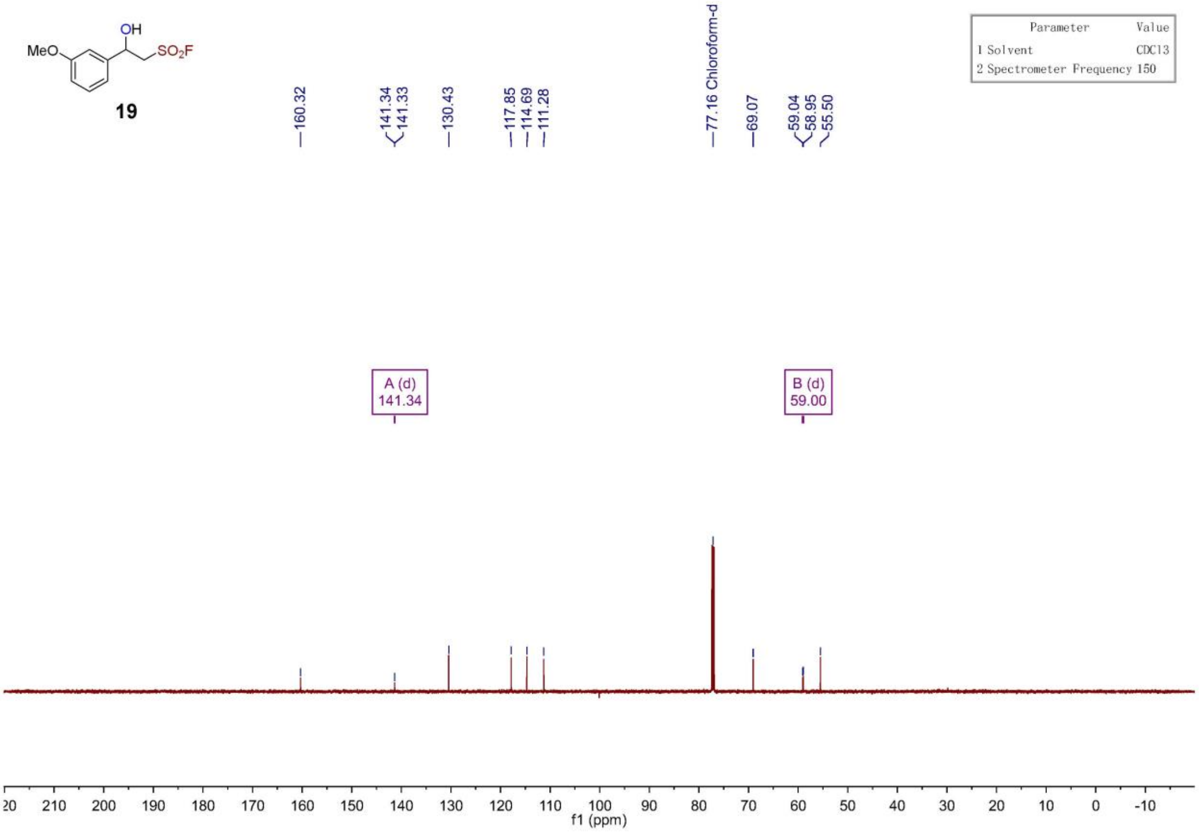

MeO
OH
SO2F
19
160.32
141.34
141.33
130.43
117.85
114.69
111.28
77.16 Chloroform-d
69.07
59.04
58.95
55.50
Parameter Value
1 Solvent CDCl3
2 Spectrometer Frequency 150
A (d)
141.34
B (d)
59.00
f1 (ppm)

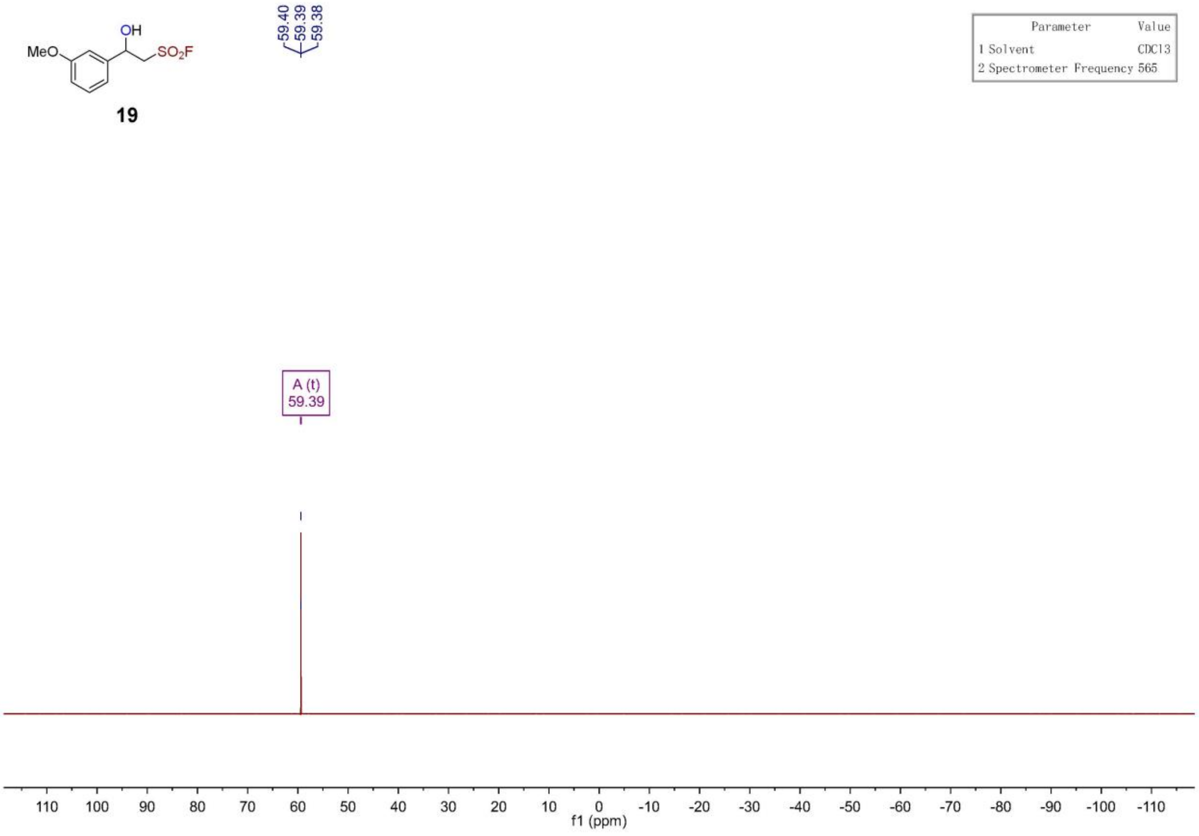

MeO
OH
SO2F
19
59.40
59.39
59.38
Parameter Value
1 Solvent CDCl3
2 Spectrometer Frequency 565
A (t)
59.39
f1 (ppm)

Supplementary Figure 38. ^{1}H, ^{13}C and ^{19}F NMR spectra of **20**.

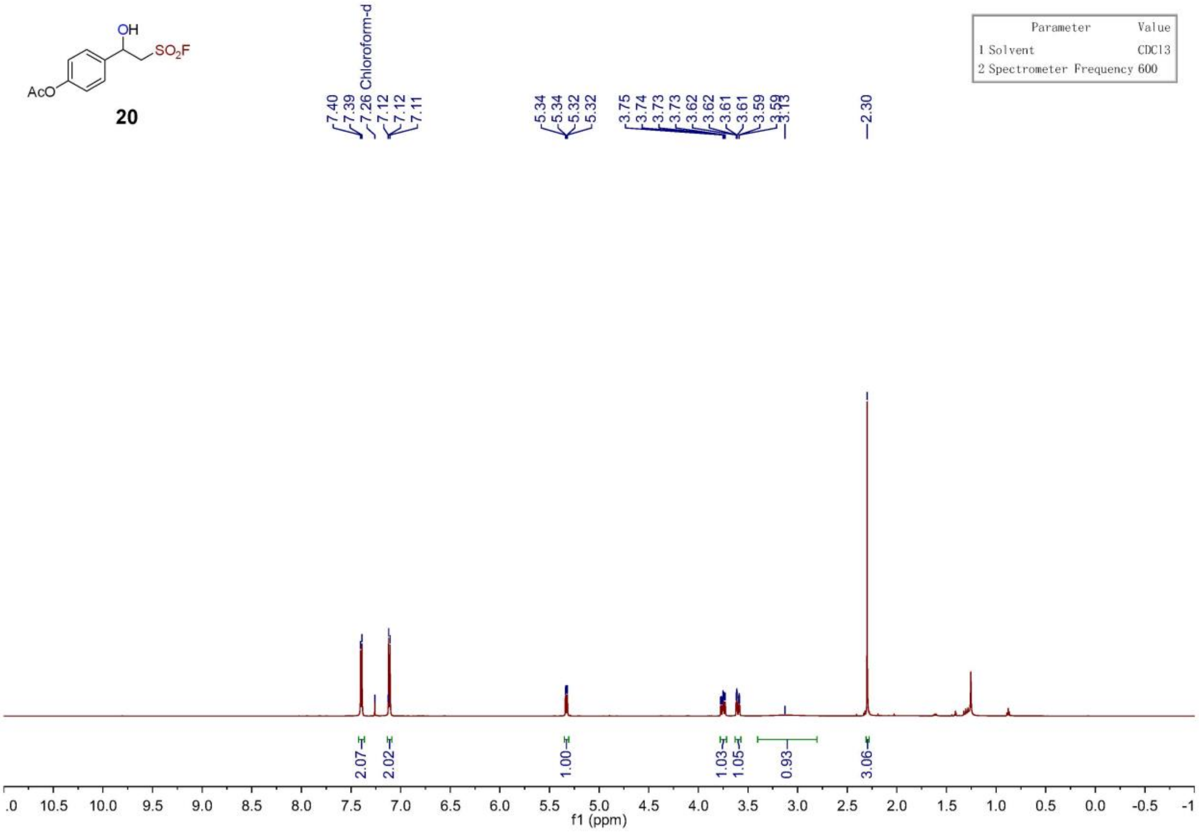

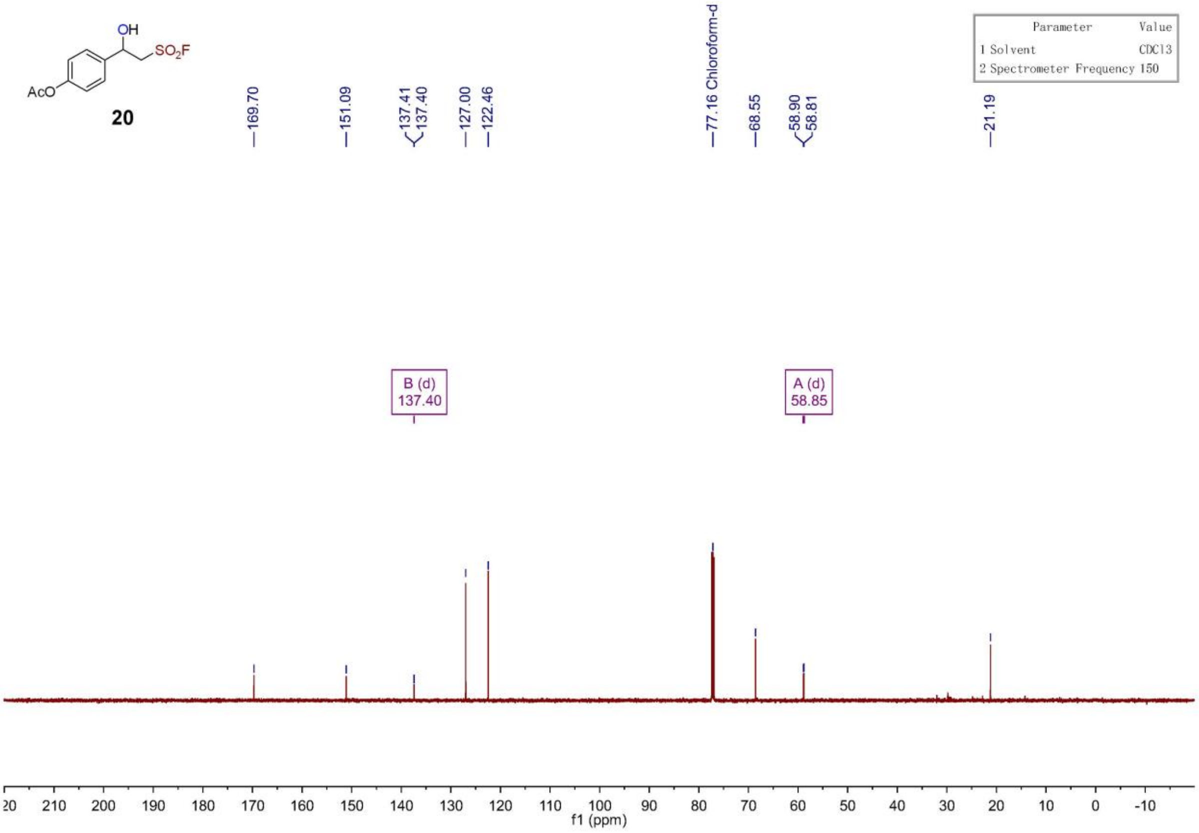

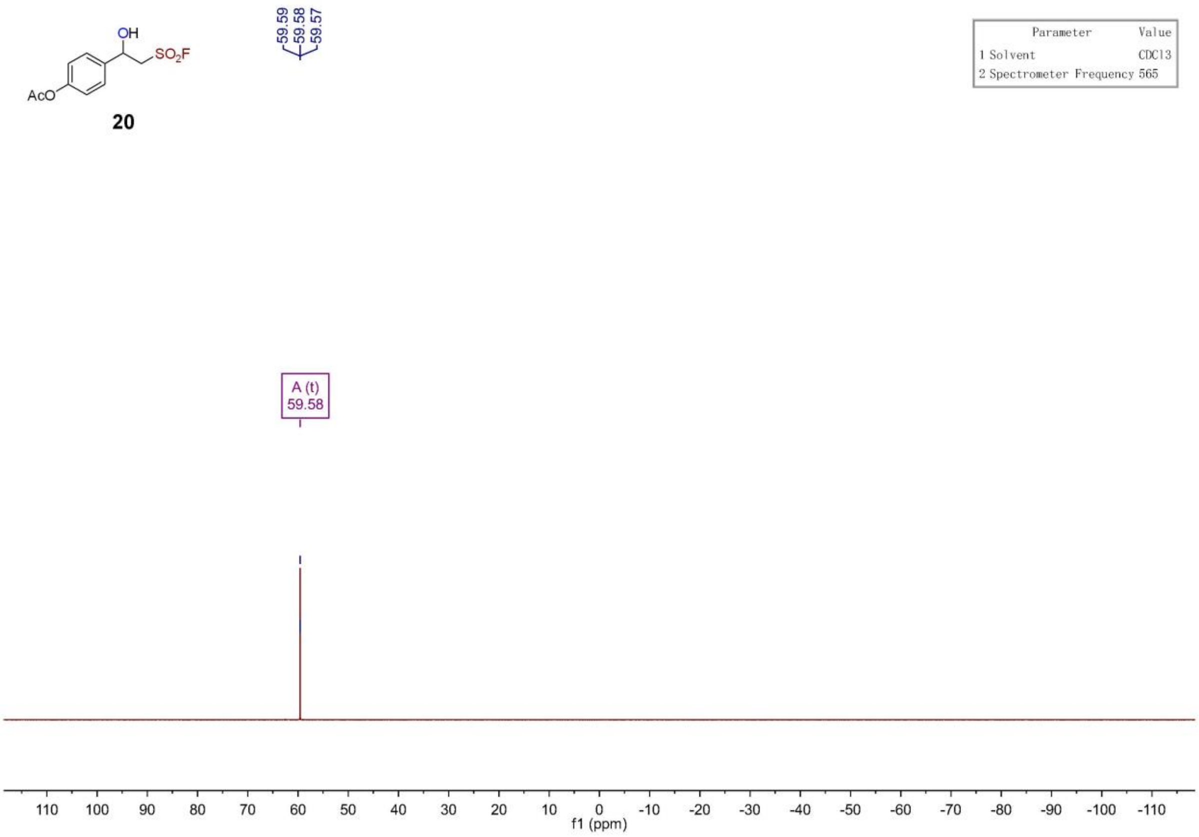

Supplementary Figure 39. ^{1}H, ^{13}C and ^{19}F NMR spectra of **21**.

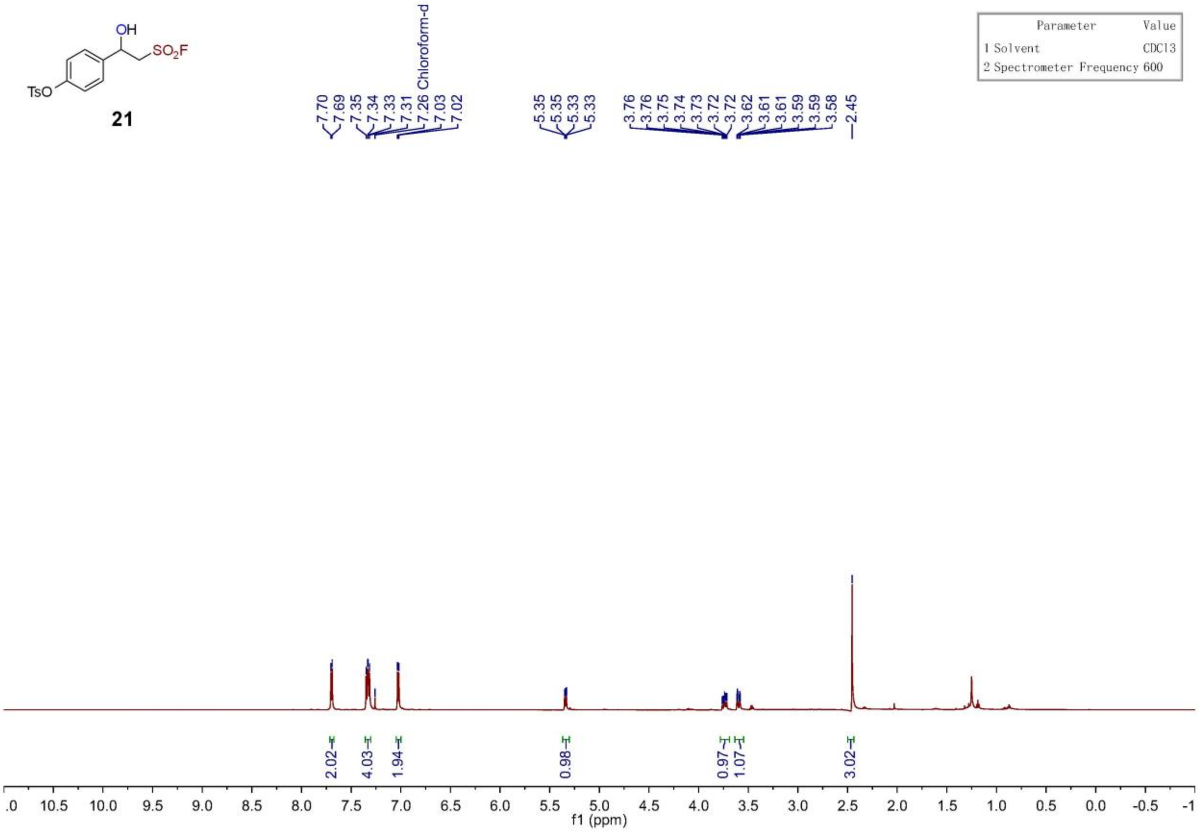

21

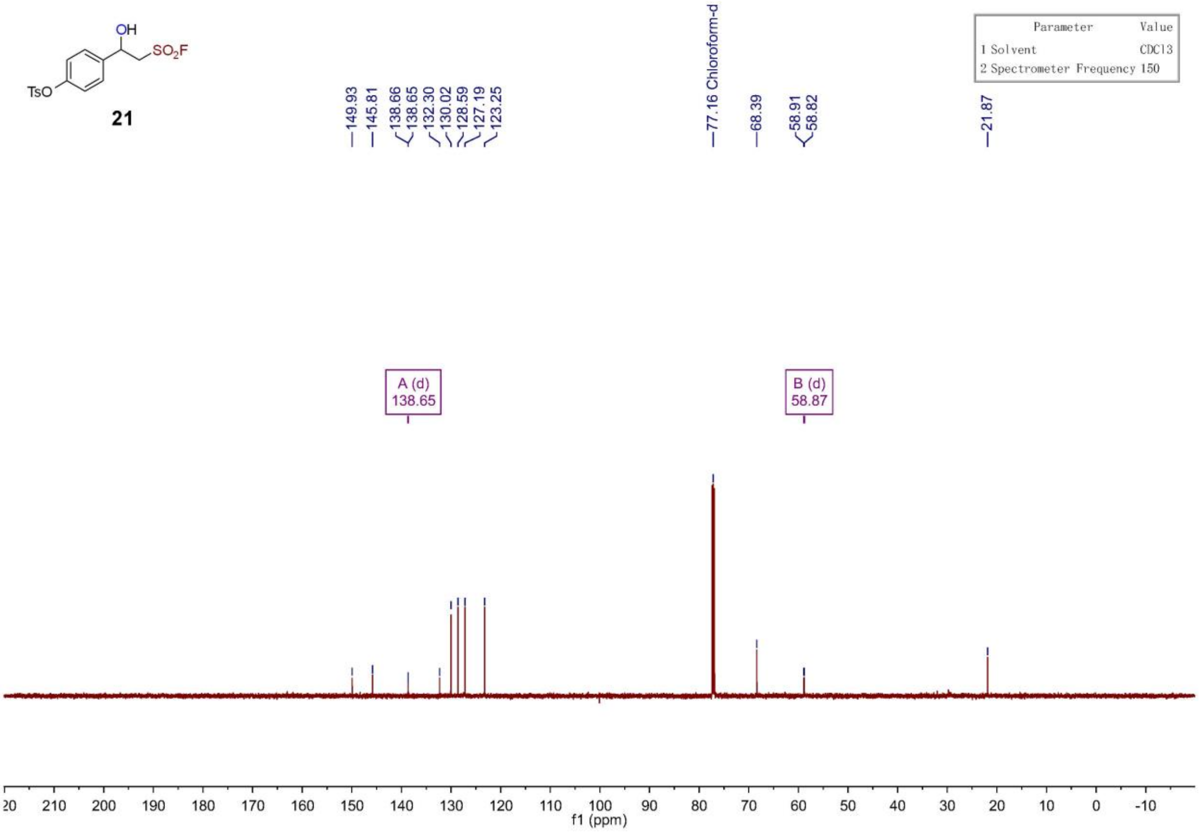
OH
SO2F
TsO
149.93
145.81
138.66
138.65
132.30
130.02
128.59
127.19
123.25
77.16 Chloroform-d
68.39
58.91
58.82
21.87
Parameter Value
1 Solvent CDCl3
2 Spectrometer Frequency 150
A (d)
138.65
B (d)
58.87
f1 (ppm)

21

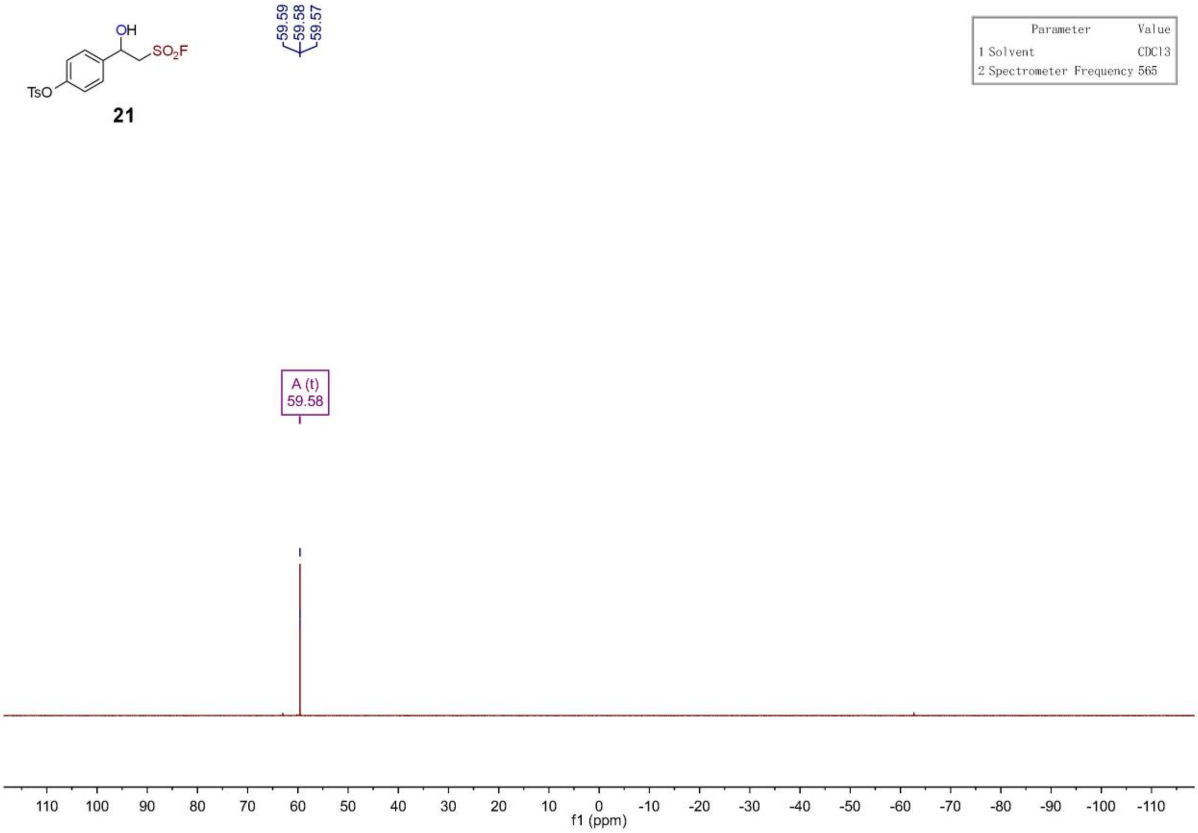
OH
SO2F
TsO
59.59
59.58
59.57
Parameter Value
1 Solvent CDCl3
2 Spectrometer Frequency 565
A (t)
59.58
f1 (ppm)

Supplementary Figure 40. ^{1}H, ^{13}C and ^{19}F NMR spectra of **22**.

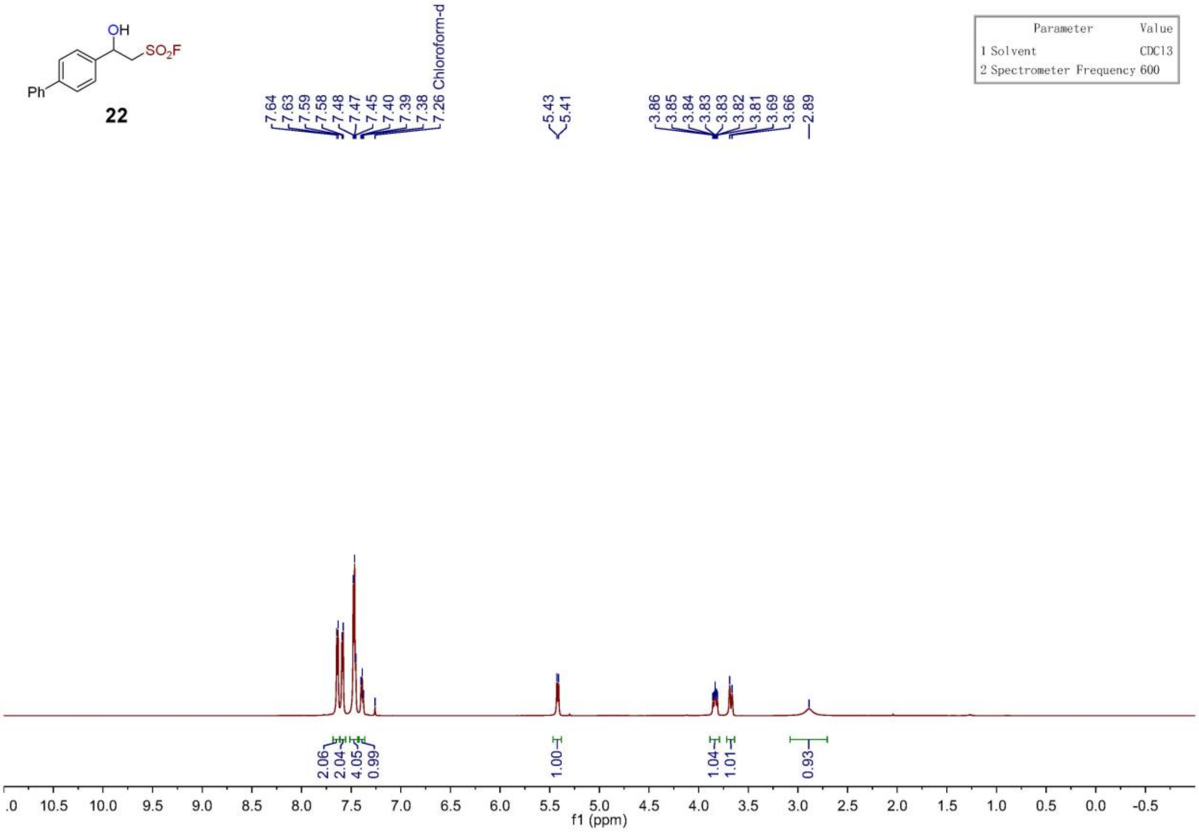

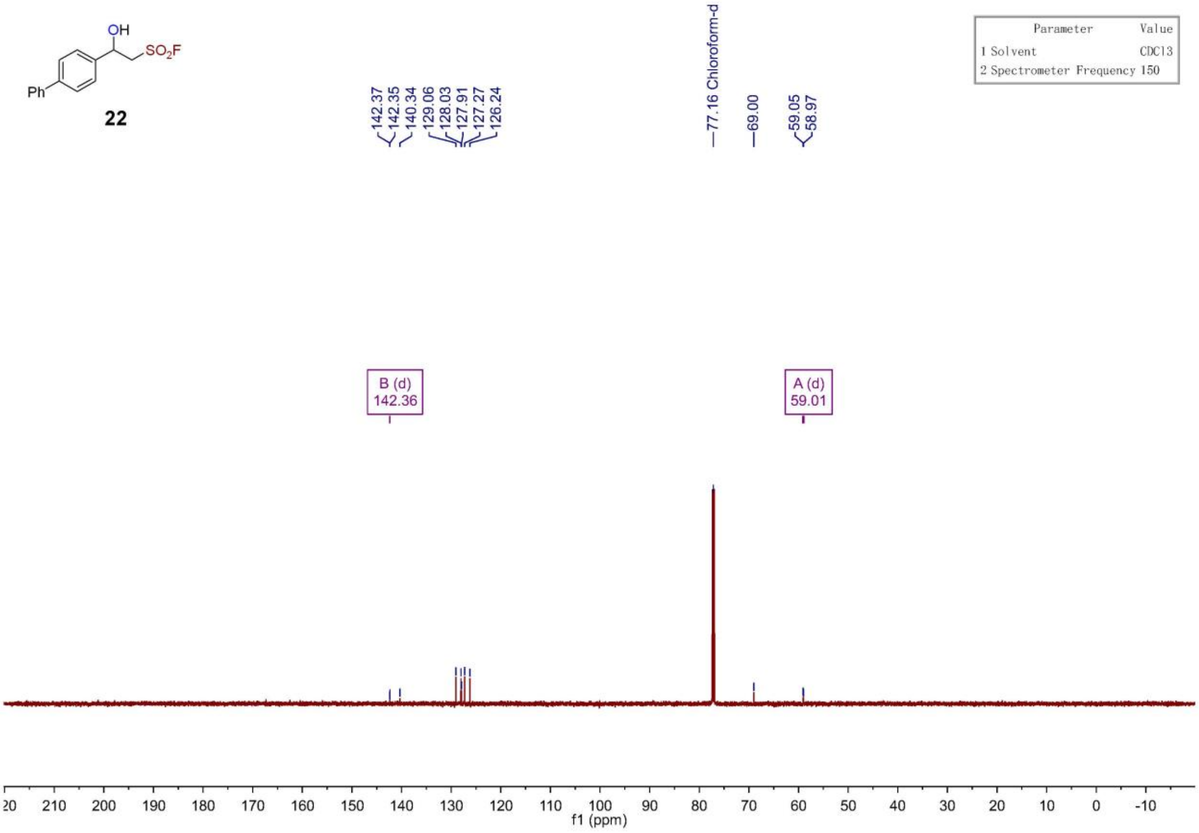

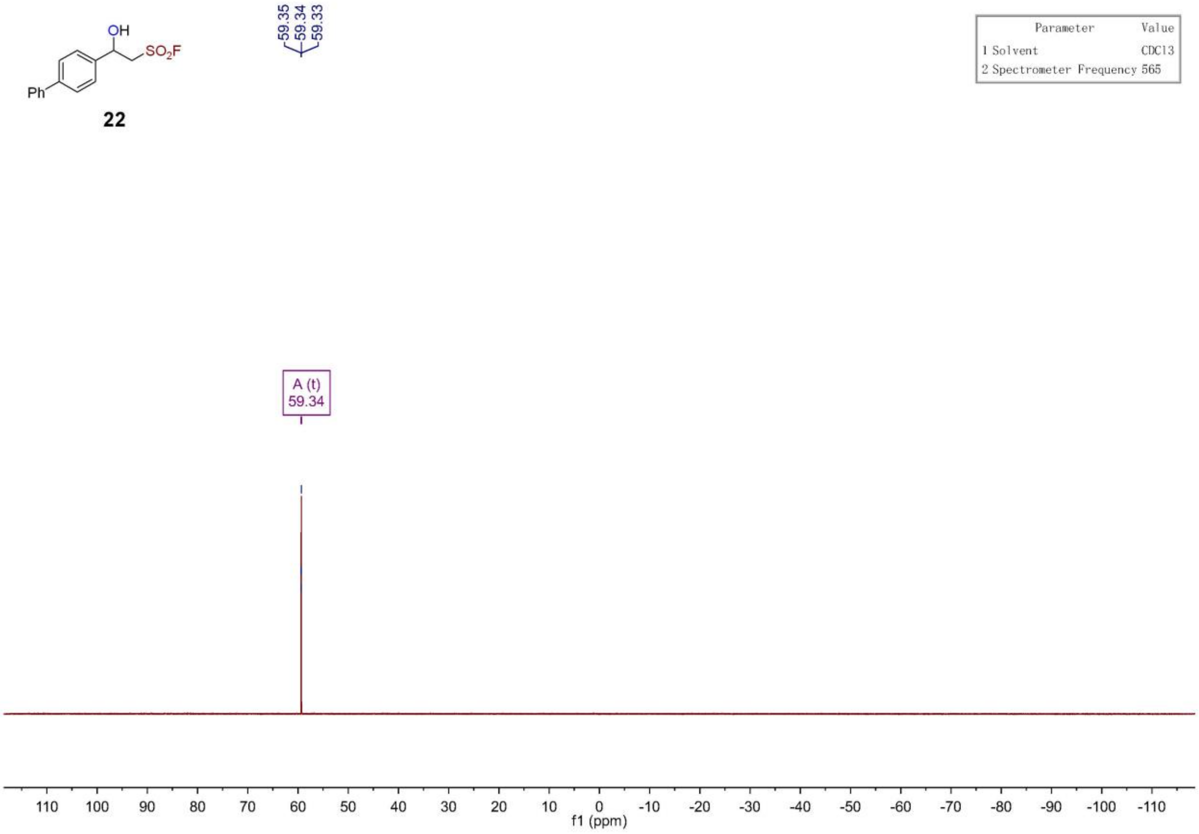

Supplementary Figure 41. ^{1}H, ^{13}C and ^{19}F NMR spectra of **23**.

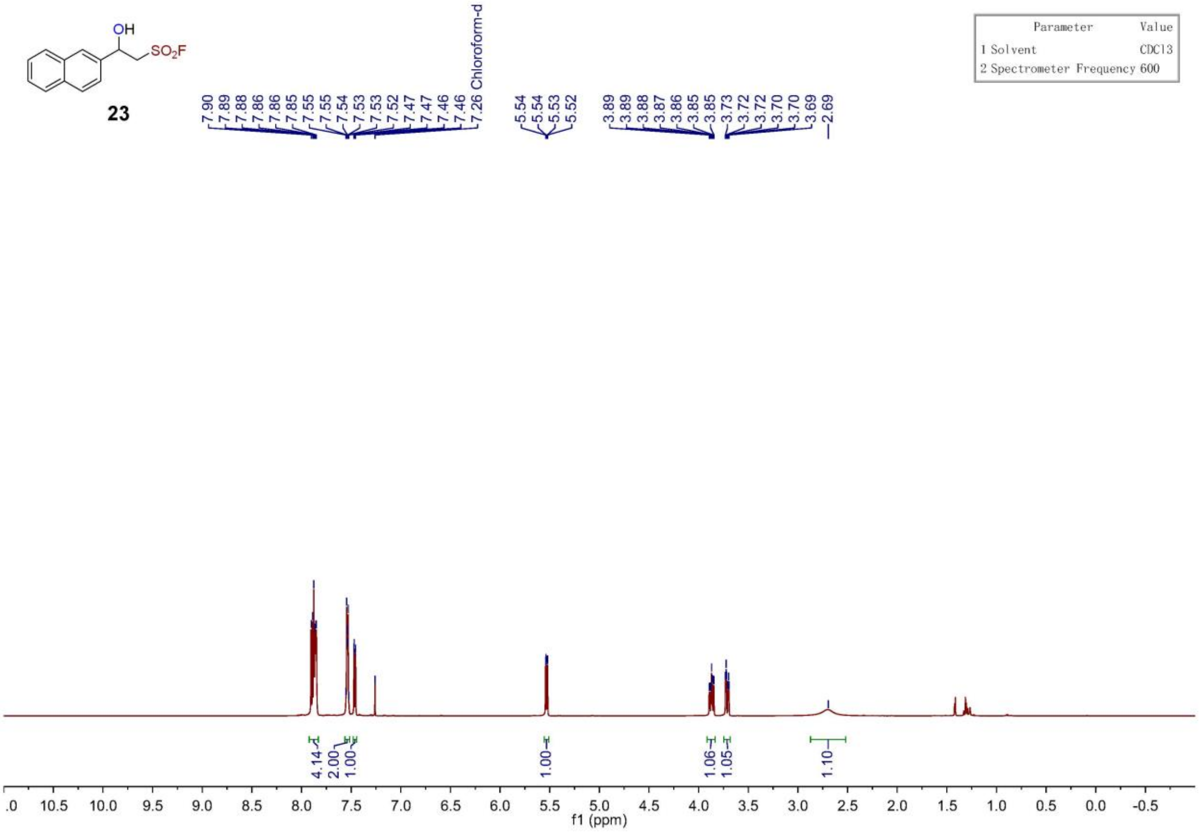

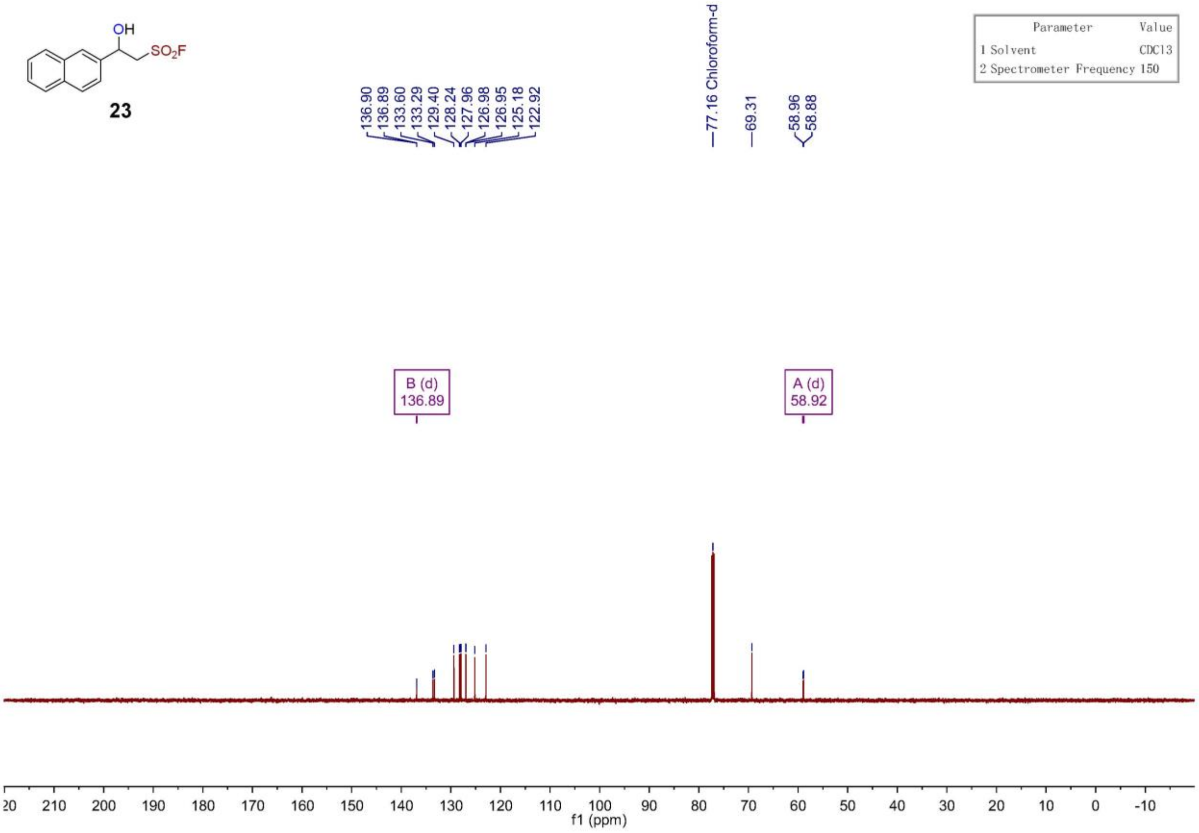
OH
SO2F
23
136.90
136.89
133.60
133.29
129.40
128.24
127.96
126.98
126.95
125.18
122.92
77.16 Chloroform-d
69.31
58.96
58.88
Parameter Value
1 Solvent CDCl3
2 Spectrometer Frequency 150
B (d)
136.89
A (d)
58.92
f1 (ppm)

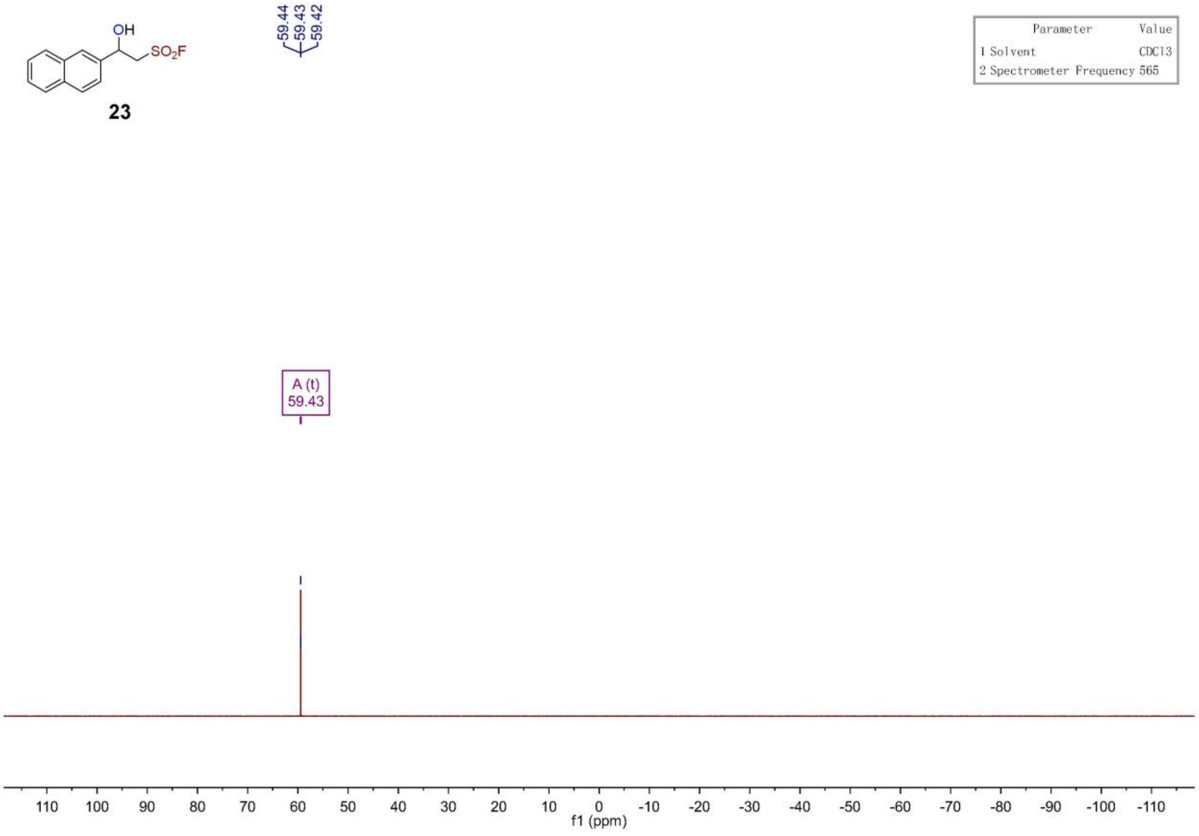
OH
SO2F
23
59.44
59.43
59.42
Parameter Value
1 Solvent CDCl3
2 Spectrometer Frequency 565
A (t)
59.43
f1 (ppm)

Supplementary Figure 42. ^{1}H, ^{13}C and ^{19}F NMR spectra of **24**.

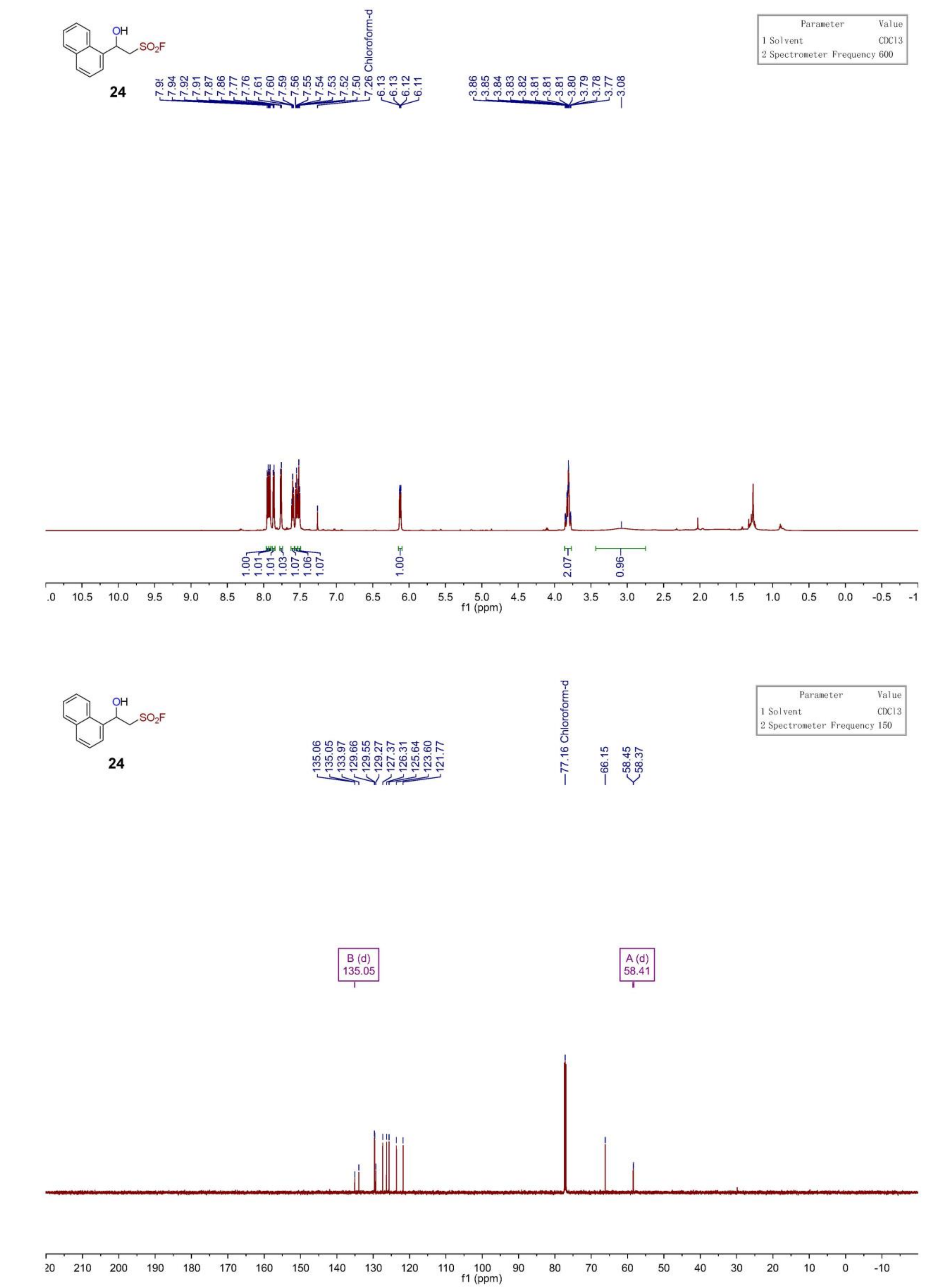

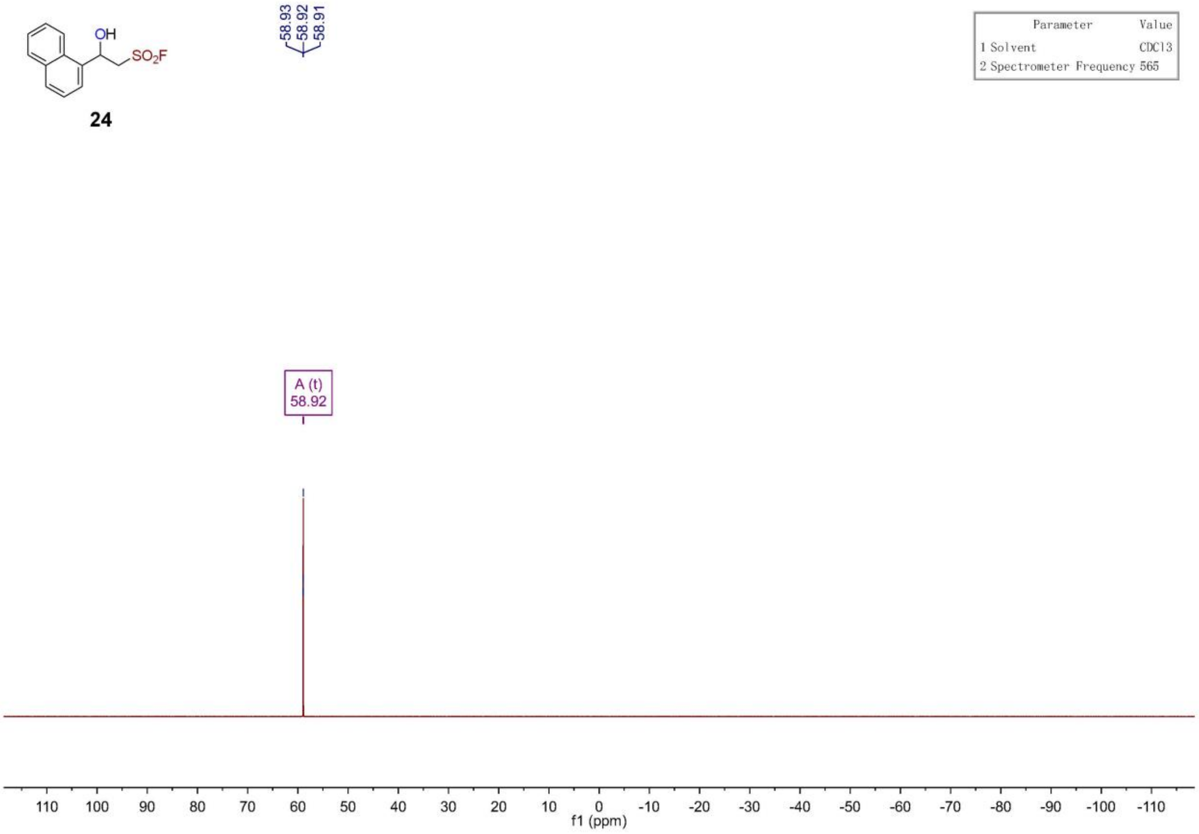

Supplementary Figure 43. ^{1}H, ^{13}C and ^{19}F NMR spectra of **25**.

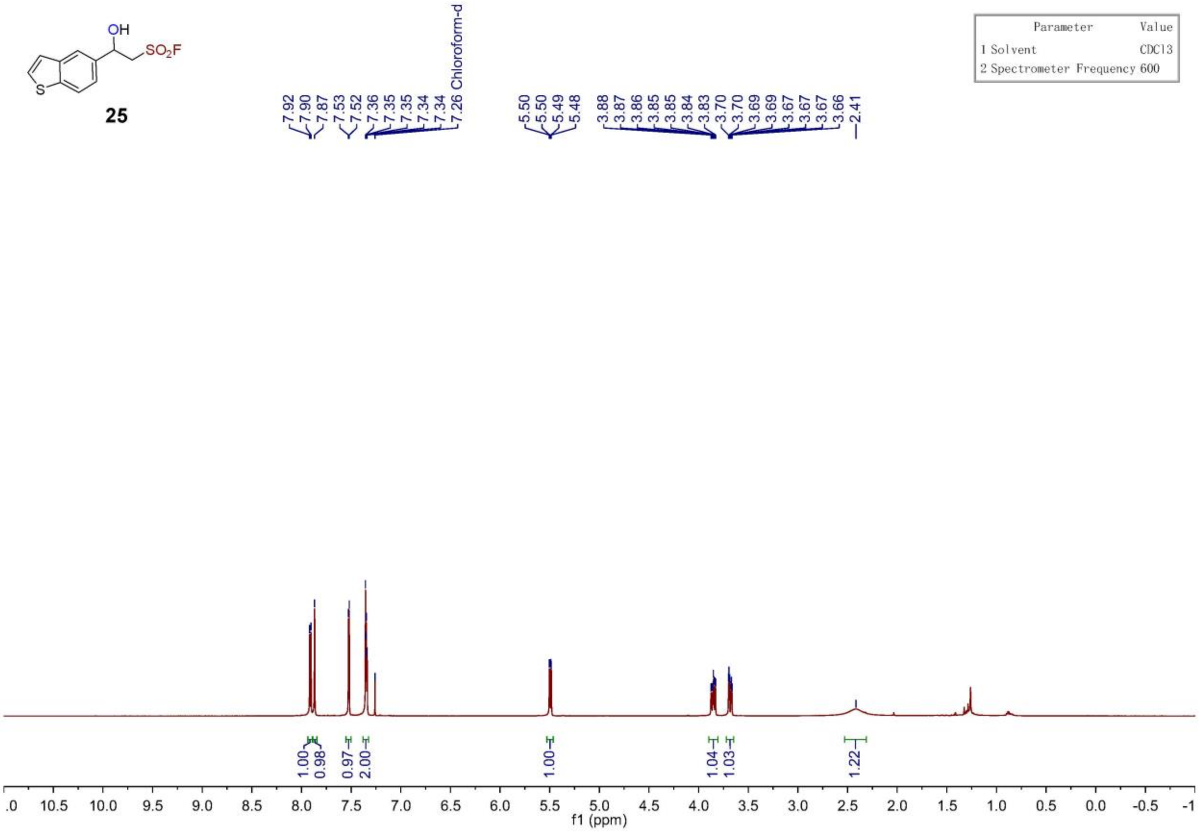

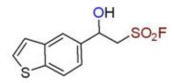

25

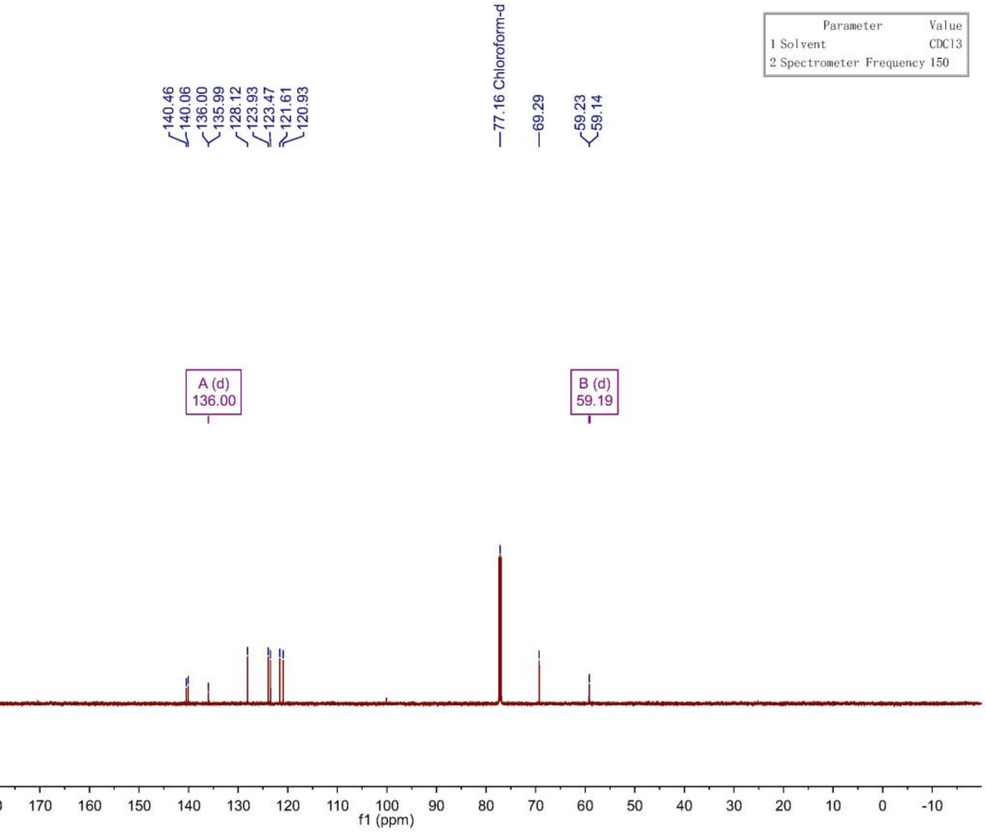

Parameter Value
1 Solvent CDCl3
2 Spectrometer Frequency 150
140.46
140.06
136.00
135.99
128.12
123.93
123.47
121.61
120.93
77.16 Chloroform-d
69.29
59.23
59.14
A (d) 136.00
B (d) 59.19
f1 (ppm)

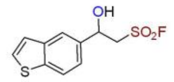

25

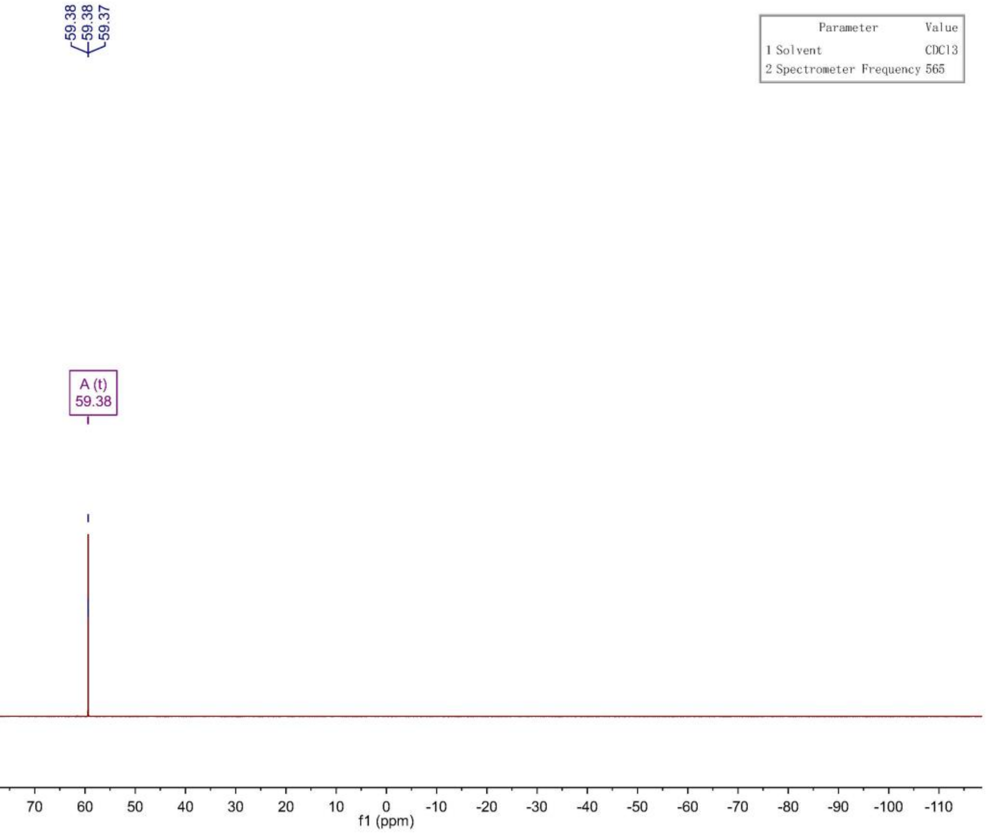

Parameter Value
1 Solvent CDCl3
2 Spectrometer Frequency 565
59.38
59.38
59.37
A (t) 59.38
f1 (ppm)

Supplementary Figure 44. ^{1}H, ^{13}C and ^{19}F NMR spectra of **26**.

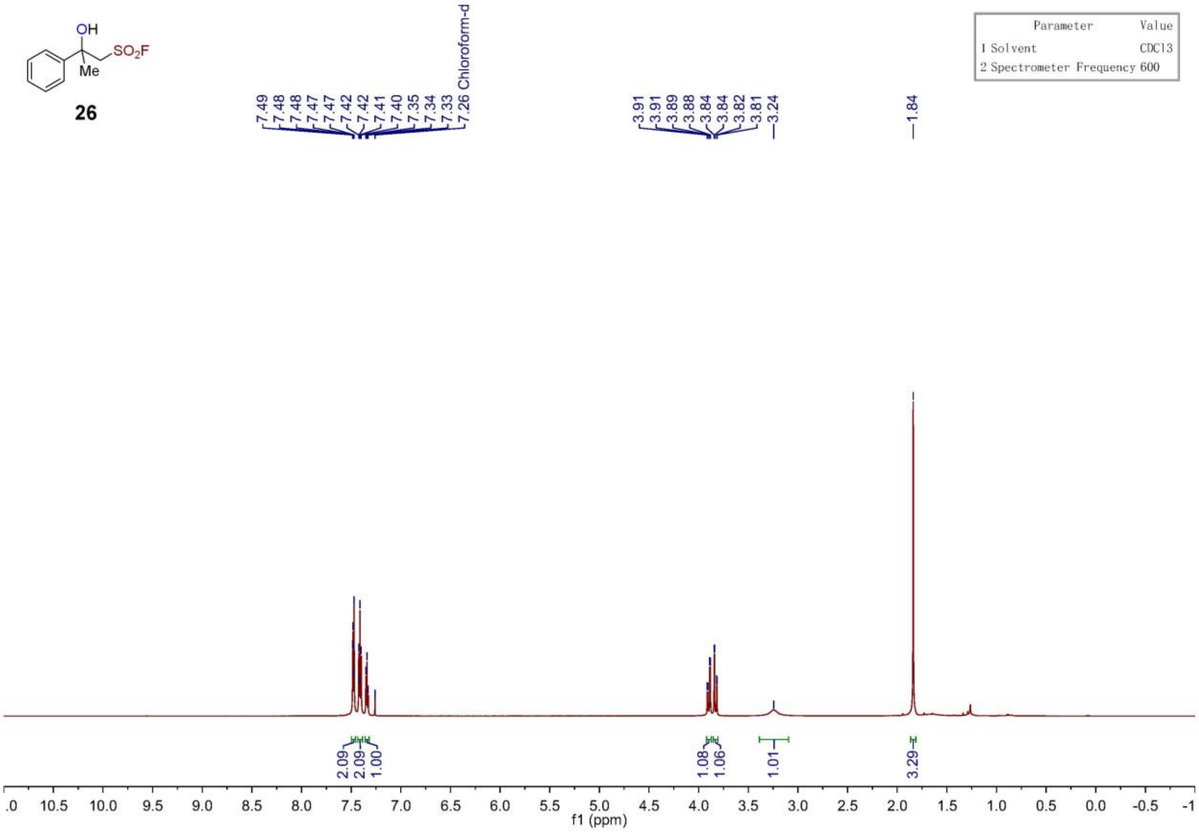

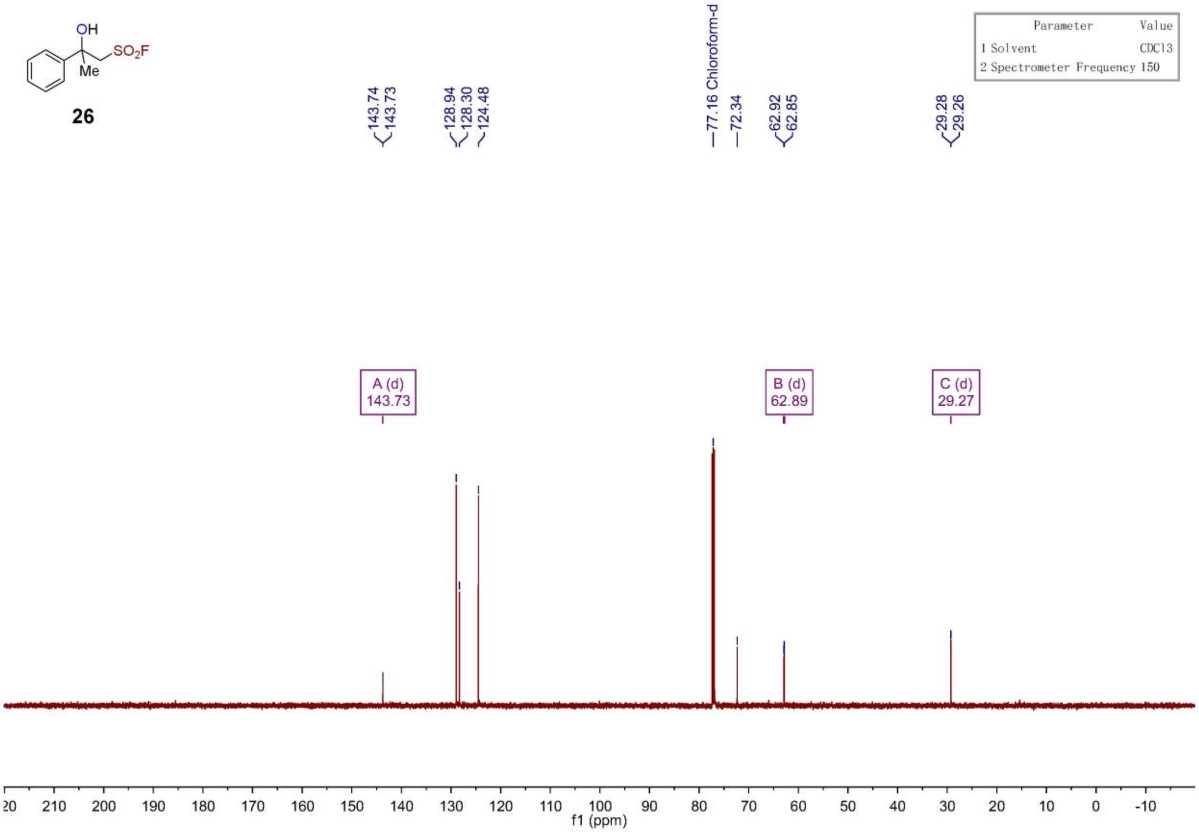

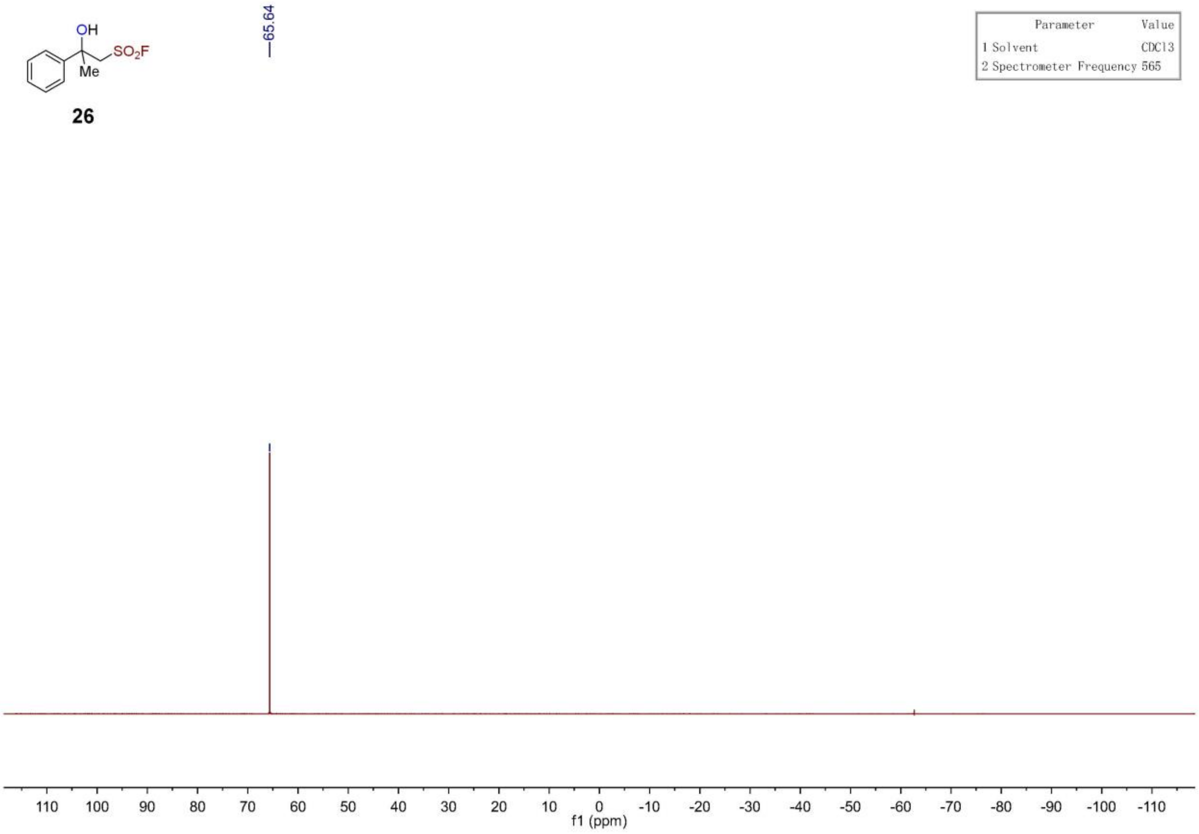

Supplementary Figure 45. ^{1}H, ^{13}C and ^{19}F NMR spectra of **27**.

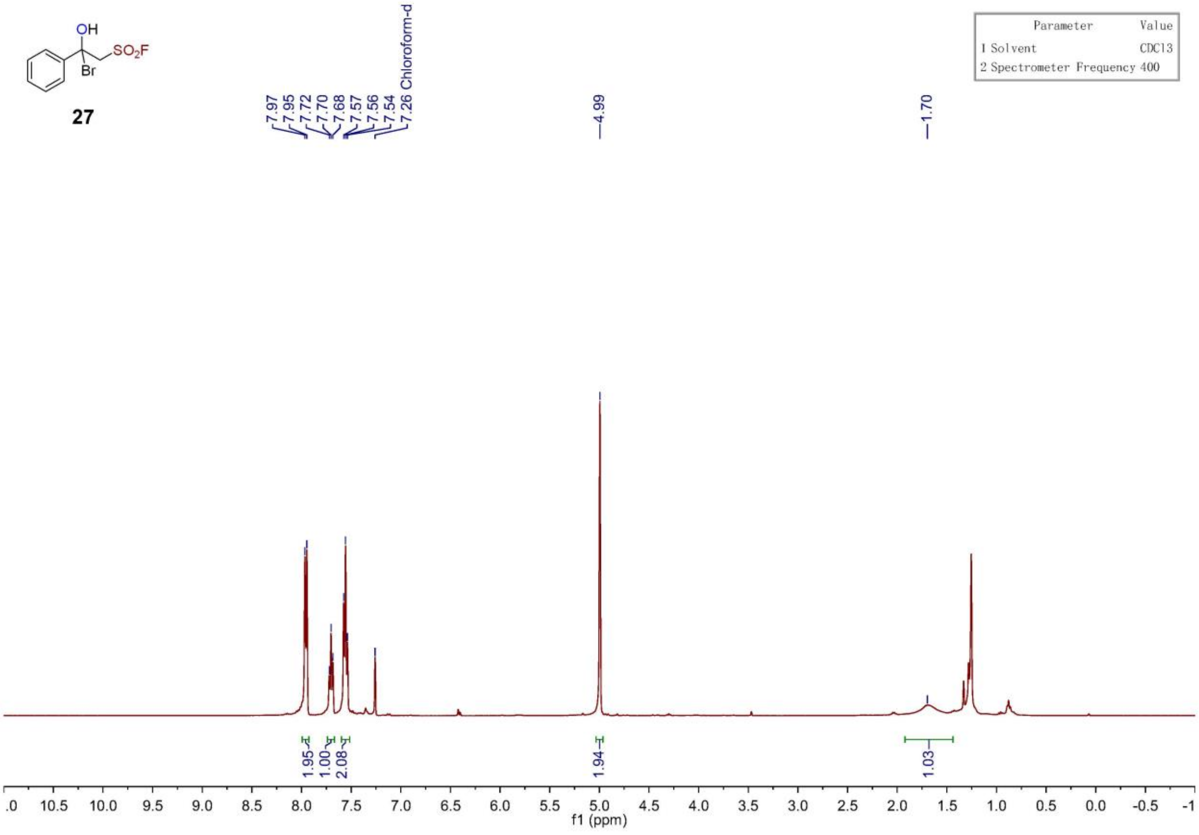

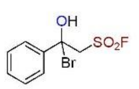

27

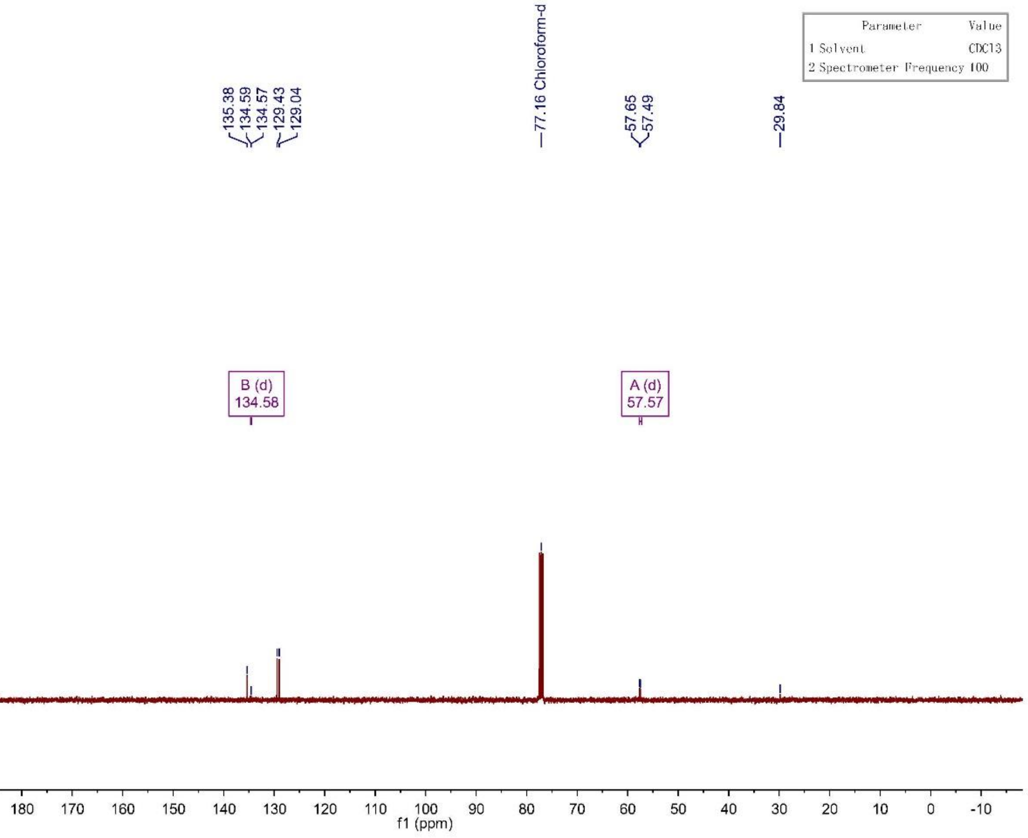

135.38
134.59
134.57
129.43
129.04
77.16 Chloroform-d
57.65
57.49
29.84
Parameter Value
1 Solvent CDCl3
2 Spectrometer Frequency 100
B (d)
134.58
A (d)
57.57
210 200 190 180 170 160 150 140 130 120 110 100 90 80 70 60 50 40 30 20 10 0 -10
f1 (ppm)

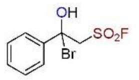

27

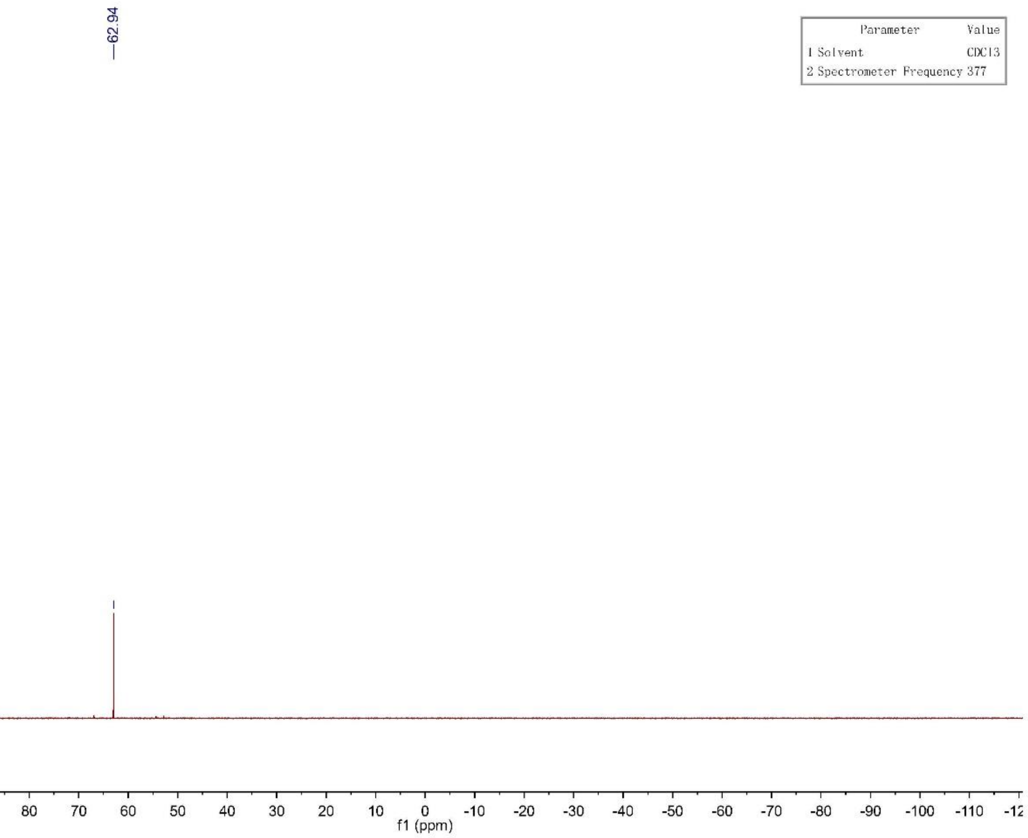

62.94
Parameter Value
1 Solvent CDCl3
2 Spectrometer Frequency 377
20 110 100 90 80 70 60 50 40 30 20 10 0 -10 -20 -30 -40 -50 -60 -70 -80 -90 -100 -110 -12
f1 (ppm)

Supplementary Figure 46. ^{1}H, ^{13}C and ^{19}F NMR spectra of **28**.

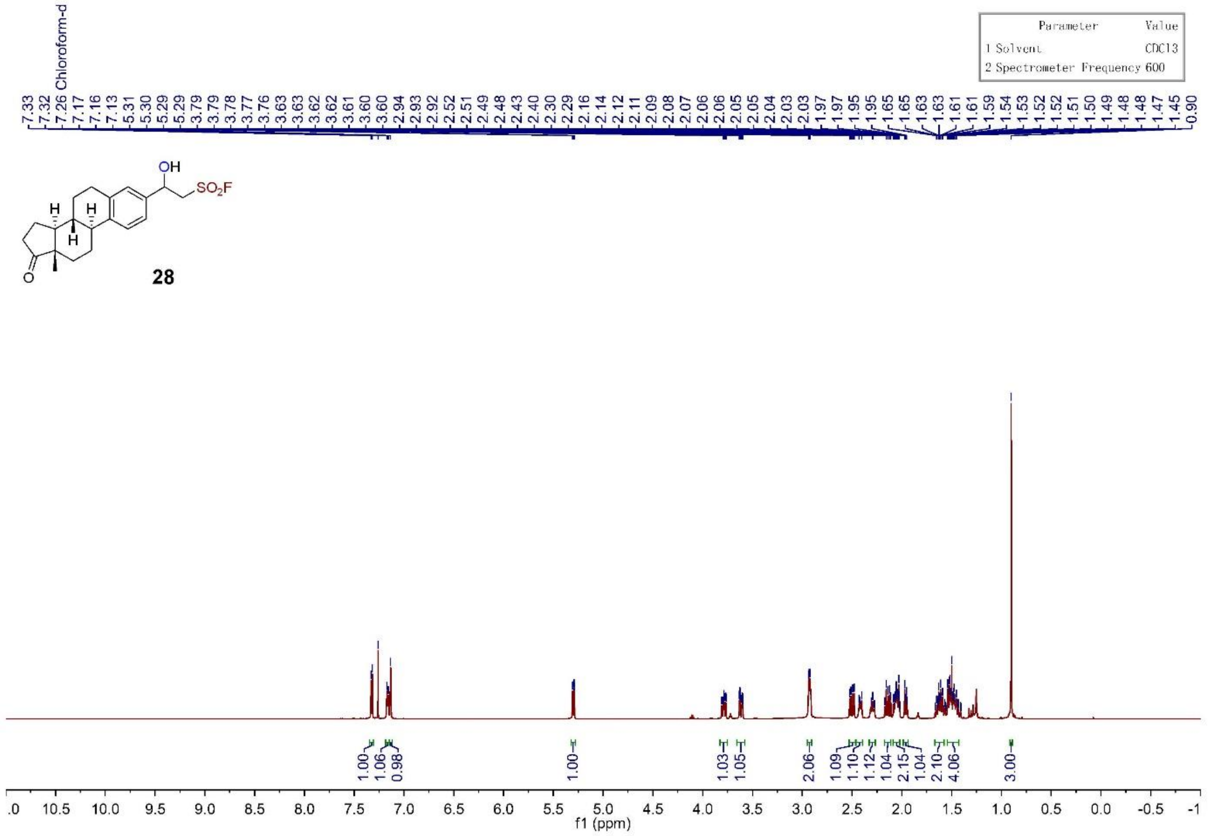

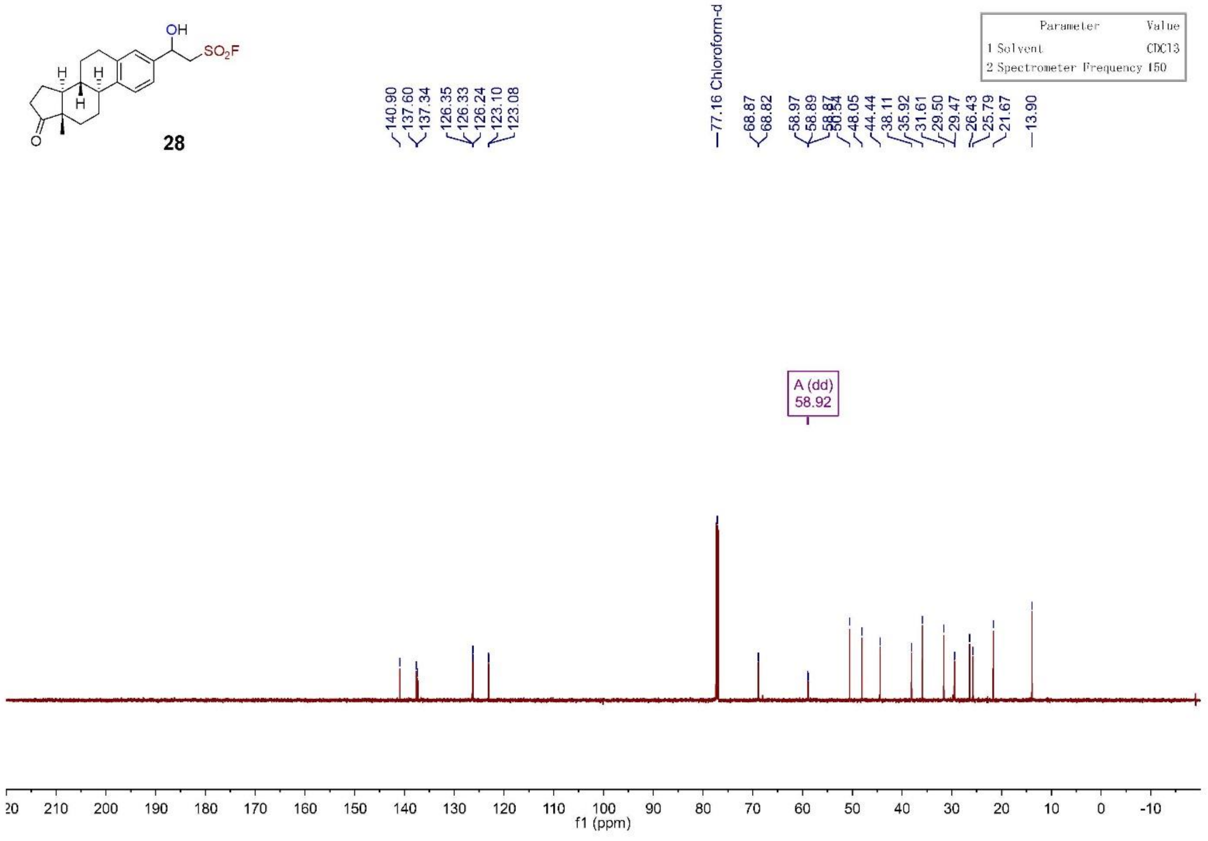

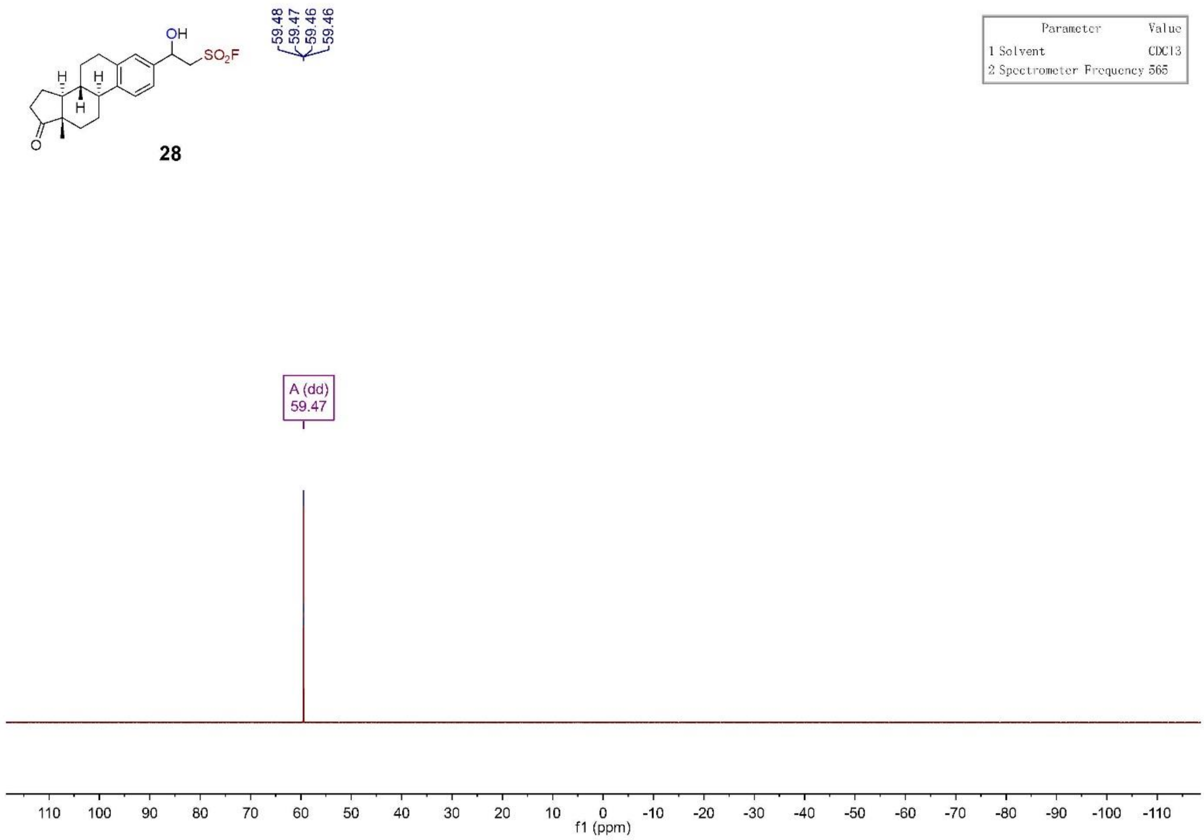

Supplementary Figure 47. ^{1}H, ^{13}C and ^{19}F NMR spectra of **29**.

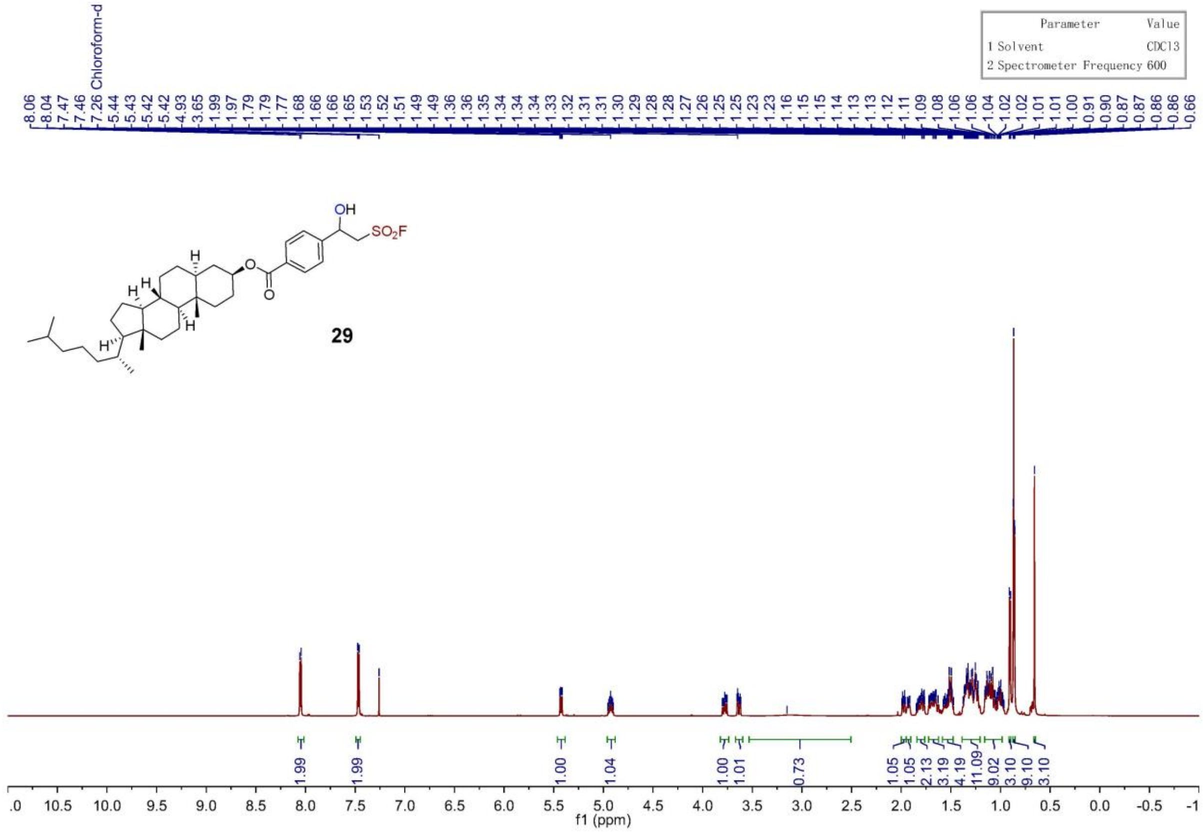

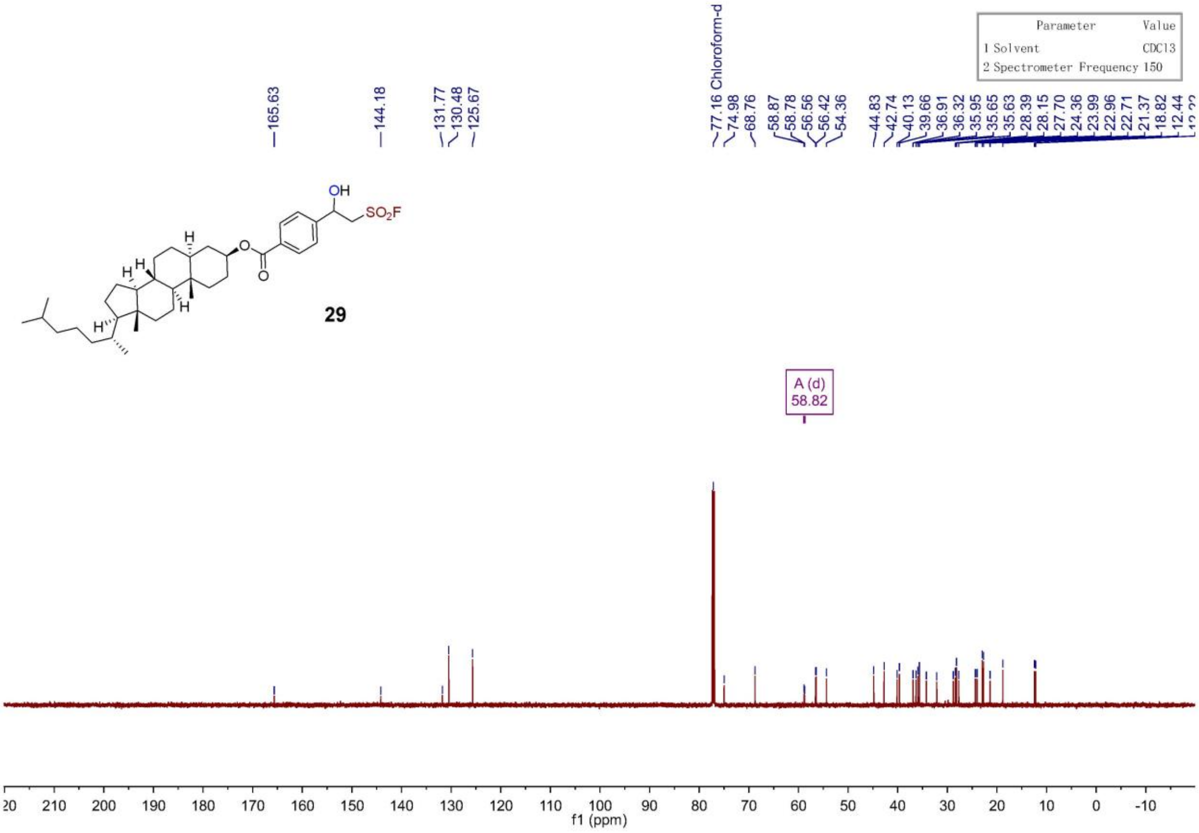
165.63
144.18
131.77
130.48
125.67
77.16 Chloroform-d
74.98
68.76
58.87
58.78
56.56
56.42
54.36
44.83
42.74
40.13
39.66
36.91
36.32
35.95
35.65
35.63
28.39
28.15
27.70
24.36
23.99
22.96
22.71
21.37
18.82
12.44
Parameter Value
1 Solvent CDCl3
2 Spectrometer Frequency 150
OH
SO2F
29
A (d)
58.82
f1 (ppm)

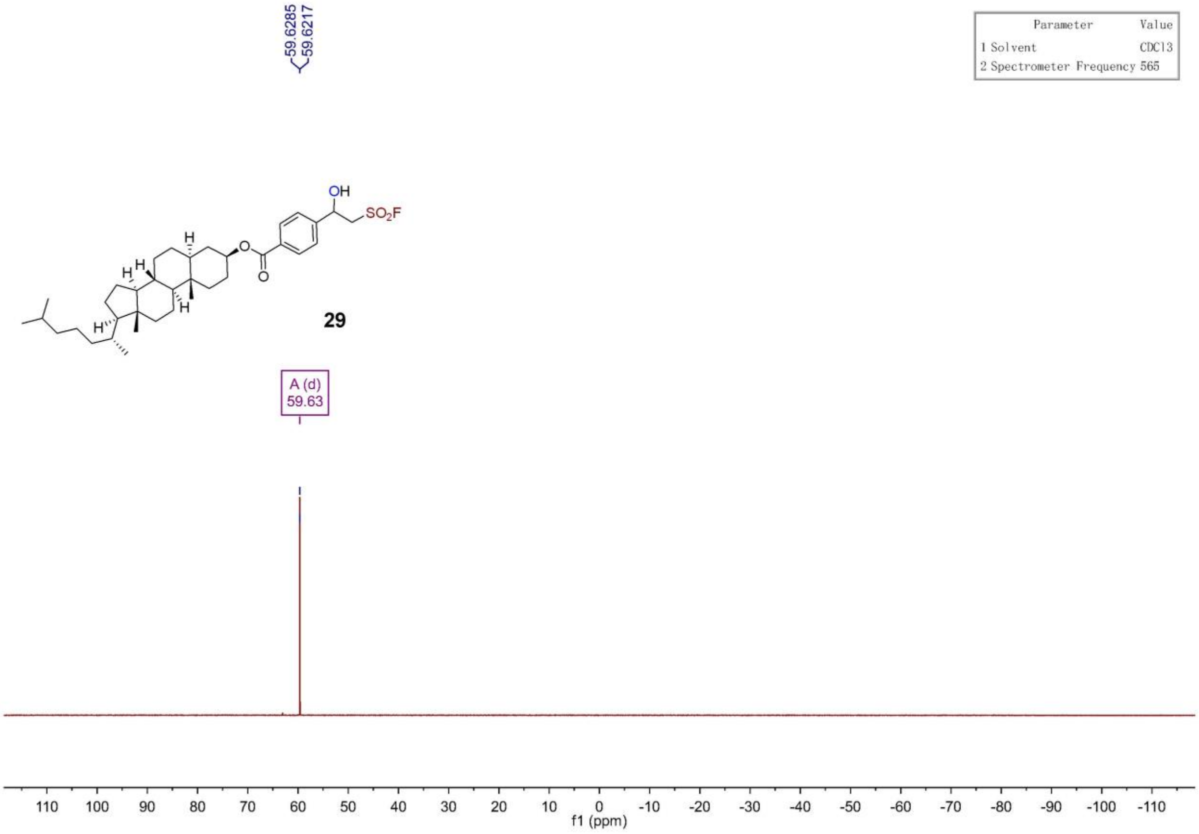
59.6285
59.6217
Parameter Value
1 Solvent CDCl3
2 Spectrometer Frequency 565
OH
SO2F
29
A (d)
59.63
f1 (ppm)

Supplementary Figure 48. ^{1}H, ^{13}C and ^{19}F NMR spectra of **30**.

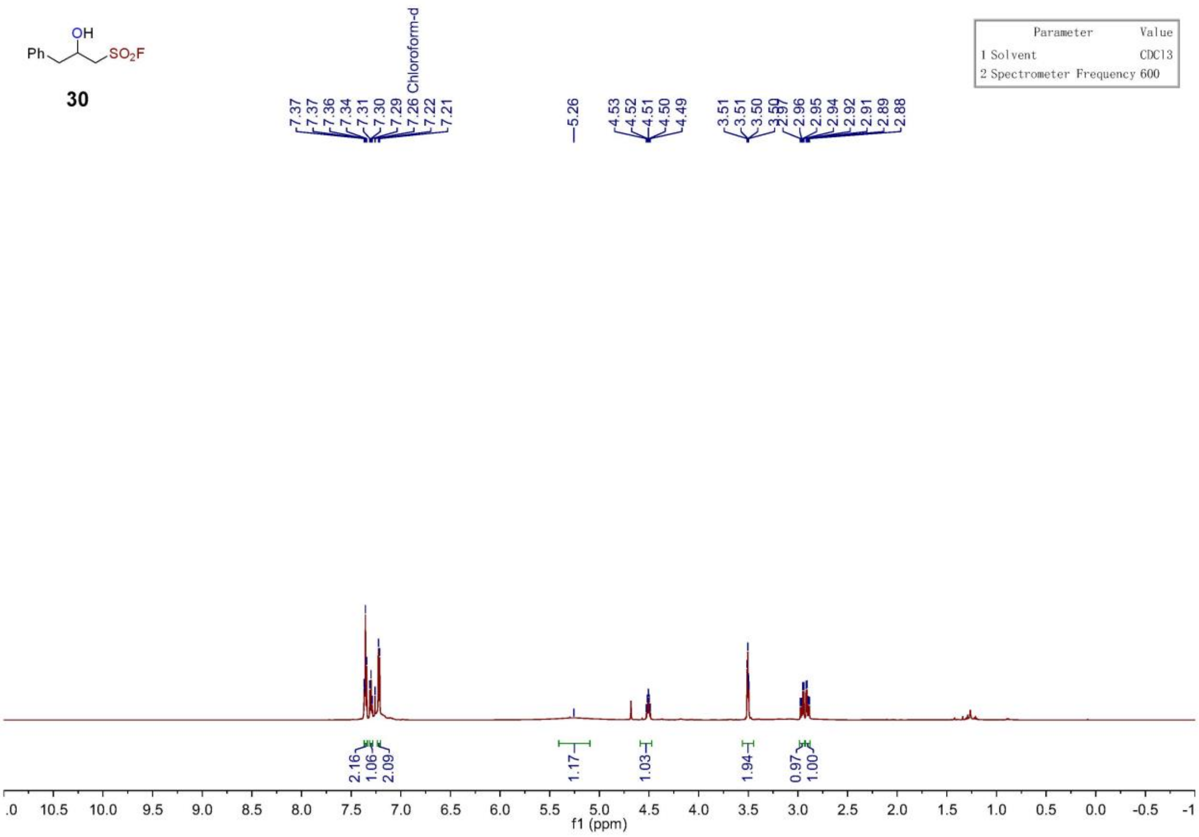

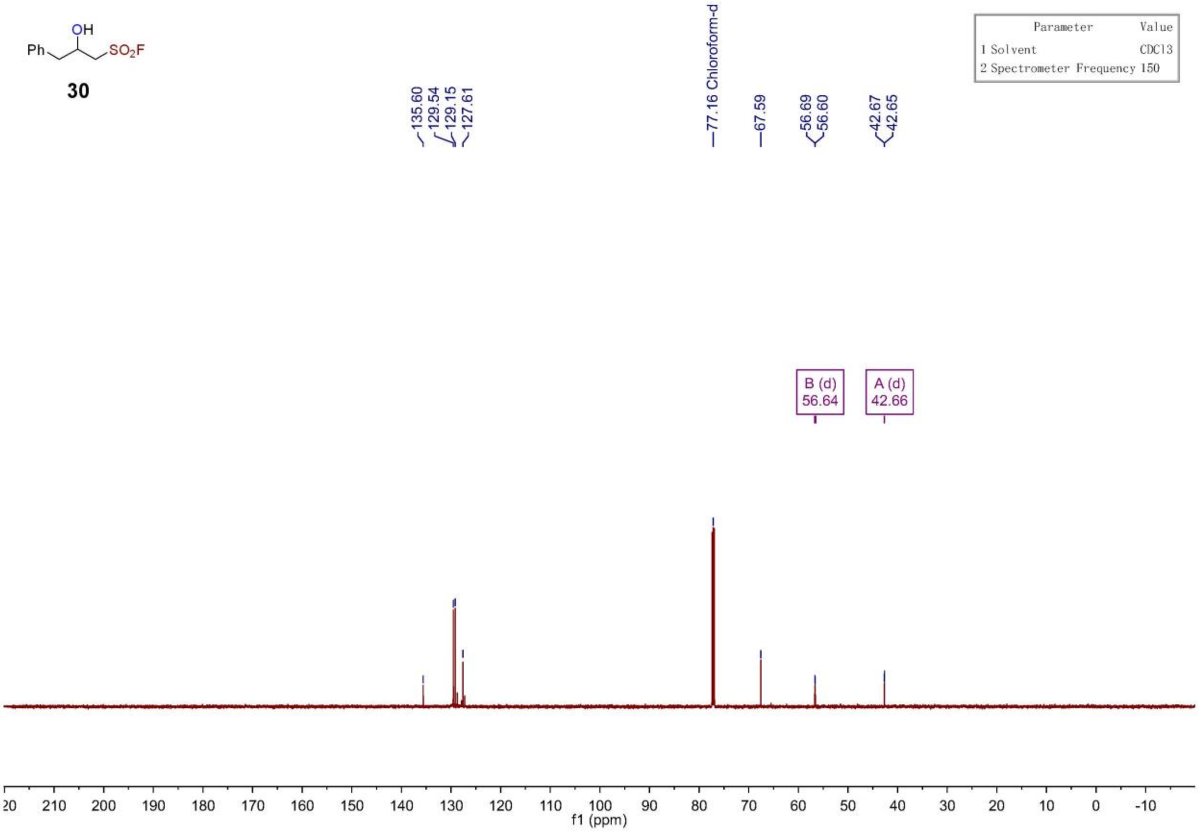

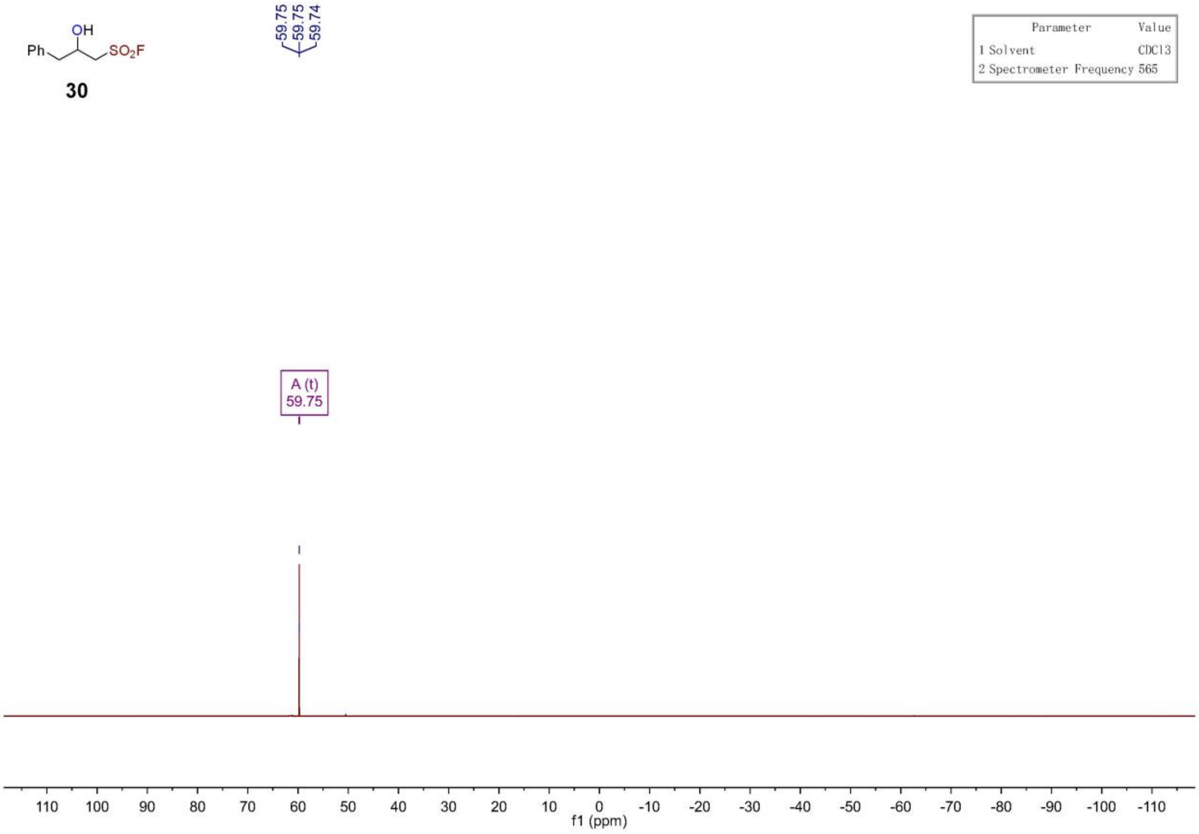

Supplementary Figure 49. ^{1}H, ^{13}C and ^{19}F NMR spectra of **31**.

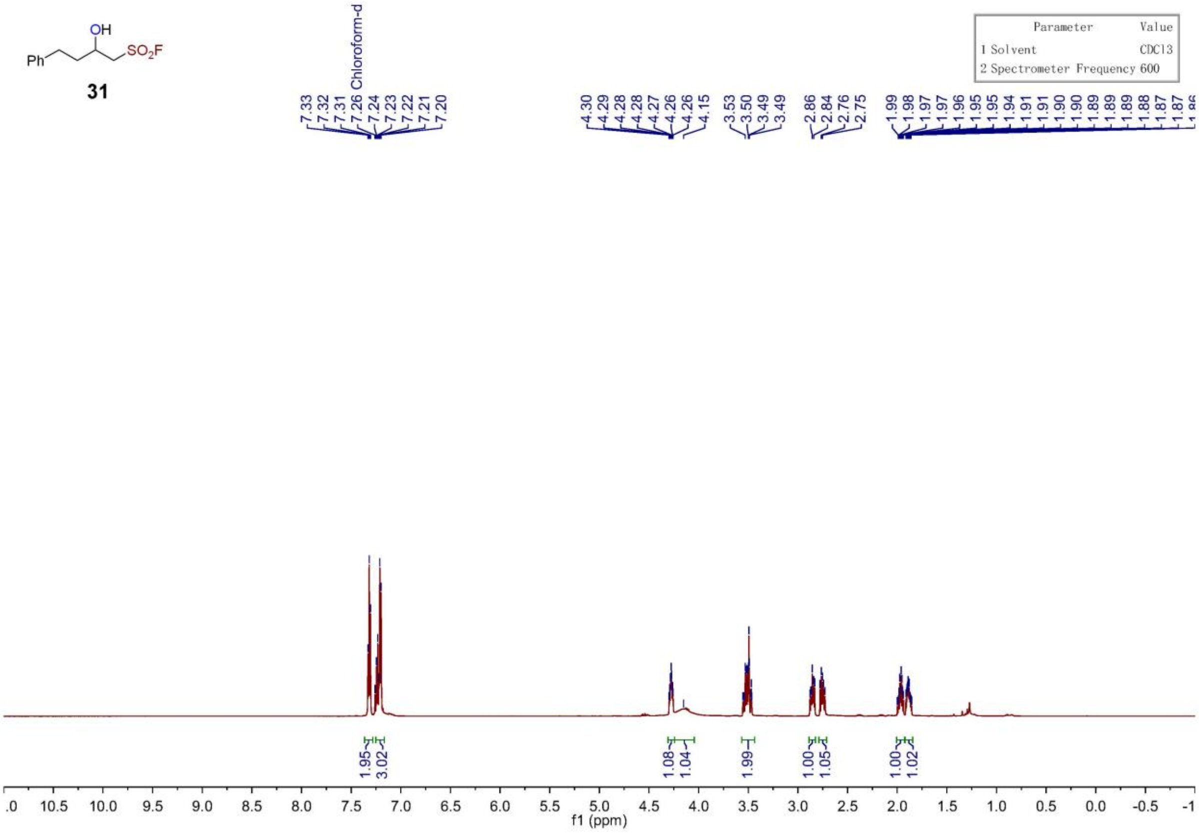

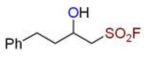

31

Parameter	Value
1 Solvent	CDCl3
2 Spectrometer Frequency	150

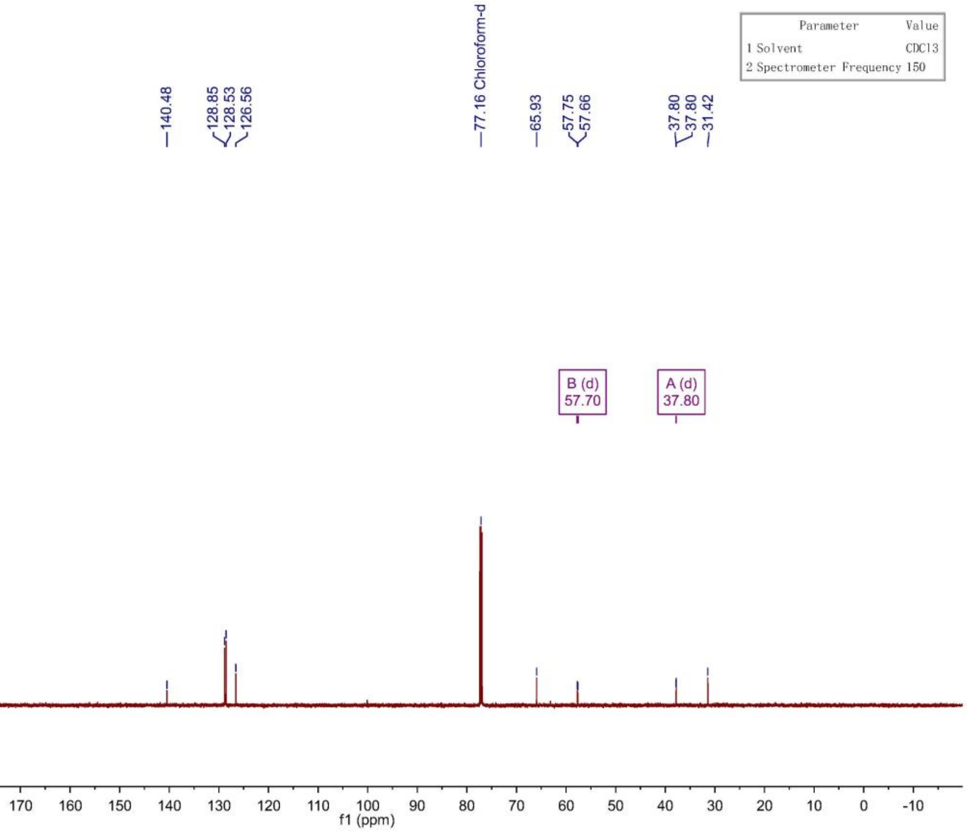

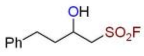

31

Parameter	Value
1 Solvent	CDCl3
2 Spectrometer Frequency	565

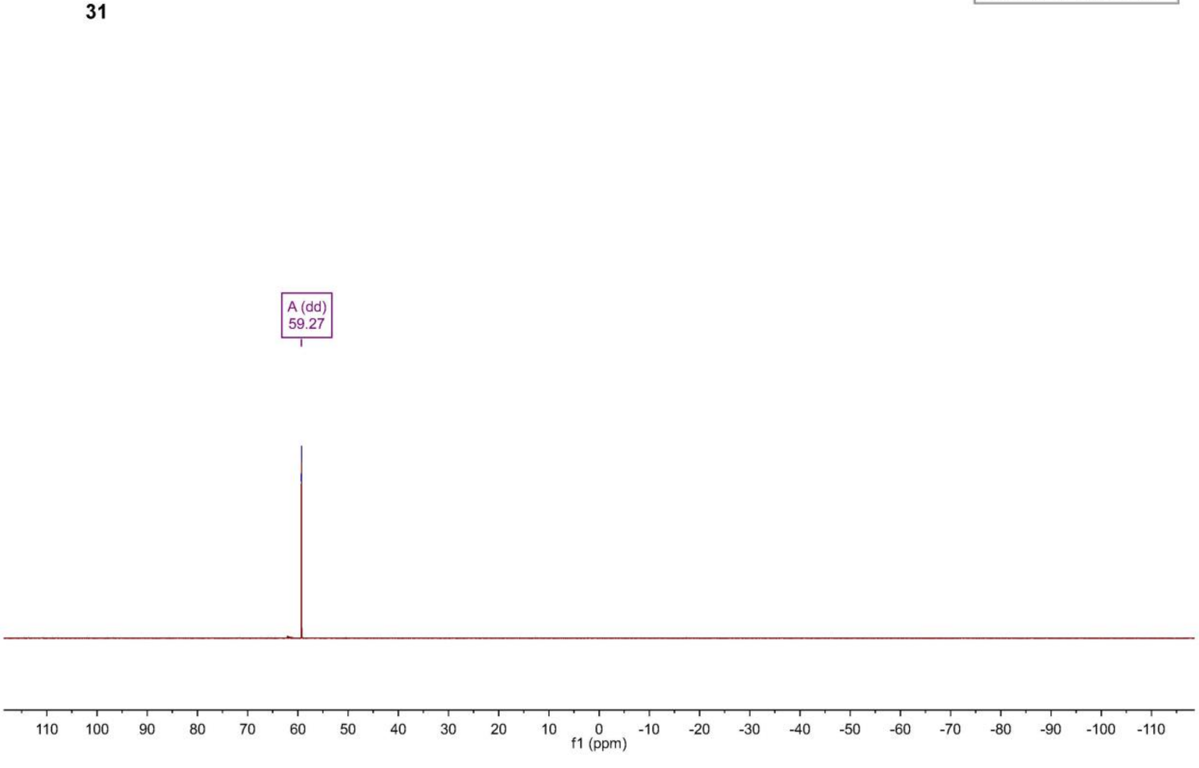

Supplementary Figure 50. ^{1}H, ^{13}C and ^{19}F NMR spectra of **32**.

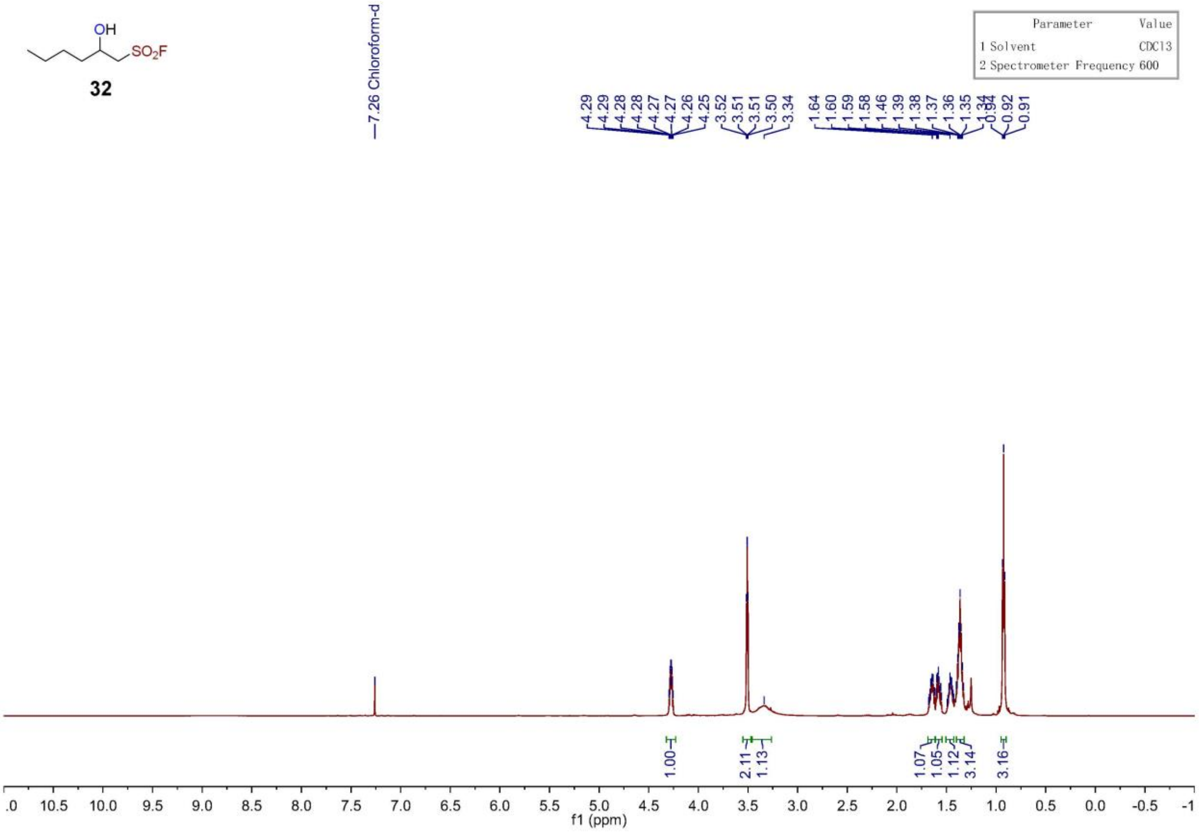

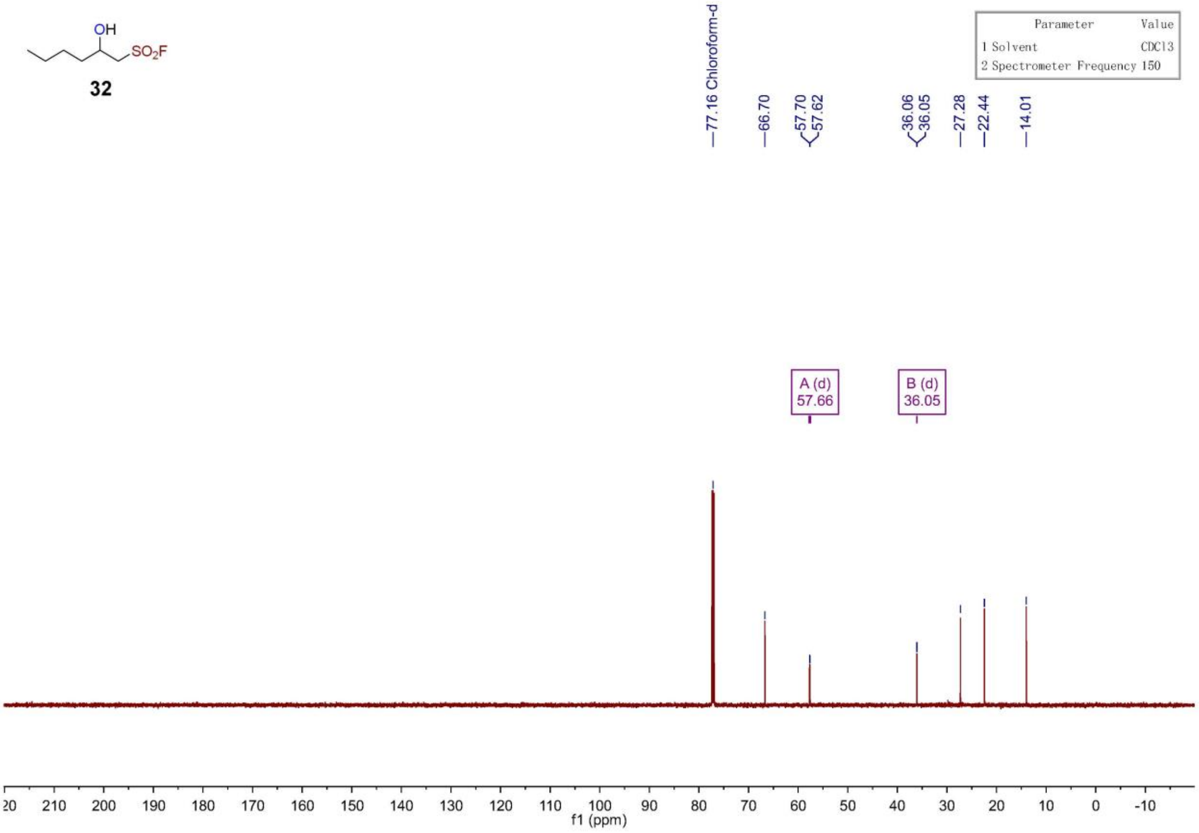

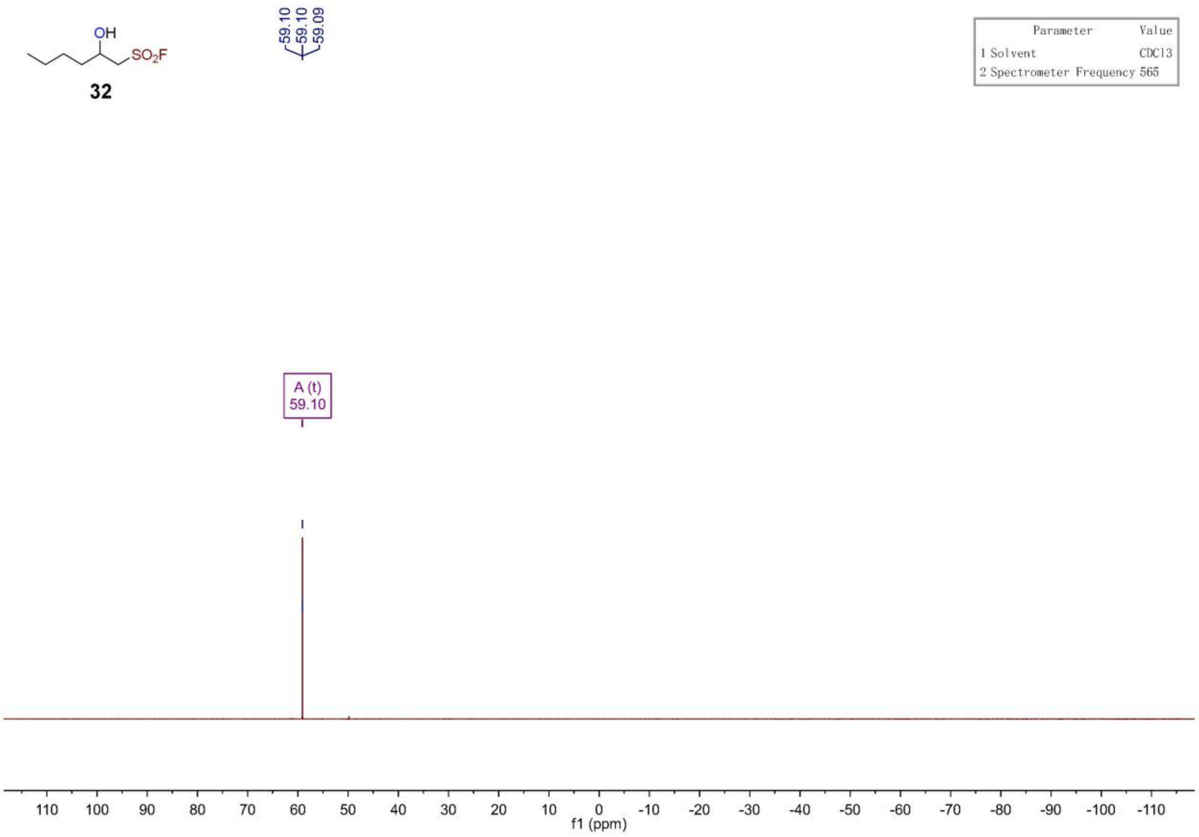

Supplementary Figure 51. ^{1}H, ^{13}C and ^{19}F NMR spectra of **33**.

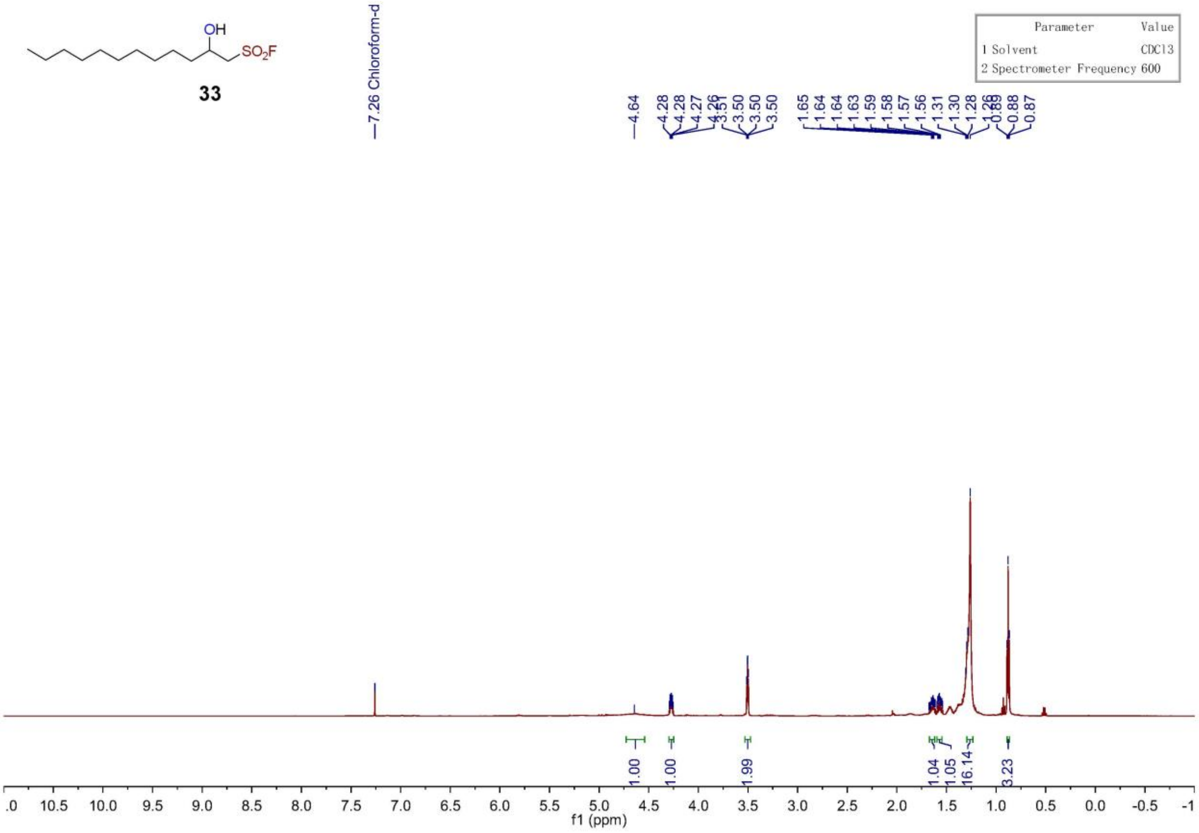

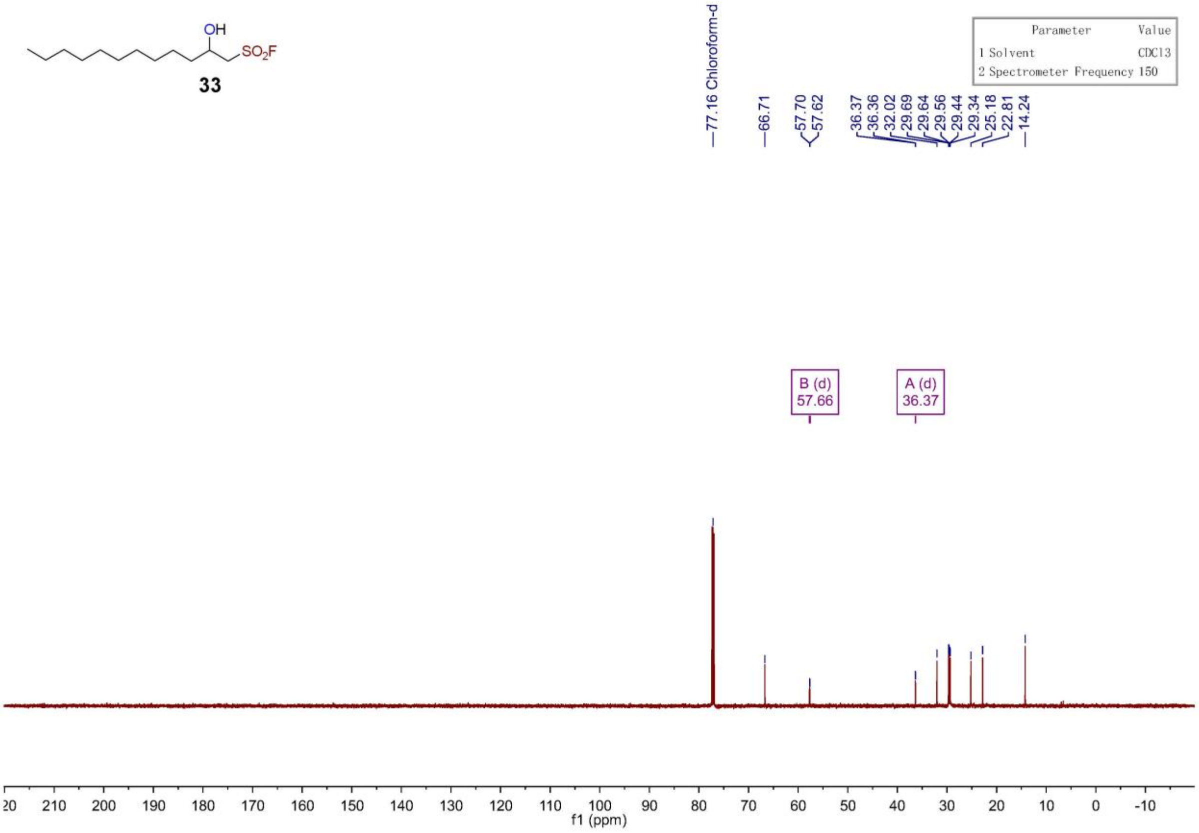
OH
SO_2F
33
77.16 Chloroform-d
66.71
57.70
57.62
36.37
36.36
32.02
29.69
29.64
29.56
29.44
29.34
25.18
22.81
14.24
Parameter Value
1 Solvent CDCl3
2 Spectrometer Frequency 150
B (d) 57.66
A (d) 36.37
f1 (ppm)

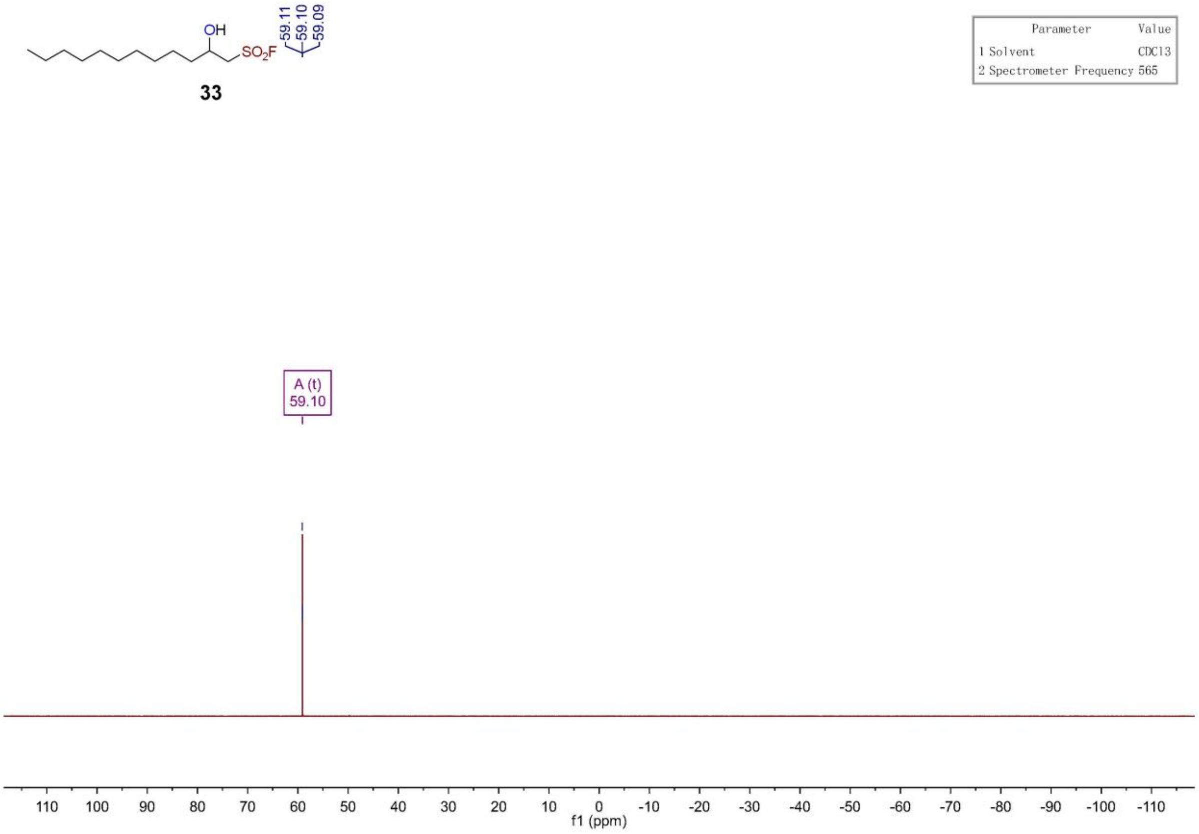
OH
SO_2F
33
59.11
59.10
59.09
Parameter Value
1 Solvent CDCl3
2 Spectrometer Frequency 565
A (t) 59.10
f1 (ppm)

Supplementary Figure 52. ^{1}H, ^{13}C and ^{19}F NMR spectra of **34**.

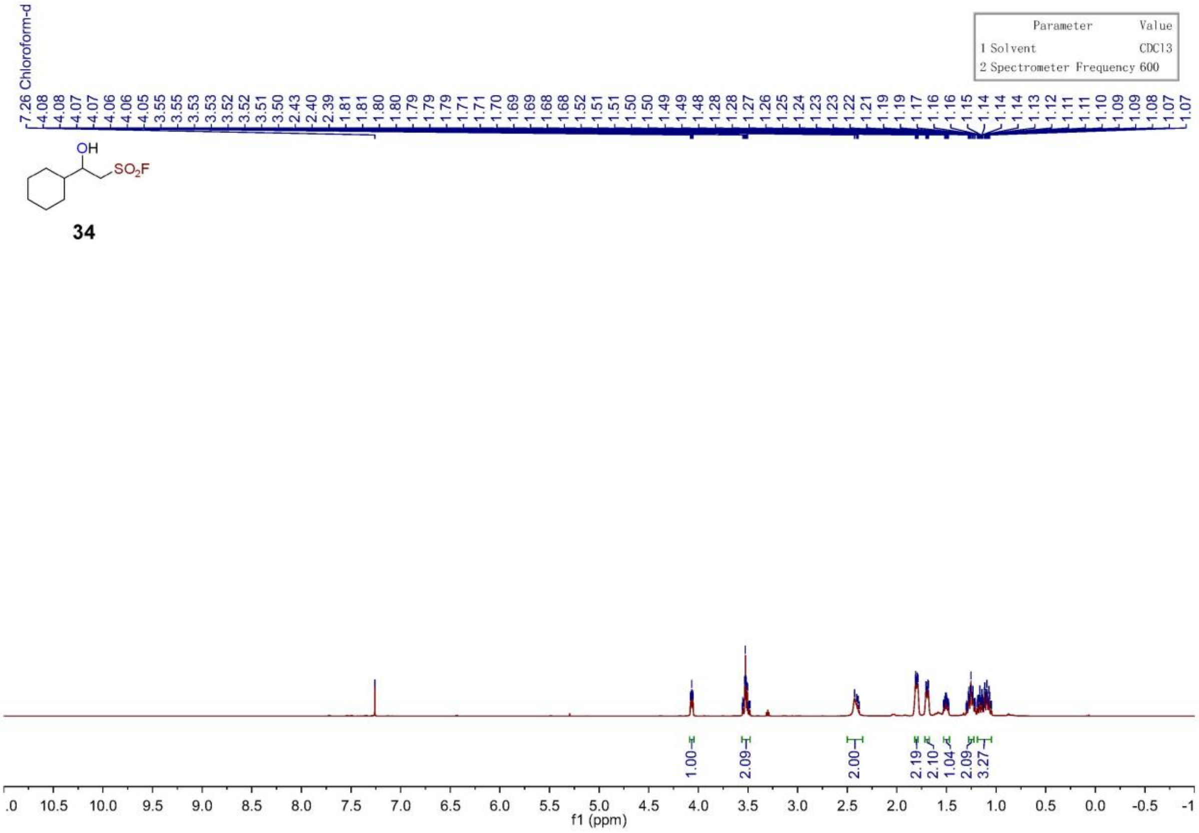

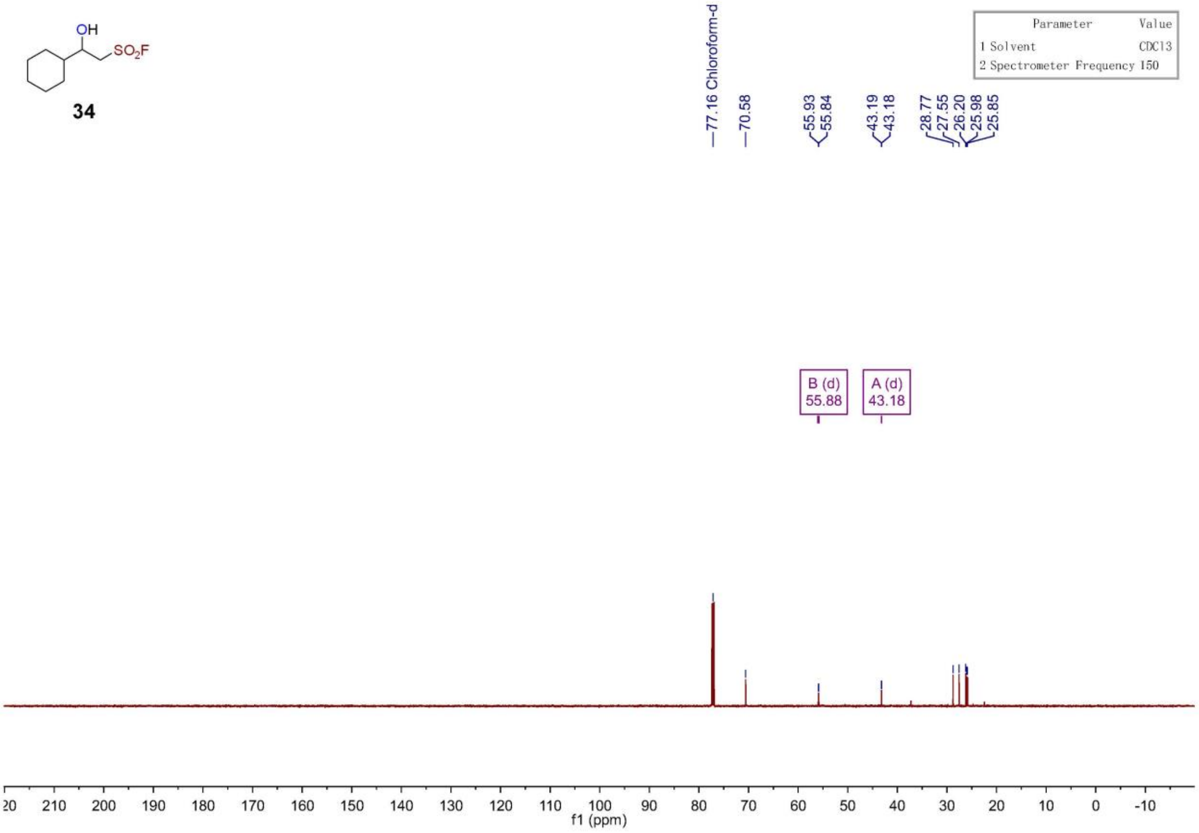

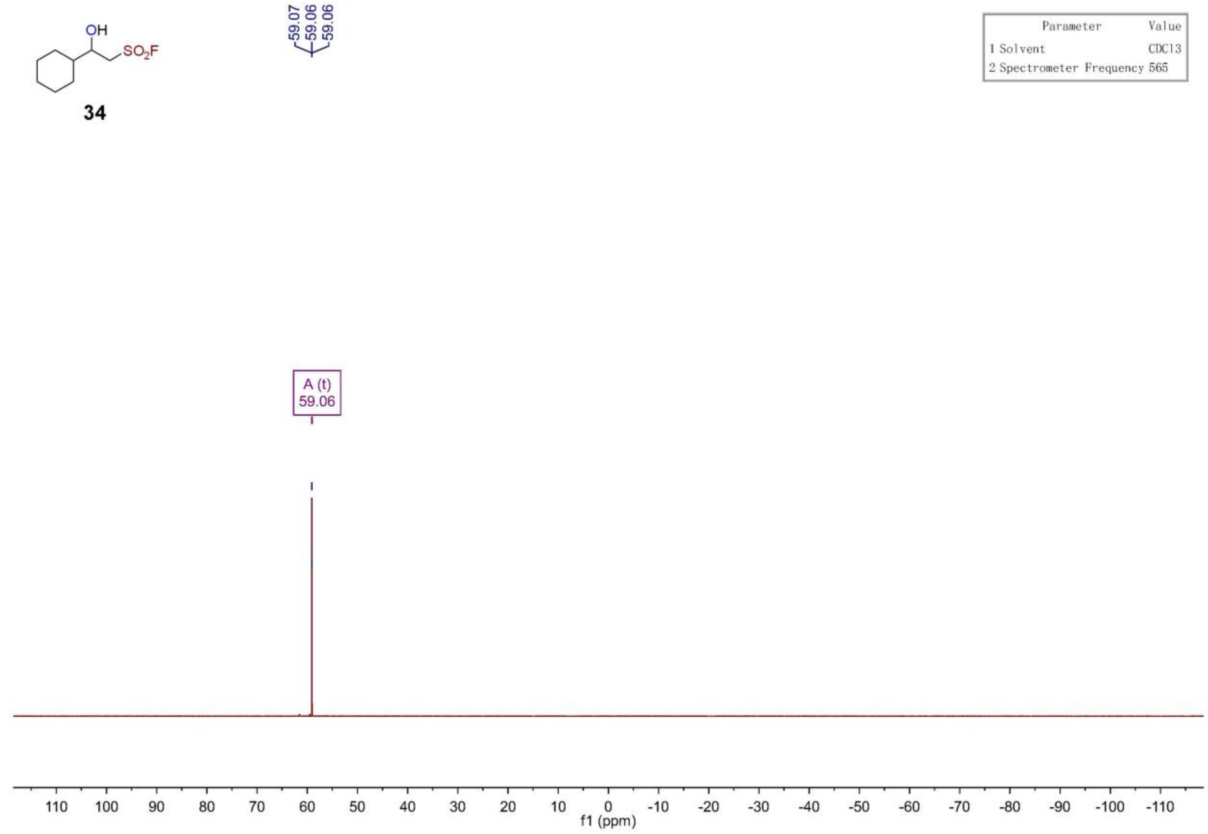

Supplementary Figure 53. ^{1}H, ^{13}C and ^{19}F NMR spectra of **35**.

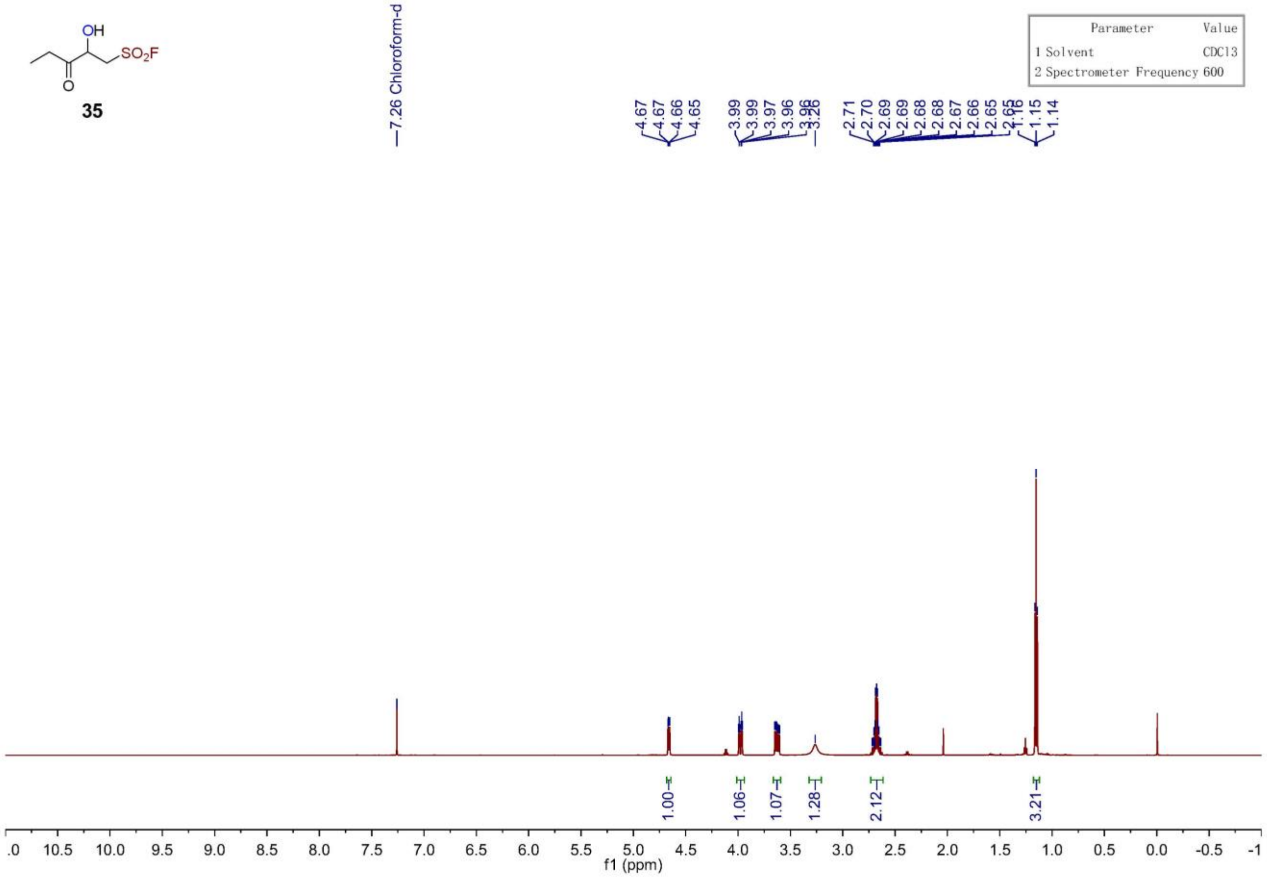

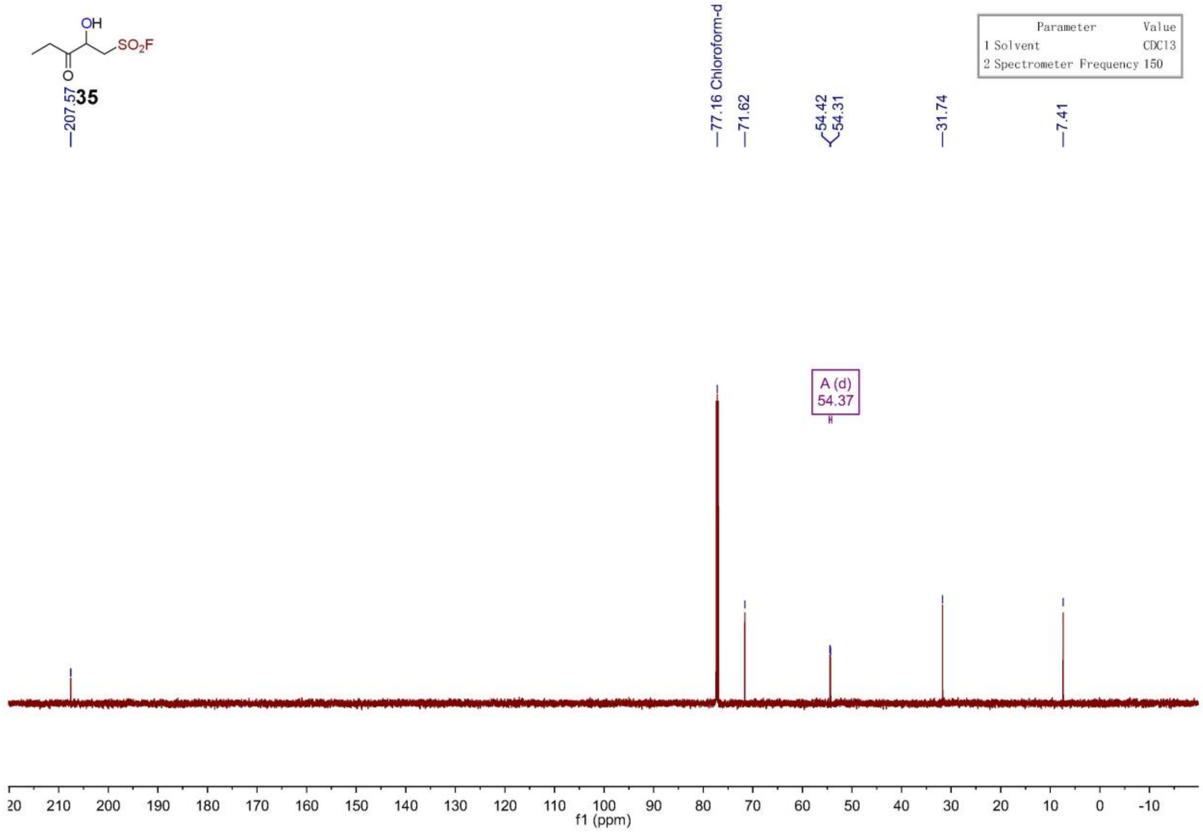
OH
SO2F
O
35
207.57
77.16 Chloroform-d
71.62
54.42
54.31
31.74
7.41
Parameter Value
1 Solvent CDCl3
2 Spectrometer Frequency 150
A (d)
54.37
f1 (ppm)

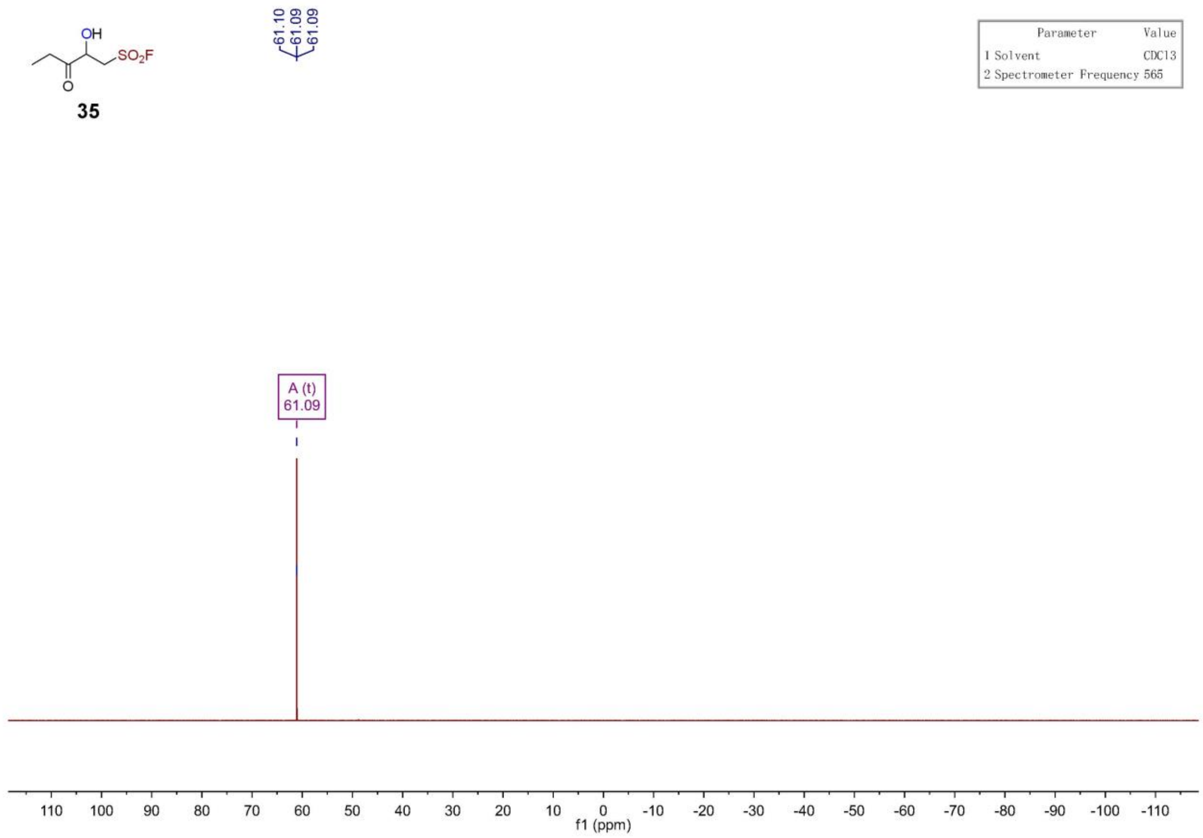
OH
SO2F
O
35
61.10
61.09
61.09
Parameter Value
1 Solvent CDCl3
2 Spectrometer Frequency 565
A (t)
61.09
f1 (ppm)

Supplementary Figure 54. ^{1}H, ^{13}C and ^{19}F NMR spectra of **36**.

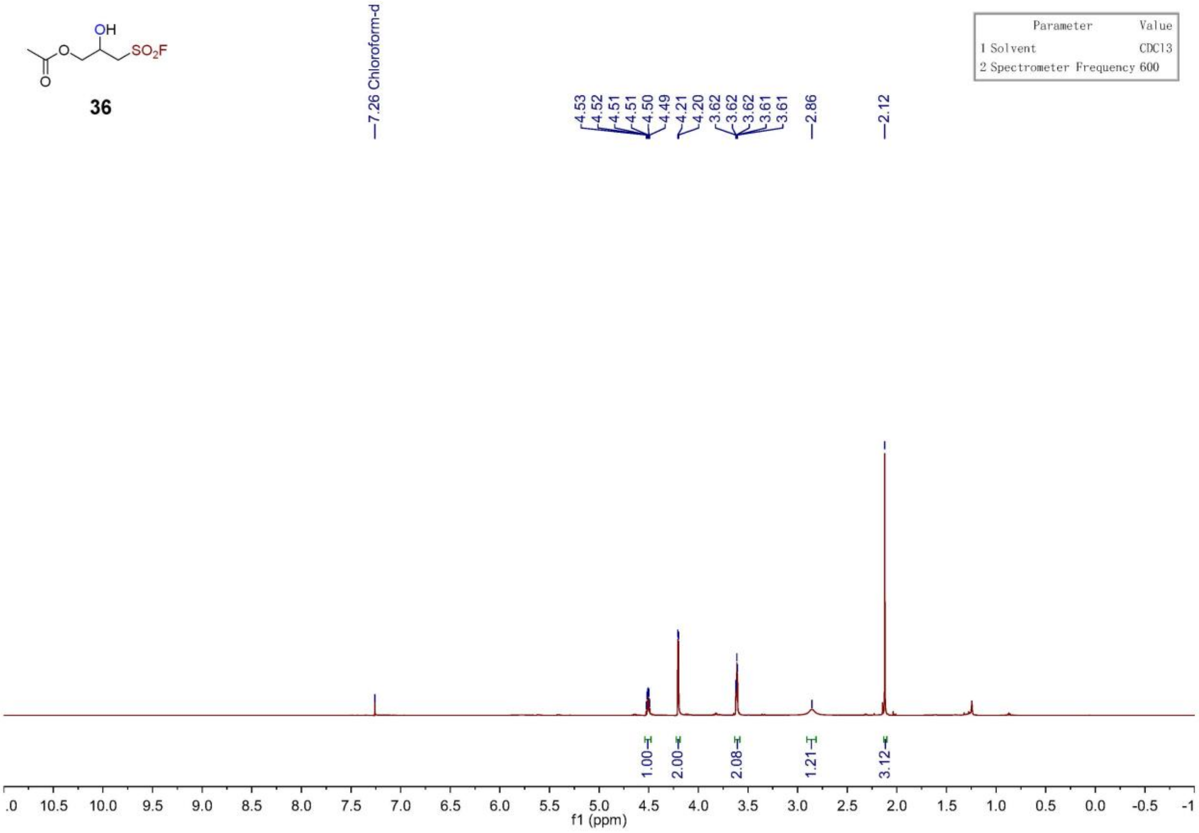

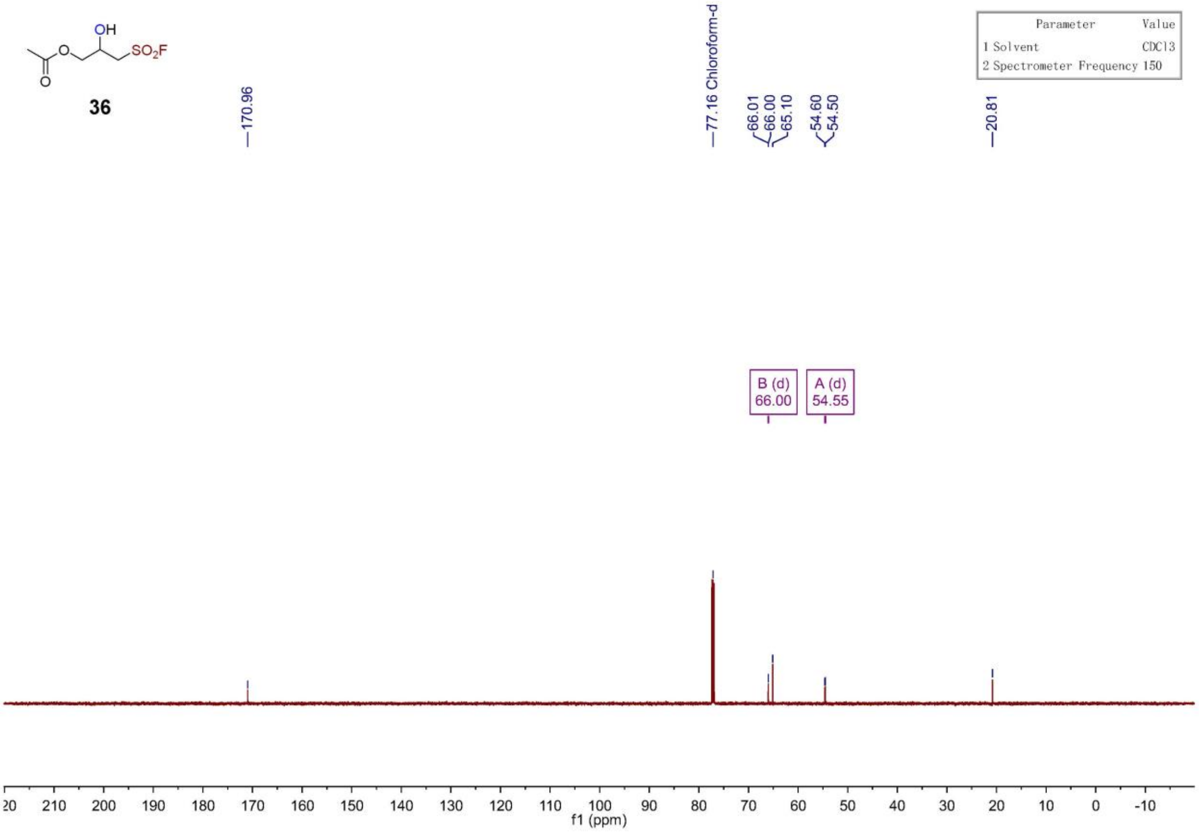

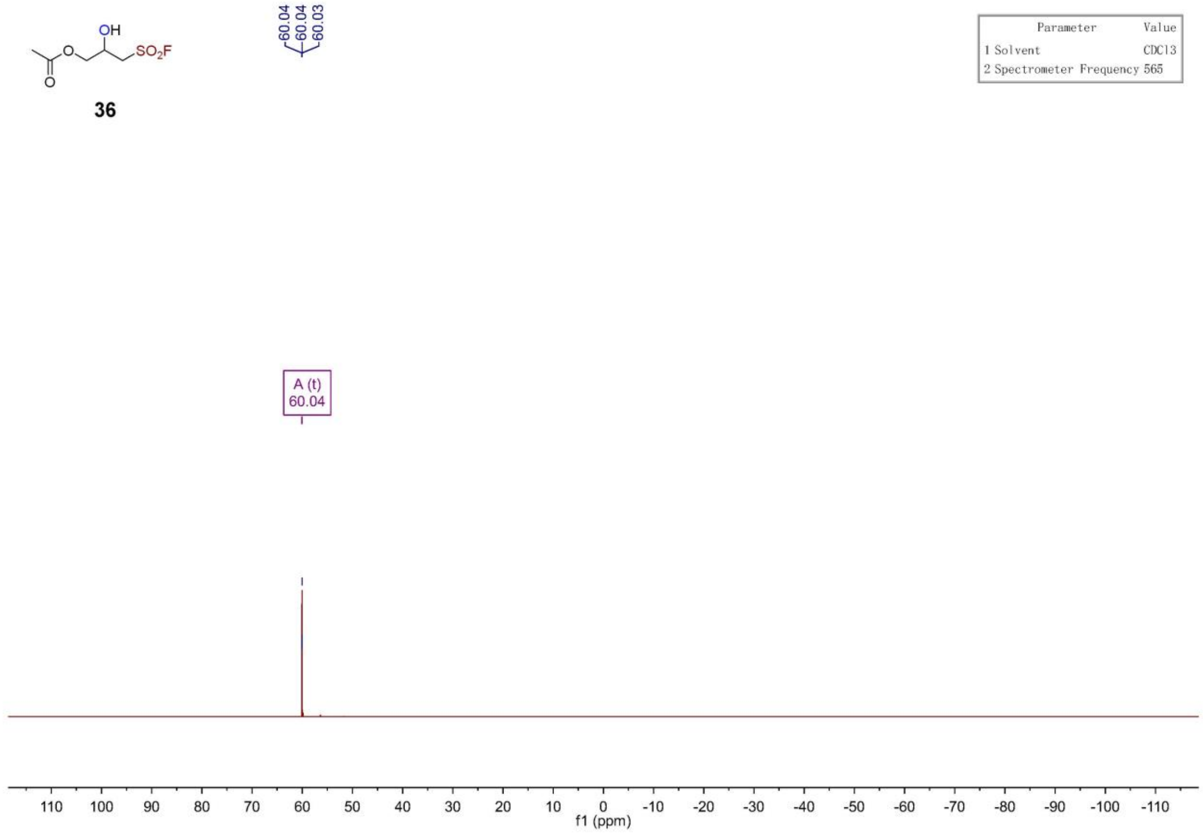

Supplementary Figure 55. ^{1}H, ^{13}C and ^{19}F NMR spectra of **37**.

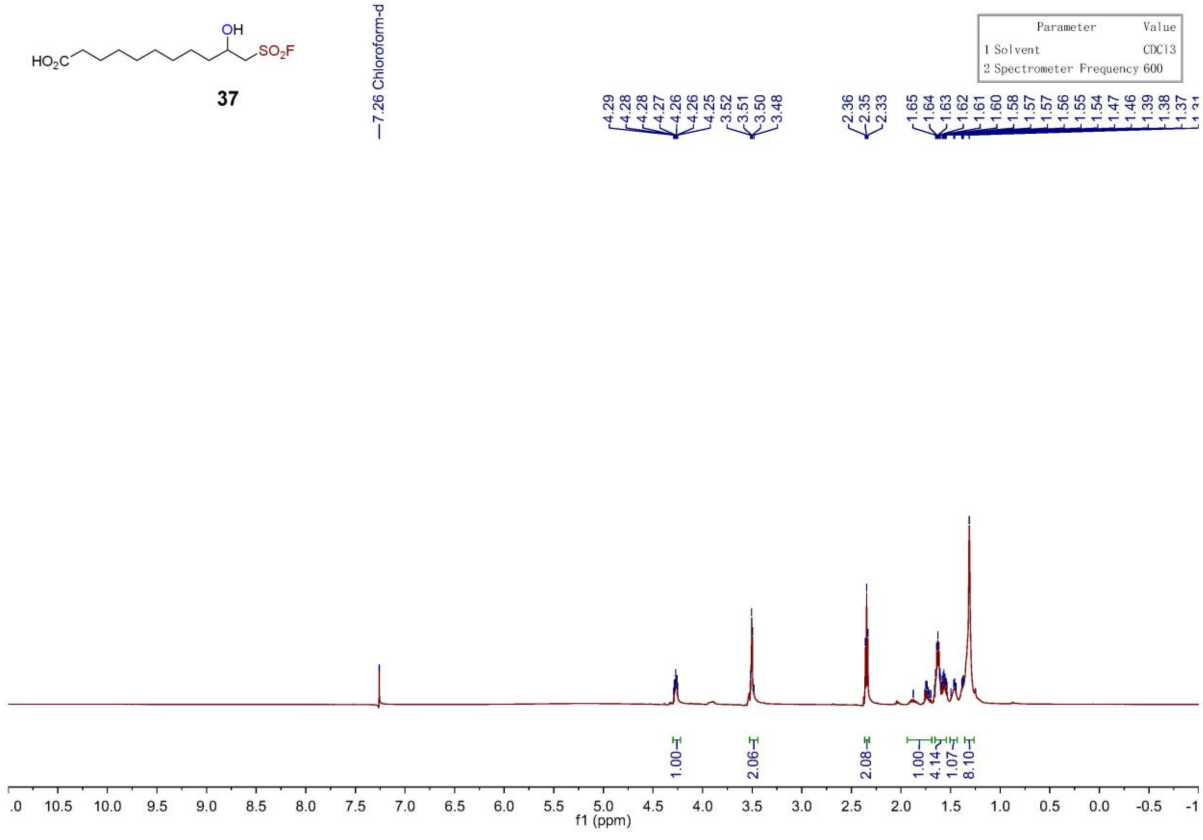

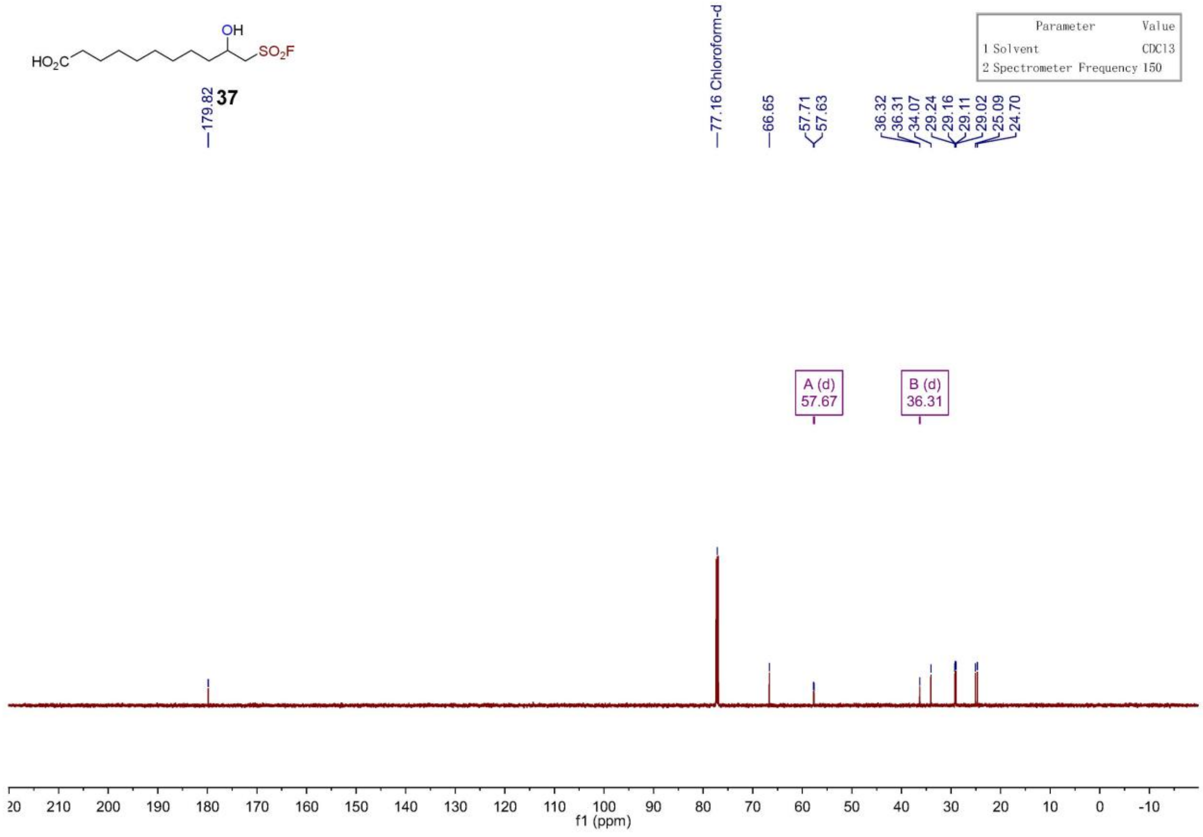
OH
HO2C
SO2F
37
179.82
77.16 Chloroform-d
66.65
57.71
57.63
36.32
36.31
34.07
29.24
29.16
29.11
29.02
25.09
24.70
Parameter Value
1 Solvent CDCl3
2 Spectrometer Frequency 150
A (d)
57.67
B (d)
36.31
f1 (ppm)

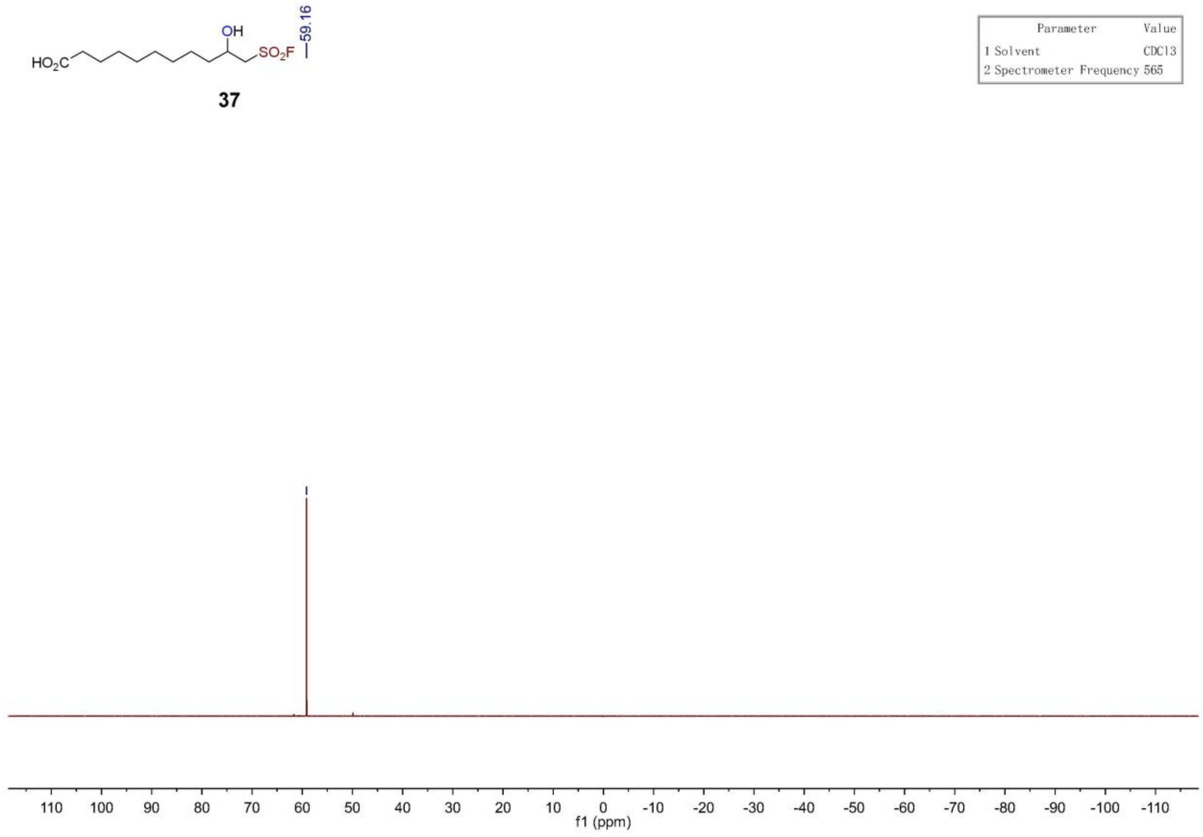
OH
HO2C
SO2F
37
59.16
Parameter Value
1 Solvent CDCl3
2 Spectrometer Frequency 565
f1 (ppm)

Supplementary Figure 56. ^{1}H, ^{13}C and ^{19}F NMR spectra of **38**.

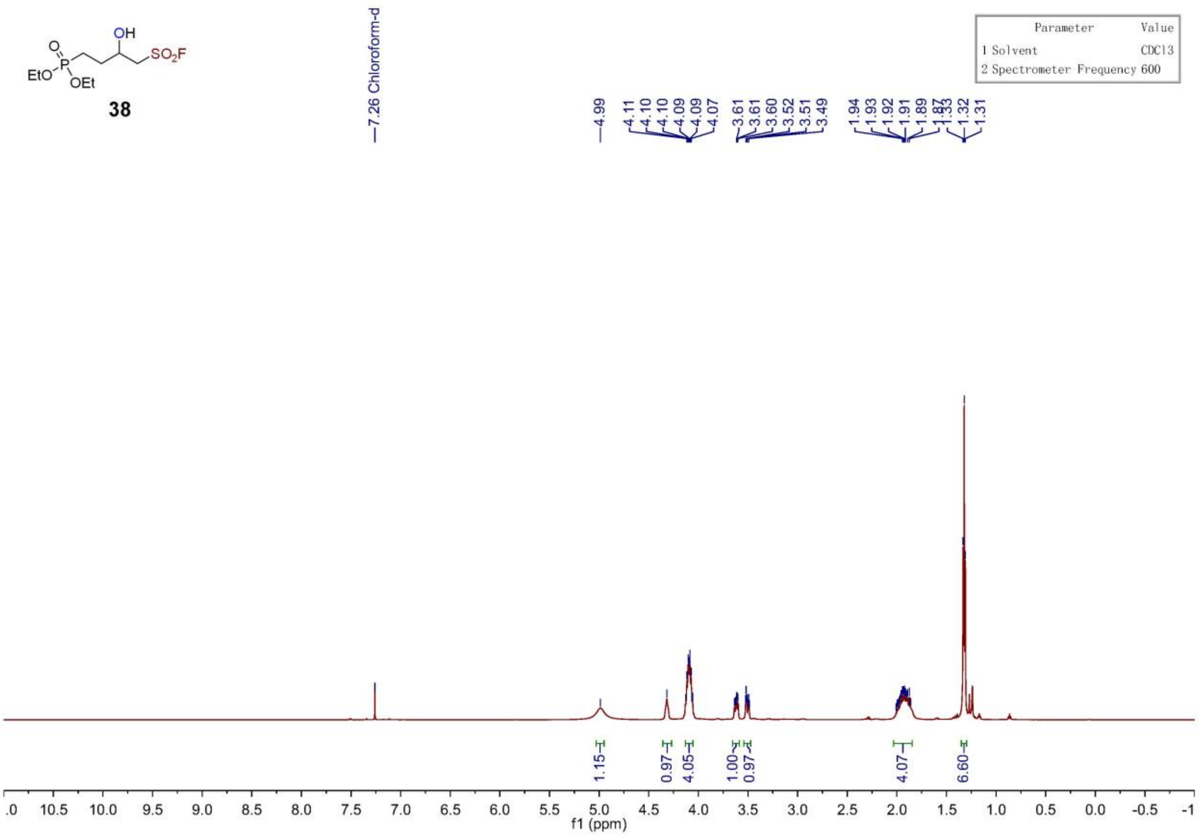

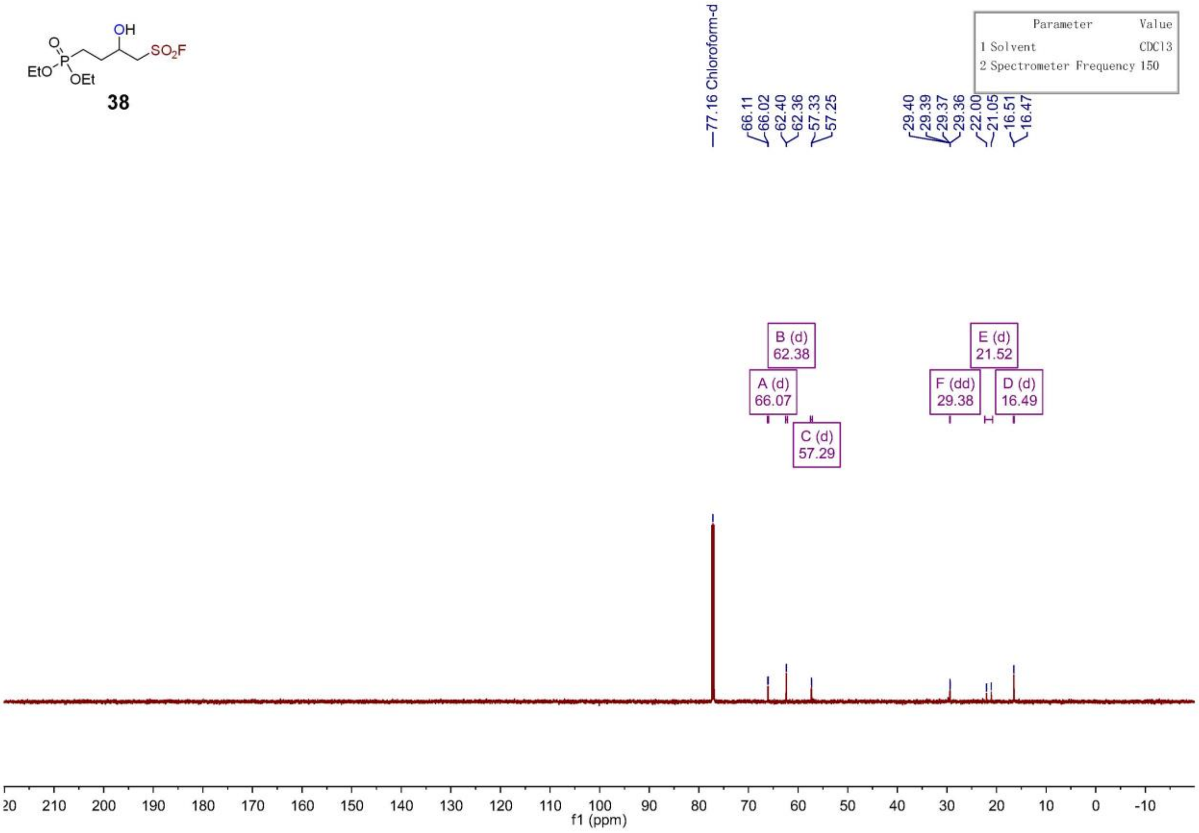

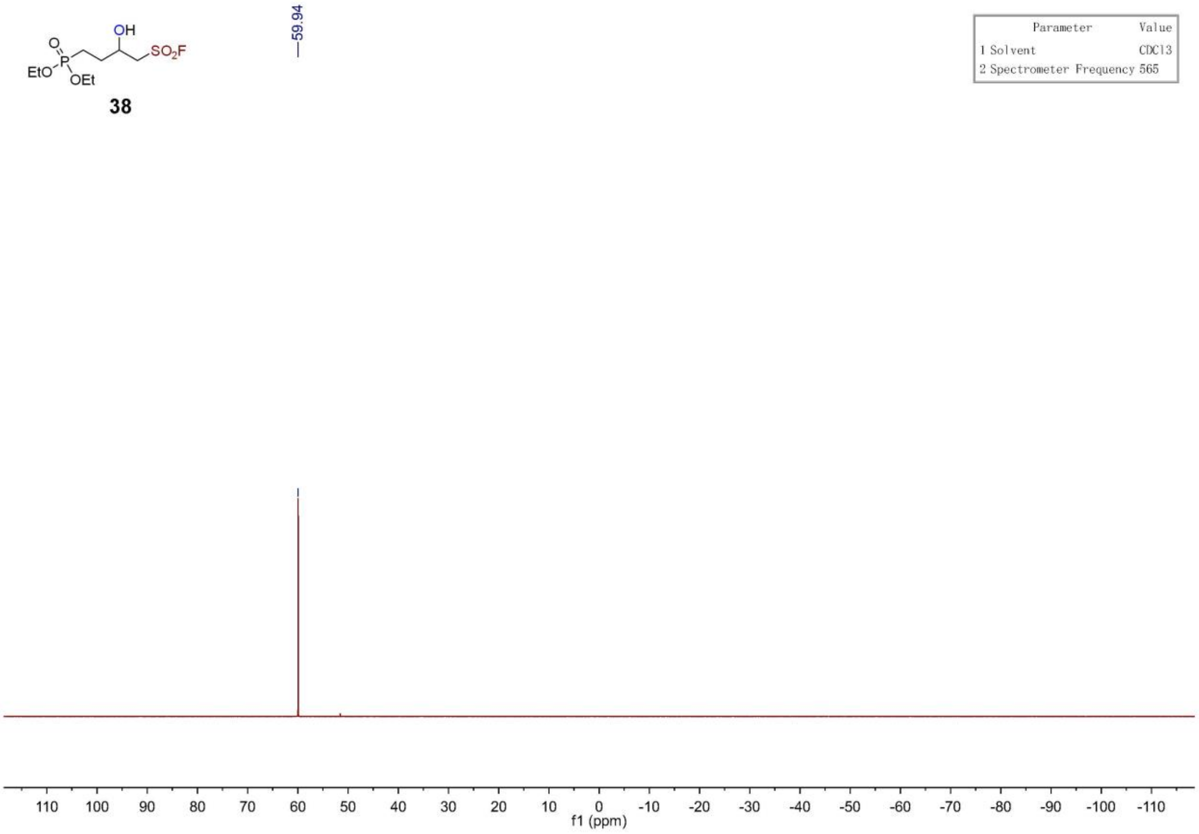

Supplementary Figure 57. ^{1}H, ^{13}C and ^{19}F NMR spectra of **39**.

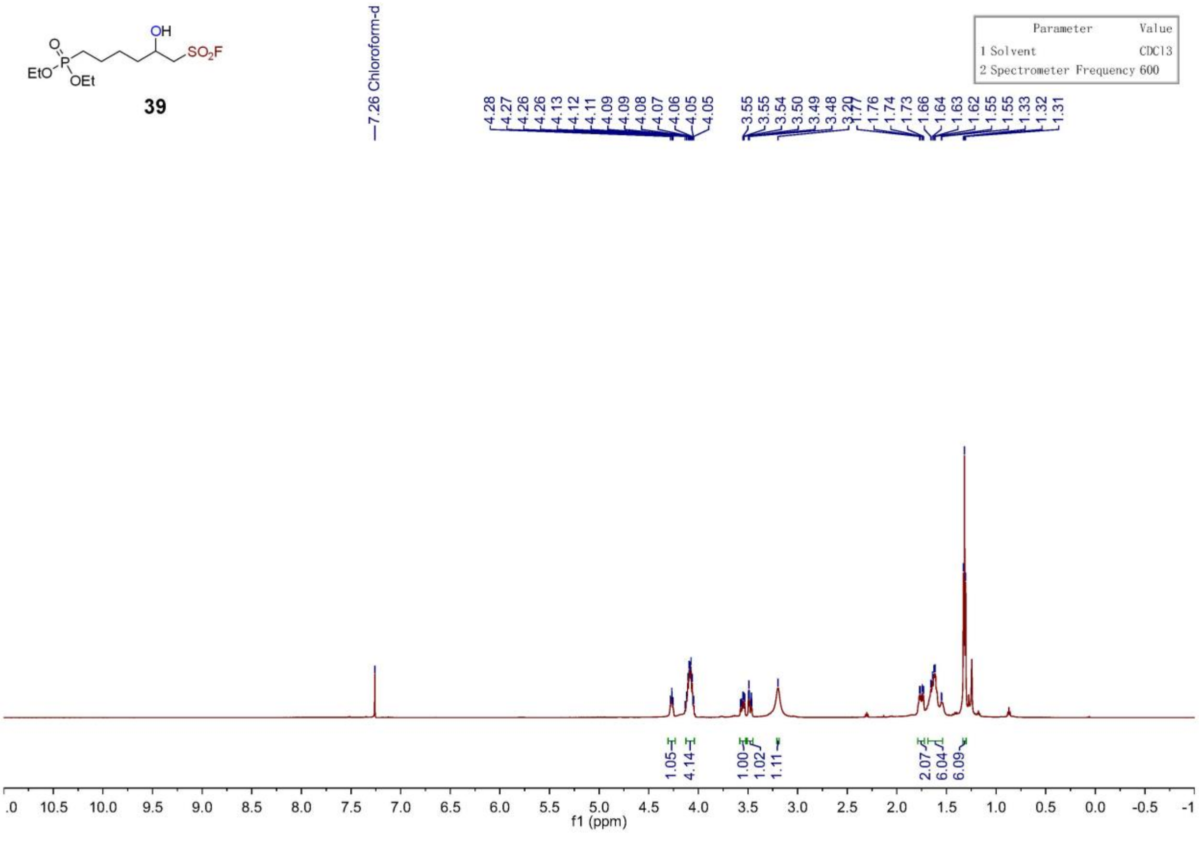

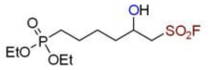

39

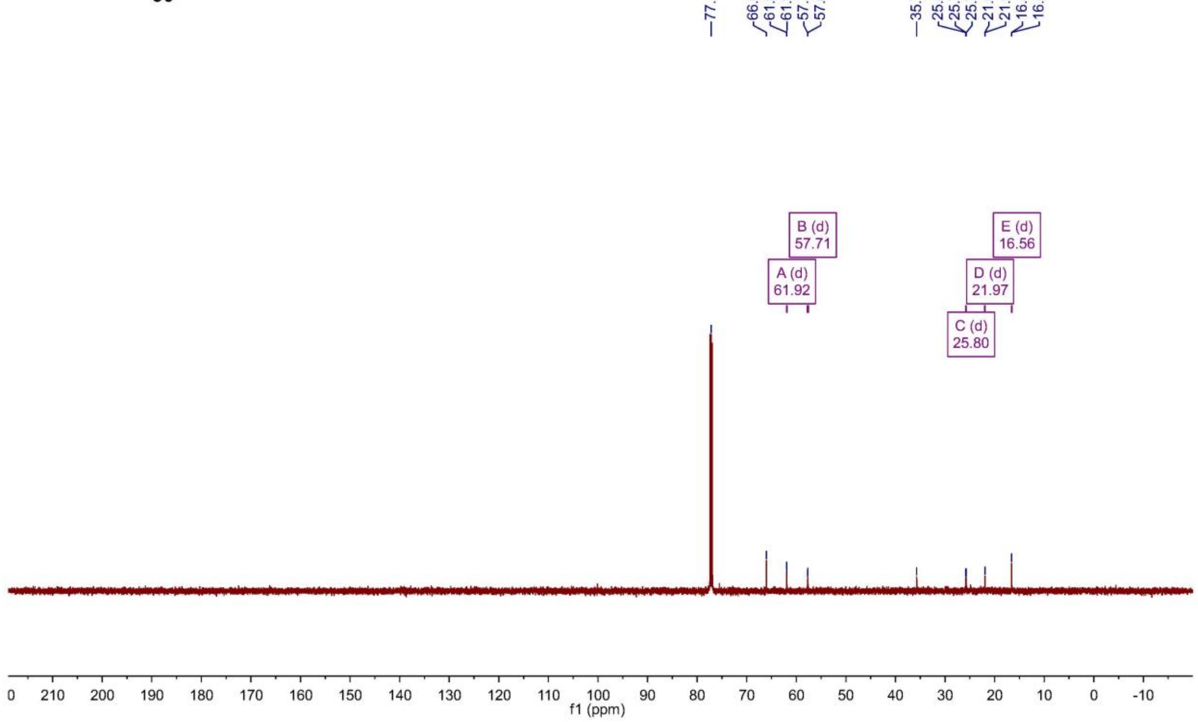

77.16 Chloroform-d
66.04
61.95
61.90
57.75
57.67
35.72
25.81
25.79
25.71
21.98
21.95
16.58
16.54
Parameter Value
1 Solvent CDCl3
2 Spectrometer Frequency 150
B (d) 57.71
A (d) 61.92
E (d) 16.56
D (d) 21.97
C (d) 25.80
0 210 200 190 180 170 160 150 140 130 120 110 100 90 80 70 60 50 40 30 20 10 0 -10
f1 (ppm)

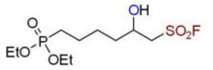

39

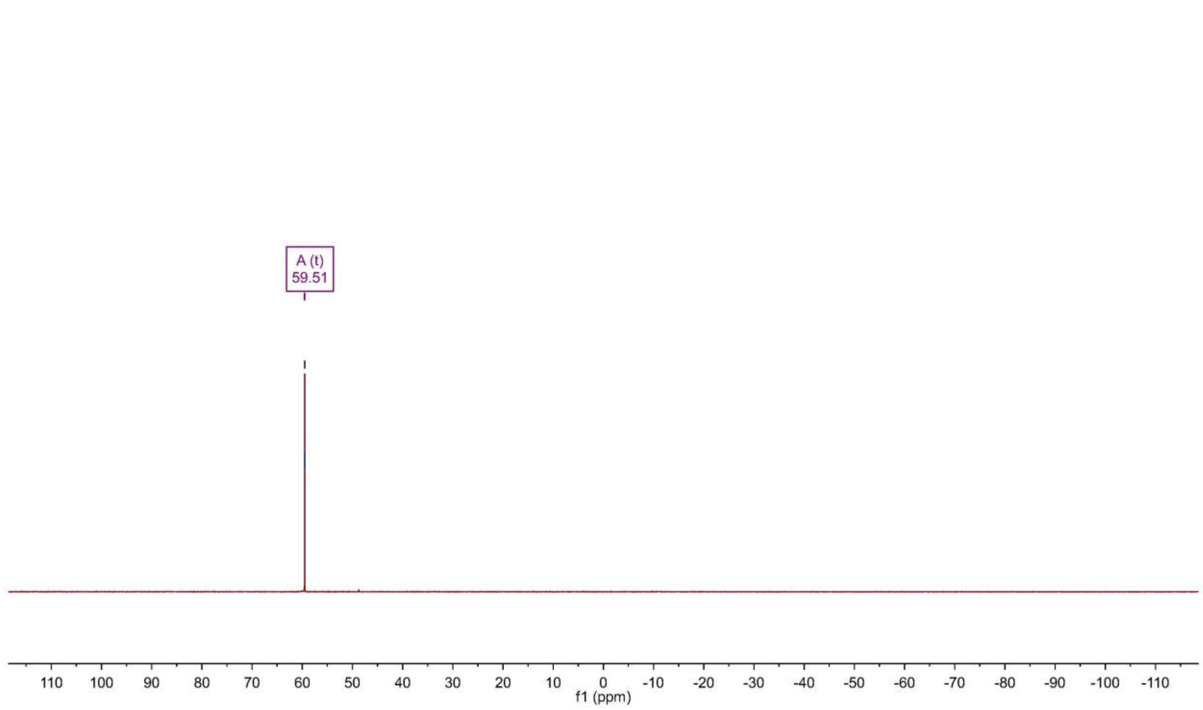

59.52
59.51
59.51
Parameter Value
1 Solvent CDCl3
2 Spectrometer Frequency 565
A (t) 59.51
110 100 90 80 70 60 50 40 30 20 10 0 -10 -20 -30 -40 -50 -60 -70 -80 -90 -100 -110
f1 (ppm)

Supplementary Figure 58. ^{1}H, ^{13}C and ^{19}F NMR spectra of **40**.

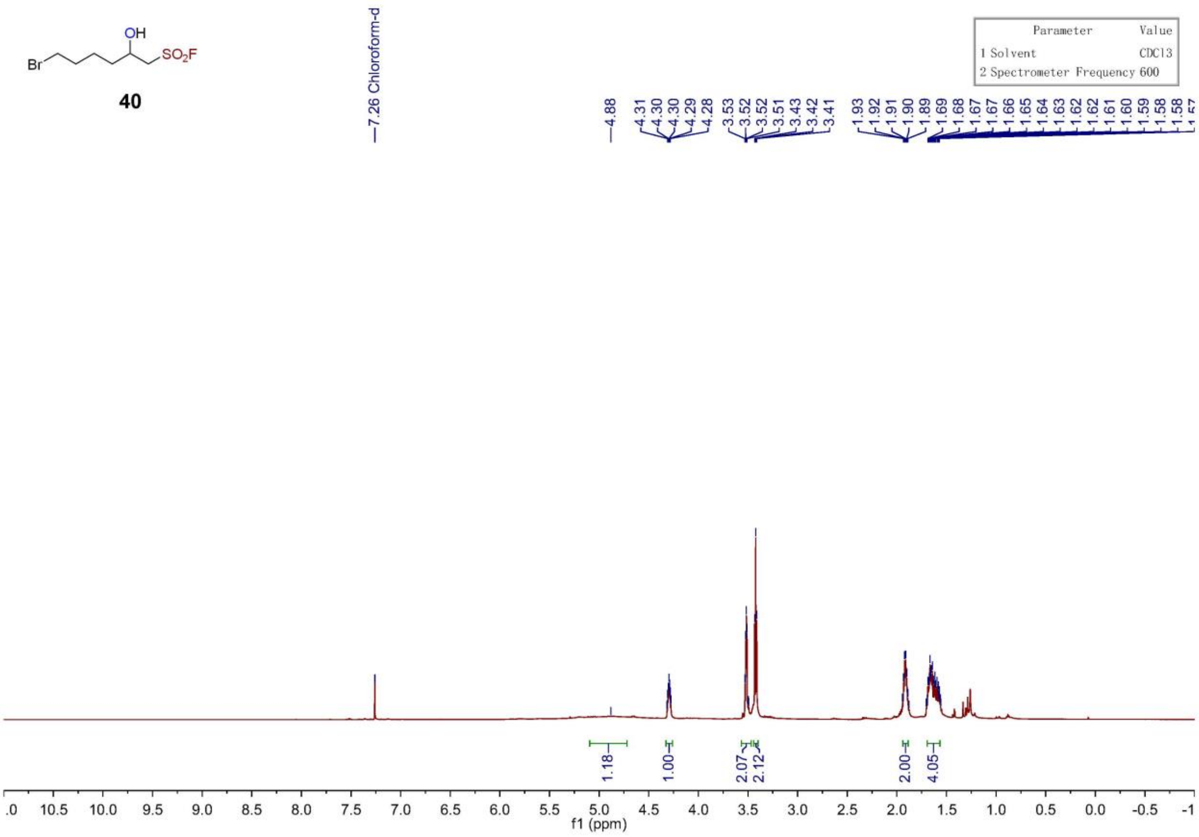

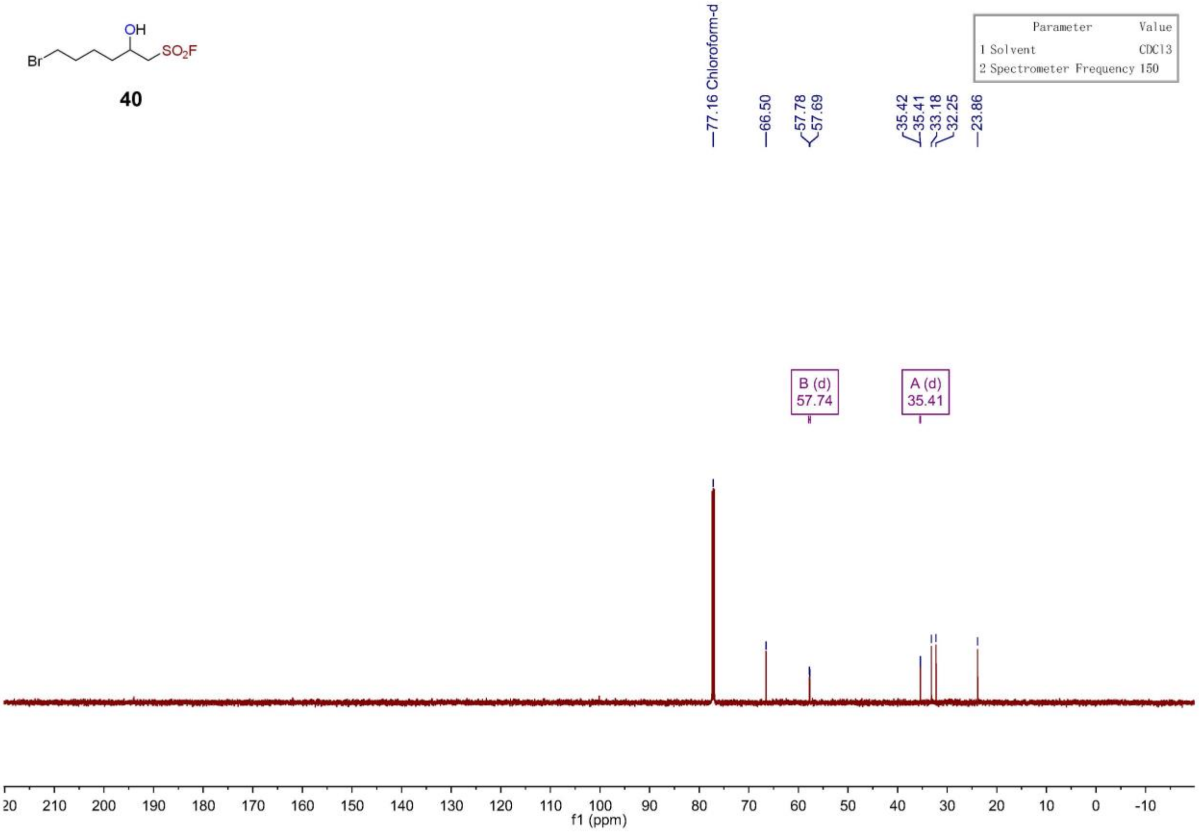

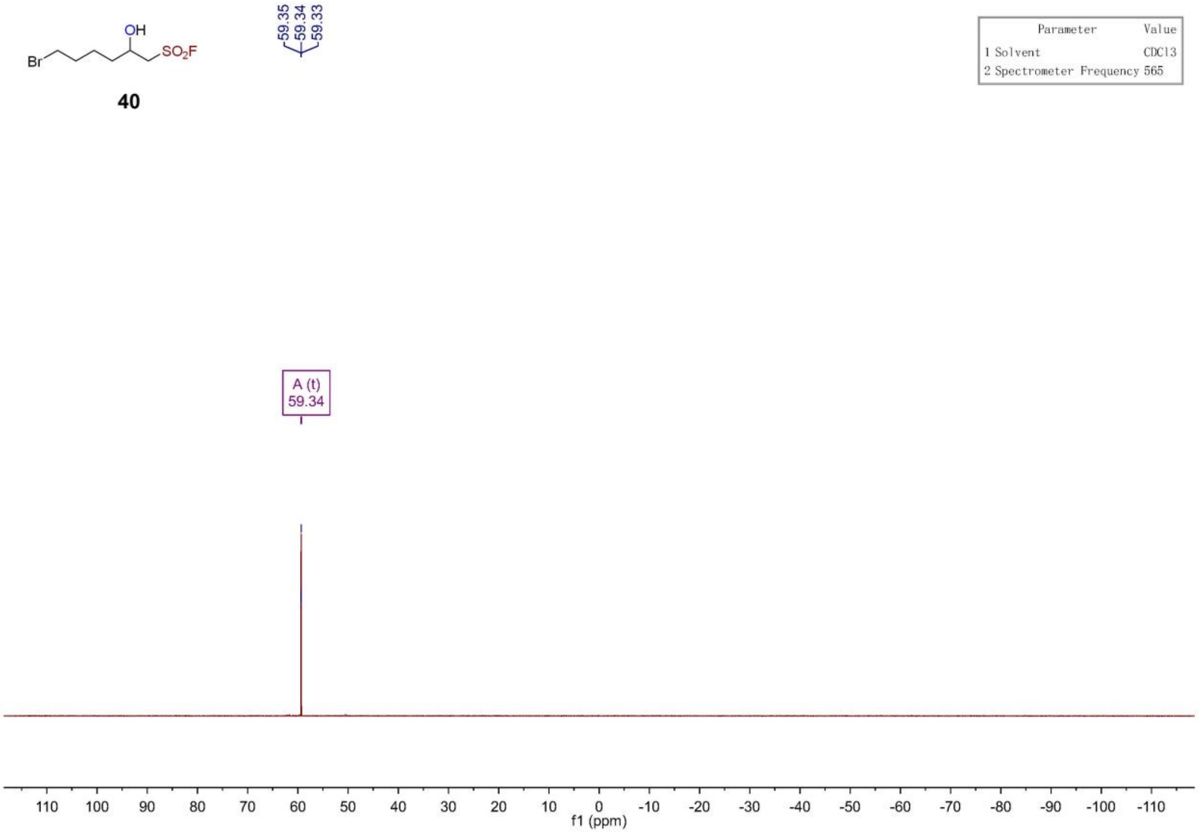

Supplementary Figure 59. ^{1}H, ^{13}C and ^{19}F NMR spectra of **41**.

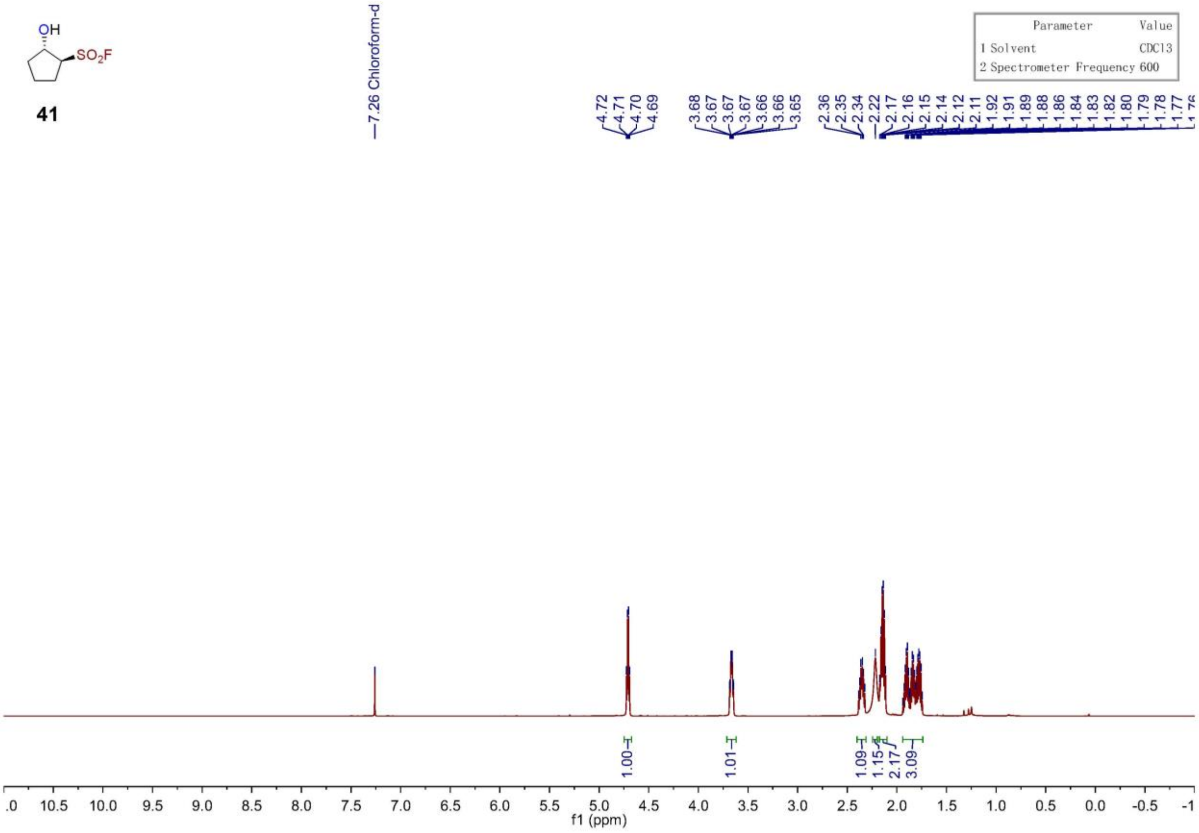

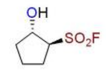

41

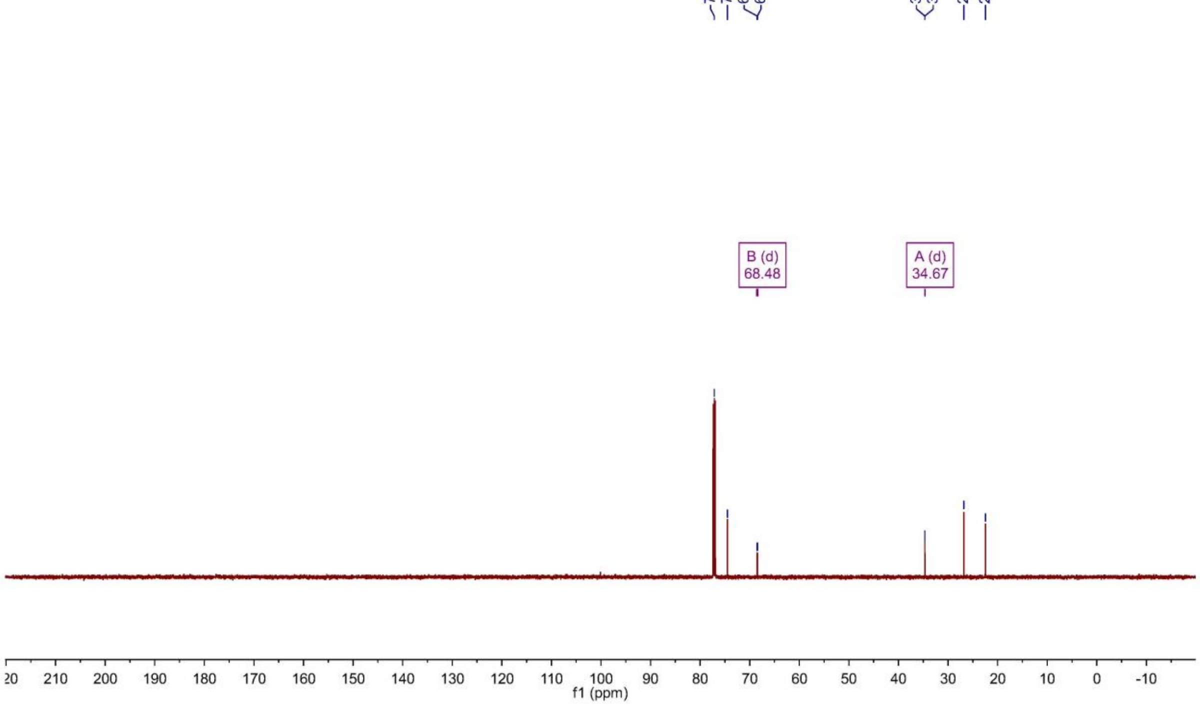

Parameter	Value
1 Solvent	CDCl3
2 Spectrometer Frequency	150

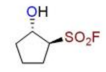

41

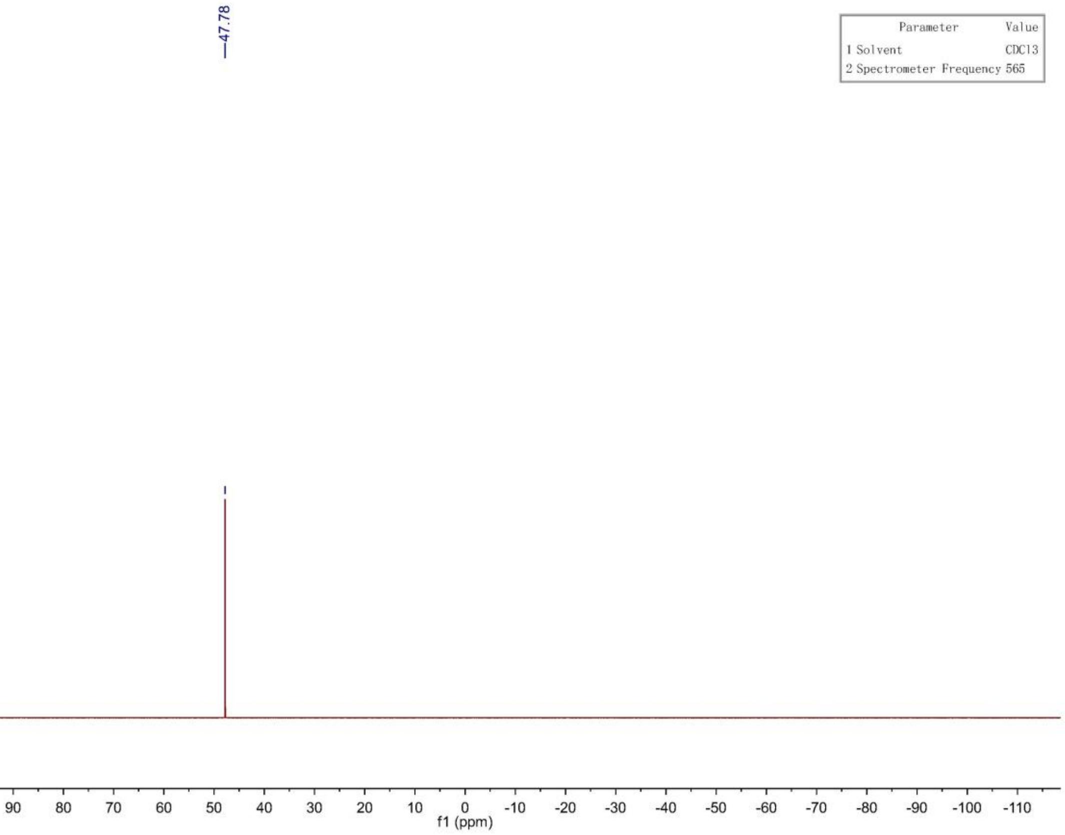

Parameter	Value
1 Solvent	CDCl3
2 Spectrometer Frequency	565

Supplementary Figure 60. ^{1}H, ^{13}C and ^{19}F NMR spectra of **42**.

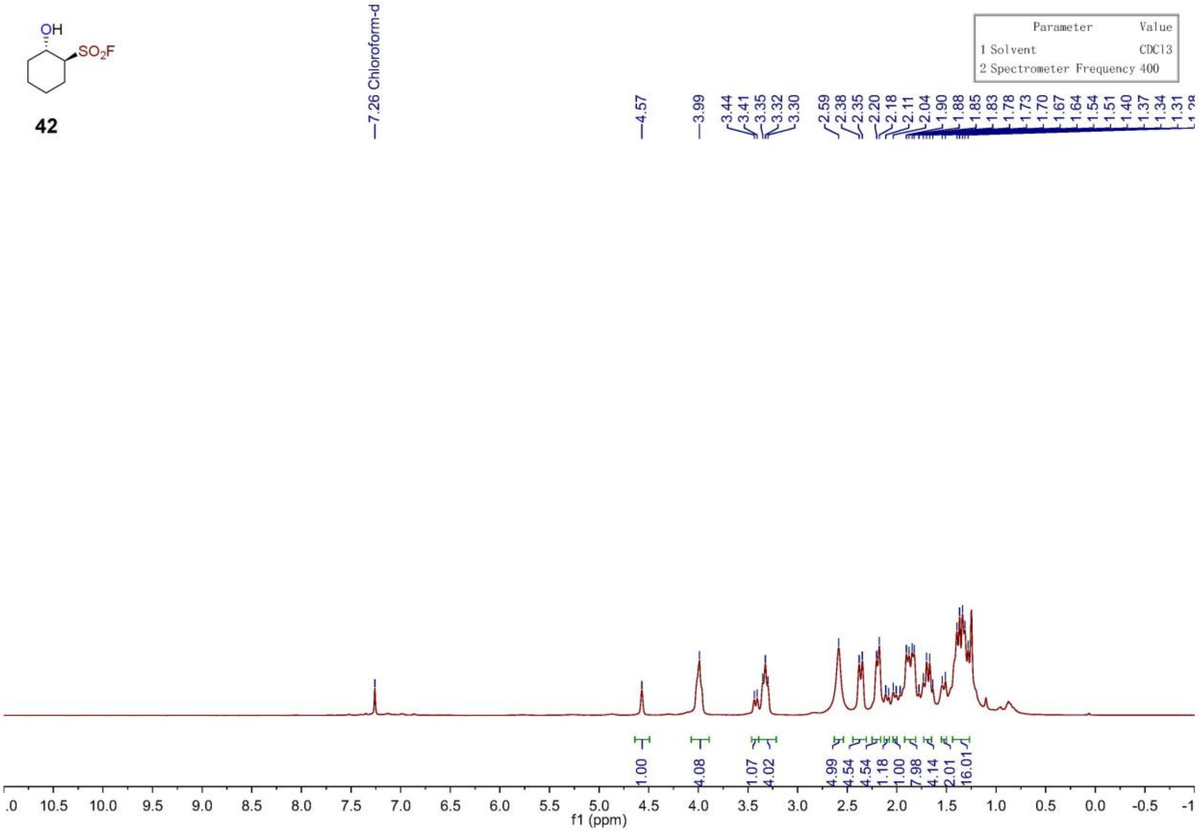

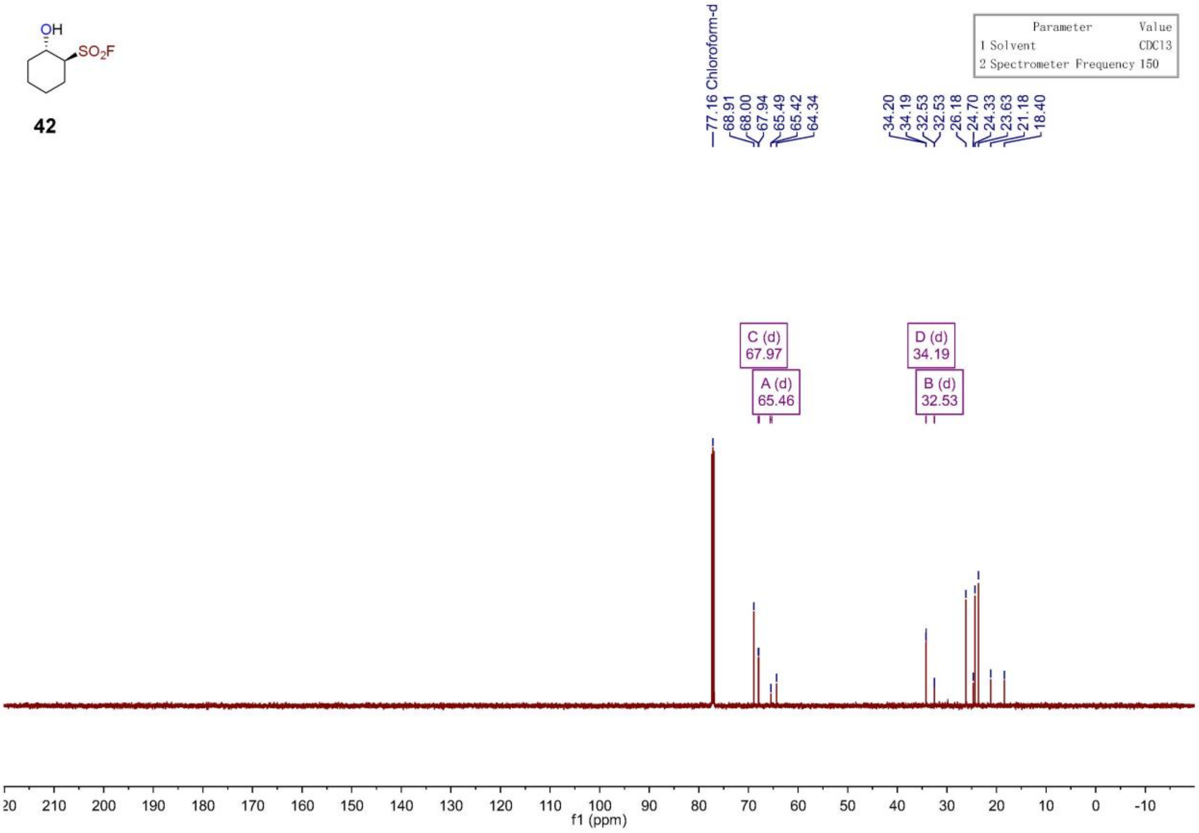

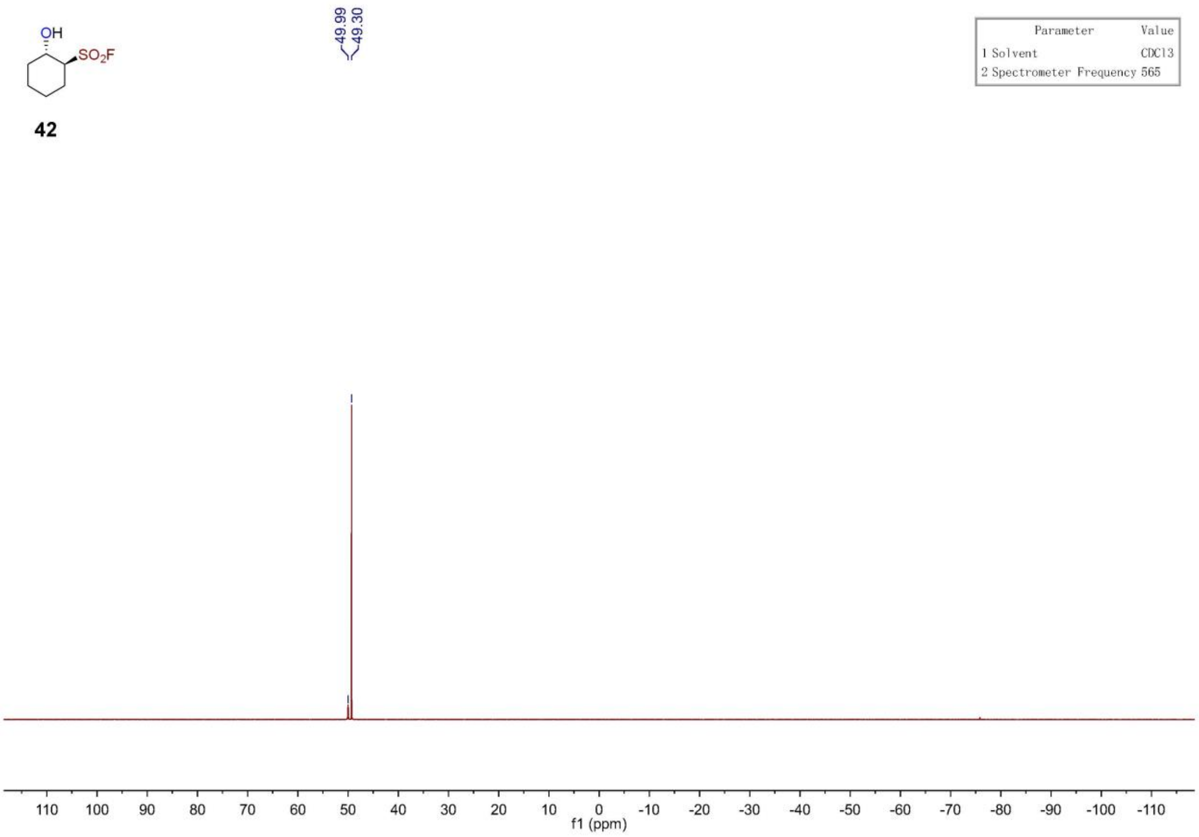

Supplementary Figure 61. ^{1}H, ^{13}C and ^{19}F NMR spectra of **43**.

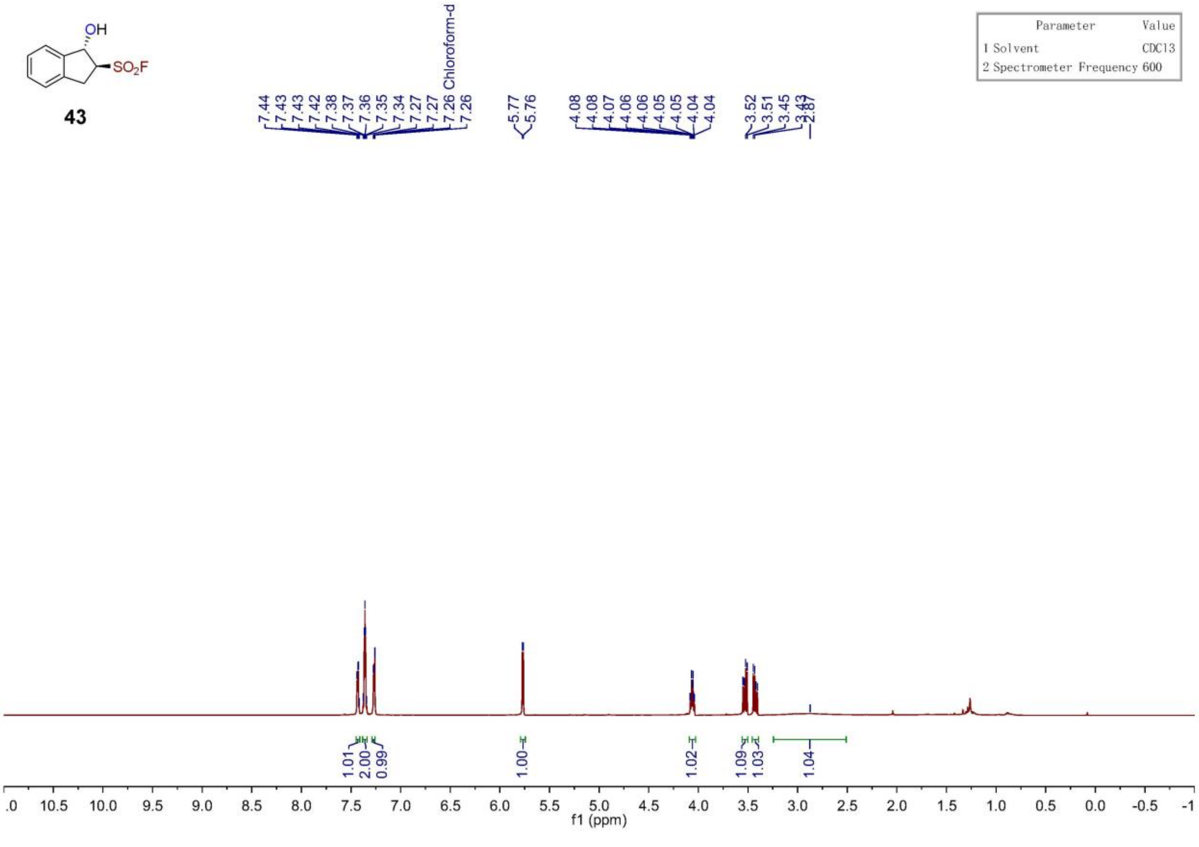

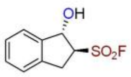

43

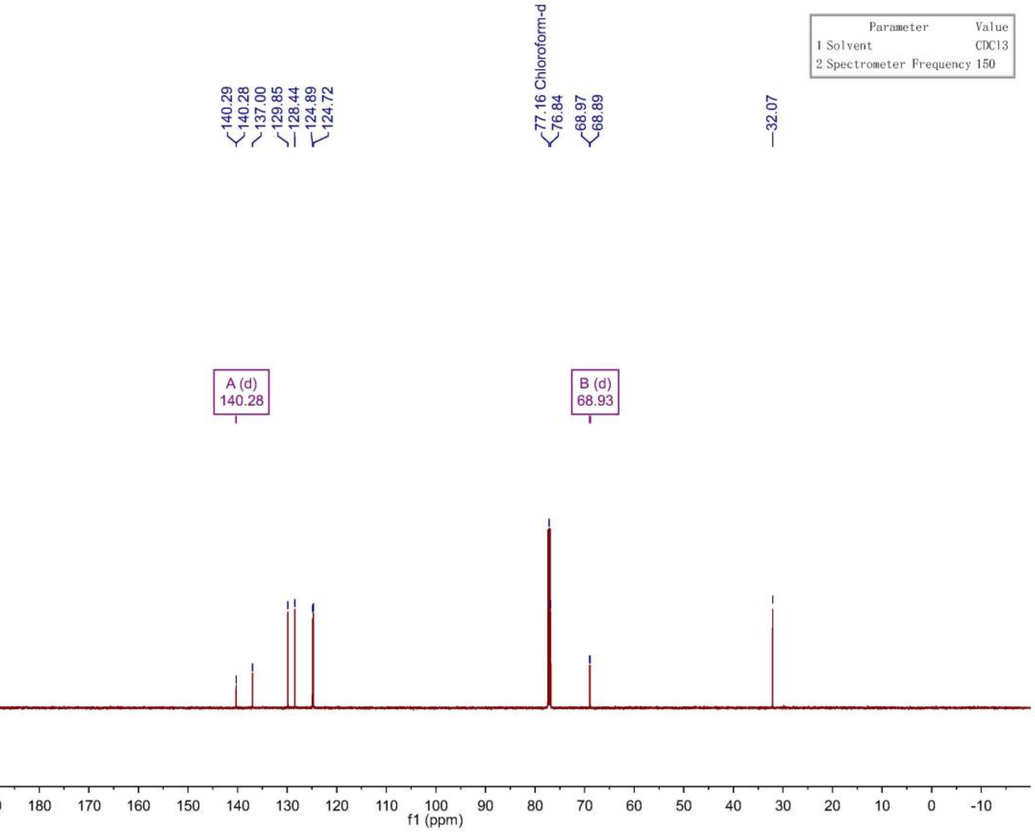

140.29
140.28
137.00
129.85
128.44
124.89
124.72
77.16 Chloroform-d
76.84
68.97
68.89
32.07
Parameter Value
1 Solvent CDCl3
2 Spectrometer Frequency 150
A (d)
140.28
B (d)
68.93
f1 (ppm)

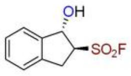

43

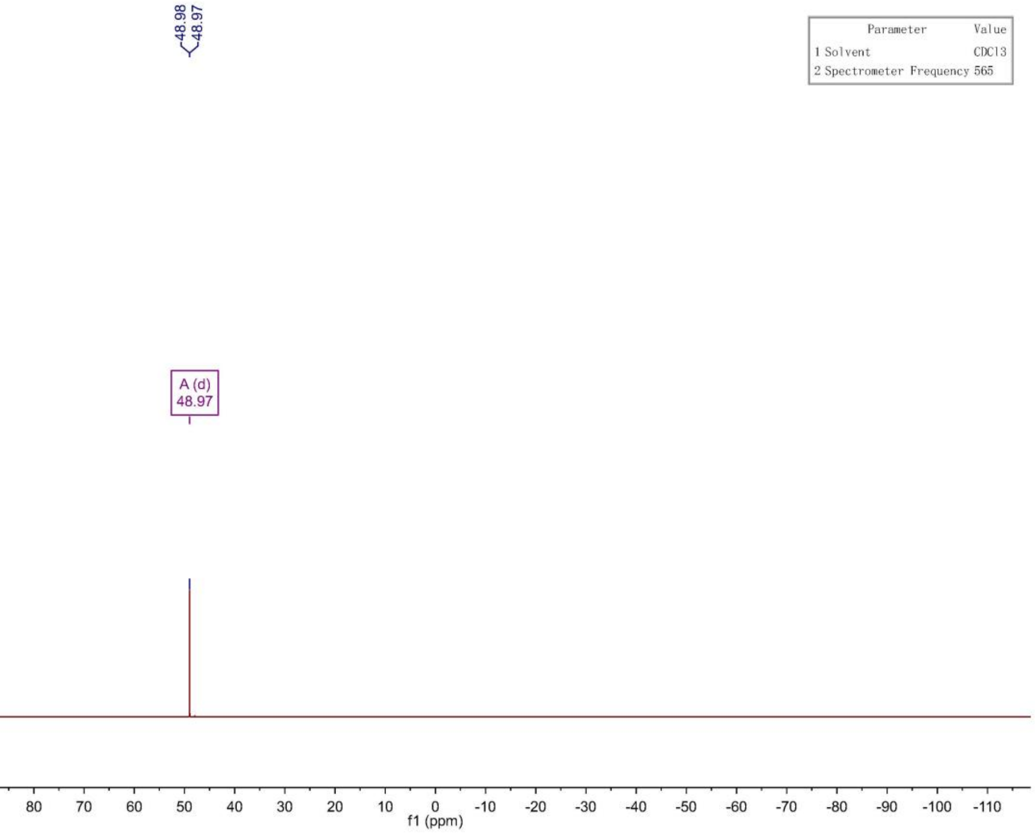

48.98
48.97
Parameter Value
1 Solvent CDCl3
2 Spectrometer Frequency 565
A (d)
48.97
f1 (ppm)

Supplementary Figure 62. ^{1}H, ^{13}C and ^{19}F NMR spectra of **44**.

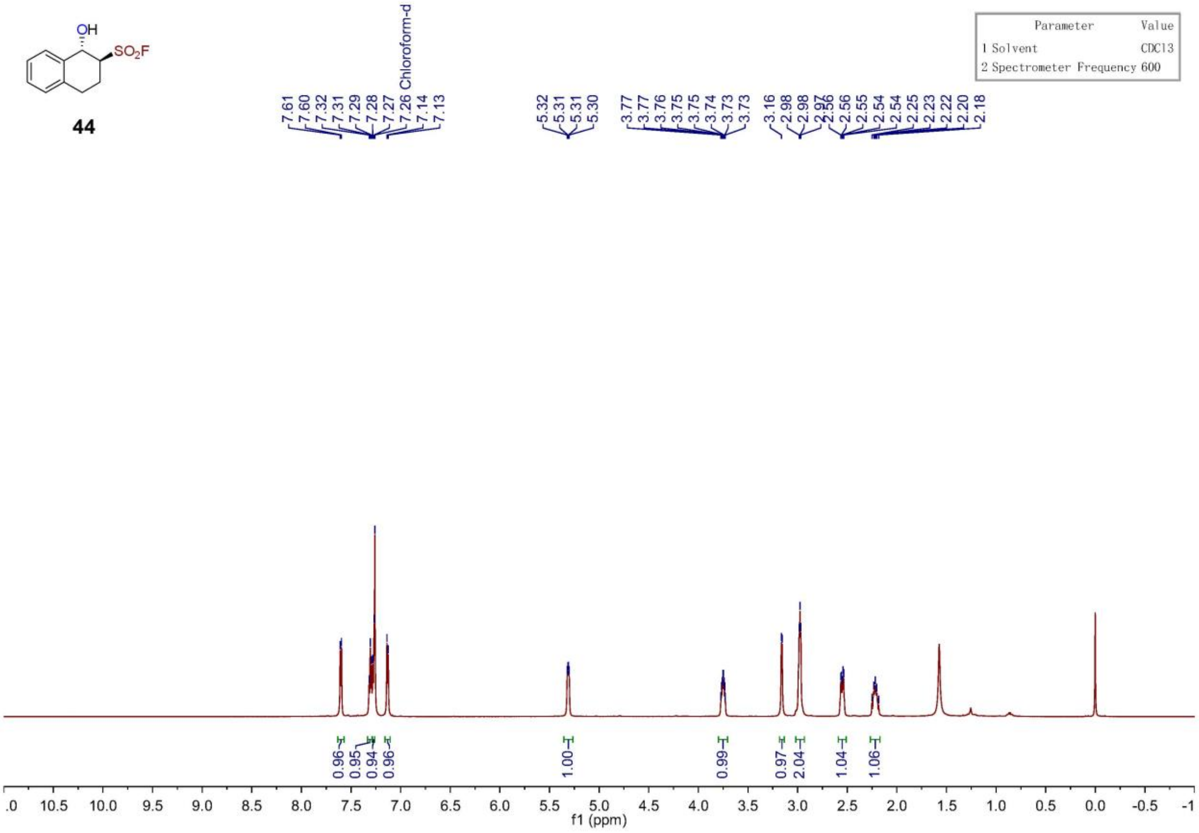

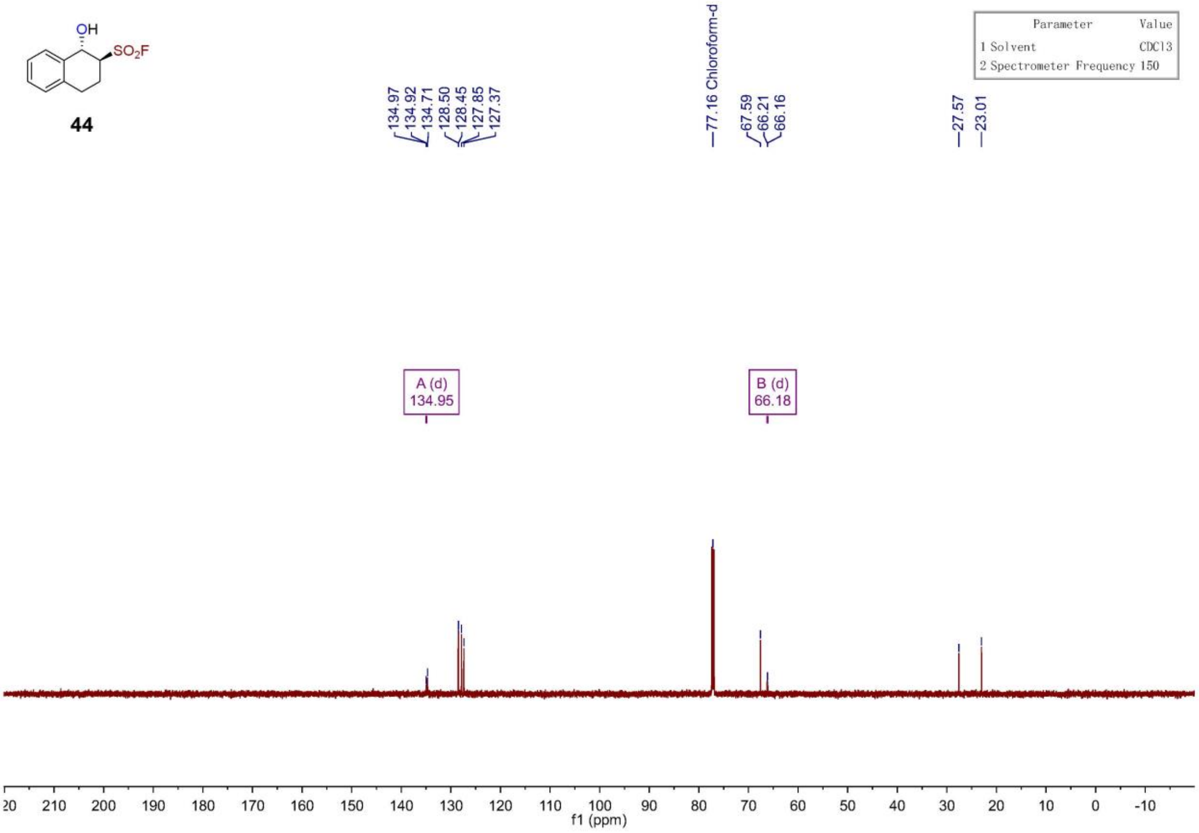

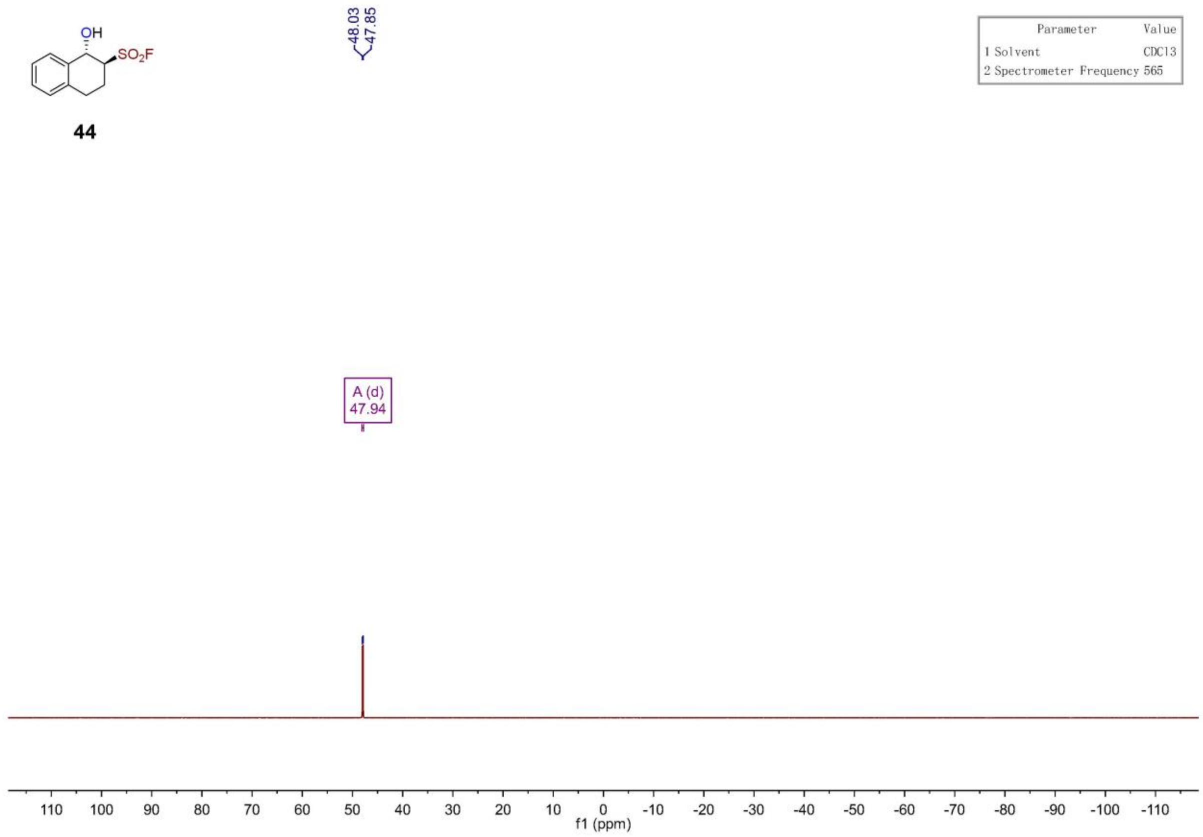

Supplementary Figure 63. ^{1}H, ^{13}C and ^{19}F NMR spectra of **45**.

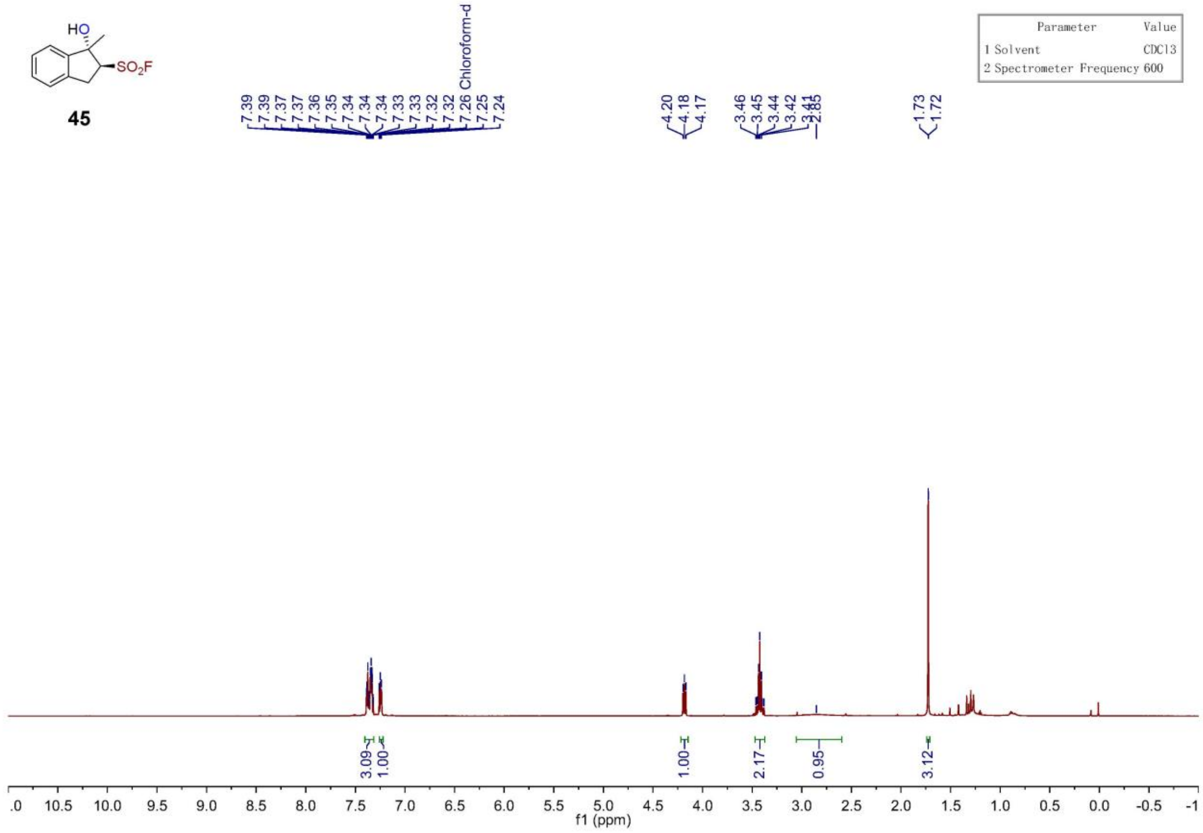

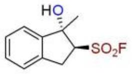

45

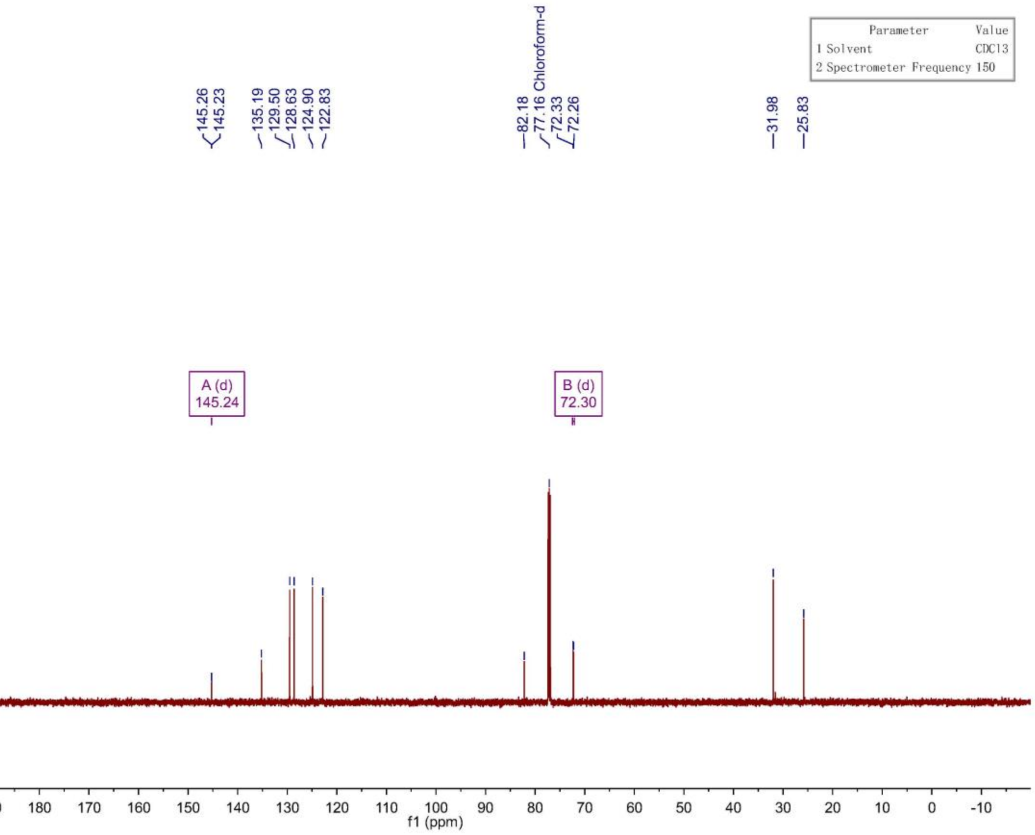

145.26
145.23
135.19
129.50
128.63
124.90
122.83
82.18
77.16 Chloroform-d
72.33
72.26
31.98
25.83
Parameter Value
1 Solvent CDCl3
2 Spectrometer Frequency 150
A (d)
145.24
B (d)
72.30
f1 (ppm)

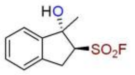

45

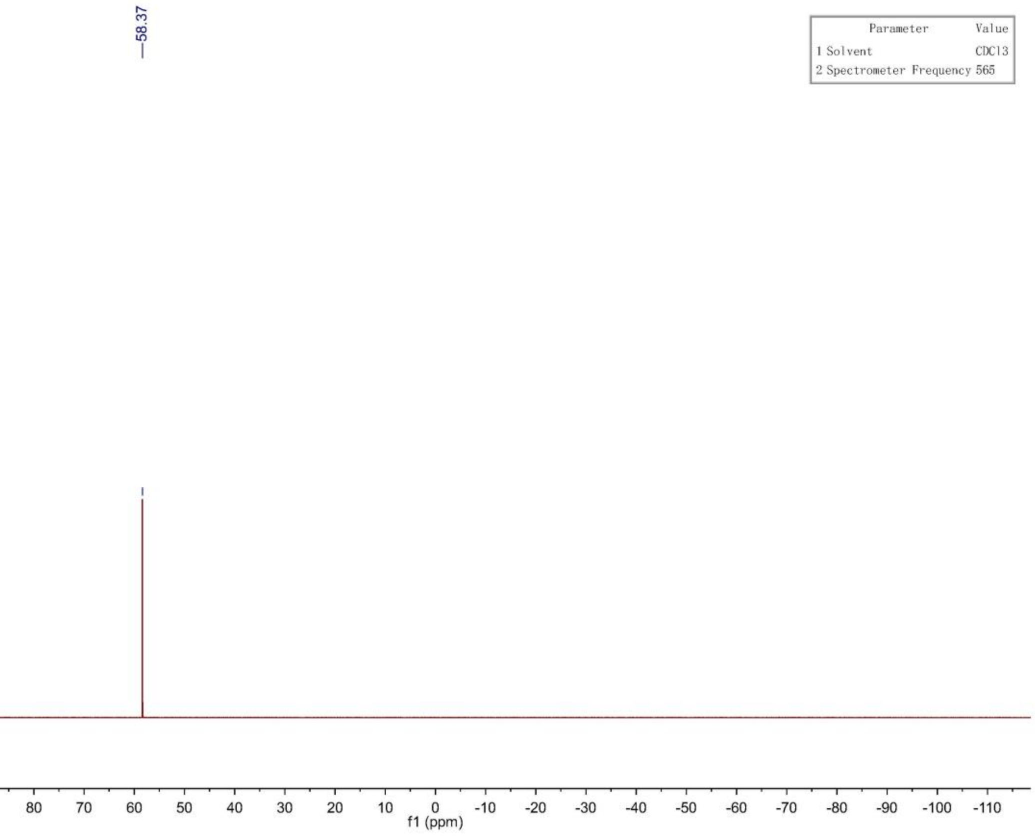

-58.37
Parameter Value
1 Solvent CDCl3
2 Spectrometer Frequency 565
f1 (ppm)

Supplementary Figure 64. ^{1}H, ^{13}C and ^{19}F NMR spectra of **46**.

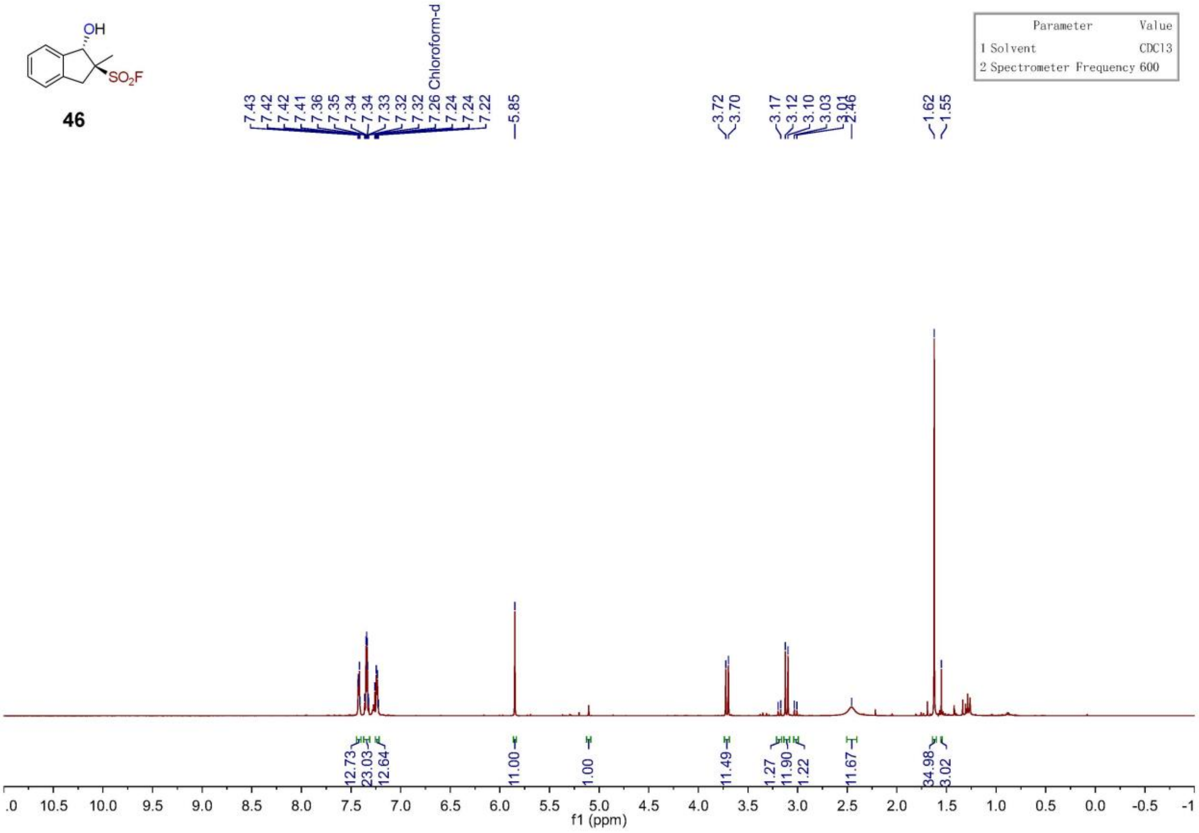

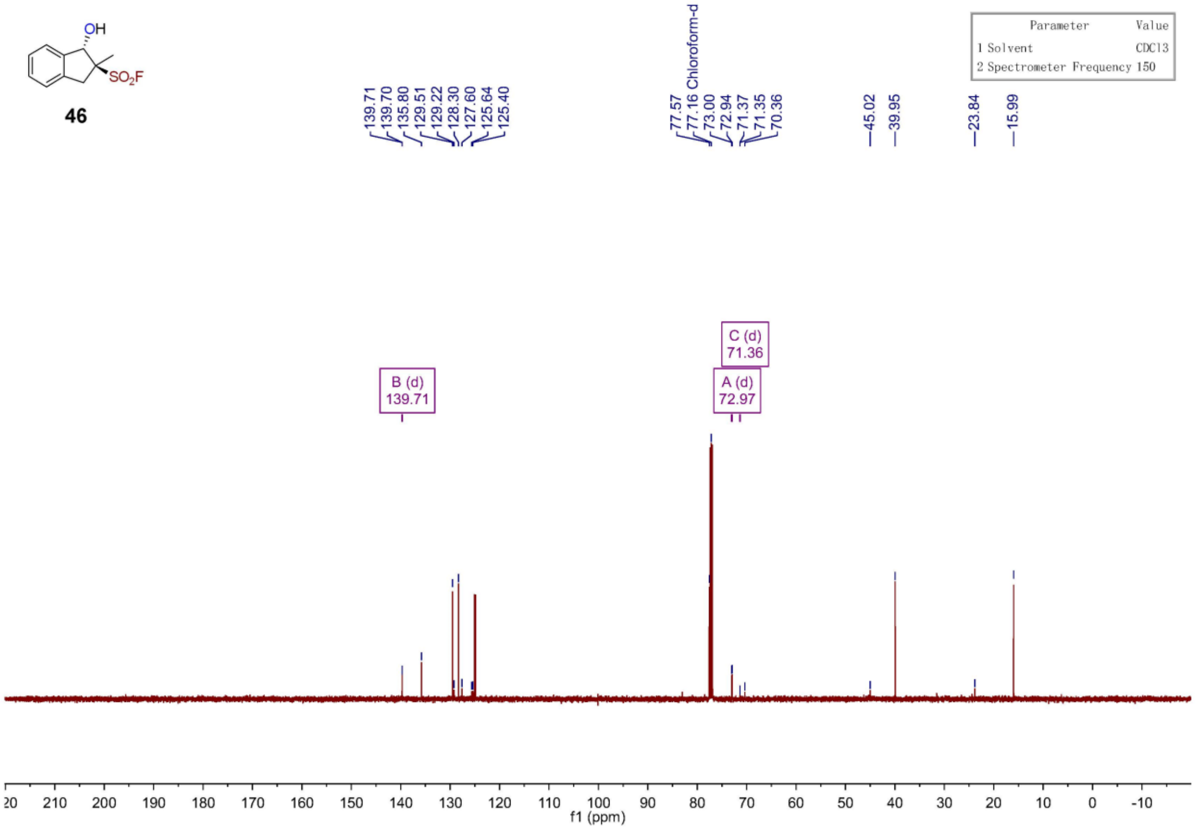

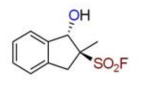

46

Parameter	Value
1 Solvent	CDCl3
2 Spectrometer Frequency	565

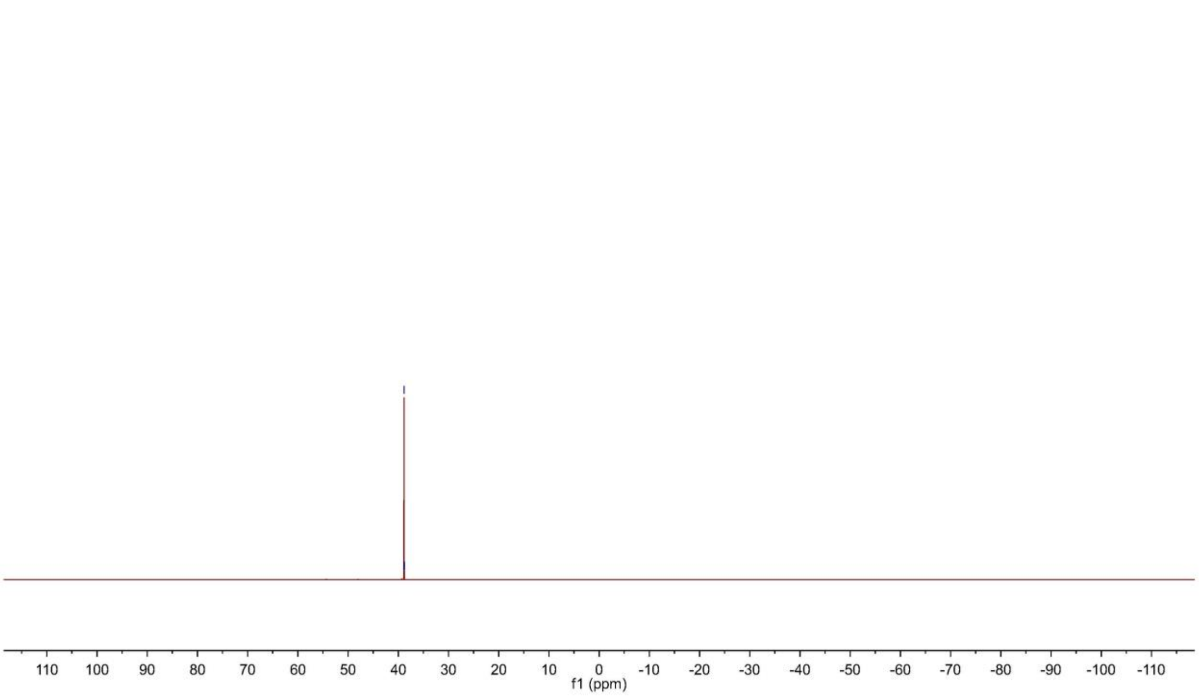

Supplementary Figure 65. ^{1}H, ^{13}C and ^{19}F NMR spectra of **47**.

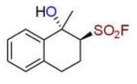

47

Parameter	Value
1 Solvent	CDCl3
2 Spectrometer Frequency	600

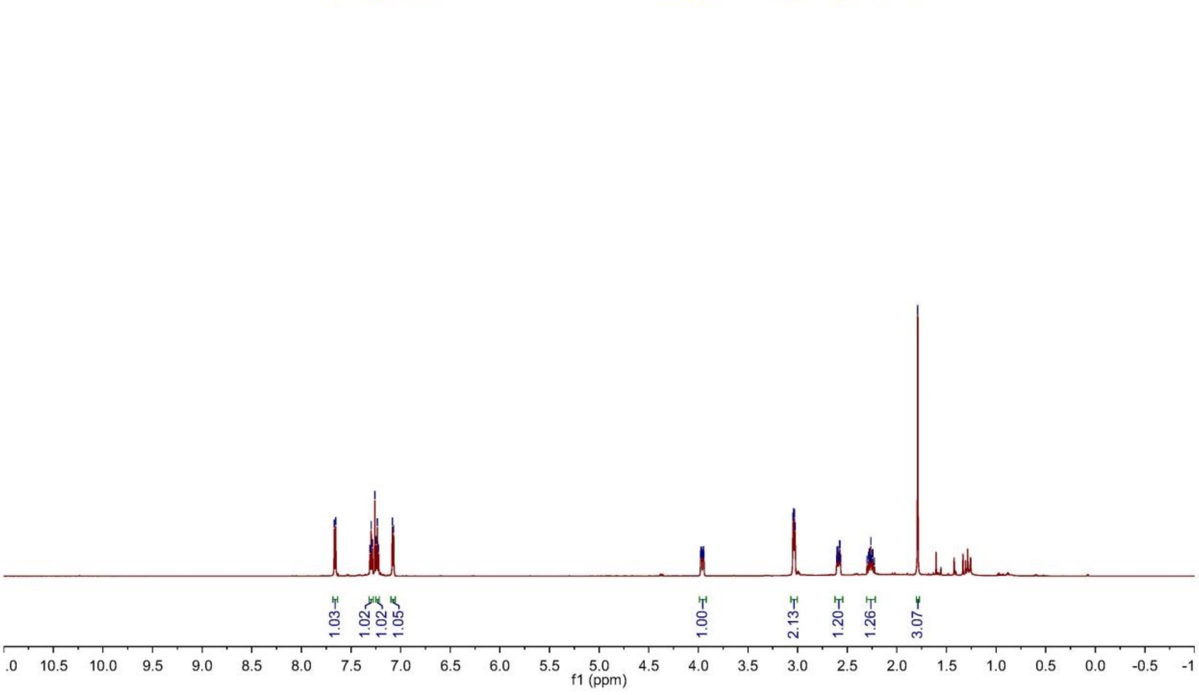

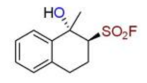

47

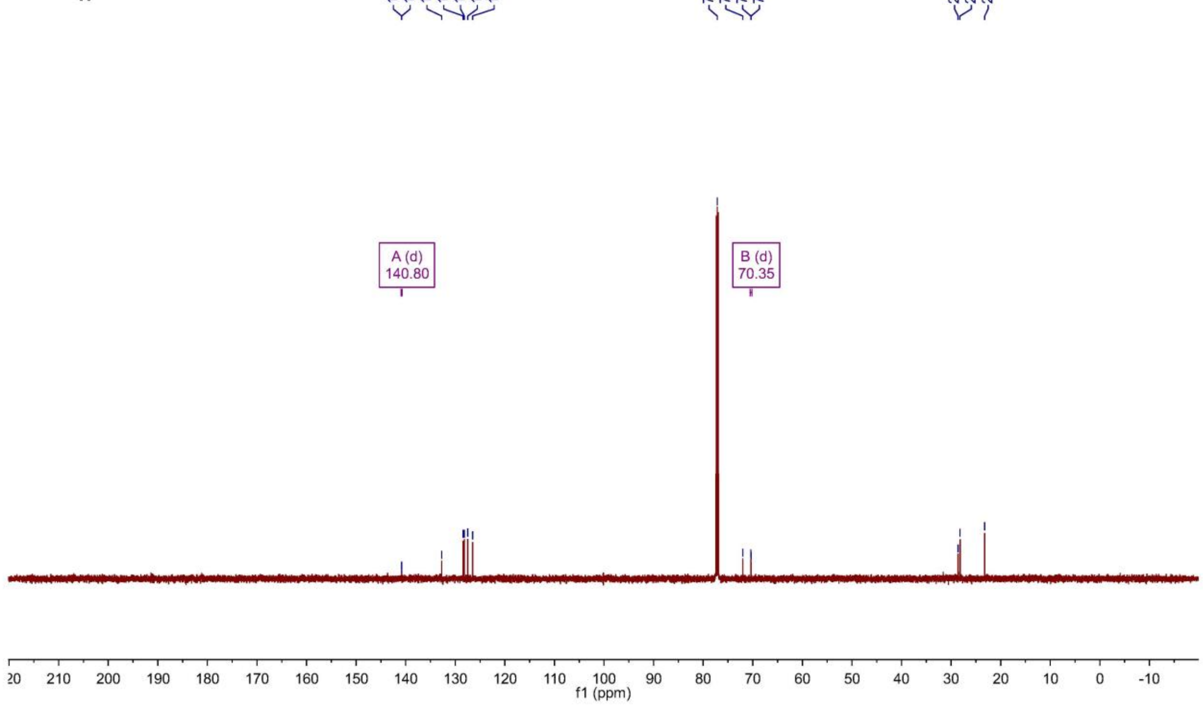

Parameter Value
1 Solvent CDCl3
2 Spectrometer Frequency 150
140.82
140.79
132.75
128.40
128.18
127.48
126.52
77.16 Chloroform-d
72.01
70.38
70.32
28.60
28.19
23.22
A (d)
140.80
B (d)
70.35
f1 (ppm)

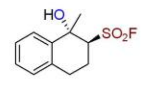

47

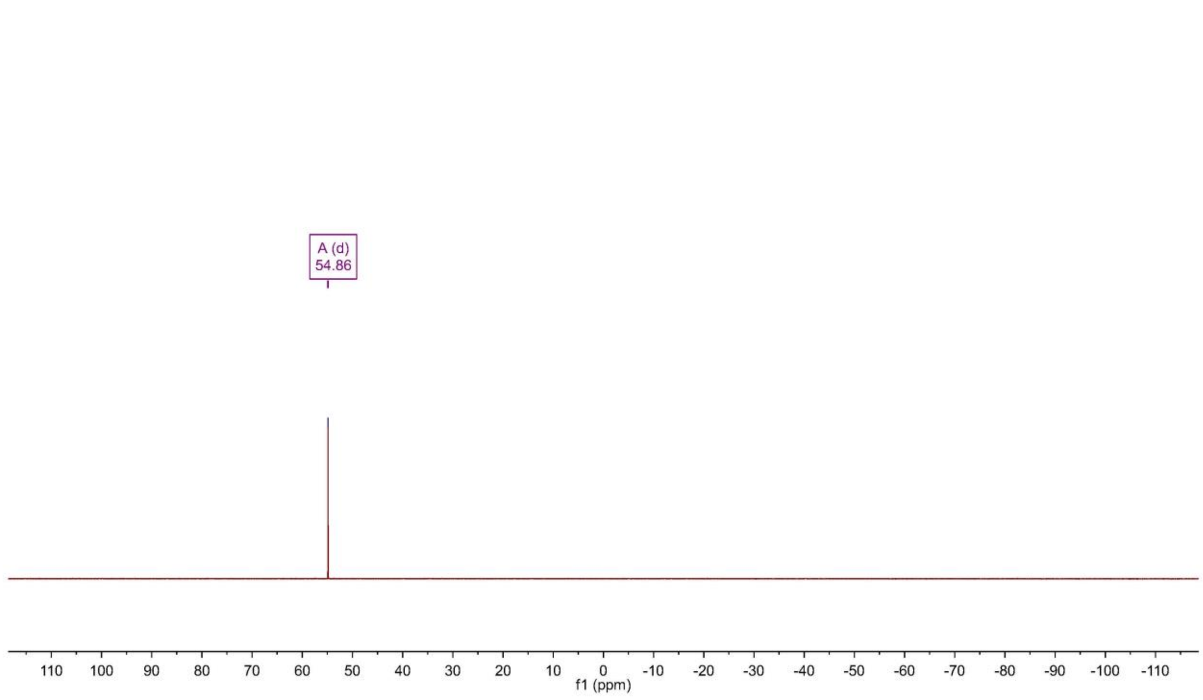

Parameter Value
1 Solvent CDCl3
2 Spectrometer Frequency 565
54.86
54.86
A (d)
54.86
f1 (ppm)

Supplementary Figure 66. ^{1}H, ^{13}C and ^{19}F NMR spectra of **48**.

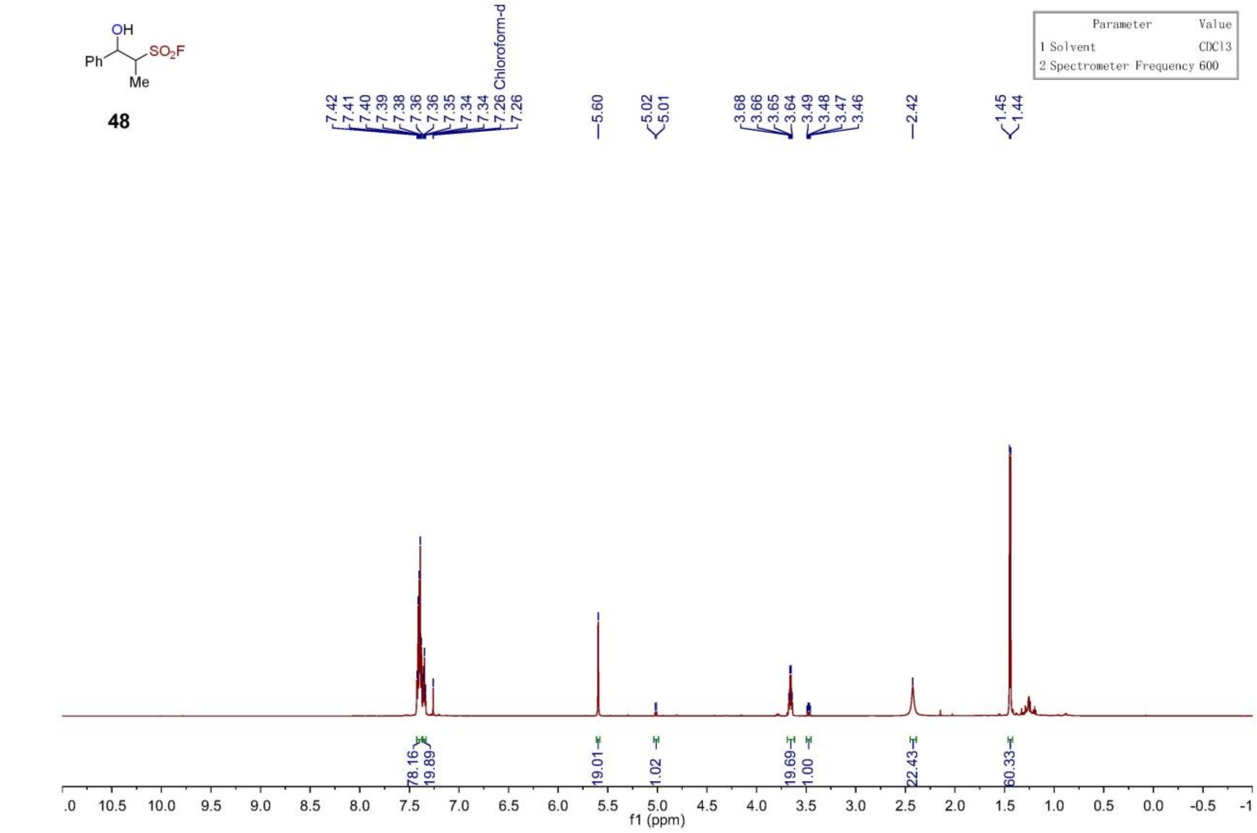

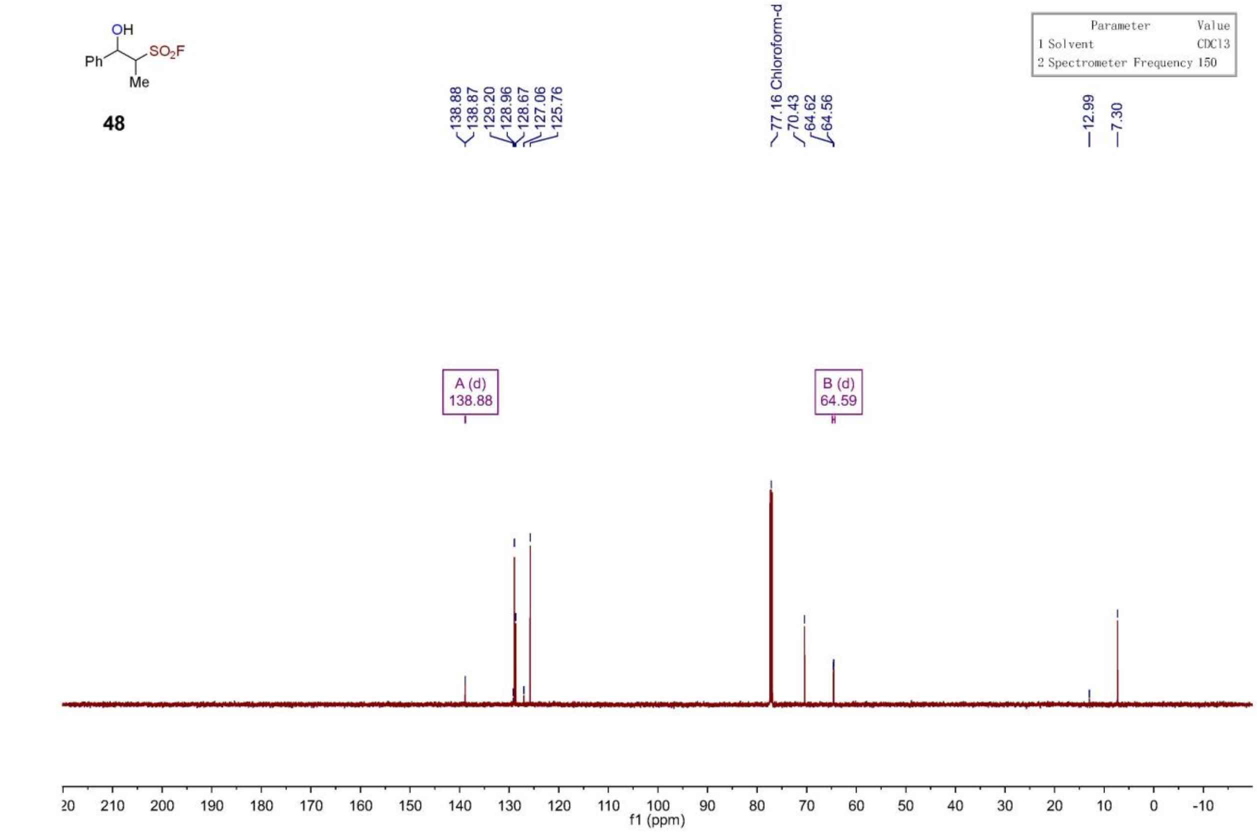

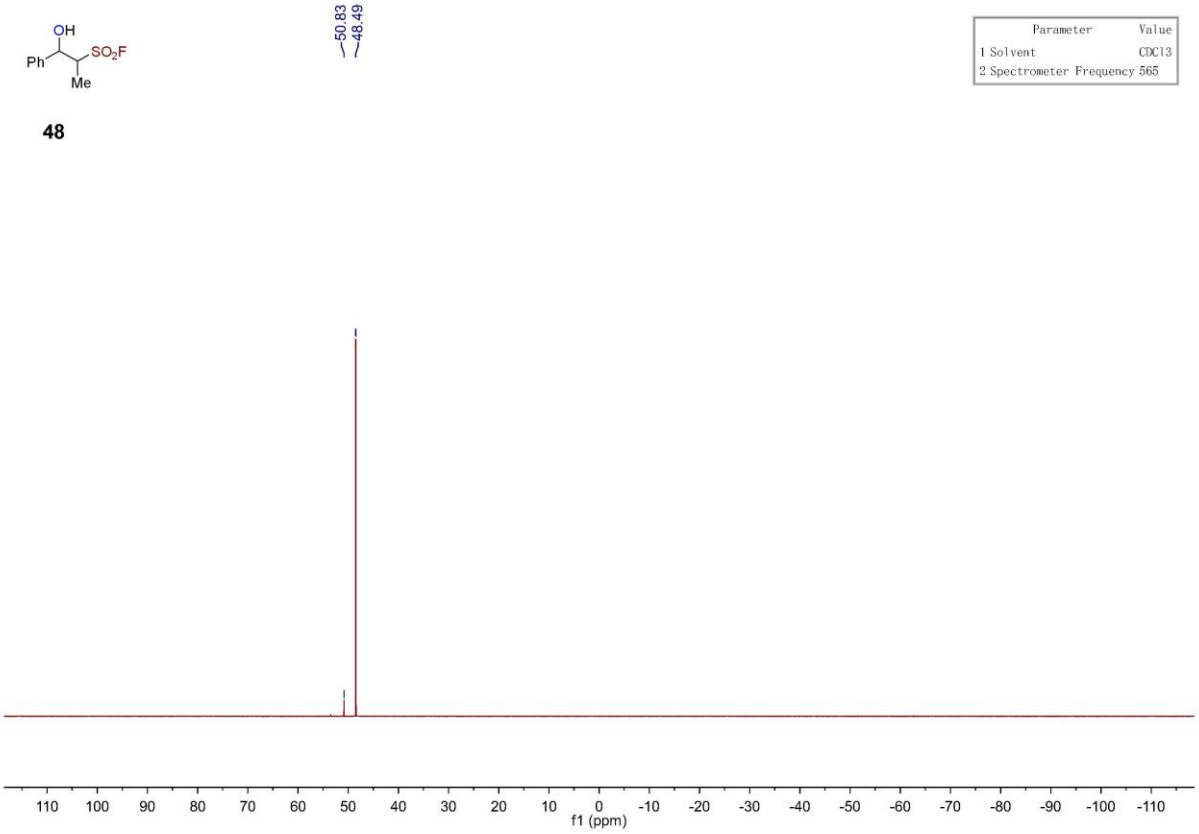

Supplementary Figure 67. ^{1}H, ^{13}C and ^{19}F NMR spectra of **49**.

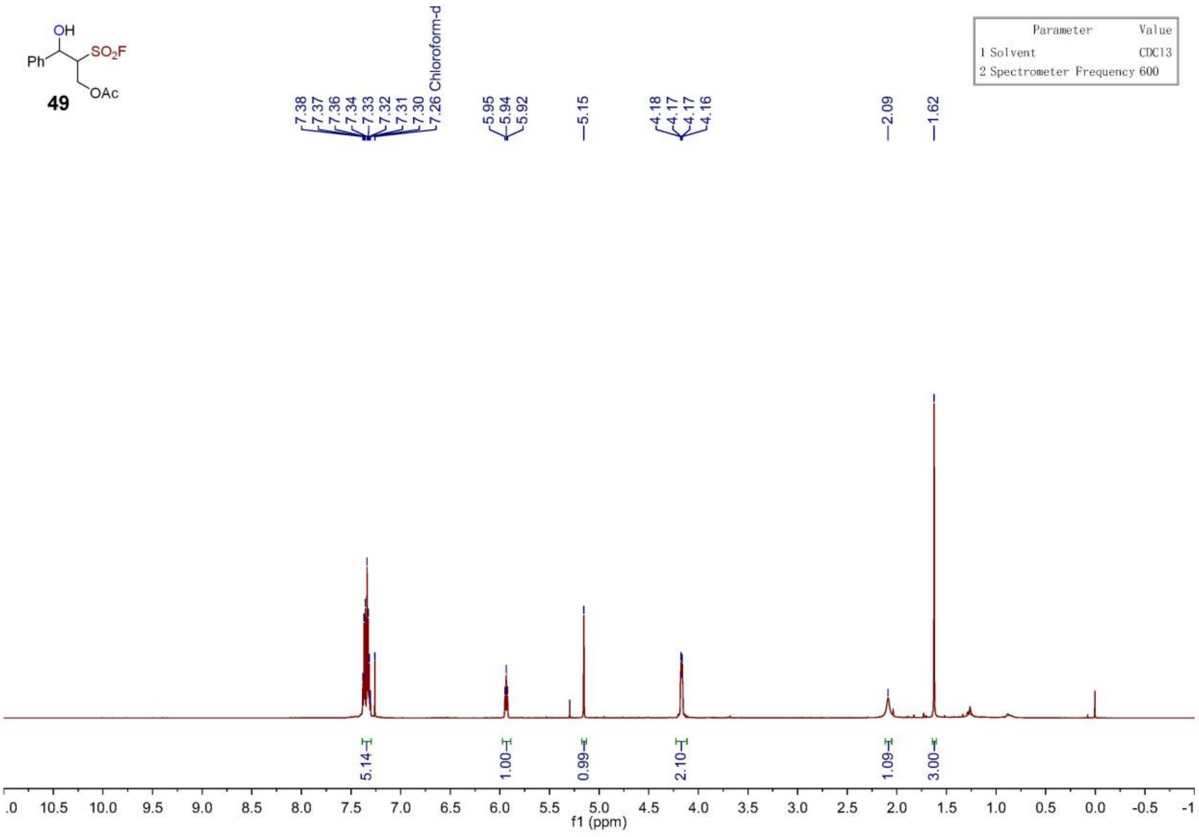

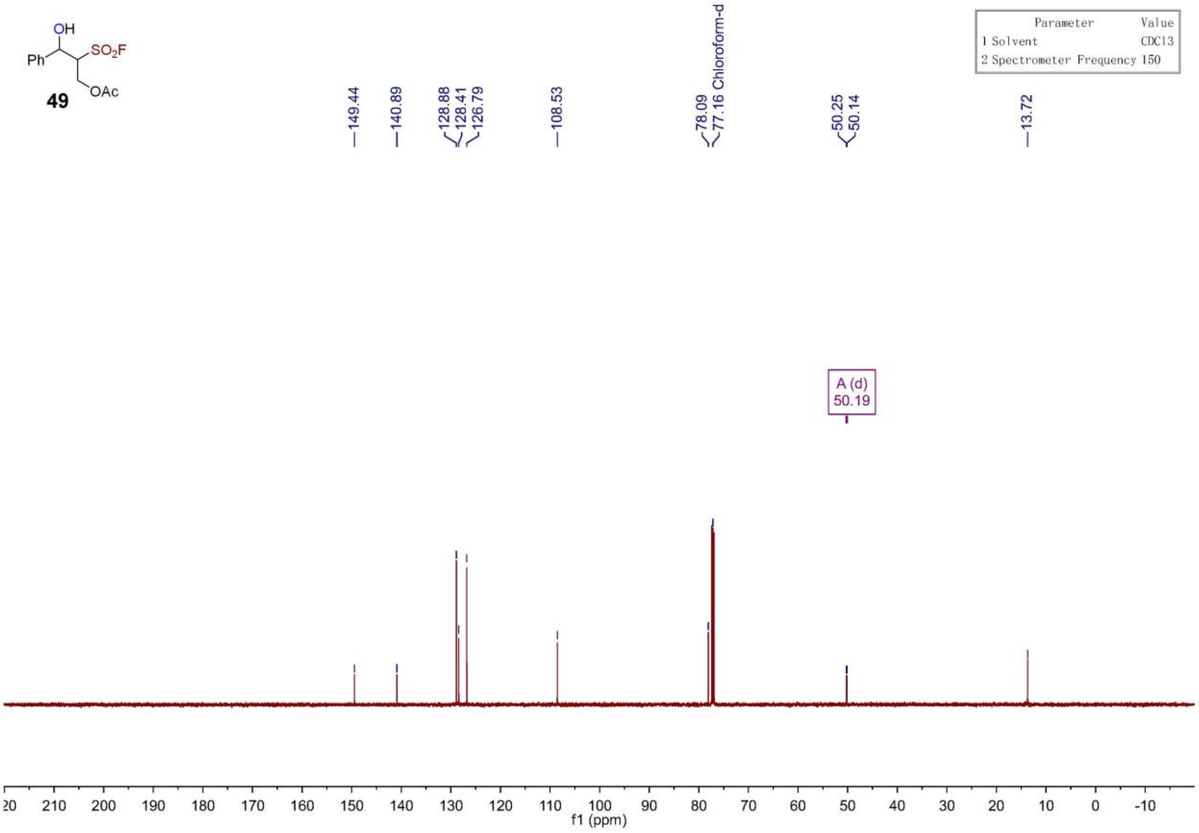
OH
SO2F
Ph
OAc
49
149.44
140.89
128.88
128.41
126.79
108.53
78.09
77.16 Chloroform-d
50.25
50.14
13.72
Parameter Value
1 Solvent CDCl3
2 Spectrometer Frequency 150
A (d)
50.19
f1 (ppm)

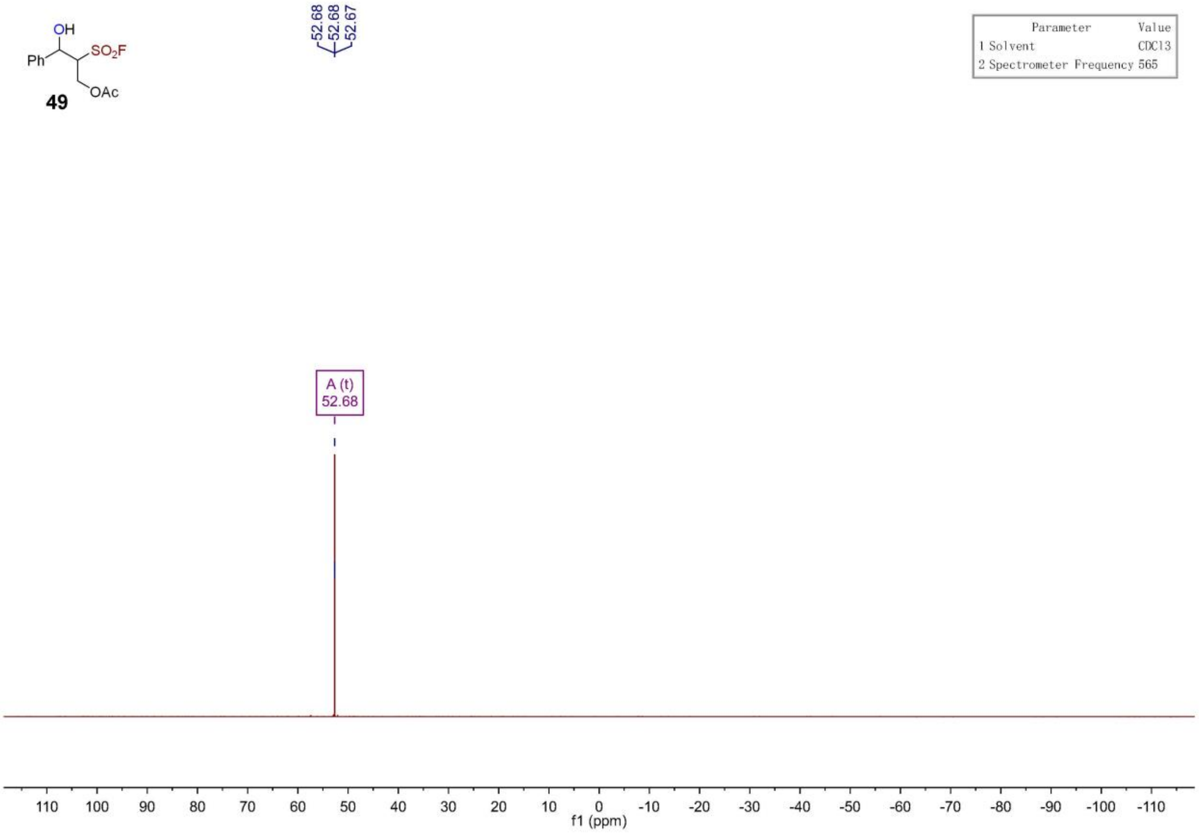
OH
SO2F
Ph
OAc
49
52.68
52.68
52.67
Parameter Value
1 Solvent CDCl3
2 Spectrometer Frequency 565
A (t)
52.68
f1 (ppm)

Supplementary Figure 68. ^{1}H, ^{13}C and ^{19}F NMR spectra of **50**.

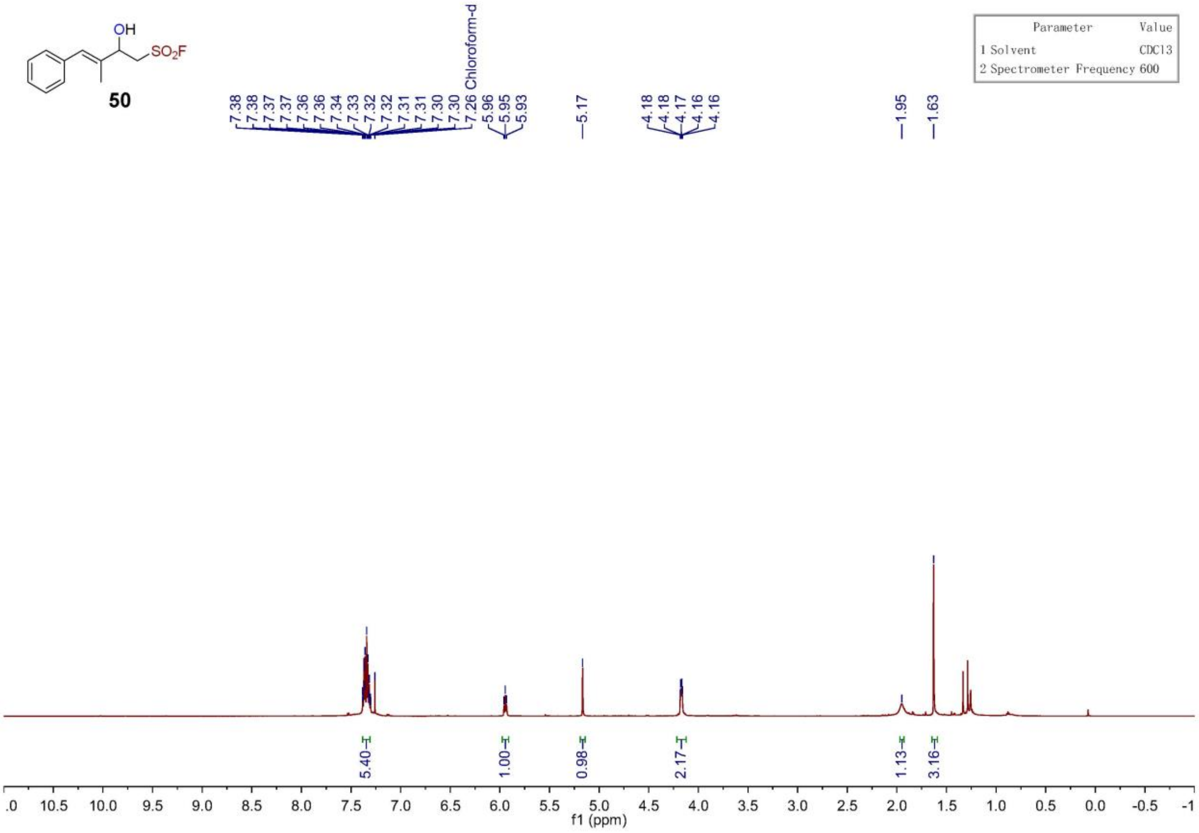

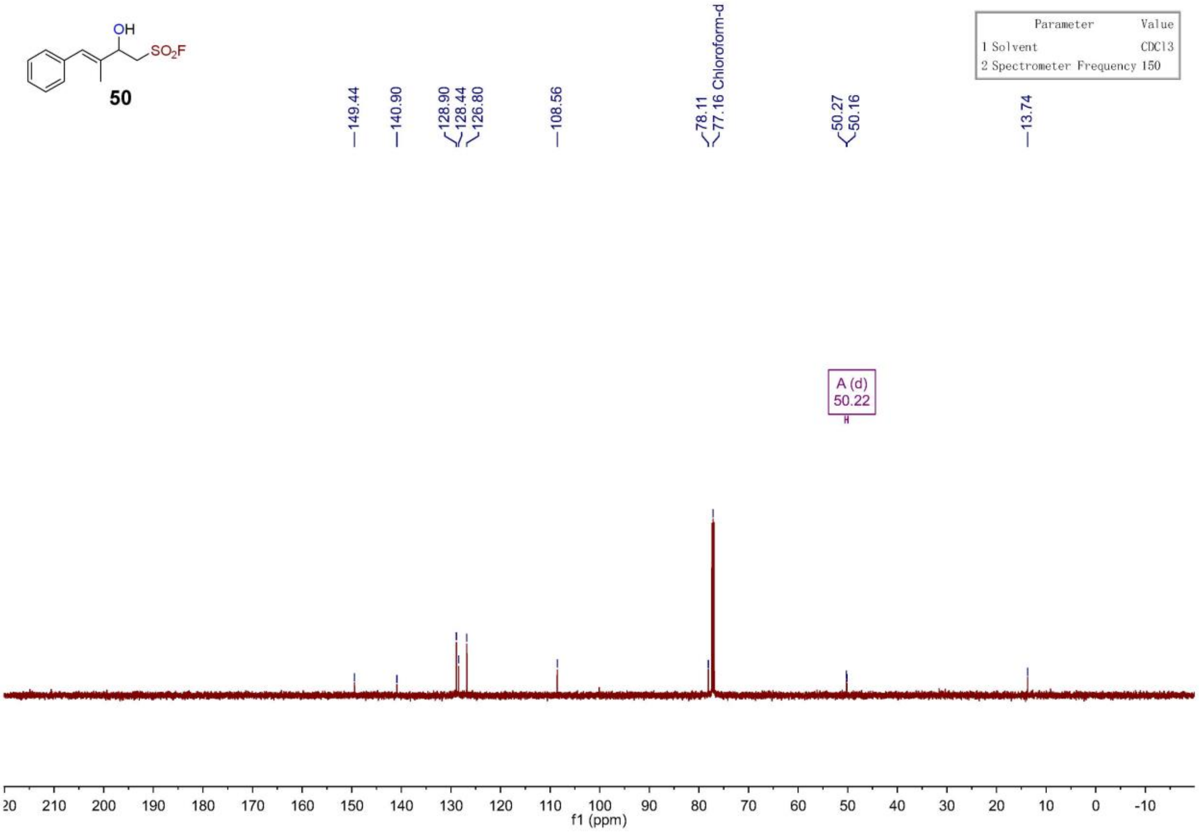

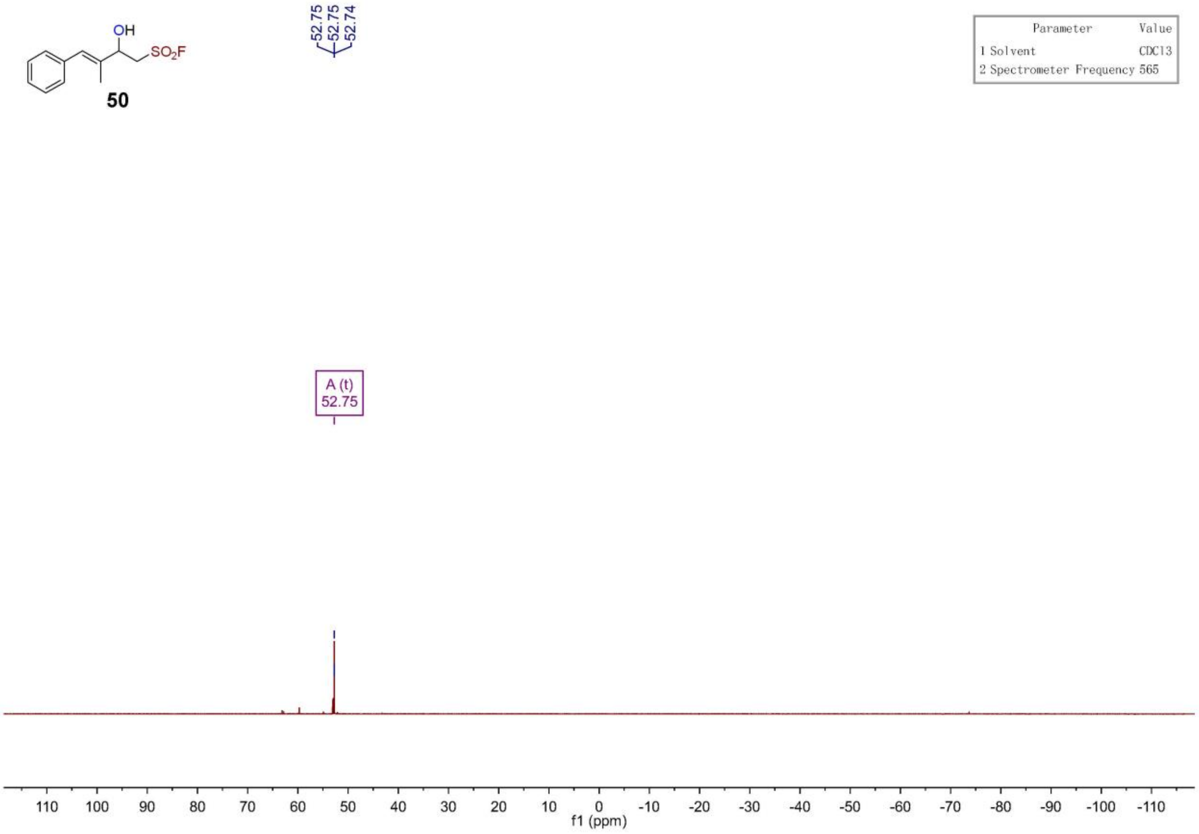

Supplementary Figure 69. ^{1}H, ^{13}C and ^{19}F NMR spectra of **51**.

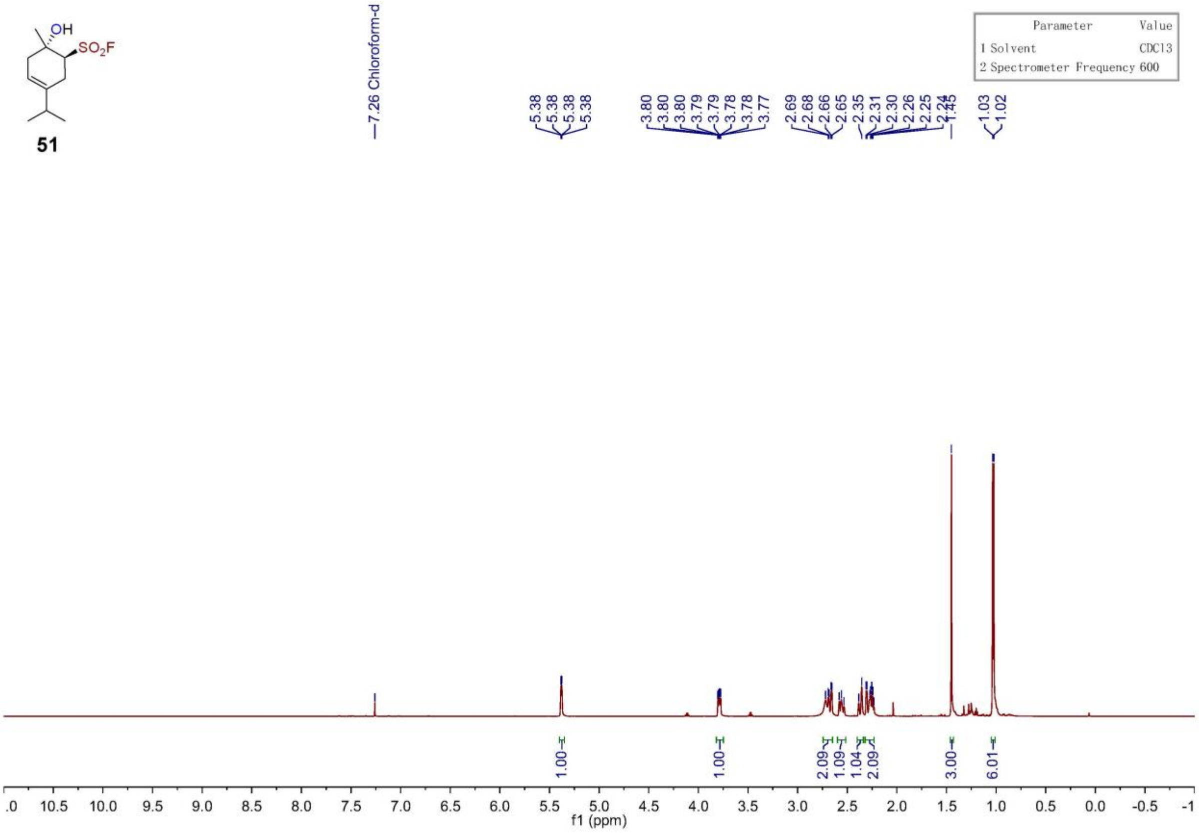

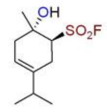

51

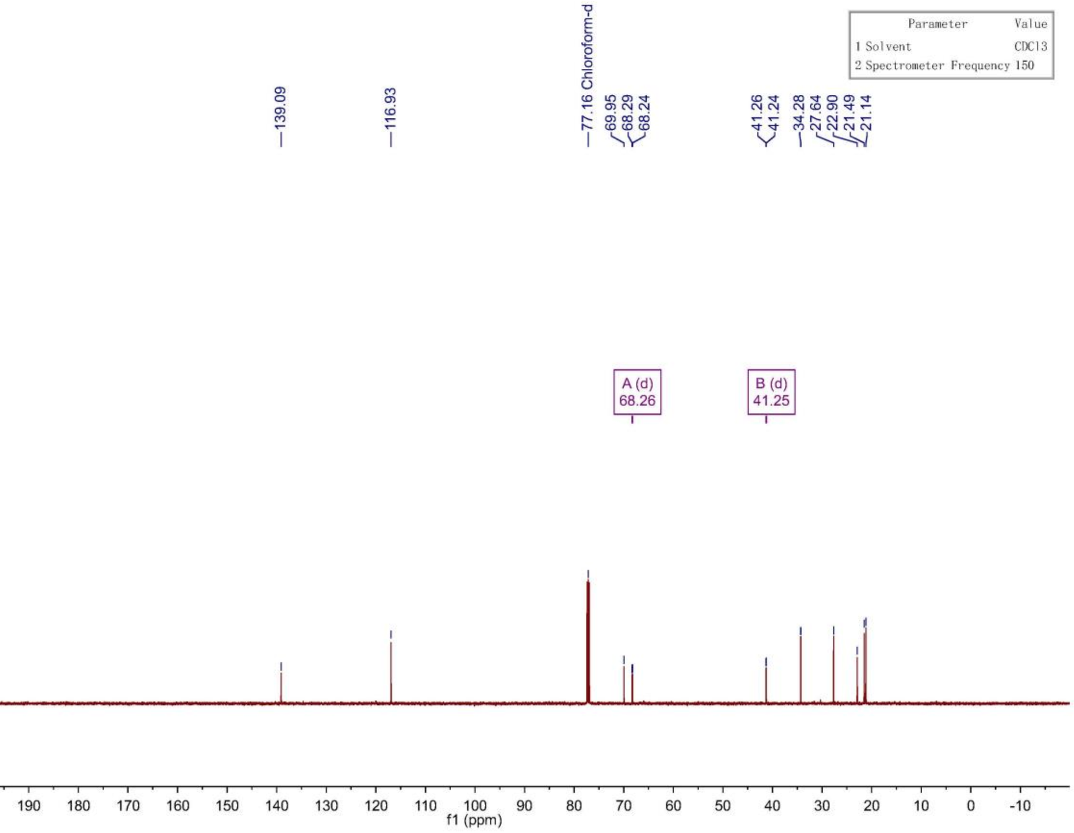

Parameter Value
1 Solvent CDCl3
2 Spectrometer Frequency 150
139.09
116.93
77.16 Chloroform-d
69.95
68.29
68.24
41.26
41.24
34.28
27.64
22.90
21.49
21.14
A (d) 68.26
B (d) 41.25
f1 (ppm)

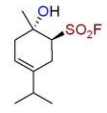

51

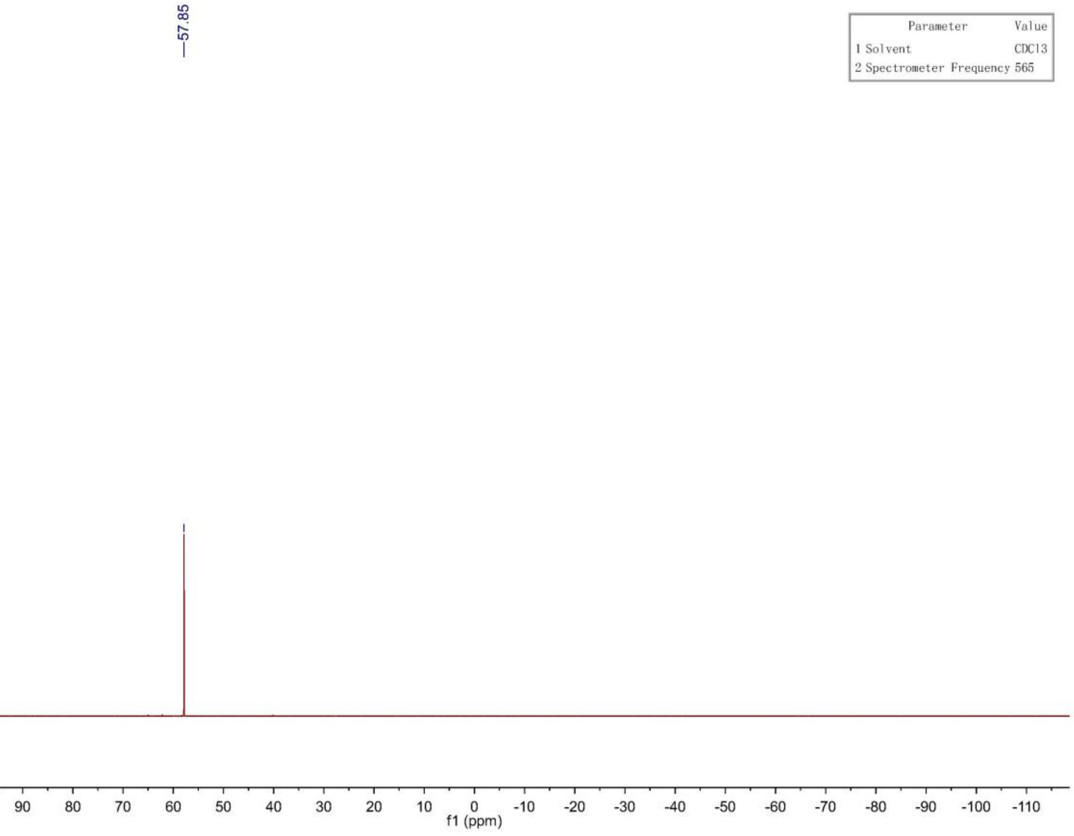

Parameter Value
1 Solvent CDCl3
2 Spectrometer Frequency 565
57.85
f1 (ppm)

Supplementary Figure 70. ^{1}H, ^{13}C and ^{19}F NMR spectra of **52**.

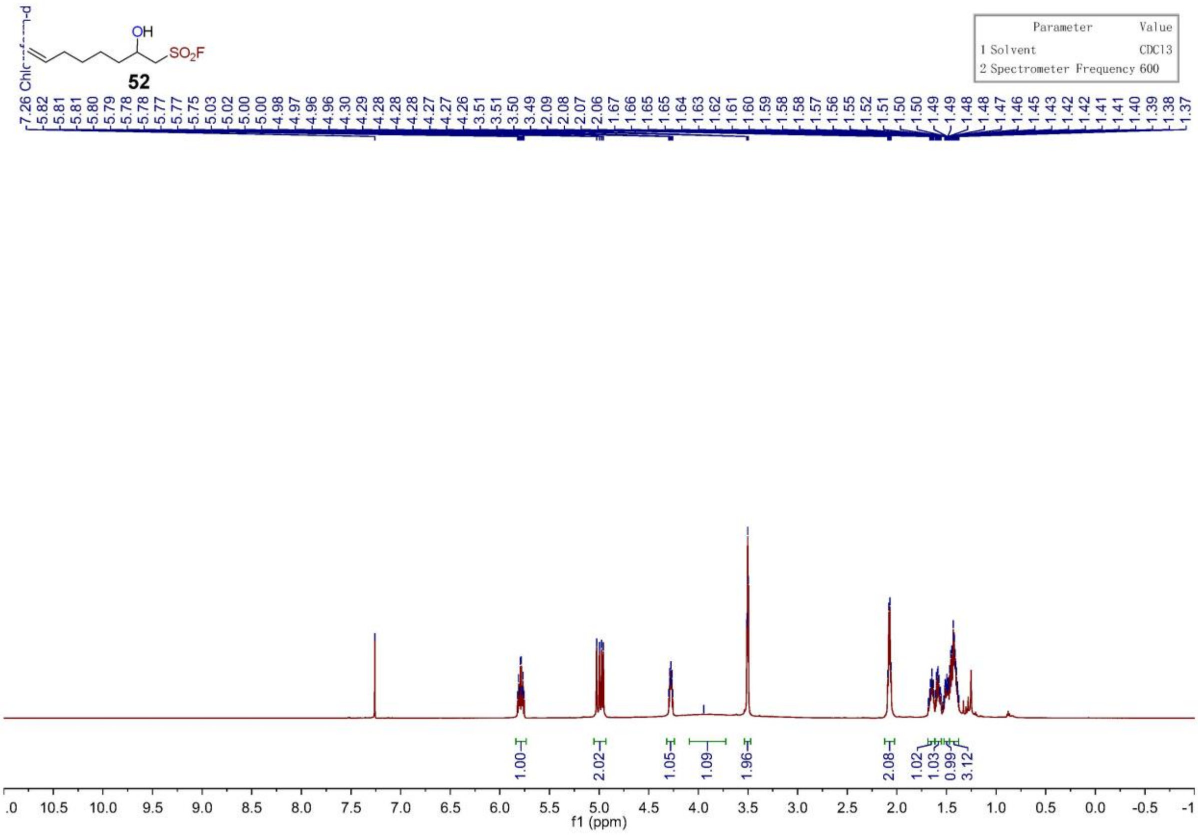

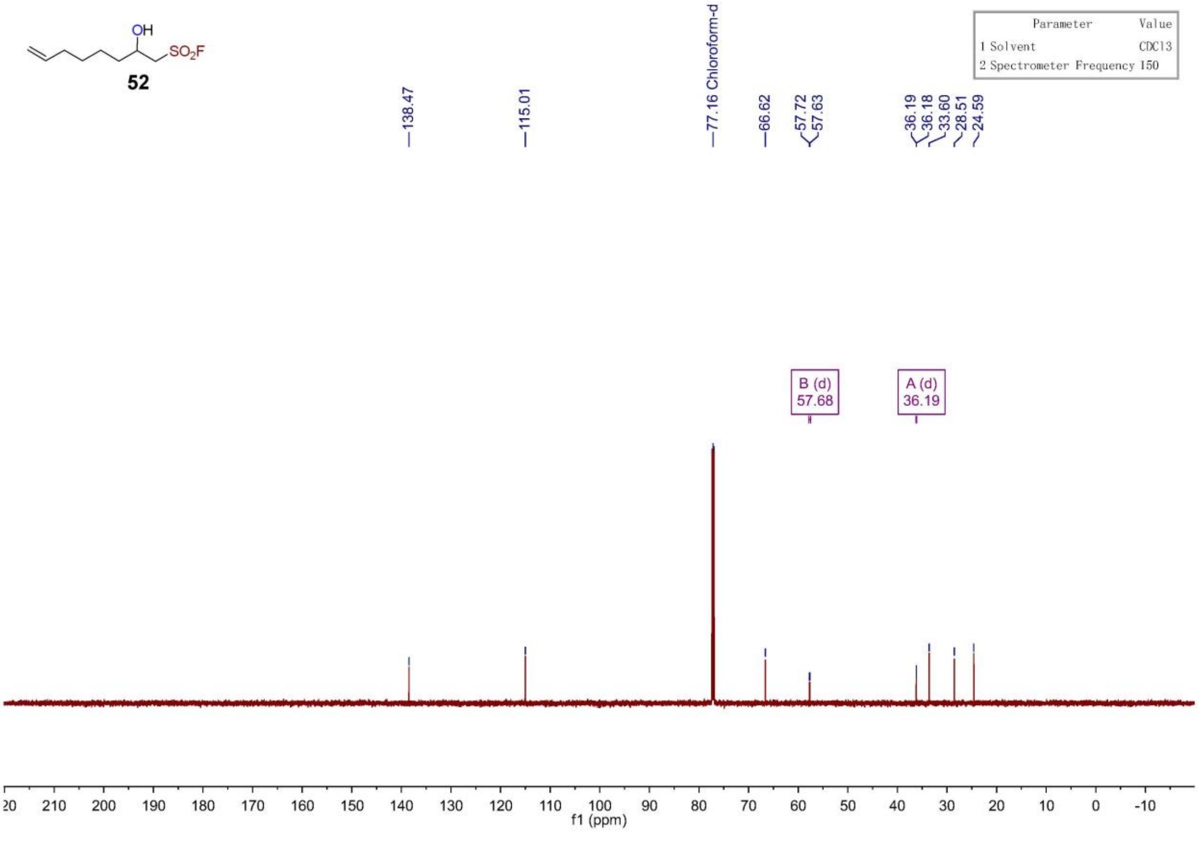

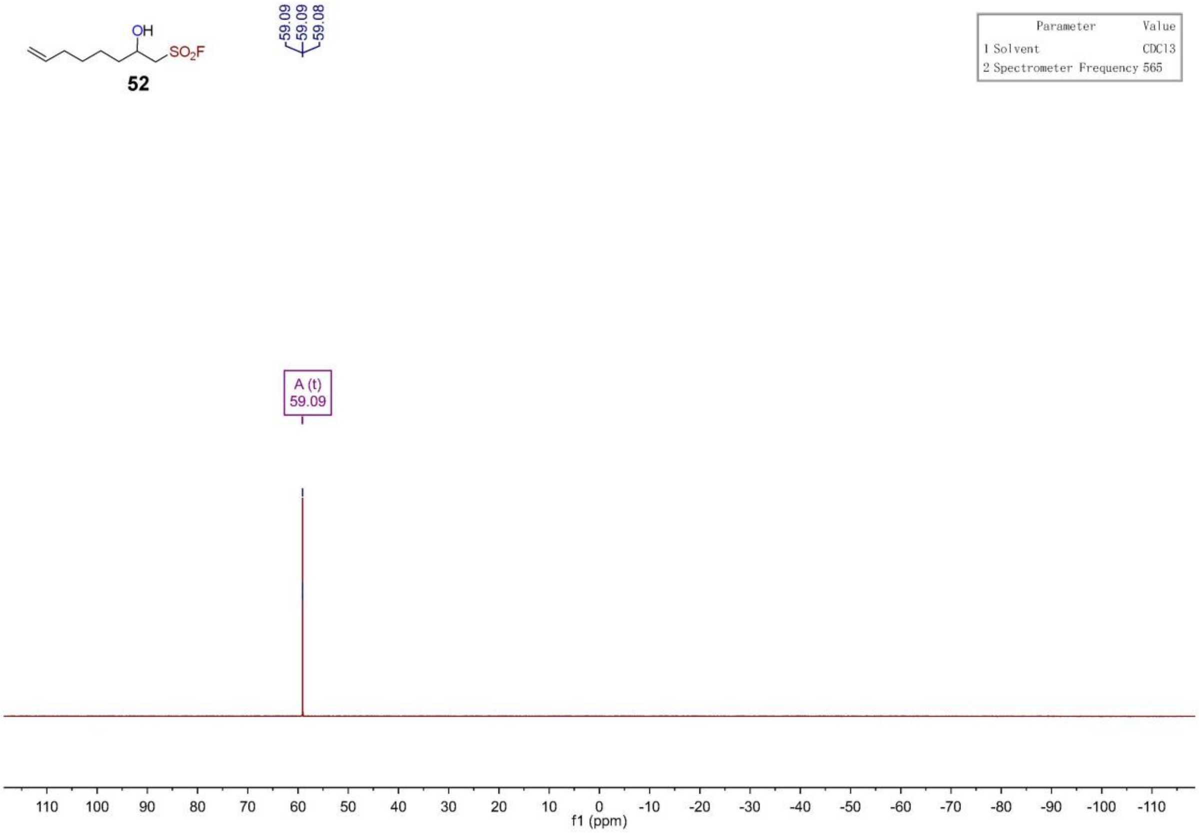

Supplementary Figure 71. ^{1}H, ^{13}C and ^{19}F NMR spectra of **53**.

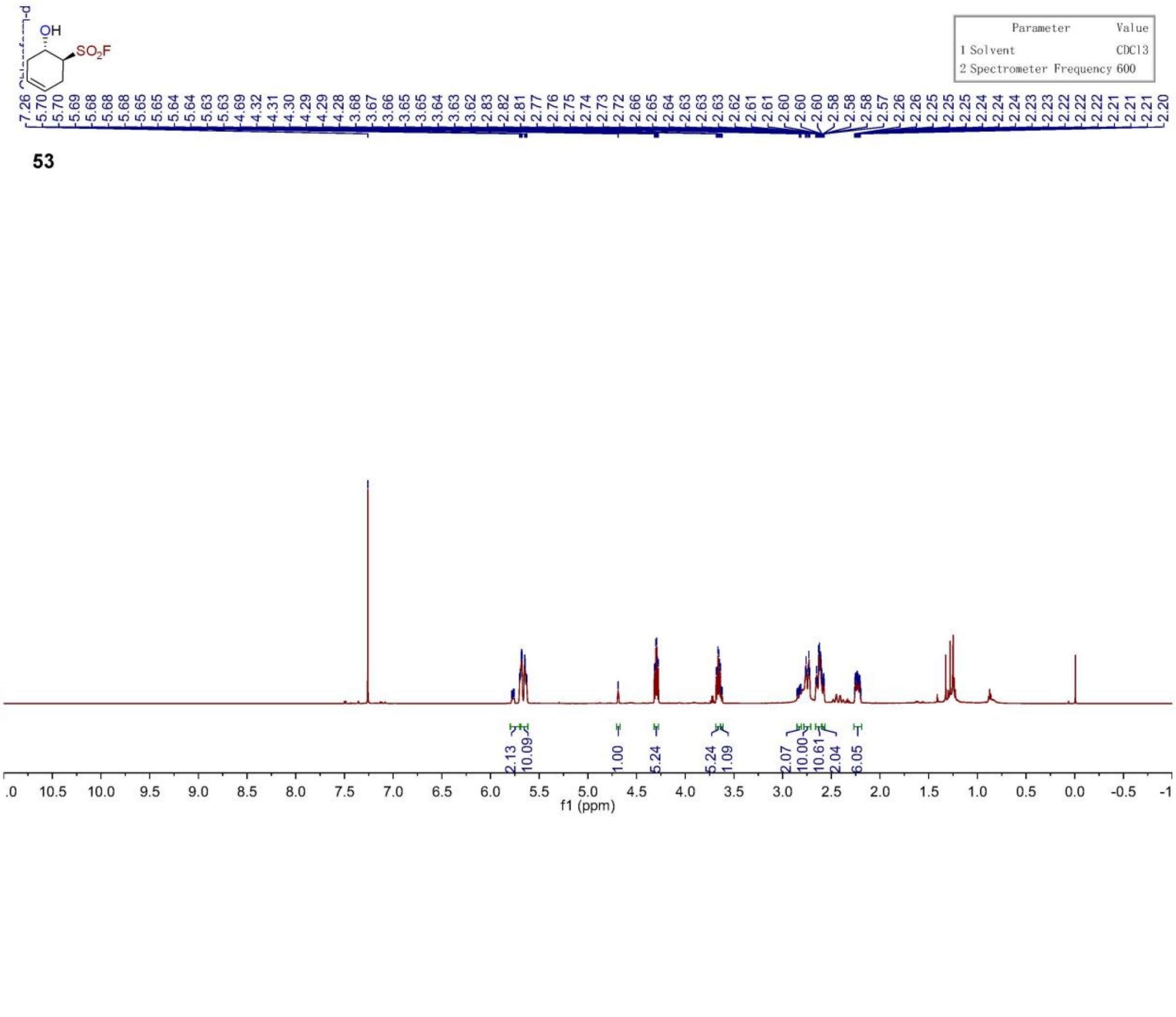

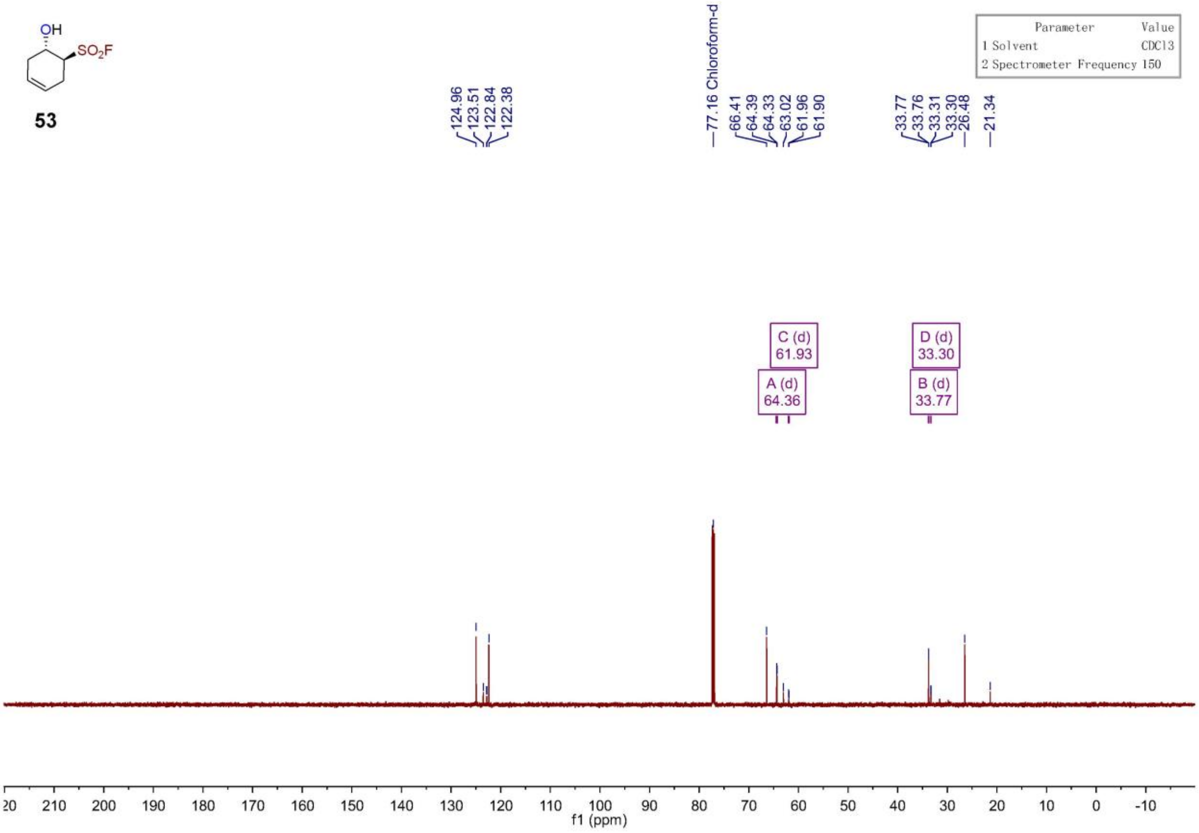
OH
SO2F
53
124.96
123.51
122.84
122.38
77.16 Chloroform-d
66.41
64.39
64.33
63.02
61.96
61.90
33.77
33.76
33.31
33.30
26.48
21.34
Parameter Value
1 Solvent CDCl3
2 Spectrometer Frequency 150
C (d) 61.93
A (d) 64.36
D (d) 33.30
B (d) 33.77
20 210 200 190 180 170 160 150 140 130 120 110 100 90 80 70 60 50 40 30 20 10 0 -10
f1 (ppm)

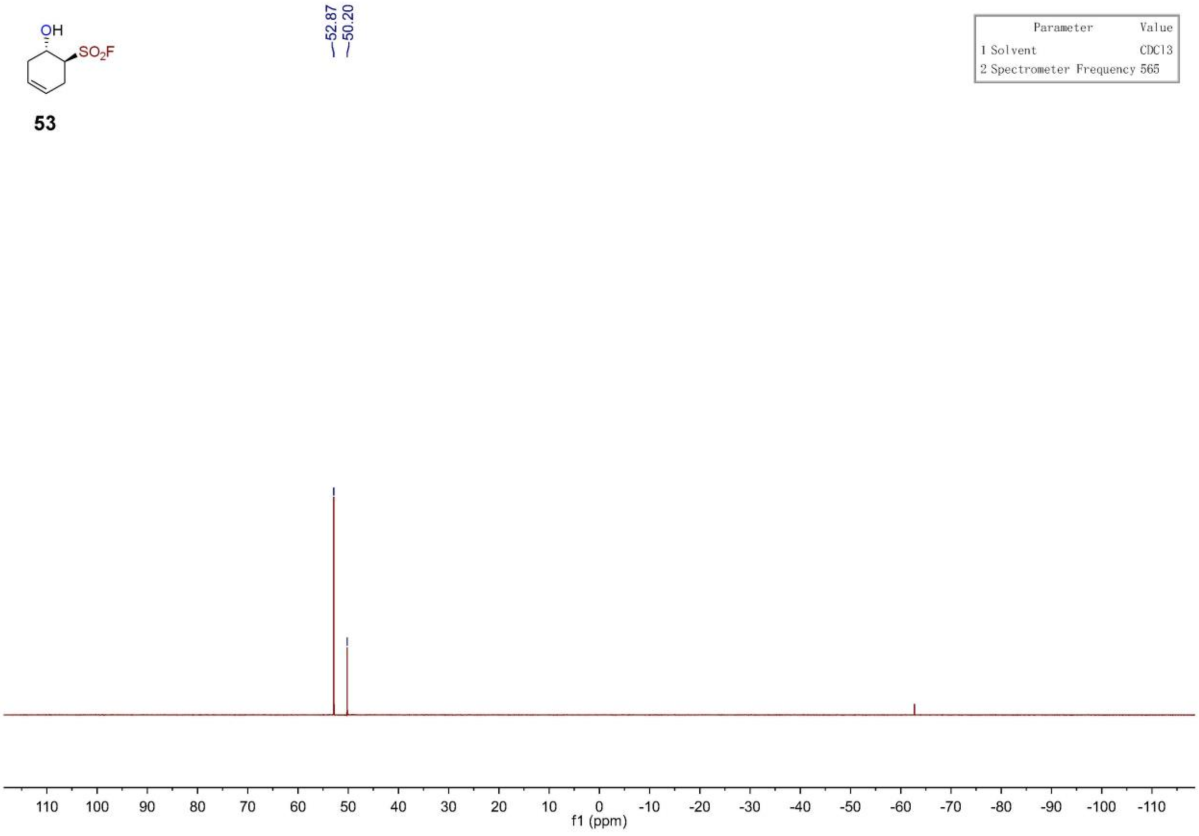
OH
SO2F
53
52.87
50.20
Parameter Value
1 Solvent CDCl3
2 Spectrometer Frequency 565
110 100 90 80 70 60 50 40 30 20 10 0 -10 -20 -30 -40 -50 -60 -70 -80 -90 -100 -110
f1 (ppm)

Supplementary Figure 72. ^{1}H, ^{13}C and ^{19}F NMR spectra of **54**.

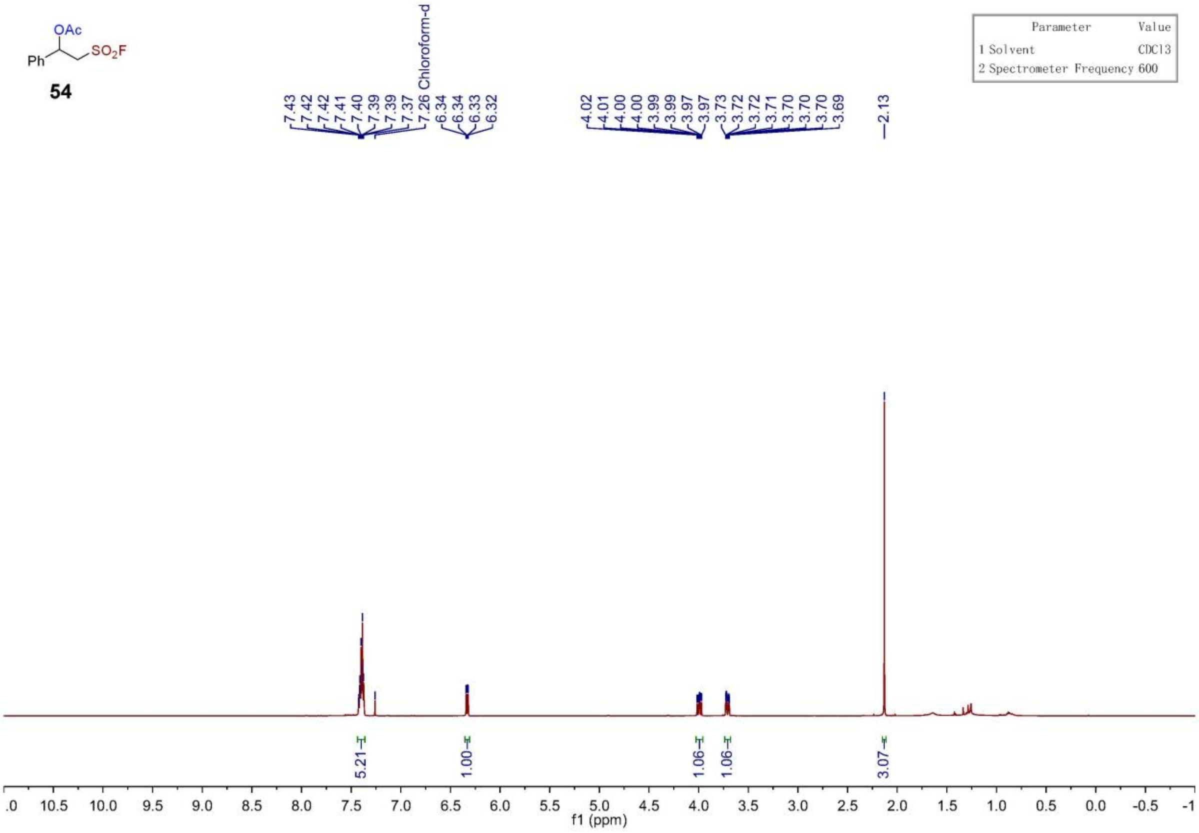

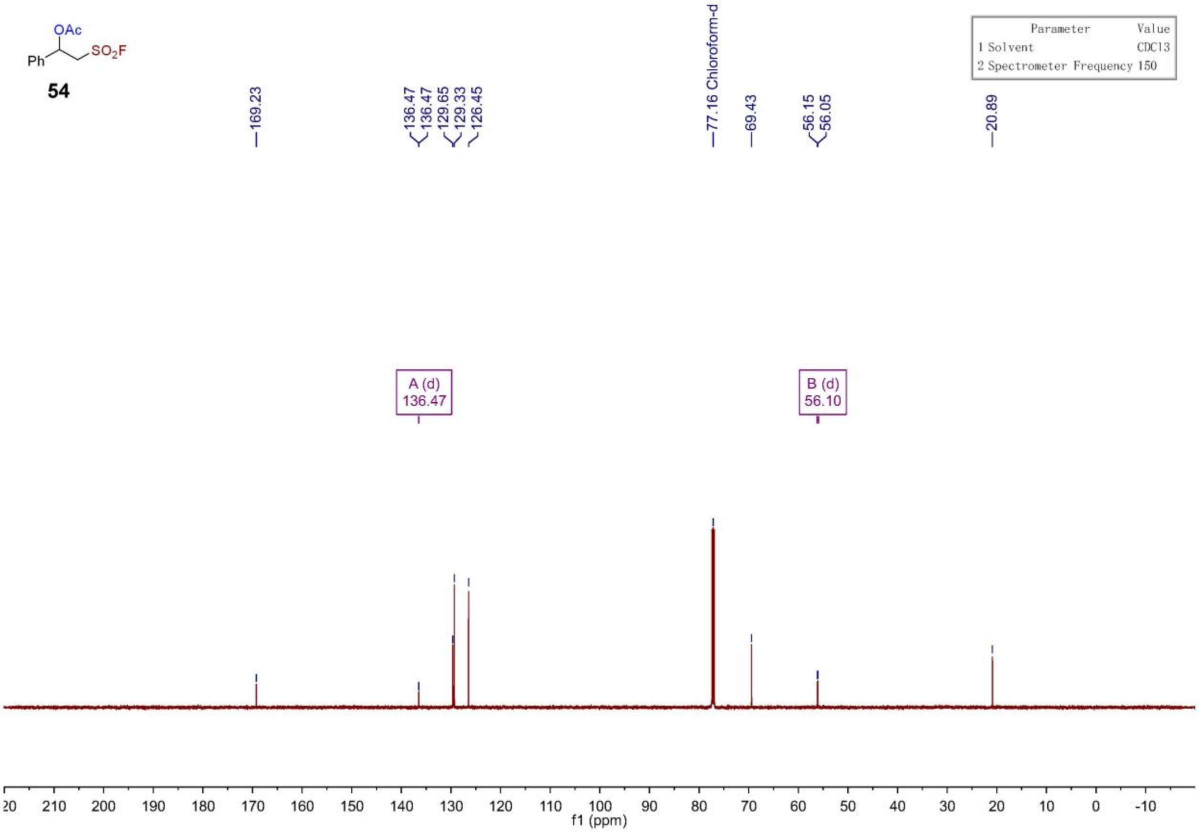

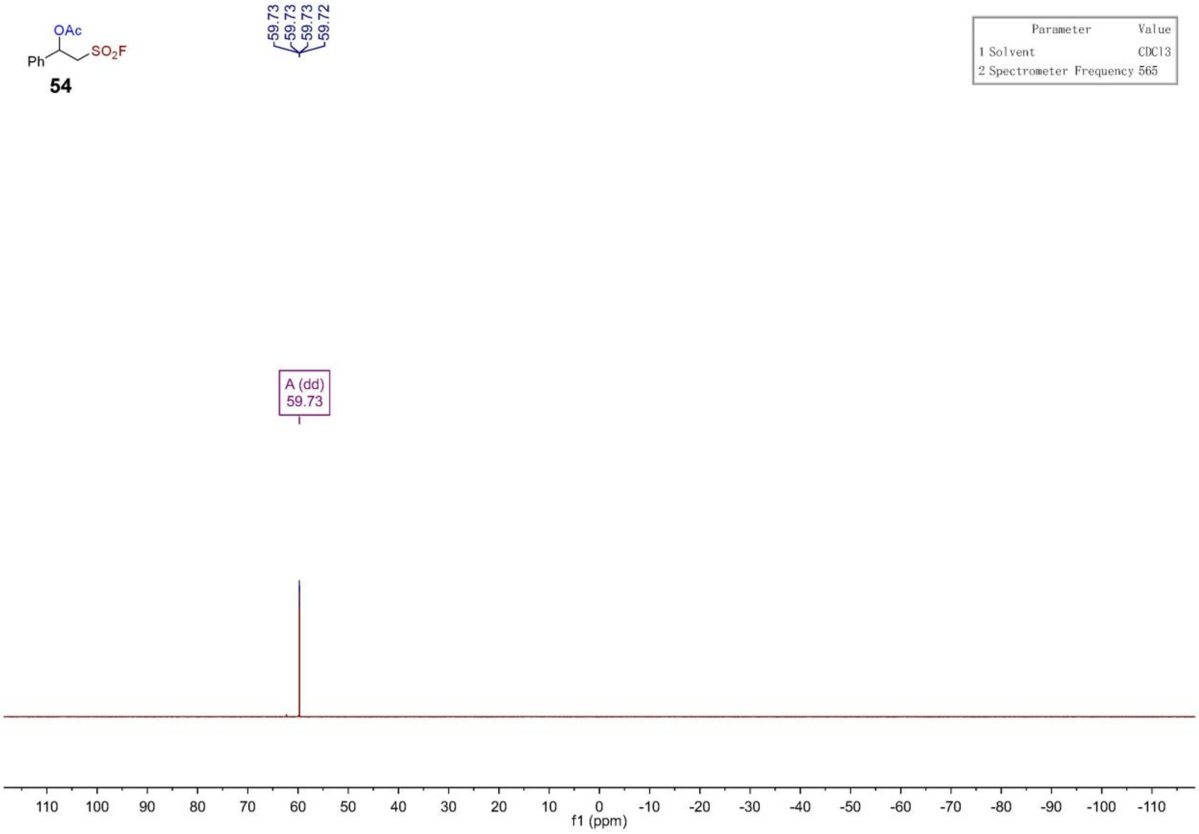

Supplementary Figure 73. ^{1}H, ^{13}C and ^{19}F NMR spectra of **55**.

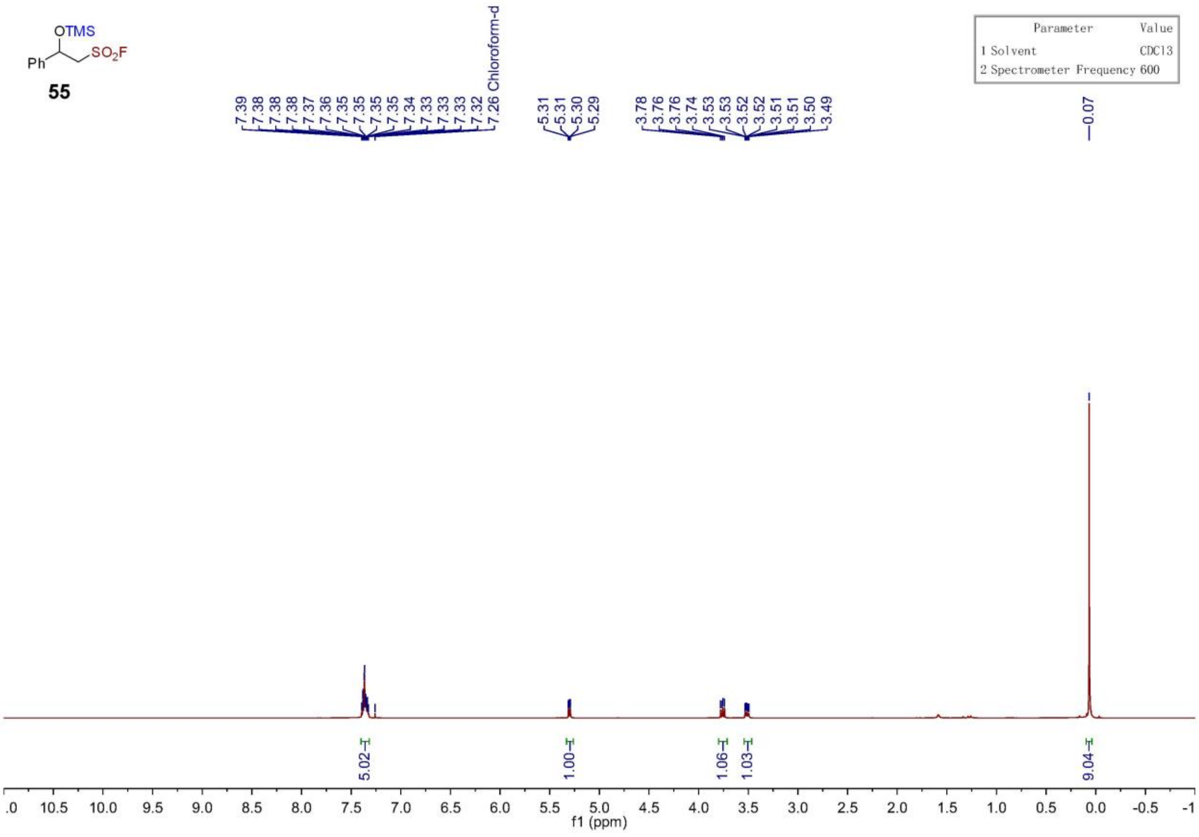

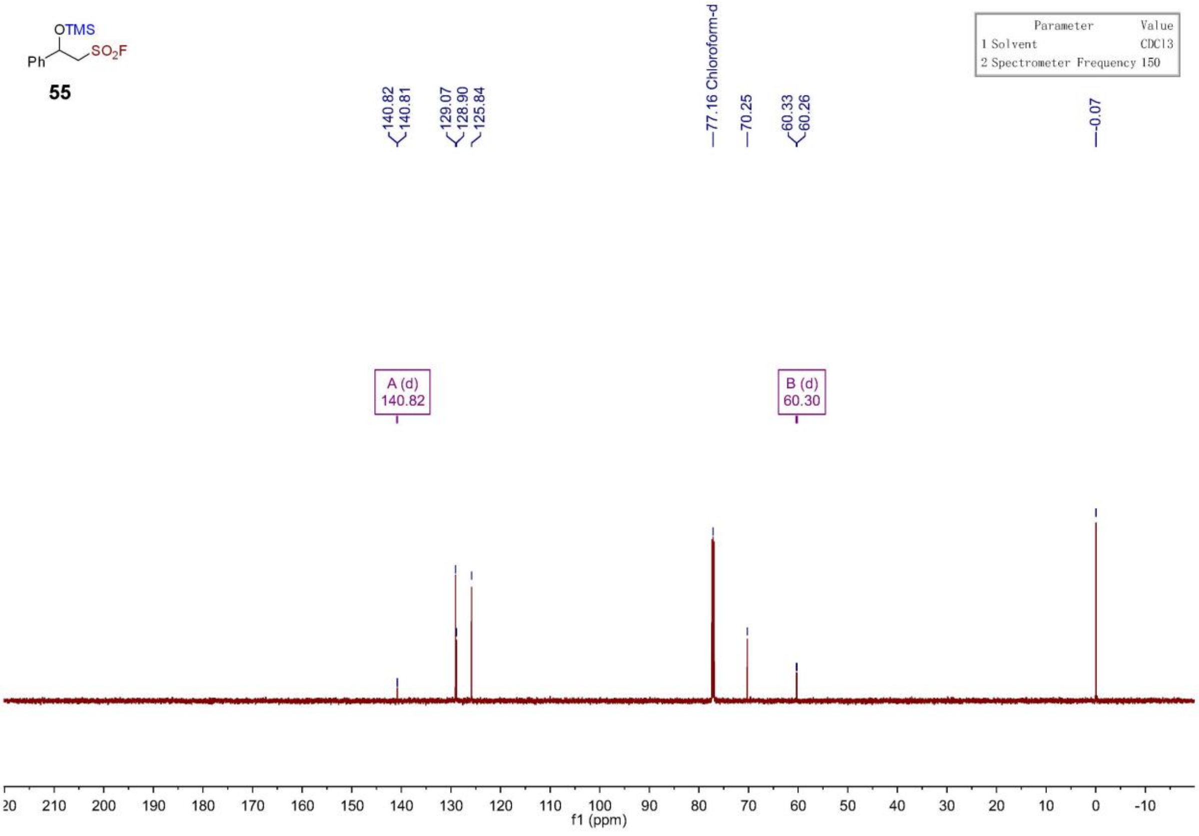
OTMS
Ph
SO2F
55
Parameter Value
1 Solvent CDCl3
2 Spectrometer Frequency 150
140.82
140.81
129.07
128.90
125.84
77.16 Chloroform-d
70.25
60.33
60.26
-0.07
A (d)
140.82
B (d)
60.30
f1 (ppm)

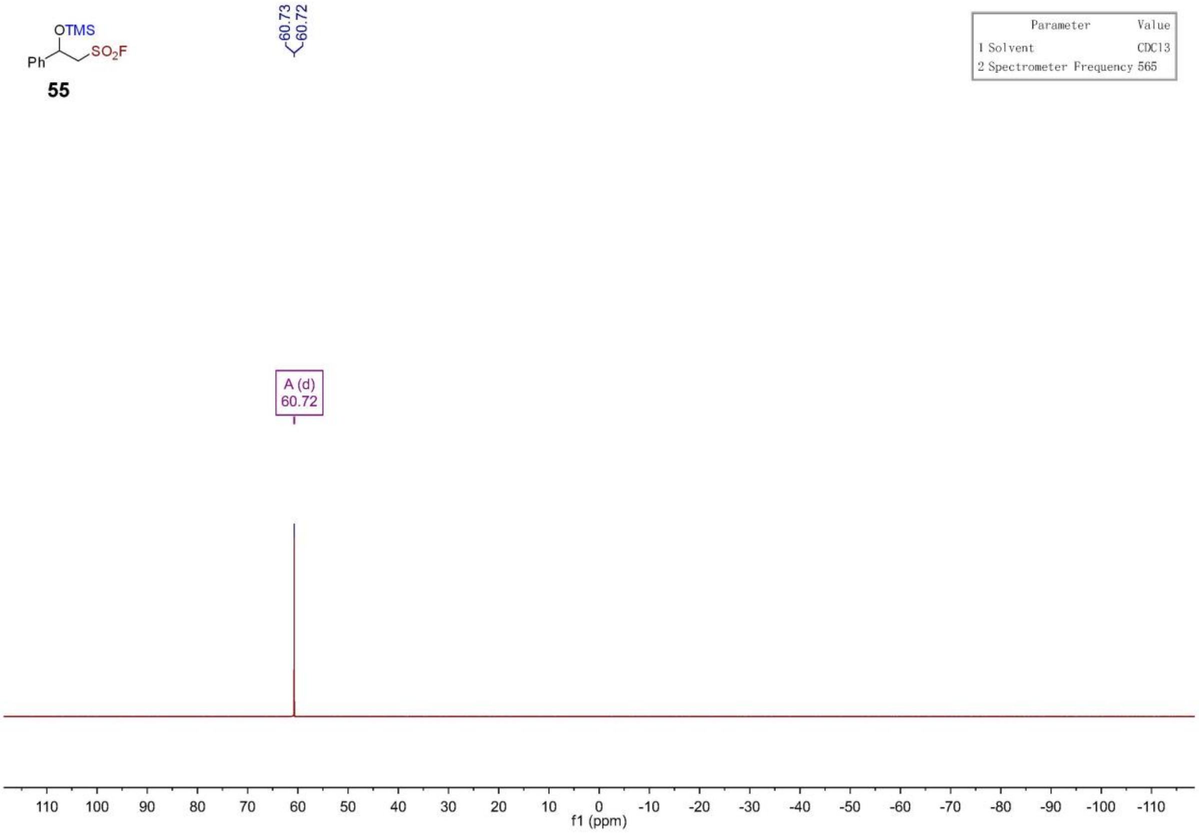
OTMS
Ph
SO2F
55
Parameter Value
1 Solvent CDCl3
2 Spectrometer Frequency 565
60.73
60.72
A (d)
60.72
f1 (ppm)

Supplementary Figure 74. ^{1}H NMR spectra of **4**.

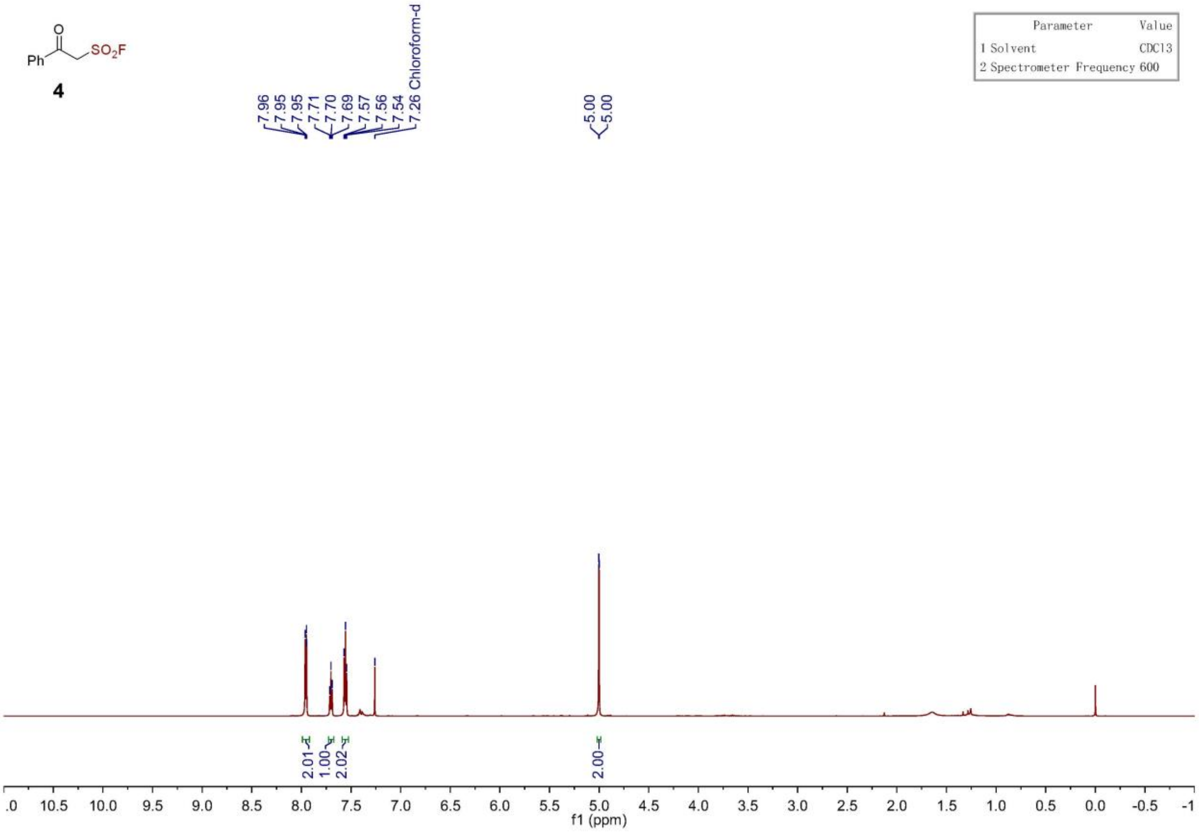

Supplementary Figure 75. ^{1}H, ^{13}C and ^{19}F NMR spectra of **56**.

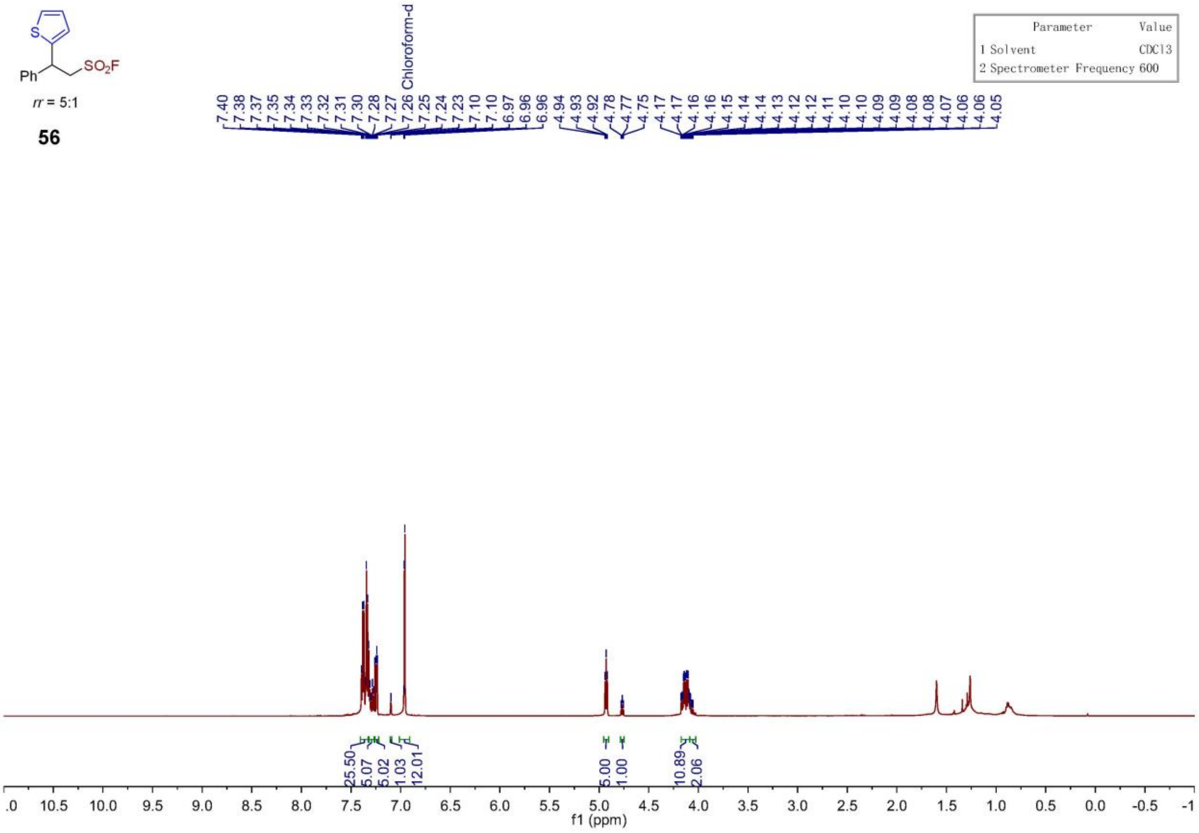

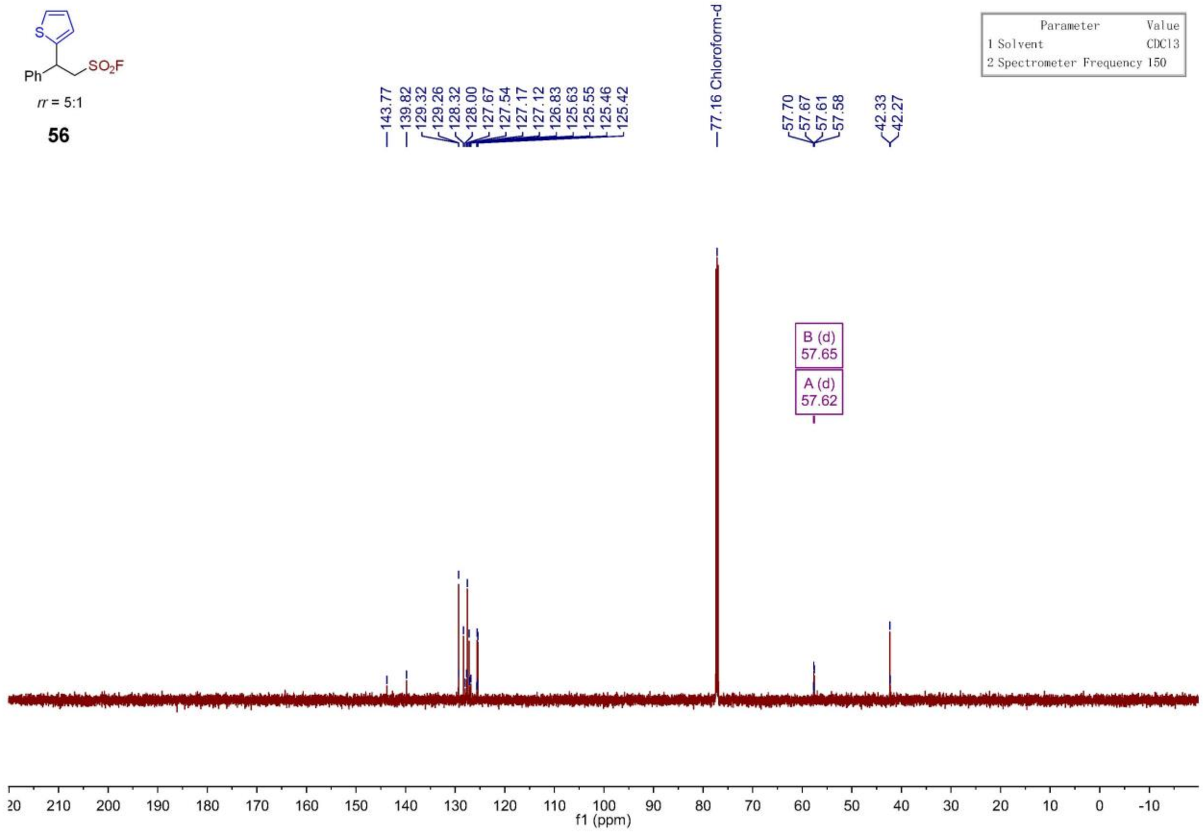
S
Ph
SO2F
rr = 5:1
56
143.77
139.82
129.32
129.26
128.32
128.00
127.67
127.54
127.17
127.12
126.83
125.63
125.55
125.46
125.42
77.16 Chloroform-d
57.70
57.67
57.61
57.58
42.33
42.27
Parameter Value
1 Solvent CDCl3
2 Spectrometer Frequency 150
B (d) 57.65
A (d) 57.62
f1 (ppm)

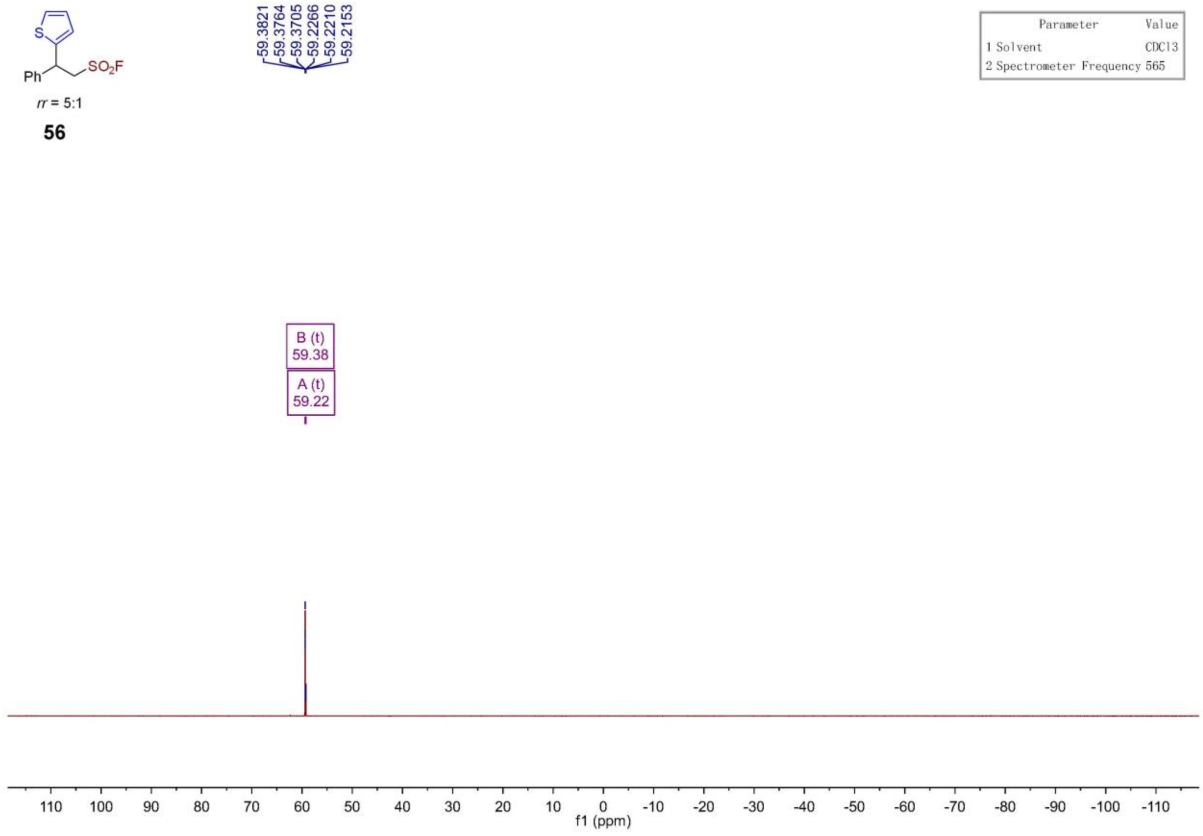
S
Ph
SO2F
rr = 5:1
56
59.3821
59.3764
59.3705
59.2266
59.2210
59.2153
Parameter Value
1 Solvent CDCl3
2 Spectrometer Frequency 565
B (t) 59.38
A (t) 59.22
f1 (ppm)

Supplementary Figure 76. ^{1}H and ^{19}F NMR spectra of **57**.

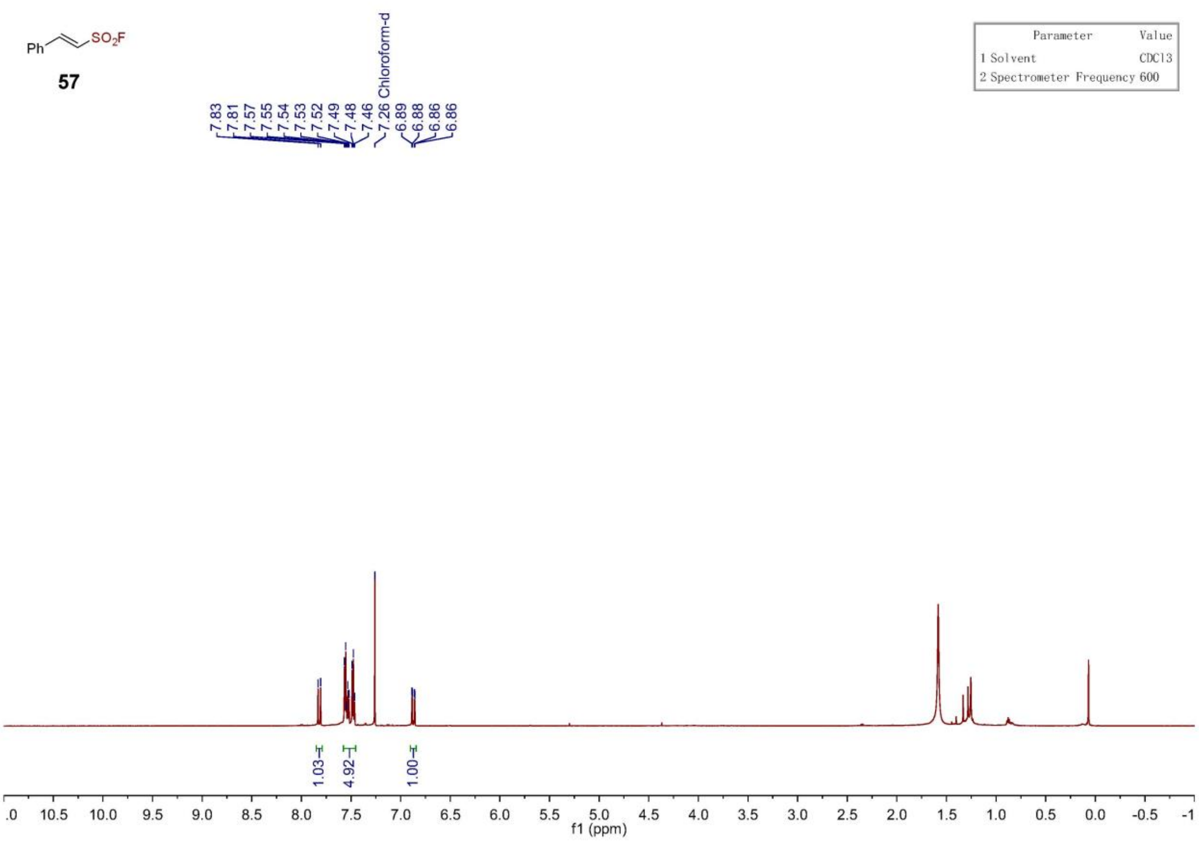

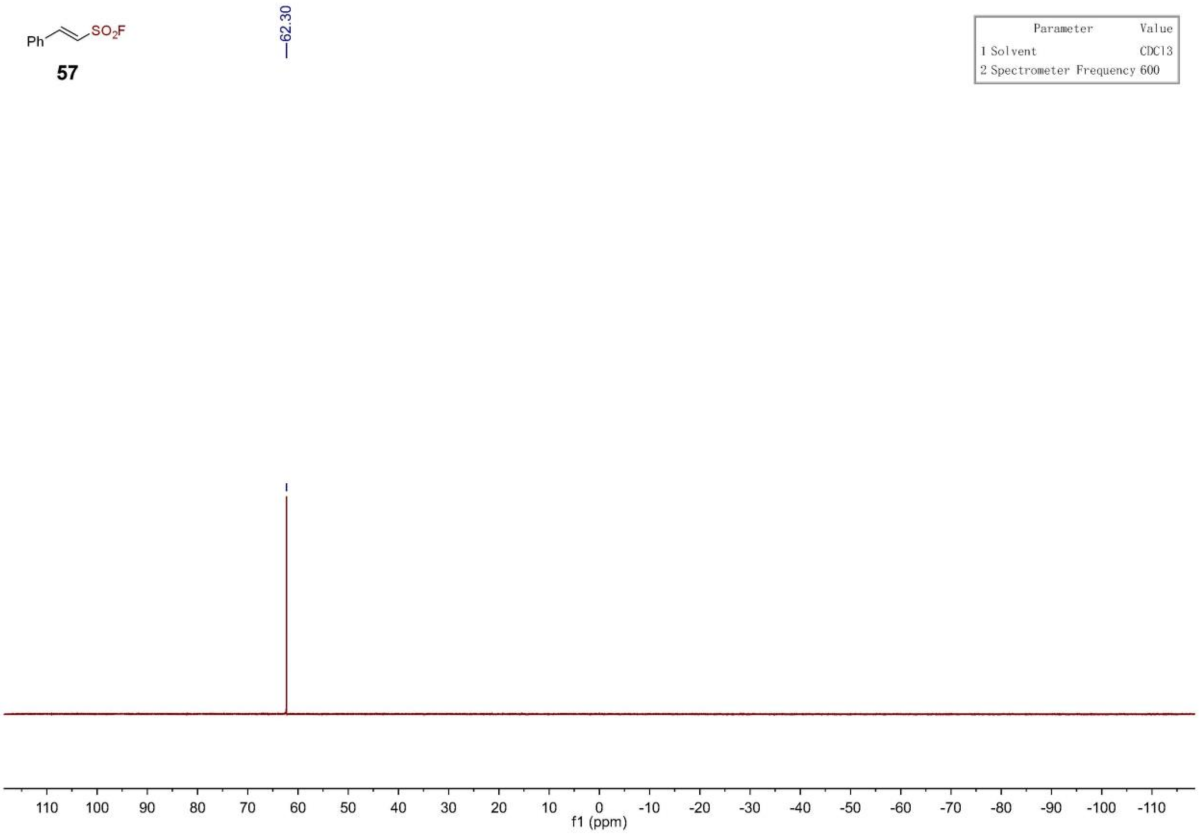

Supplementary Figure 77. ^{1}H NMR spectra of **59**.

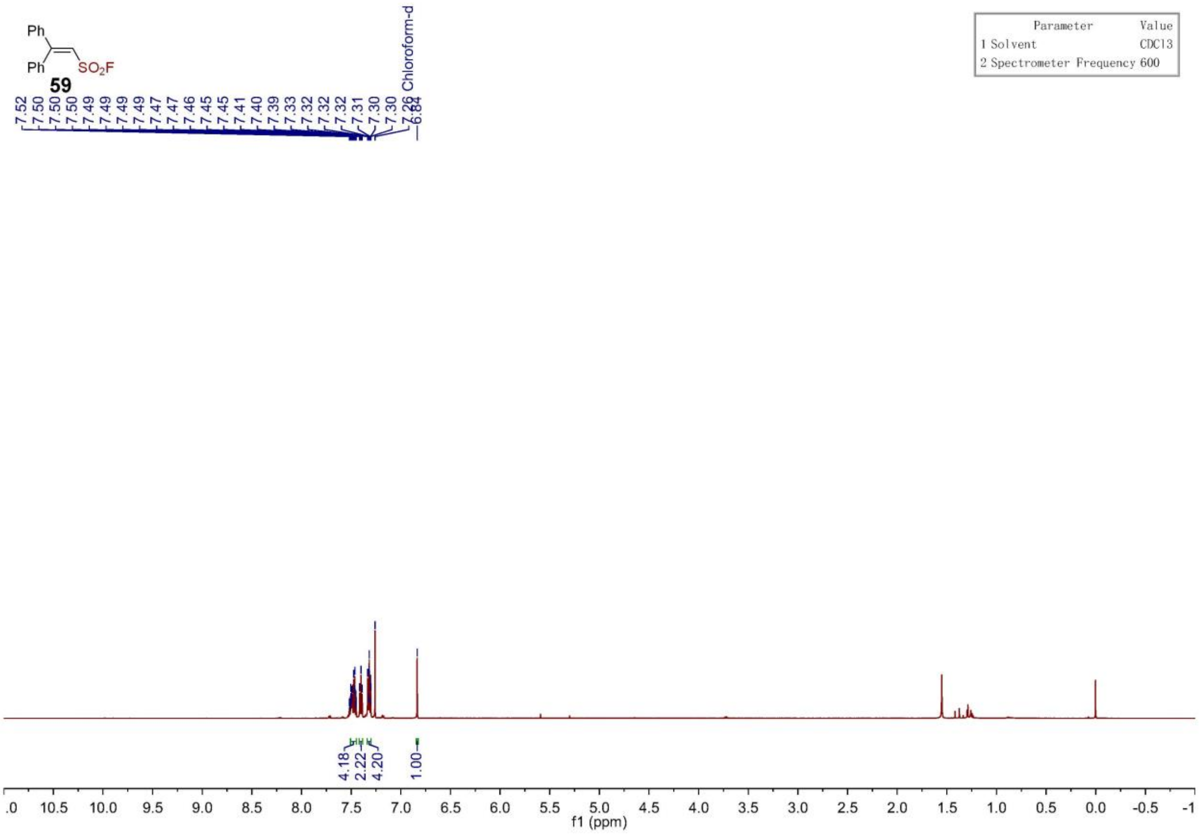

Supplementary Figure 78. ^{1}H NMR spectra of **S1**.

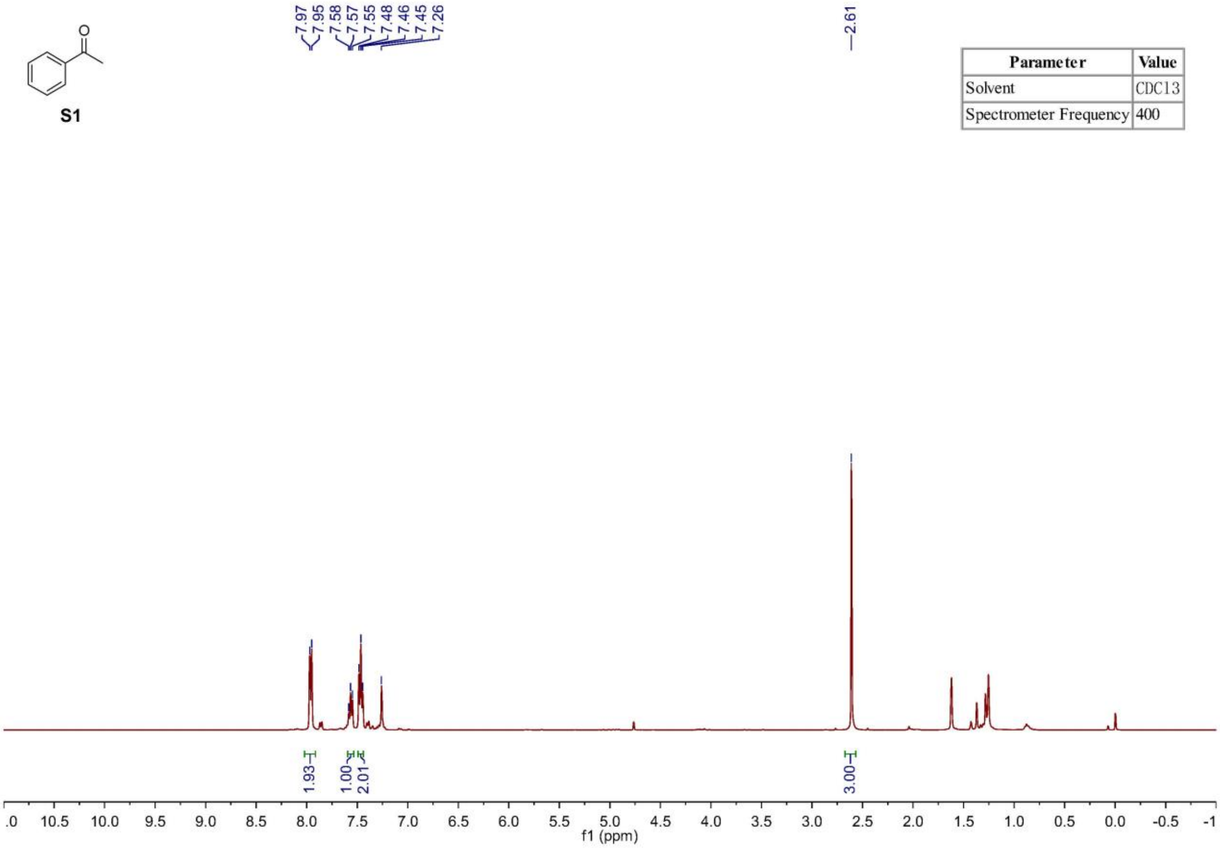

Supplementary Figure 79. ^{1}H, ^{13}C and ^{19}F NMR spectra of **S3**.

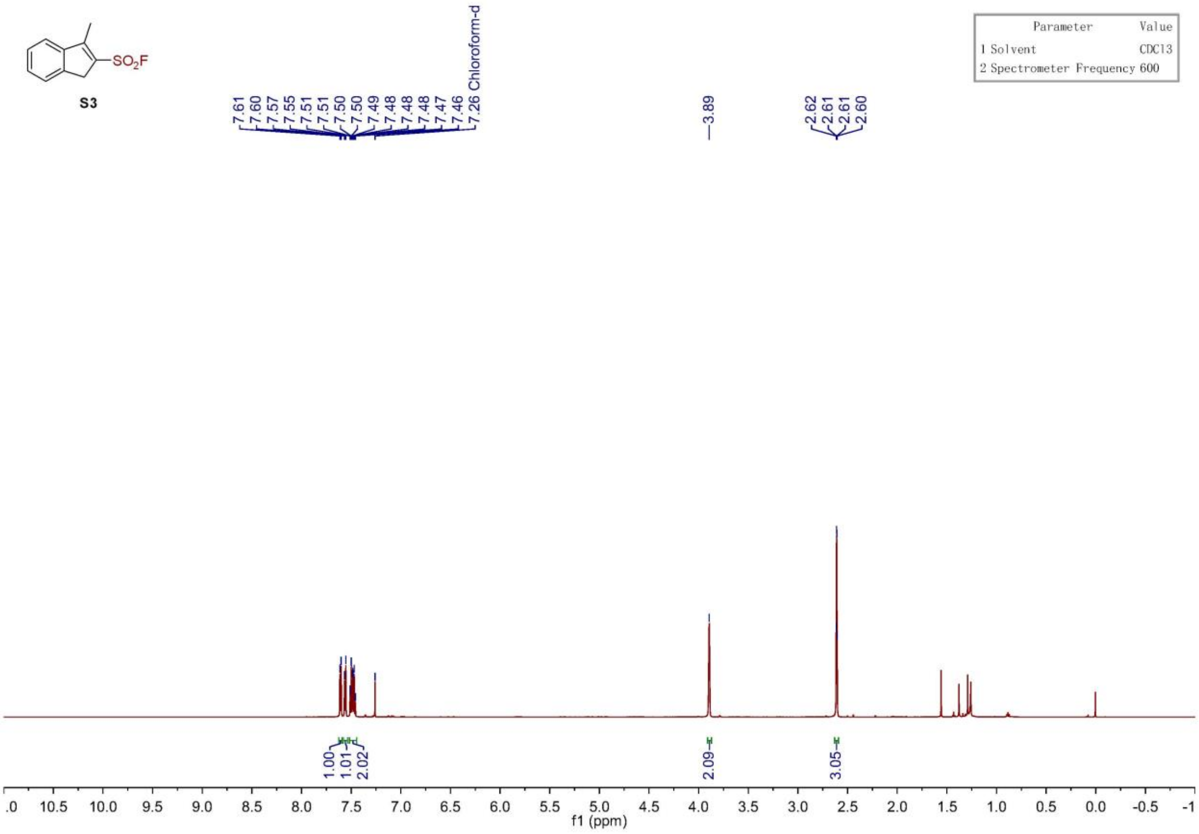

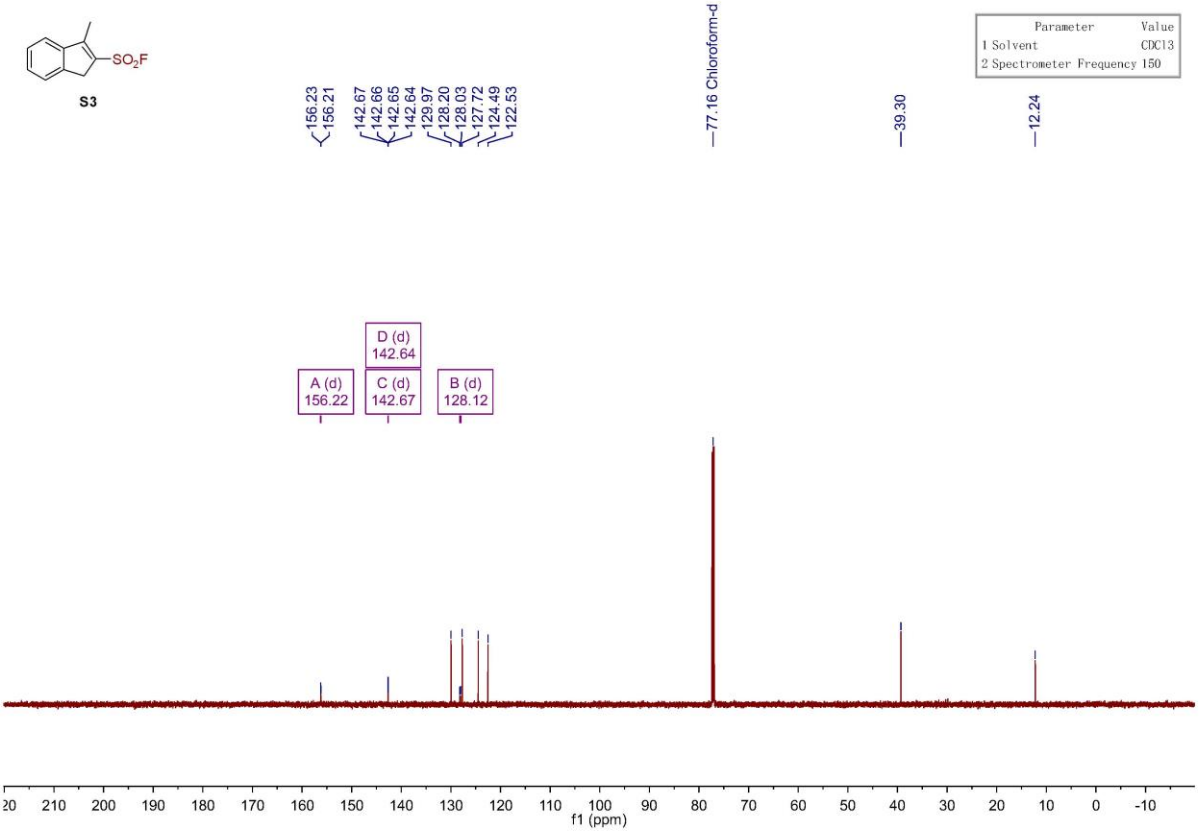

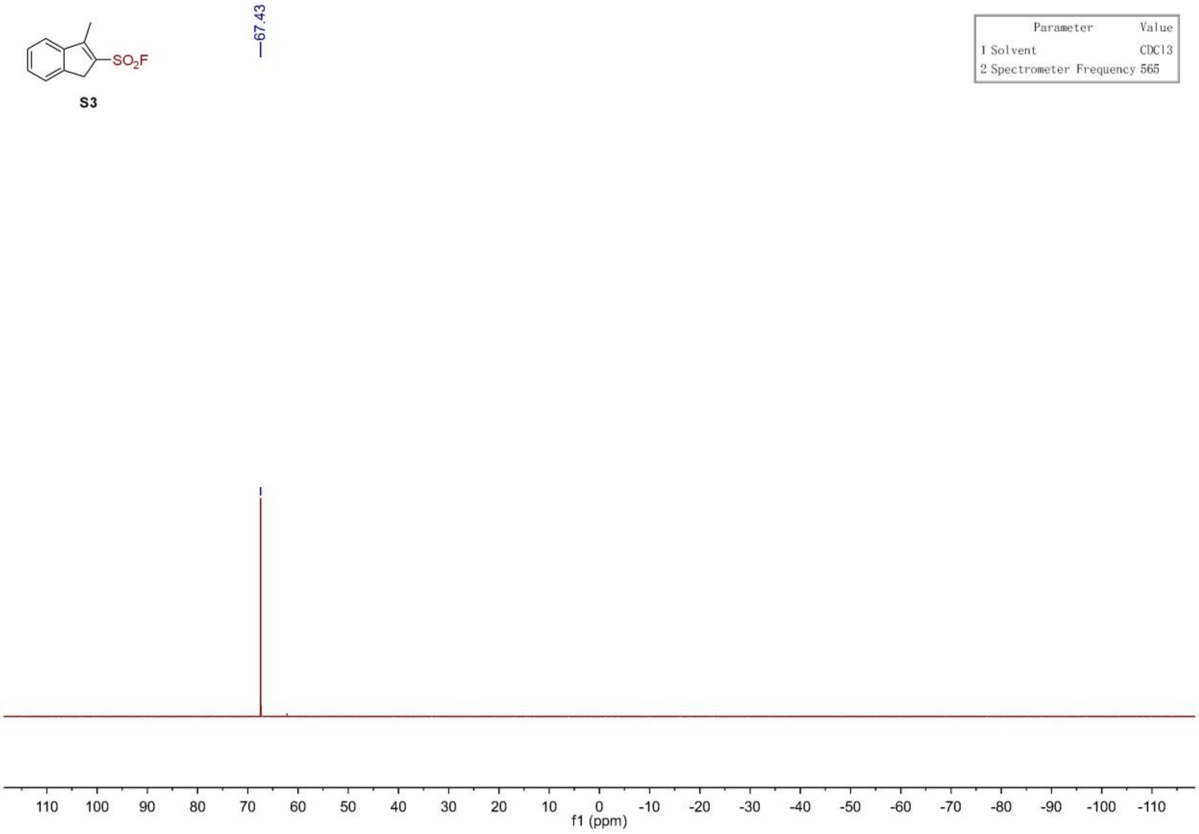

Supplementary Figure 80. ^{1}H, ^{13}C and ^{19}F NMR spectra of **S8**.

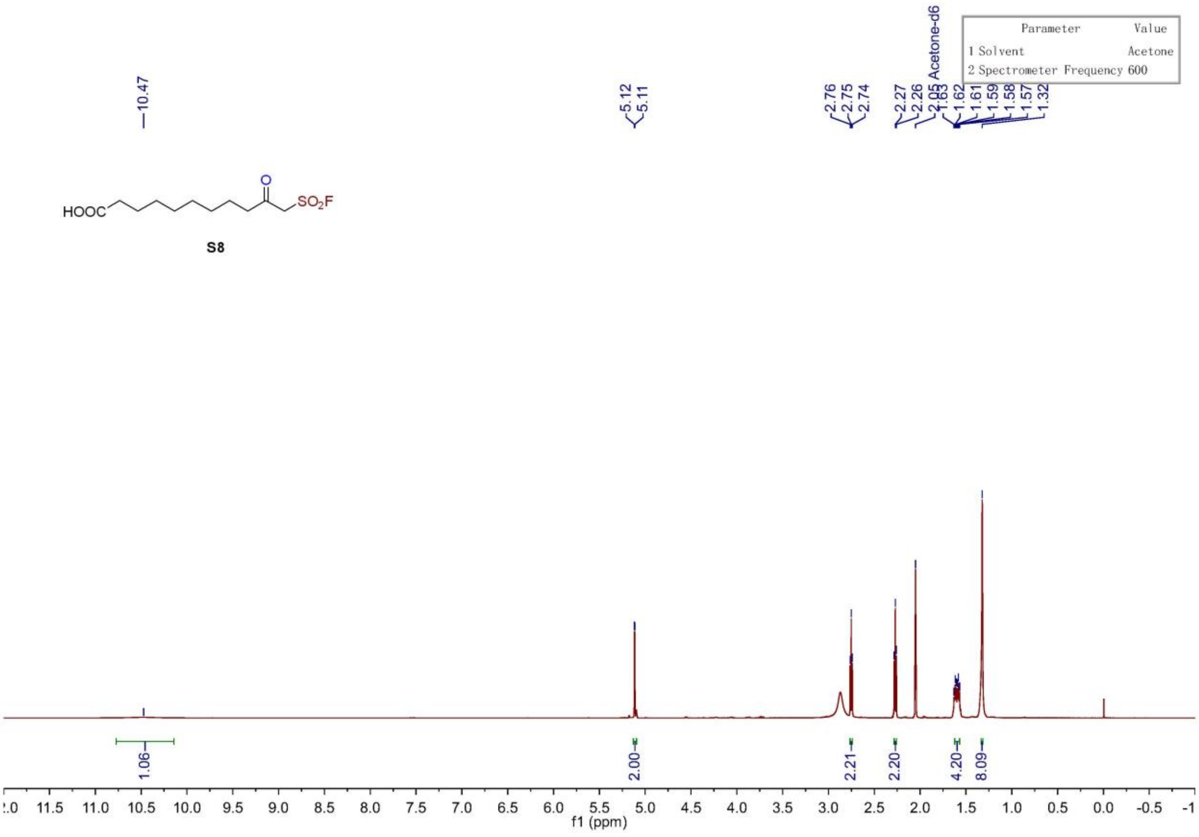

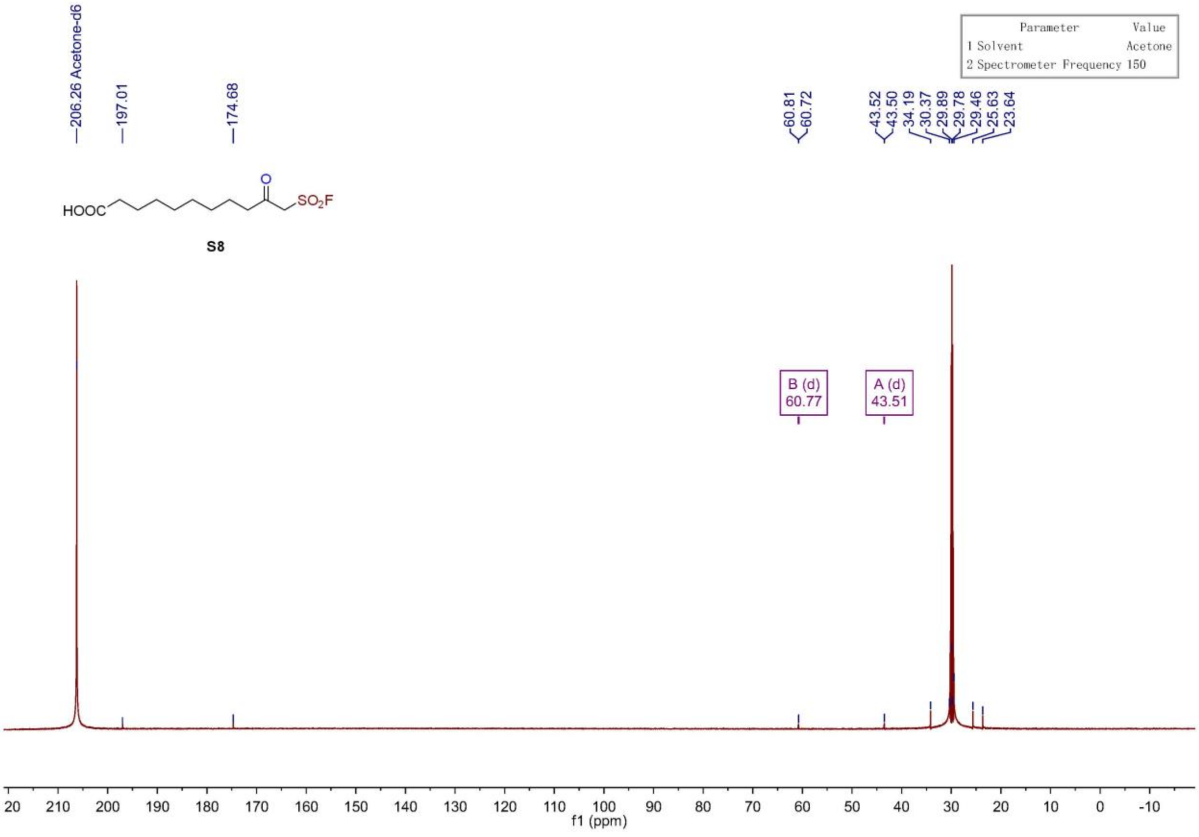
Parameter Value
1 Solvent Acetone
2 Spectrometer Frequency 150
206.26 Acetone-d6
197.01
174.68
60.81
60.72
43.52
43.50
34.19
30.37
29.89
29.78
29.46
25.63
23.64
HOOC
O
SO2F
S8
B (d)
60.77
A (d)
43.51
f1 (ppm)

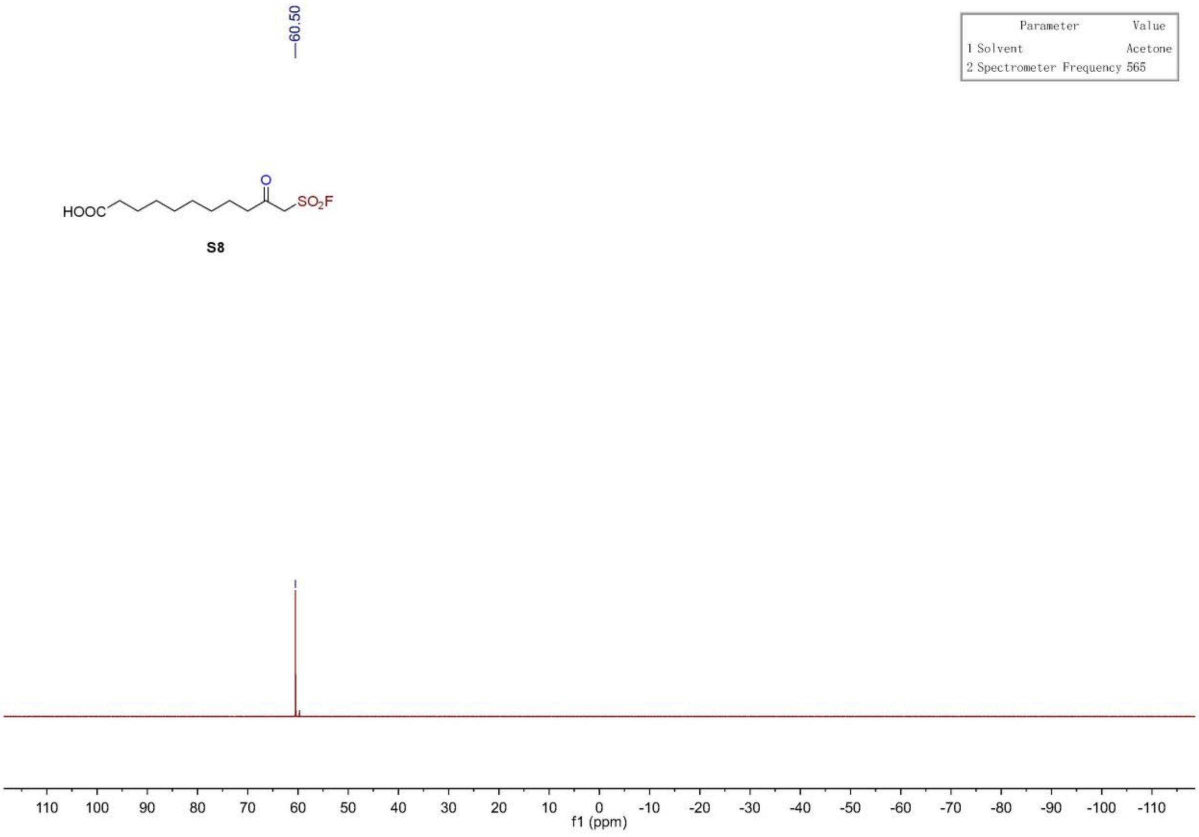
Parameter Value
1 Solvent Acetone
2 Spectrometer Frequency 565
60.50
HOOC
O
SO2F
S8
f1 (ppm)

3. Supplementary references

1. Prakash Reddy, V., Bellew, D. R., and Prakash, G. K. S. A convenient preparation of sulfuryl chloride fluoride. *J. Fluor. Chem.* **56**, 195-197 (1992).

2. Granados, A., Dhungana, R. K., Sharique, M., Majhi, J., and Molander, G. A. From styrenes to fluorinated benzyl bromides: a photoinduced difunctionalization via atom transfer radical addition. *Org. Lett.* **24**, 4750-4755 (2022).

3. Chen, G., Xu, J., Xiong, B., Song, H., Zhang, X., Ma, X., and Lian, Z. Copper-catalyzed trifluoromethylthio-arylsulfonylation of styrene derivatives via the insertion of sulfur dioxide. *Org. Lett.* **24**, 1207-1212 (2022).

4. Mato, M., Montesinos-Magraner, M., Sugranyes, A. R., and Echavarren, A. M. Rh(II)-catalyzed alkynylcyclopropanation of alkenes by decarbenation of alkynylcycloheptatrienes. *J. Am. Chem. Soc*. **143***,* 10760-10769 (2021).

5. Cheng, Y.-Y., Yu, J.-X., Lei, T., Hou, H.-Y., Chen, B., Tung, C.-H., and Wu, L.-Z. Direct 1,2-dicarbonylation of alkenes towards 1,4-diketones via photocatalysis. *Angew. Chem. Int. Ed.* **60**, 26822-26828 (2021).

6. Vaith, J., Rodina, D., Spaulding, G. C., and Paradine, S. M. Pd-catalyzed heteroannulation using N-arylureas as a sterically undemanding ligand platform. *J. Am. Chem. Soc*. **144**, 6667-6673 (2022).

7. Chen, Y.-X., He, J.-T., Wu, M.-C., Liu, Z.-L., Tang, K., Xia, P.-J., Chen, K., Xiang, H.-Y., Chen, X.-Q., Yang, H. Photochemical organocatalytic aerobic cleavage of C═C bonds enabled by charge-transfer complex formation. *Org. Lett*. 24, 3920-3925 (2022).

8. Chen, D., Nie, X., Feng, Q., Zhang, Y., Wang, Y., Wang, Q., Huang, L., Huang, S., and Liao, S. Electrochemical oxo-fluorosulfonylation of alkynes under air: facile access to *β*-keto sulfonyl fluorides. *Angew. Chem. Int. Ed.* **60**, 27271-27276 (2021).

9. Nie X., Xu T., Song J., Devaraj A., Zhang B., Chen Y., and Liao S. Radical fluorosulfonylation: accessing alkenyl sulfonyl fluorides from alkenes. *Angew. Chem*. **133**, 3869-4423 (2021).

10. Cummings, S. P., Le, T.-N., Fernandez, G. E., Quiambao, L. G., and Stokes, B. J. Tetrahydroxydiboron-mediated palladium-catalyzed transfer hydrogenation and deuteriation of alkenes and alkynes using water as the stoichiometric H or D atom donor. *J. Am. Chem. Soc*. **138**, 6107- 6110 (2016).

11. Kurouchi, H., Sugimoto, H., Otani, Y., Ohwada, T. Cyclization of arylacetoacetates to indene and dihydronaphthalene derivatives in strong acids. evidence for involvement of further protonation of O,O-diprotonated β-ketoester, leading to enhancement of cyclization. *J. Am. Chem. Soc*. **132**, 807–815 (2010).

12. Feng, Q., Fu, Y., Zheng, Y., Liao, S., Huang, S. Electrochemical synthesis of β-keto sulfonyl fluorides via radical fluorosulfonylation of vinyl triflates. *Org. Lett*. **24**, 3702-3706 (2022).

13. Wang, P., Li, S.-J., Zhang, H., Yang, Na., Liao, S. Photo-organocatalytic synthesis of β-keto sulfonyl fluorides via radical fluorosulfonylation of vinyl acetates. *Synlett*; **34**, 471-476 (2023).
